# Bioinformatics and System Biology Approach to Identify the Influences of COVID-19 on Rheumatoid Arthritis

**DOI:** 10.3389/fimmu.2022.860676

**Published:** 2022-04-07

**Authors:** Huan Hu, Nana Tang, Facai Zhang, Li Li, Long Li

**Affiliations:** ^1^ Department of Rheumatology and Immunology, The Affiliated Hospital of Guizhou Medical University, Guiyang, China; ^2^ Clinical Medical College, Guizhou Medical University, Guiyang, China; ^3^ Medical Intensive Care Unit, The Affiliated Hospital of Guizhou Medical University, Guiyang, China; ^4^ Department of Urology/Institute of Urology, West China Hospital, Sichuan University, Chengdu, China

**Keywords:** COVID-19, rheumatoid arthritis, differentially expressed genes, proteinprotein interaction, drug molecule

## Abstract

**Background:**

Severe coronavirus disease 2019 (COVID -19) has led to a rapid increase in mortality worldwide. Rheumatoid arthritis (RA) was a high-risk factor for severe acute respiratory syndrome coronavirus 2 (SARS-CoV-2) infection, whereas the molecular mechanisms underlying RA and CVOID-19 are not well understood. The objectives of this study were to analyze potential molecular mechanisms and identify potential drugs for the treatment of COVID-19 and RA using bioinformatics and a systems biology approach.

**Methods:**

Two Differentially expressed genes (DEGs) sets extracted from GSE171110 and GSE1775544 datasets were intersected to generate common DEGs, which were used for functional enrichment, pathway analysis, and candidate drugs analysis.

**Results:**

A total of 103 common DEGs were identified in the two datasets between RA and COVID-19. A protein-protein interaction (PPI) was constructed using various combinatorial statistical methods and bioinformatics tools. Subsequently, hub genes and essential modules were identified from the PPI network. In addition, we performed functional analysis and pathway analysis under ontological conditions and found that there was common association between RA and progression of COVID-19 infection. Finally, transcription factor-gene interactions, protein-drug interactions, and DEGs-miRNAs coregulatory networks with common DEGs were also identified in the datasets.

**Conclusion:**

We successfully identified the top 10 hub genes that could serve as novel targeted therapy for COVID-19 and screened out some potential drugs useful for COVID-19 patients with RA.

## Introduction

The Coronavirus Disease 2019 (COVID-19) pandemic is caused by severe acute respiratory syndrome coronavirus 2 (SARS-CoV-2) (1–3). SARS-CoV-2 virus spreads rapidly through respiratory droplets and aerosols generated by patients’ coughing and sneezing (4). The most common symptoms of COVID-19 are fever, dry cough and sore throat, fatigue, and diarrhea, according to a recent WHO report conducted in China (5–7), while musculoskeletal symptoms, like muscular soreness, arthralgia may also occur with coronavirus infection (8, 9). Although the symptoms of COVID-19, especially Omicron variation, are usually similar to a cold or flu, severe pneumonia or acute respiratory distress syndrome (ARDS) may occur for COVID-19 patients with autoimmune diseases (10). SARS-CoV-2 could lead to uncontrolled immune activation and cytokine response in lung tissue. Immune dysregulation is commonly observed in patients with COVID-19 (11). Given the pandemic of COVID-19 and incidence of rheumatoid arthritis (RA), it brings rheumatologists’ attention that whether patients with RA suffering from COVID-19 will lead to further exacerbation of RA as well as worse COVID-19 outcomes (11–14).

Several studies have investigated a possible association between respiratory viral infections and the development of RA (15). A study by Kerea suggested that respiratory viral infection might be a novel environmental risk factor for RA (16). Compared with the general population, the risk of infection increased in rheumatoid arthritis (10), for the overall immune system damage in typical autoimmune diseases, the side effects of corticosteroids and immunosuppressants (17). Moreover, infections was also a tough problem for RA patients, which could contribute to the disease progression (18). Up to date, there were only two studies reporting clinical outcomes for RA patients with COVID-19 (19, 20), which showed that RA patients with COVID-19 had a higher risk of hospitalization or death than non-RA patients. Therefore, it was essential to understand how COVID-19 impacts on these RA patients and to find out potential effective drugs for RA patients with COVID-19, which might reduce the risk of hospitalization or death. Herein, we succeed to use bioinformatics and a systems biology approach to analyze potential molecular mechanism and identify some drugs that might be useful for the treatment of COVID-19 and RA.

## Materials And Methods

### Data Collection

We used both microarray and RNA-seq datasets to uncover common genetic correlation between COVID-19 and RA from the National Center for Biotechnology Information (NCBI) database GEO (https://www.ncbi.nlm.nih.gov/geo/) (21). The GEO accession ID of the COVID-19 dataset was GSE171110, which contained whole-blood RNA-seq profiling from 44 COVID-19 patients and 10 healthy donors using high throughput sequencing Illumina HiSeq 2500 platform for RNA sequence extraction (22). Similarly, the RA dataset (GEO accession ID: GSE17755) consisted of 112 RA blood samples, 8 healthy children and 45 healthy individuals, which were sequenced using microarrays called Hitachisoft AceGene Human Oligo Chip 30K Chip Version (23). The summarized information of the datasets is shown in **Table 1**.

**Table 1:** Overview of datasets with their geo-features and quantitative measurements in this analysis.

Disease name	GEO accession	GEO platform	Total DEGs count	Up regulatedDEGs count	Down regulatedDEGs count
COVID-19	GSE171110	GPL16791	3803	1783	2020
RA	GSE17755	GPL1291	757	352	405

### Identification of Differentially Expressed Genes (DEGs) and Common DEGs Among RA and COVID-19

The primary purpose of this analysis was to acquire DEGs for the datasets GSE171110 and GSE17755. We utilized the “limma” package in R software (v 4.0.2) to select out DEGs with |log Fold-Change| ≥ 1 and False Discover Rate (FDR) <0.05 for GSE171110, and with |log Fold-Change| ≥ 0.5 and False Discover Rate (FDR) <0.05 for GSE17755, respectively. The common DEGs of GSE171110 and GSE17755 were obtained using Jvenn, an online Venn analysis tool (24).

### Gene Ontology and Pathway Enrichment Analysis

We used the “clusterProfiler” package in R software to investigated the possible function and pathways in these DEGs by Gene Ontology (GO) enrichment analysis and Kyoto Encyclopedia of Genes and Genomes (KEGG) pathway analysis and characterized biological mechanisms and signaling pathways of the common DEGs. For quantifying the top listed functional items and pathways, the *P-*value < 0.05 and Q value < 0.25 were used as standardized metric.

### Protein-Protein Interaction (PPI) Network Analysis

PPI is a major component of the cellular biochemical response network. Evaluation PPI network and its functions is essential goal for understanding and gaining insight into cellular machine processes in both cellular and molecular systems biology (25). Differentially expressed genes were also uploaded to STRING (www.string-db.org) (version 11.0) for critical assessment and integration of protein-protein interactions, including direct (physical) and indirect (functional) associations. A combined score larger than 0.4 was used to construct the PPI network of frequent DEGs in this experiment. For visual representation and additional experimental testing of the PPI network, Cytoscape (v.3.7.1) was used to consume our PPI network.

### Hub Gene Extraction and Submodule Analysis

To anticipate the hub genes, we utilized Cytoscape plug-in CytoHubba for ranking and examining significant nodes in the PPI network modules. Cytohubba (http://apps.cytoscape.org/apps/cytohubba) is a significant Cytoscape application that allows users to assess and extract central, hidden, or targeted aspects of a biological network based on network metrics (26). In addition, we performed validation of the hub gene by ROC analysis and provided the figure of ROC analysis in **Figure S1** of the supplemental material. The top 10 genes were identified depend on the degree algorithm, a commonly used centrality criteria, and their ranks were presented *via* gradients from red to yellow in plots. We used Cytohubba’s proximate neighborhood ranking features to rank the shortest accessible paths between hub genes.

### Recognition of Common DEGs Associated Transcription Factors and MiRNAs

Transcription factors (TFs) were the proteins that identified specific DNA sequences to control chromatin and transcription. They formed a complex system that controlled the expression of the genome and were therefore essential for molecular understanding (27). JASPAR (http://jaspar.genereg.net) was an open-access resource of curated, non-redundant transcription factor (TF) binding profiles stored as position frequency matrices (PFMs) for TF flexible models (TFFMs) for TFs across numerous species in 6 different taxonomic groups (28). We used the NetworkAnalyst function to find topologically plausible TFs from the JASPAR database that bind to our common DEGs. NetworkAnalyst was launched in 2014 to address the critical need to analyze gene expression data in the context of protein-protein interaction (PPI) networks to gain insights into biological mechanisms, roles, and interpretations (29, 30). In addition, Interaction analysis of target gene-miRNA were incorporated to detect miRNAs which could succeed to attach to target gene transcripts and negatively affect protein expression by disrupting the stability and translational efficiency of targeted mature messenger RNAs (31). MiRTarBase, the most famous comprehensive miRNA-target interaction databases, was employed to explore the miRNAs which interacted with the common DEGs *via* the tool of networkAnalyst (32). after that, we identified and extracted them from gene—miRNA interaction. Both TF-gene and miRNA-gene interaction networks were displayed in Cytoscape.

### Evaluation of Applicant Drugs

One of the most important aspects of this research was the prediction of protein-drug interactions (PDI) or the identification of pharmacological molecules. We used Enrichr and the Drug Signatures Database (DSigDB) to study the drug molecular based on the DEGs of COVID-19 and RA. Enrichr (http://amp.pharm.mssm.edu/Enrichr) was a comprehensive resource for curated gene sets and a search engine that accumulated biological knowledge for further biological discovery (33). DSigDB, a new gene set resource that related drugs/compounds and their target genes for gene set enrichment analysis. The DSigDB database was accessed through Enrichr under the Diseases/Drugs function (34).

### Gene-Disease Association Analysis

DisGeNET (http://www.disgenet.org/), a knowledge management platform for integrating and standardizing data on disease-associated genes and variants from multiple sources. The current version of DisGeNET contained more than 24,000 diseases and traits, 17,000 genes, and 117,000 genomic variants (35). It illustrated the growing understanding of human genetic diseases. We also used NetworkAnalyst to study gene-disease association to identify diseases and chronic problems associated with common DEGs (29, 30).

## Results

### Identification of DEGs and Common DEGs Between RA and COVID-19

**Figure 1:**
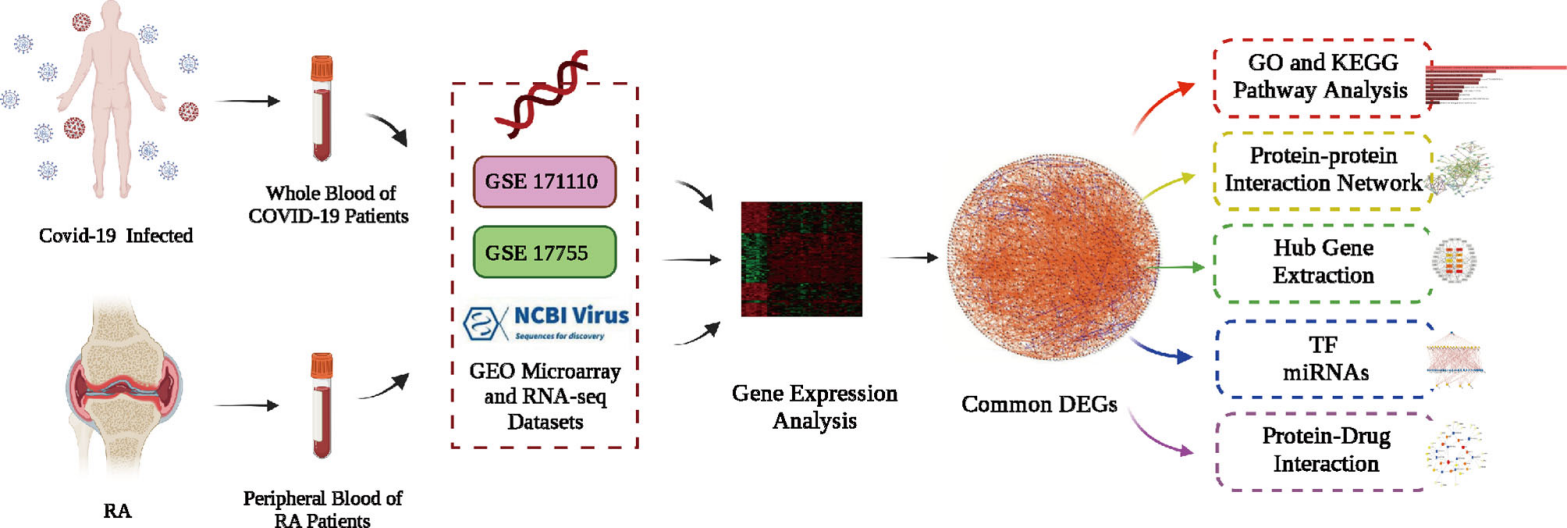
Principle scheme diagram of the whole workflow of this study.

**Figure 2:**
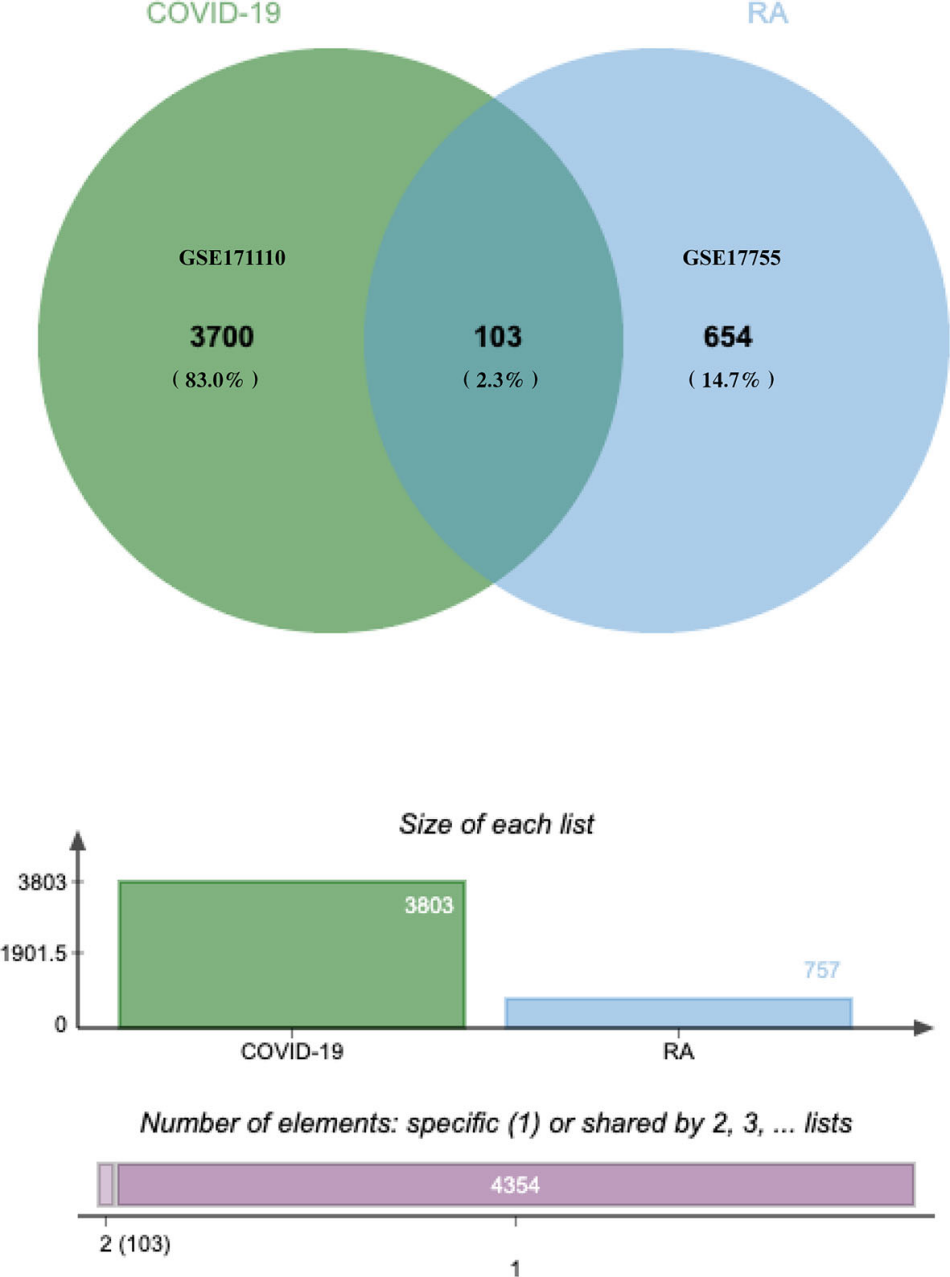
The study included a microarray and an RNA-seq dataset, RA (GSE17755) and COVID-19 (GSE171110). A comprehensive analysis showed that there were 103 common DEGs between COVID-19 and RA.

The flowchart displayed all critical and important procedures of our study (**Figure 1**). We evaluated the human RNA-seq dataset and the microarray dataset from the GEO, and identified the disrupting genes that trigger COVID-19 and RA to investigate the interrelationship and effects between RA and COVID-19. First of all, a total of 3803 DEGs from COVID-19 were screened out, including 1783 up-regulated genes and 2020 down-regulated genes. Similarly, we also found 757 DEGs, including 352 up-regulated genes and 405 down-regulated genes in the RA dataset. LogFC values of DEGs in GSE171110 and GSE17755 were listed in **Table S1** and **Table S2**, respectively. After cross-comparison analysis on Jvenn (24), a reliable Venn analysis portal, we identified 103 common DEG from RA and COVID -19 datasets that were used for further experiments. RA and COVID-19 have many genes in common and are related to each other. The cumulative comparative analysis of the two datasets and the extracted DEGs were shown in **Figure 2**, and all 103 DEGs were listed in **Table S3**.

### Gene Ontology and Pathway Enrichment Analysis

**Figure 3:**
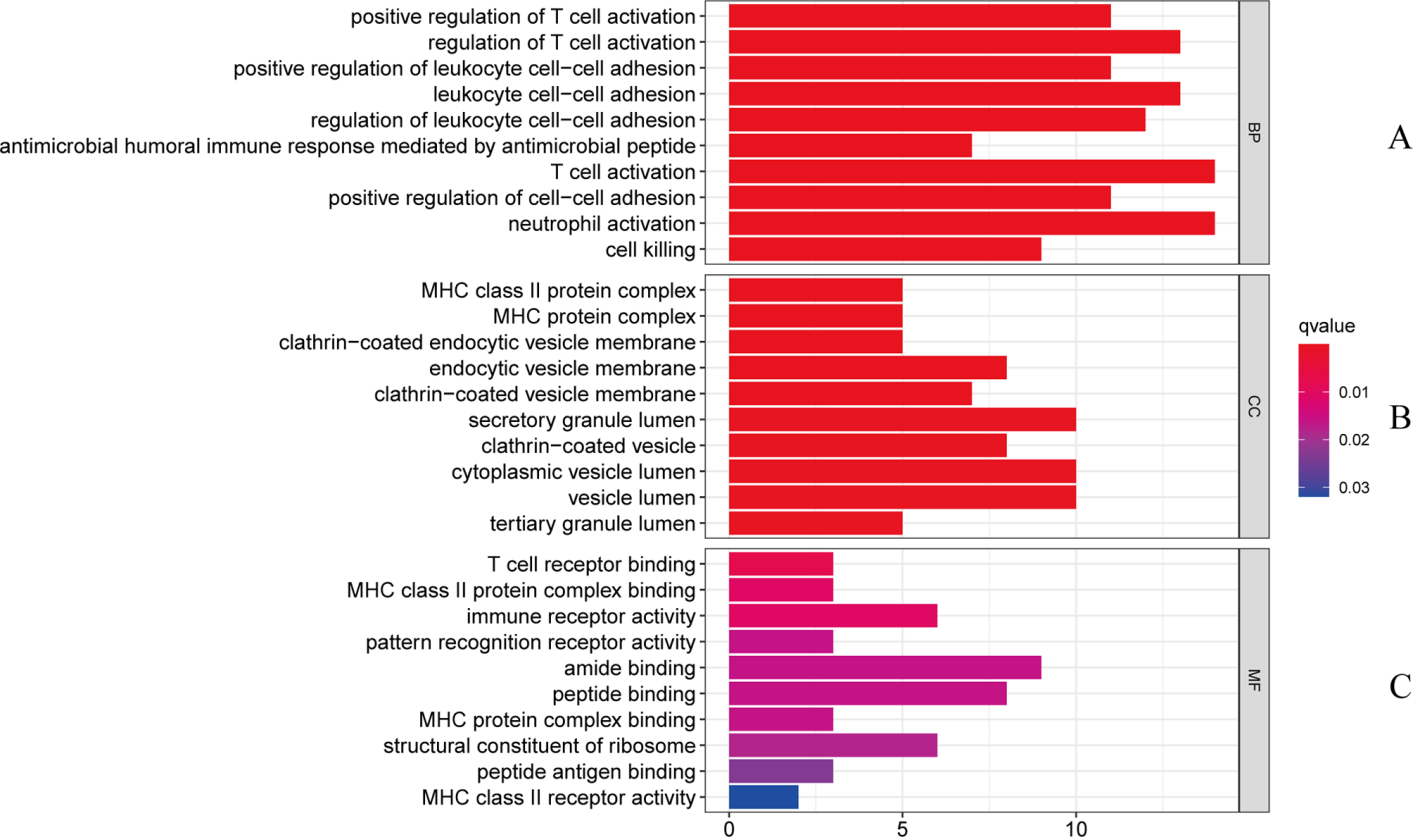
The bar graphs of the ontological analysis of the common DEGs between COVID-19 and RA: **(A)** biological processes; **(B)** cellular components; and **(C)** molecular functions.

The gene ontology enrichment approach is extensively used to reflect the interaction between genes and gene ontology terms, while the KEGG enrichment method can reveal correlations between genes and pathways (36). To discover the biological features and enriched pathways highlighted as common DEGs in this work, “clusterProfiler” package was used to perform gene ontology and pathway enrichment analysis.GO analysis was performed from three aspects: biological process, cell composition, and molecular function. The GO database was selected as an annotation source. **Table 2** summarized the top ten items in the categories of biological process, molecular functions, and cellular components. **Figure 3** also showed the overall ontological analysis linearly in the bar graph for each category. The differentially expressed genes were significantly enriched in positive regulation of T-cell activation in the subset of biological process (BP), enriched in the MHC class II protein complex in the subset of cell compartment (CC), and enriched in T-cell receptor binding in the subset of molecular function (MF), which were all involved into immunotherapy related functional enrichment.

**Table 2:** Ontological analysis of common DEGs among COVID-19 and RA.

Category	GO ID	Term	*P*-values	Genes
GO Biological Process	GO:0050870	positive regulation of T cell activation	1.65E-08	RPS3/HLA-DPB1/HLA-DRA/HLA-DPA1/CD74/KLRK1/CD3E/LCK/IL7R/CCR7/CCL5
	GO:0050863	regulation of T cell activation	1.85E-08	RPS3/HLA-DPB1/HLA-DRA/HLA-DPA1/CD74/KLRK1/CD3E/ARG1/LCK/IL7R/CCR7/CCL5/CLC
	GO:1903039	positive regulation of leukocyte cell-cell adhesion	4.55E-08	RPS3/HLA-DPB1/HLA-DRA/HLA-DPA1/CD74/KLRK1/CD3E/LCK/IL7R/CCR7/CCL5
	GO:0007159	leukocyte cell-cell adhesion	6.94E-08	RPS3/S100A9/HLA-DPB1/HLA-DRA/HLA-DPA1/CD74/KLRK1/CD3E/ARG1/LCK/IL7R/CCR7/CCL5
	GO:1903037	regulation of leukocyte cell-cell adhesion	1.78E-07	RPS3/HLA-DPB1/HLA-DRA/HLA-DPA1/CD74/KLRK1/CD3E/ARG1/LCK/IL7R/CCR7/CCL5
	GO:0061844	antimicrobial humoral immune response mediated by antimicrobial peptide	1.85E-07	S100A9/S100A12/GNLY/CXCL9/PPBP/DEFA4/CAMP
	GO:0042110	T cell activation	2.03E-07	RPS3/CD1C/HLA-DPB1/HLA-DRA/HLA-DPA1/CD74/KLRK1/CD3E/ARG1/LCK/IL7R/CCR7/CCL5/CLC
	GO:0022409	positive regulation of cell-cell adhesion	2.42E-07	RPS3/HLA-DPB1/HLA-DRA/HLA-DPA1/CD74/KLRK1/CD3E/LCK/IL7R/CCR7/CCL5
	GO:0042119	neutrophil activation	3.87E-07	EEF1A1/TXNDC5/S100A9/QPCT/MMP9/S100A12/ANXA3/ARG1/TNFAIP6/TLR2/CCL5/PPBP/DEFA4/CAMP
	GO:0001906	cell killing	4.63E-07	CD1C/HLA-DRA/S100A12/KLRK1/ARG1/IL7R/GNLY/DEFA4/CAMP
GO Cellular Component	GO:0042613	MHC class II protein complex	1.40E-08	HLA-DPB1/HLA-DRA/HLA-DPA1/HLA-DMA/CD74
	GO:0042611	MHC protein complex	1.64E-07	HLA-DPB1/HLA-DRA/HLA-DPA1/HLA-DMA/CD74
	GO:0030669	clathrin-coated endocytic vesicle membrane	1.68E-06	HLA-DPB1/HLA-DRA/HLA-DPA1/FCGR1A/CD74
	GO:0030666	endocytic vesicle membrane	2.08E-06	HLA-DPB1/HLA-DRA/HLA-DPA1/FCGR1A/CD74/ANXA3/TLR2/MARCO
	GO:0030665	clathrin-coated vesicle membrane	2.51E-06	HLA-DPB1/HLA-DRA/HLA-DPA1/FCGR1A/CD74/HIP1/IL7R
	GO:0034774	secretory granule lumen	6.61E-06	EEF1A1/TXNDC5/S100A9/QPCT/S100A12/ARG1/CTSW/PPBP/DEFA4/CAMP
	GO:0030136	clathrin-coated vesicle	7.01E-06	HLA-DPB1/HLA-DRA/HLA-DPA1/FCGR1A/CD74/HIP1/IL7R/SNX9
	GO:0060205	cytoplasmic vesicle lumen	7.37E-06	EEF1A1/TXNDC5/S100A9/QPCT/S100A12/ARG1/CTSW/PPBP/DEFA4/CAMP
	GO:0031983	vesicle lumen	7.78E-06	EEF1A1/TXNDC5/S100A9/QPCT/S100A12/ARG1/CTSW/PPBP/DEFA4/CAMP
	GO:1904724	tertiary granule lumen	9.51E-06	QPCT/MMP9/TNFAIP6/PPBP/CAMP
GO Molecular Function	GO:0042608	T cell receptor binding	2.67E-05	HLA-DRA/CD3E/LCK
	GO:0023026	MHC class II protein complex binding	1.07E-04	HLA-DRA/HLA-DMA/CD74
	GO:0140375	immune receptor activity	1.12E-04	HLA-DRA/HLA-DPA1/CD74/IL7R/CCR7/IL1R2
	GO:0038187	pattern recognition receptor activity	2.73E-04	TLR2/CLEC4E/MARCO
	GO:0033218	amide binding	3.32E-04	CD1C/HLA-DPB1/HLA-DRA/HLA-DPA1/CD74/NQO2/TLR2/BDKRB1/MARCO
	GO:0042277	peptide binding	3.76E-04	CD1C/HLA-DPB1/HLA-DRA/HLA-DPA1/CD74/TLR2/BDKRB1/MARCO
	GO:0023023	MHC protein complex binding	3.96E-04	HLA-DRA/HLA-DMA/CD74
	GO:0003735	structural constituent of ribosome	5.08E-04	RPS3/RPL13A/RPL13/RPSA/RPL3/RPL18
	GO:0042605	peptide antigen binding	7.37E-04	HLA-DPB1/HLA-DRA/HLA-DPA1
	GO:0032395	MHC class II receptor activity	1.34E-03	HLA-DRA/HLA-DPA1

Top 10 terms of each category are listed.

**Figure 4:**
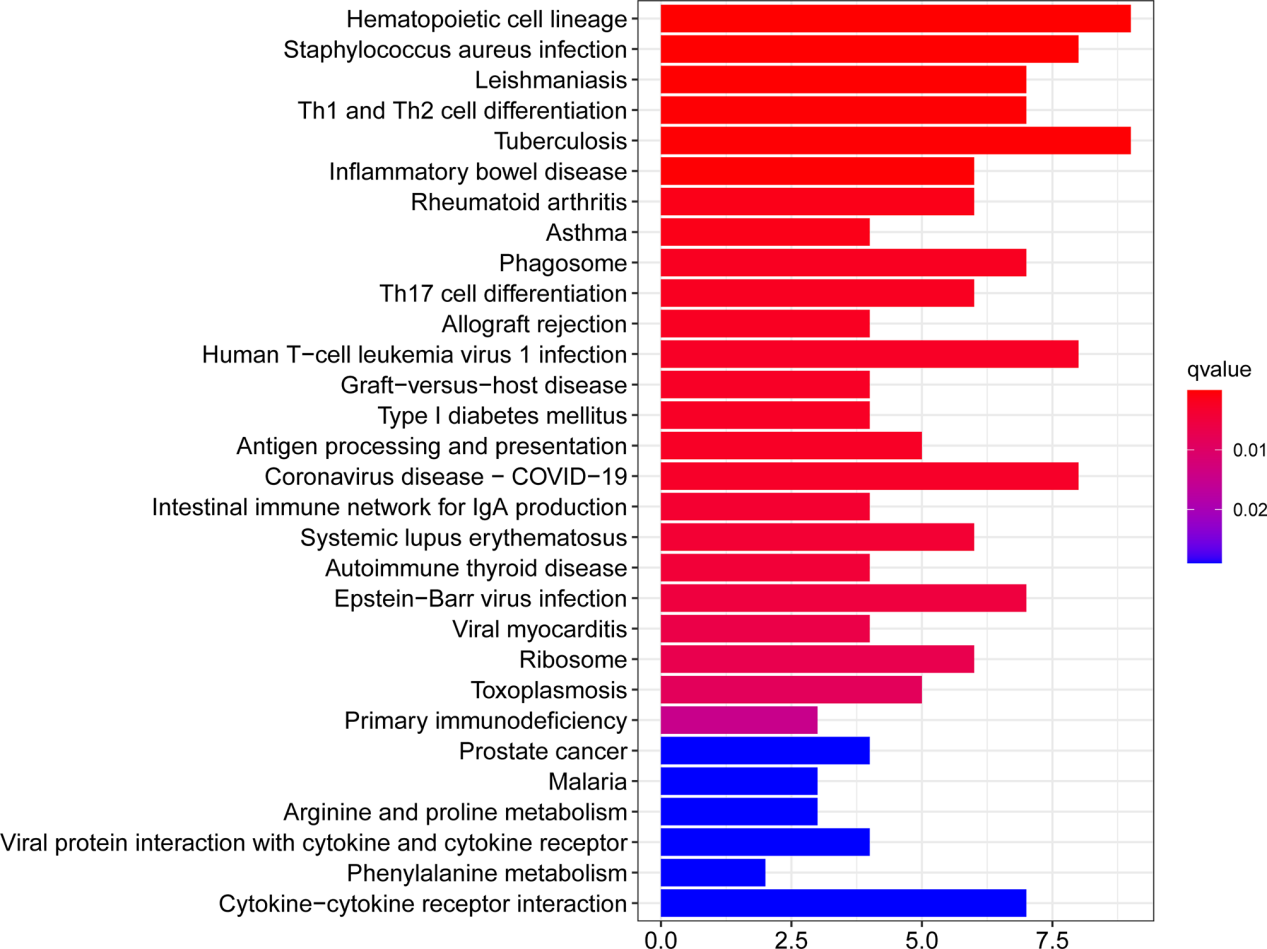
The bar graphs of pathway enrichment analysis of the common DEGs between COVID-19 and RA performed by KEGG 2021 human pathway.

The KEGG Pathway analysis was a modeling technique that revealed the mutual effect between different diseases through fundamental molecular or biological processes. The most affected common DEG approach between RA and COVID-19 was collected by the global database KEGG. KEGG pathway analysis revealed the following top 10 pathways: hematopoietic cell lineage, staphylococcus aureus infection, leishmaniasis, Th1 and Th2 cell differentiation, tuberculosis, inflammatory bowel disease, rheumatoid arthritis, asthma, phagosome, Th17 cell differentiation. **Table 3** listed the KEGG enrichment pathways generated from the selected dataset. For a more detailed illustration, **Figure 4** also showed the pathway enrichment analysis in a bar plot. In addition, GO and KEGG pathway analysis along with Gene counts and gene symbols were shown in **Table S4** and **Table S5**, respectively.

**Table 3:** Pathway enrichment analysis of common DEGs among COVID-19 and RA.

Category	Pathways	*P*-values	Genes
KEGG 2021 Human	Hematopoietic cell lineage	5.71E-08	CD1C/HLA-DPB1/HLA-DRA/HLA-DPA1/FCGR1A/HLA-DMA/CD3E/IL7R/IL1R2
	Staphylococcus aureus infection	6.50E-07	HLA-DPB1/HLA-DRA/HLA-DPA1/FCGR1A/HLA-DMA/C2/DEFA4/CAMP
	Leishmaniasis	1.90E-06	EEF1A1/HLA-DPB1/HLA-DRA/HLA-DPA1/FCGR1A/HLA-DMA/TLR2
	Th1 and Th2 cell differentiation	6.33E-06	HLA-DPB1/HLA-DRA/HLA-DPA1/HLA-DMA/CD3E/LCK/STAT4
	Tuberculosis	9.01E-06	HLA-DPB1/HLA-DRA/HLA-DPA1/FCGR1A/HLA-DMA/CD74/TLR2/CLEC4E/CAMP
	Inflammatory bowel disease	9.95E-06	HLA-DPB1/HLA-DRA/HLA-DPA1/HLA-DMA/STAT4/TLR2
	Rheumatoid arthritis	7.75E-05	HLA-DPB1/HLA-DRA/HLA-DPA1/HLA-DMA/TLR2/CCL5
	Asthma	8.88E-05	HLA-DPB1/HLA-DRA/HLA-DPA1/HLA-DMA
	Phagosome	1.62E-04	HLA-DPB1/HLA-DRA/HLA-DPA1/FCGR1A/HLA-DMA/TLR2/MARCO
	Th17 cell differentiation	1.78E-04	HLA-DPB1/HLA-DRA/HLA-DPA1/HLA-DMA/CD3E/LCK
	Allograft rejection	2.00E-04	HLA-DPB1/HLA-DRA/HLA-DPA1/HLA-DMA
	Human T-cell leukemia virus 1 infection	2.94E-04	CCNE2/HLA-DPB1/HLA-DRA/HLA-DPA1/HLA-DMA/CD3E/LCK/IL1R2
	Graft-versus-host disease	2.96E-04	HLA-DPB1/HLA-DRA/HLA-DPA1/HLA-DMA
	Type I diabetes mellitus	3.25E-04	HLA-DPB1/HLA-DRA/HLA-DPA1/HLA-DMA
	Antigen processing and presentation	3.28E-04	HLA-DPB1/HLA-DRA/HLA-DPA1/HLA-DMA/CD74
	Coronavirus disease - COVID-19	3.95E-04	RPS3/RPL13A/RPL13/RPSA/RPL3/RPL18/TLR2/C2
	Intestinal immune network for IgA production	5.39E-04	HLA-DPB1/HLA-DRA/HLA-DPA1/HLA-DMA
	Systemic lupus erythematosus	6.17E-04	HLA-DPB1/HLA-DRA/HLA-DPA1/FCGR1A/HLA-DMA/C2
	Autoimmune thyroid disease	7.27E-04	HLA-DPB1/HLA-DRA/HLA-DPA1/HLA-DMA
	Epstein-Barr virus infection	9.08E-04	CCNE2/HLA-DPB1/HLA-DRA/HLA-DPA1/HLA-DMA/CD3E/TLR2
	Viral myocarditis	1.16E-03	HLA-DPB1/HLA-DRA/HLA-DPA1/HLA-DMA
	Ribosome	1.35E-03	RPS3/RPL13A/RPL13/RPSA/RPL3/RPL18
	Toxoplasmosis	1.71E-03	HLA-DPB1/HLA-DRA/HLA-DPA1/HLA-DMA/TLR2
	Primary immunodeficiency	3.10E-03	CD3E/LCK/IL7R
	Prostate cancer	6.68E-03	CCNE2/MMP9/FGFR2/IL1R2
	Malaria	6.75E-03	KLRB1/KLRK1/TLR2
	Arginine and proline metabolism	7.13E-03	MAOB/ARG1/MAOA
	Viral protein interaction with cytokine and cytokine receptor	7.43E-03	CCR7/CCL5/CXCL9/PPBP
	Phenylalanine metabolism	7.49E-03	MAOB/MAOA
	Cytokine-cytokine receptor interaction	7.58E-03	IL32/IL7R/CCR7/CCL5/CXCL9/IL1R2/PPBP

### Classification of Hub Proteins and Submodule

**Figure 5:**
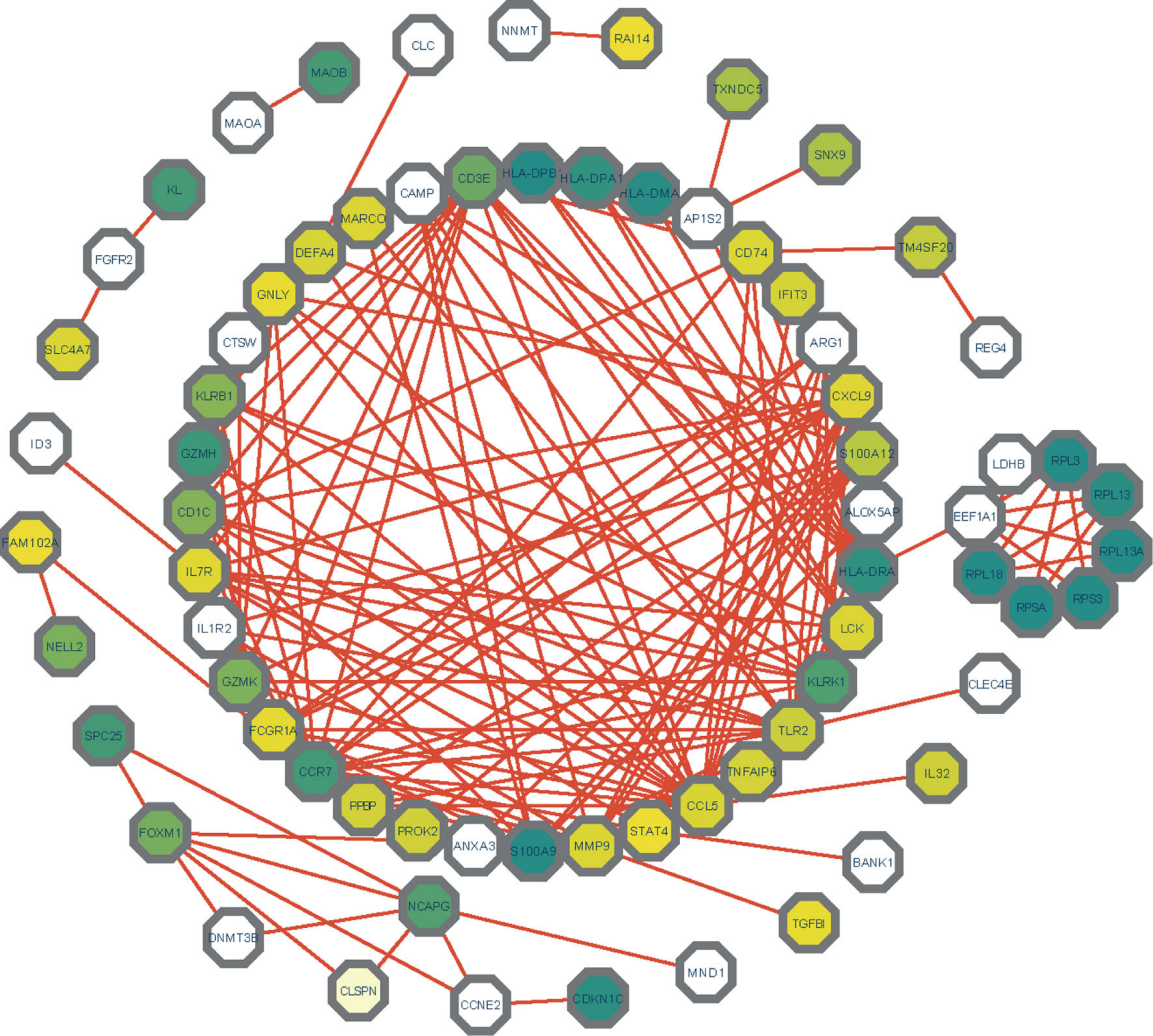
The PPI network of common DEGs among COVID-19 and RA. In the figure, the octagonal nodes represent DEGs and edges represent the interactions between nodes. The PPI network was generated using String and visualized in Cytoscape.

**Figure 6:**
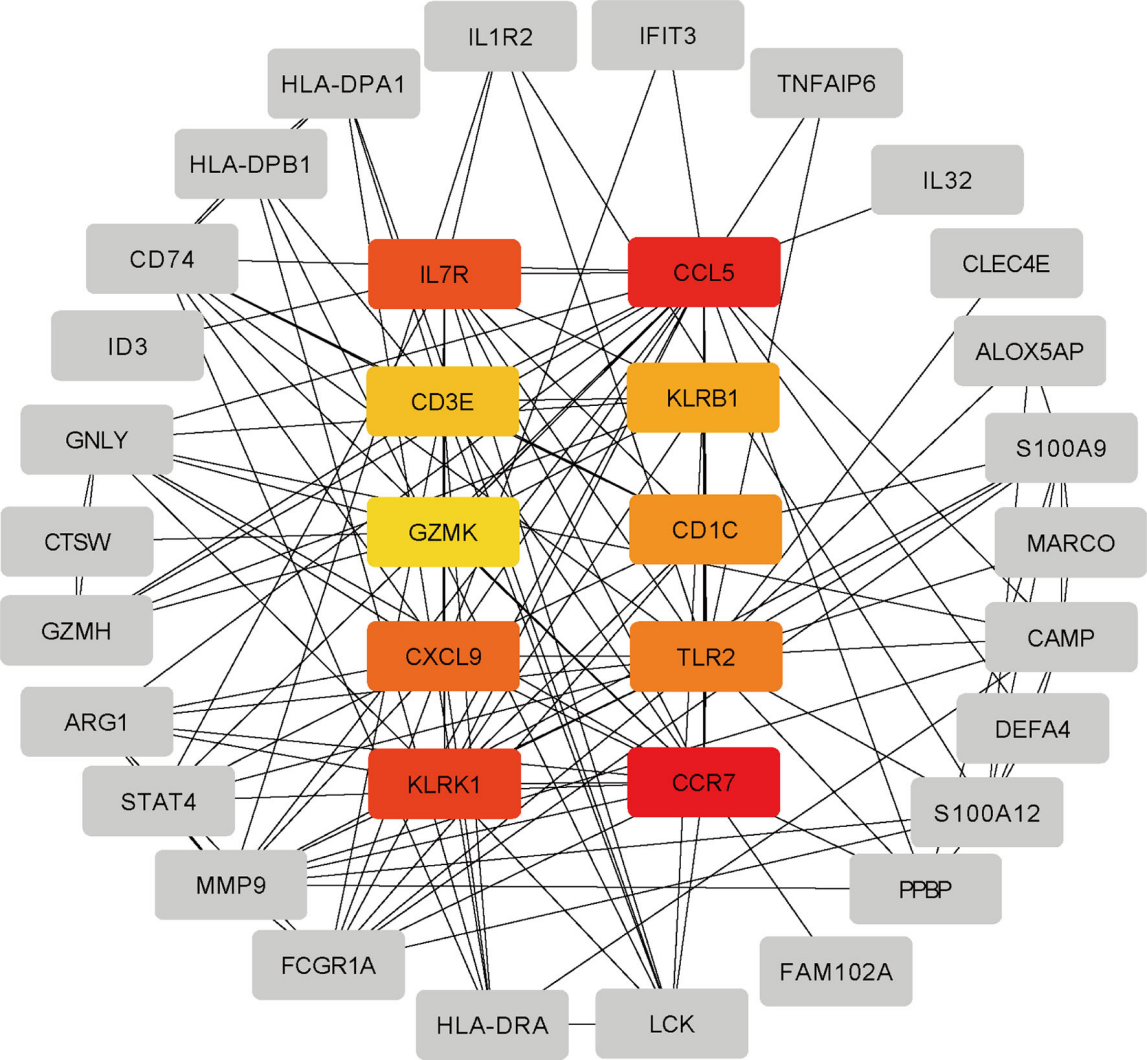
The hub gene was identified from the PPI network using the Cytohubba plug in Cytosacpe. Here, the colored nodes represent the highlighted top 10 hub genes and their interactions with other molecules.

The differentially expressed genes between COVID-19 and RA were uploaded to STRING to explore protein-protein interactions and identify common DEGs interactions and association pathways. The PPI network of shared DEGs between COVID-19 and RA is depicted in **Figure 5**. And the topology table of PPI with node degree, betweenness centrality, stress centrality and closeness centrality in **Table S6**. In PPI networks, most of the interconnected nodes were considered as hub genes. We identified the top 10 (9.71%) DEGs as the most influential genes based on PPI network analysis using the Cytohubba plugin in Cytoscape. CCR7, CCL5, KLRK1, IL7R, CXCL9, TLR2, CD1C, KLRB1, CD3E, and GZMK are the hub genes. We performed a ROC analysis for RA and COVID-19 in **Figure S1**. In the RA cohort, the AUC values of CCR7, CCL5, KLRK1, IL7R, TLR2, CD1C, CD3E, and GZMK were greater than 0.8. Ten hub genes were included in the logistic regression analysis and the model was built, the AUC value of the model was 1. In the cohort COVID-19, the AUC values of all hub genes were greater than 0.8. Similarly, 10 genes were included in the logistic regression analysis, and the AUC value of the model was also 1. The hub genes may be feasible biomarkers that may also generate new therapeutic strategies for diseases under investigation. After that, we created a submodule network using the Cytohubba plugin to better capture their close connection and proximity. The extended network of hub gene interactions derived from the PPI network was shown in **Figure 6**.

### Construction of Regulatory Networks

**Figure 7:**
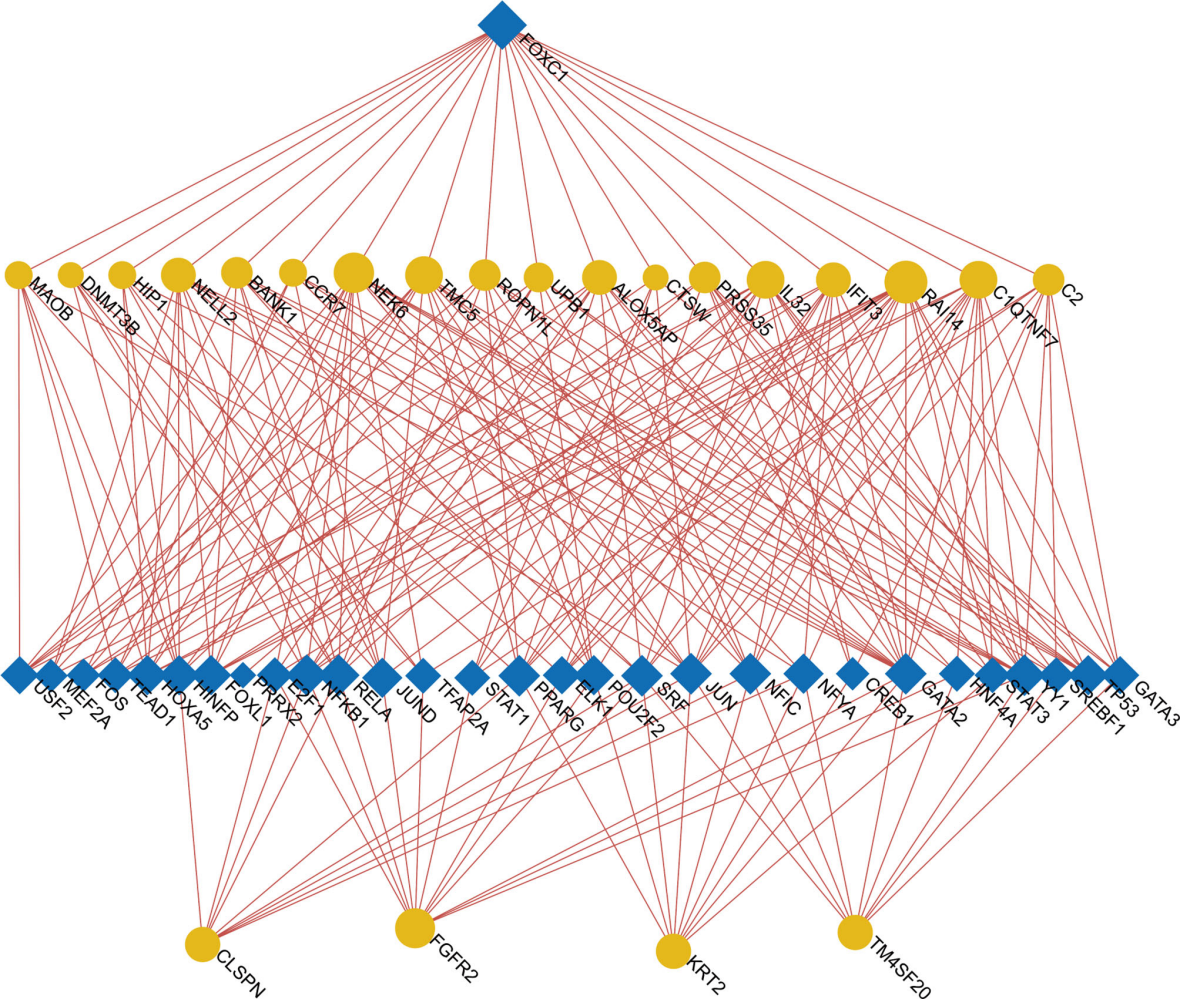
The Network Analyst created an interconnected regulatory interaction network of DEG-TFs. In it, blue square nodes represent TFs, gene symbols interact with TFs as yellow circle nodes.

**Figure 8:**
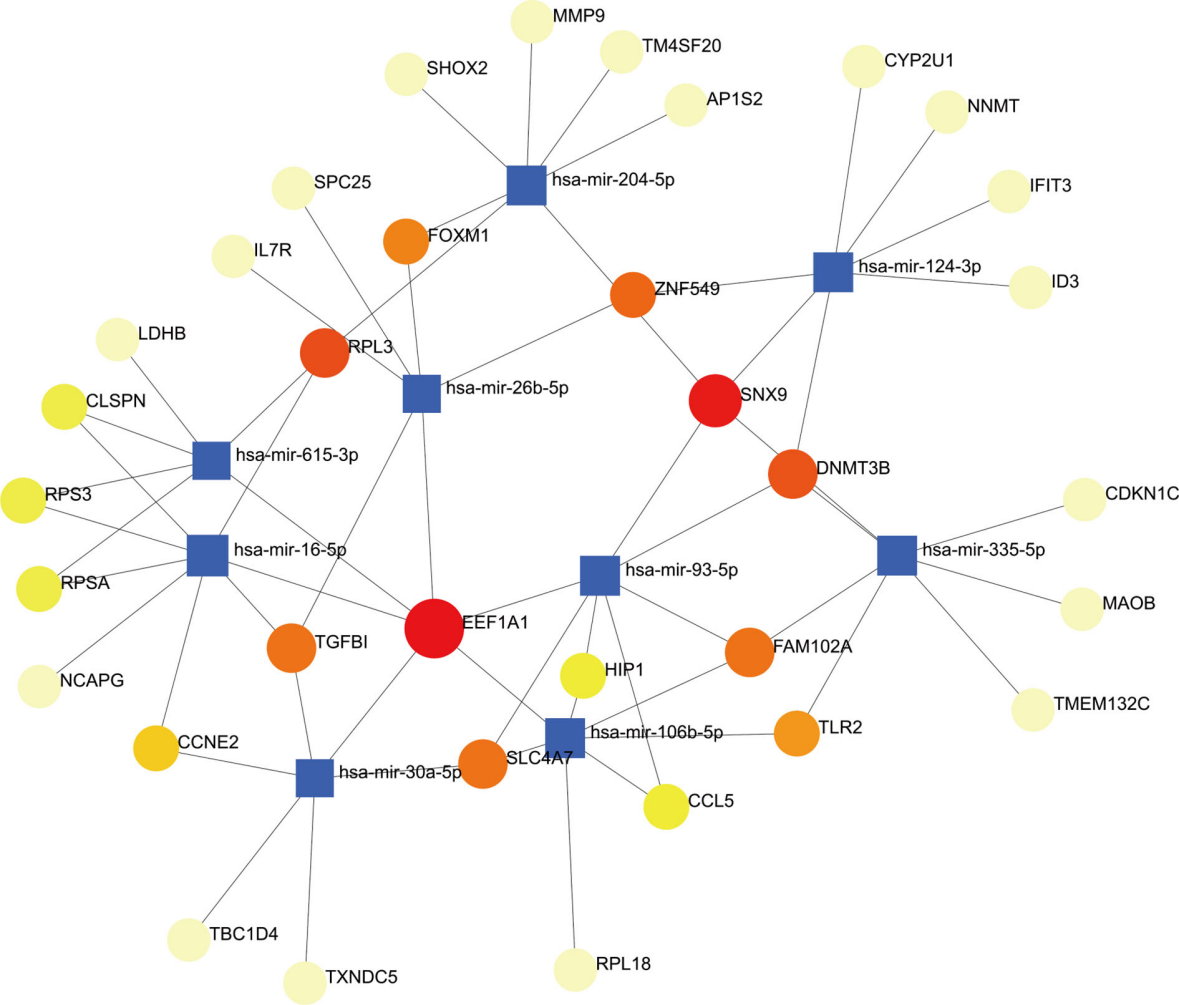
The interconnected regulatory interaction network of DEGs-miRNAs. The circle node indicates miRNAs and the gene symbols interact with miRNAs in the shape of a square.

To determine the major variationat the transcriptional level and gain a deeper understanding of the key protein regulatory molecules or common DEG, we used a network-based method to decipher the regulatory TFs and miRNAs. By analyzing the interaction network between TFs genes and miRNAs genes, we found that 52 transcription factors (TFs) and 43 post-transcriptional (miRNAs) regulatory signals regulate several common DEGs, essentially indicating that there was strong interference between them. The interaction between regulatory TFs and common DEGs was shown in **Figure 7**. The interaction between miRNA regulatory factors and common DEGs was depicted in **Figure 8**. The construction and analysis of the regulatory network of target TF-genes and target miRNA-genes, including topology table, were shown in **Table S7** and **Table S8**, respectively.

### Identification of Candidate Drugs

Under the aspect of common DEGs as potential drug objects in RA and COVID-19, 10 possible drug molecules were identified by Enrichr based on the transcriptional characteristics from the DSigDB database, and the top 10 compounds were extracted based on *P*-value. These potential drugs were recommended for use in the common DEGs, which was a common compound for the treatment of two diseases. **Table 4** showed the effective drugs of common DEGs in the DSigDB database.

**Table 4:** The recommended drugs for COVID-19.

Name	*P*-values	Chemical Formula	Structure
progesterone CTD 00006624	9.75602E-10	C_21_H_30_O_2_	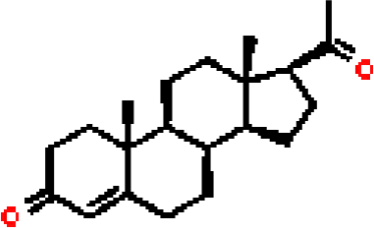
estradiol CTD 00005920	4.90477E-09	C_18_H_24_O_2_	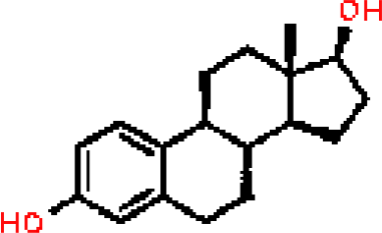
Tetradioxin CTD 00006848	1.99339E-07	C_12_H_4_Cl_4_O_2_	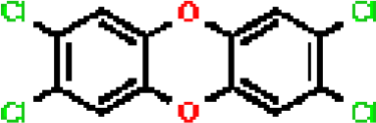
NICKEL SULFATE CTD 00001417	2.55916E-07	NiO_4_S	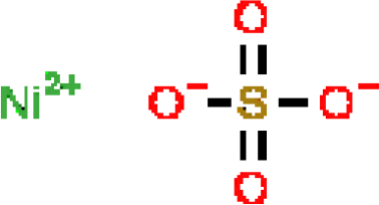
Sodium dodecyl sulfate CTD 00006753	1.91096E-06	C_12_H_25_NaO_4_S	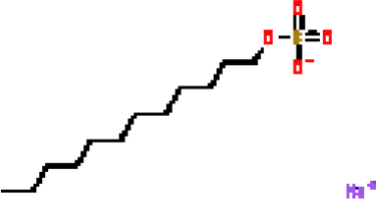
genistein CTD 00007324	3.99898E-06	C_15_H_10_O_5_	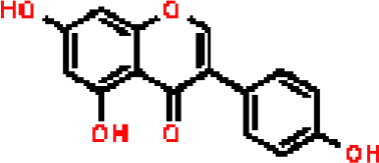
calcitriol CTD 00005558	4.67458E-06	C_27_H_44_O_3_	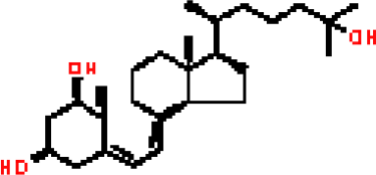
Retinoic acid CTD 00006918	5.78219E-06	C_20_H_28_O_2_	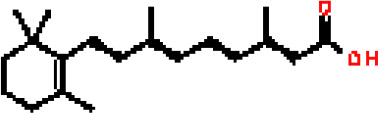
Tamibarotene CTD 00002527	5.93463E-06	C_22_H_25_NO_3_	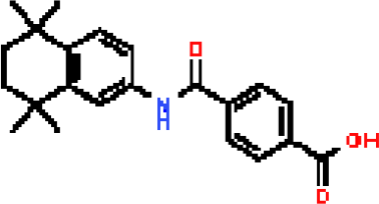
dexamethasone CTD 00005779	1.10828E-05	C_22_H_29_FO_5_	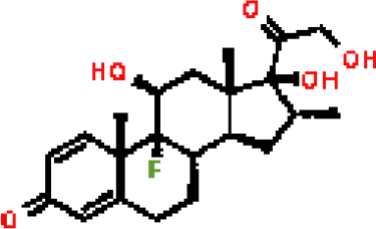

### Identification of Disease Association

**Figure 9:**
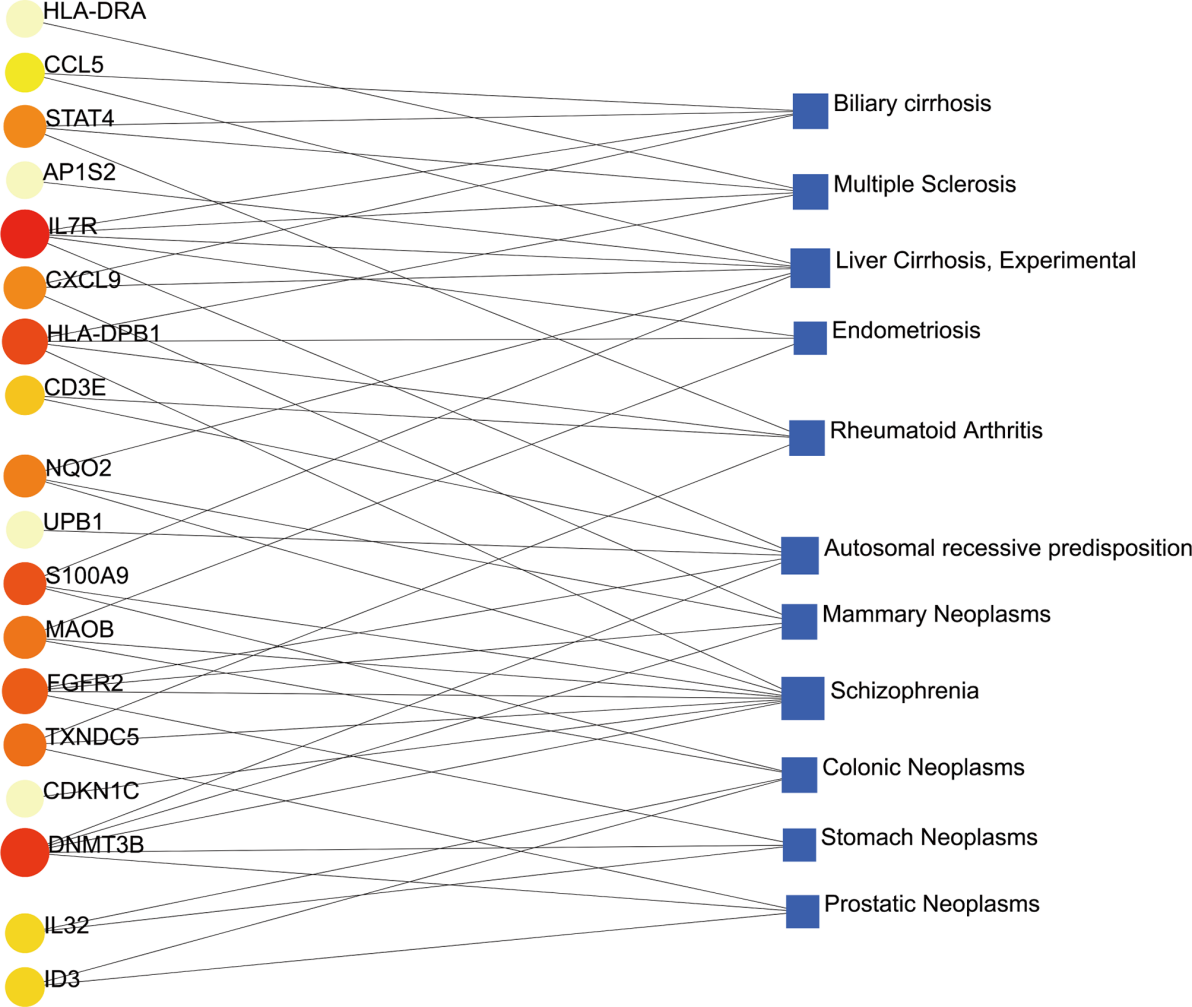
The gene-disease association network represents diseases associated with common DEGs. The disease represented by the square node and also its subsequent gene symbols are defined by the circular node.

Different diseases may be linked and usually must have one or more similar genes (37). Techniques for developing disease therapies begin with deciphering the relationship between genes and disease (38). DisGeNET was a knowledge management platform that integrates and standardizes data from multiple sources on disease-associated genes and variants. In our gene-disease association analysis, we found that the diseases biliary cirrhosis, multiple sclerosis, liver cirrhosis, experimental cirrhosis, endometriosis, rheumatoid arthritis, autosomal recessive predisposition, and mammary neoplasms coordinated most strongly with the hub genes we reported, and even in COVID-19. The gene-disease association was shown in **Figure 9**.

## Discussion

Inspired by Mahmud’s (37) research investigating the effects of SARS-COV-2 infection on patients with idiopathic pulmonary fibrosis and chronic obstructive pulmonary disease, we explored the potential interaction between RA and COVID -19 as a unique perspective. The aim of this study was to find out the possible interaction between RA and COVID-19. RA was a systemic inflammatory and autoimmune disease whose inflammation and immune response might promote the excessive secretion of inflammatory cytokines, growth factors, and matrix metalloproteinases (MMPs) (39, 40). COVID-19 increased proinflammatory cytokines, granulocyte colony-stimulating factors(G-CSF), and chemokines were detected, which was equivalent to excessive activation of innate immunity (41). Therefore, COVID-19 infection was a high risk factor for exacerbation of RA disease (42). In this work, we developed a network-based method to study the gene expression profiling of two RNA-seq datasets from RA and COVID-19 and to identify molecular targets that may be useful as potential biomarkers for COVID-19. Expression profiling analysis from high-throughput sequencing datasets has been used in biomedical systems research and has become an important source of biomarkers for identifying various diseases (43). We analyzed the gene set enrichment by discovering the biological process of cell signaling pathway and gene ontology (GO) to obtain the association between COVID-19 and RA from the perspective of common DEGs between diseases. Here, transcriptomics analysis of RA and COVID-19 revealed that 103 common DEGs assessed by GO pathway analysis based on *P*-value had similar expression patterns in the two diseases. With the new scientific discoveries, the structure of GO constantly evolved, acquired biological knowledge of gene functions, and constantly improved to represent the latest state of biological knowledge (44). The “clusterProfiler” package was used to perform three types of GO analysis: biological process (molecular activity), cell composition (gene regulatory function), and molecular function (activity of molecular level) in the ontological process with the GO database as a source information. For the biological process, positive regulation of T cell activation (11 genes) and regulation of T cell activation (13 genes) were among the top GO terms. T cell activation was caused by the intracellular signaling cascade triggered by the antigen-activated T cell receptor (TCR) (45). RA was an autoimmune disease characterized by chronic inflammation and T cell hyper-activation (46). Immunogenetics of RA suggested that RA was closely associated with the regulation of T cell activation, which was ultimately determined by positive signals from costimulatory molecules and negative signals from regulatory T cells (47, 48). The phenotype of the SARS-CoV-2-specific CD8+ T cells, revealed a strong T cell activation in COVID-19 patients, T cells exhibited a more robust activation profile in severe disease than in mild disease (49, 50). In the cellular component experiment, MHC class II protein complex (5 genes) and MHC protein complex (5 genes) were two major GO pathways. Early data had shown that major histocompatibility complex (MHC) was associated with the risk of RA (51). This risk effect was related to differences in protein structure and expression (52). SARS-CoV-2 infection was found to cause downregulation of major histocompatibility complex class I (MHC-I) *in vivo* and *in vitro* (53). According to the molecular function, top GO terms were T cell receptor binding (3 genes) and MHC class II protein complex binding (3 genes). In convalescent COVID-19 patients, the study observed a public and diverse T cell response to SARS-CoV-2 epitopes that revealed T cell receptor (TCR) motifs with germline-encoded features (54).

KEGG pathway analysis was the main method used to assess an organism’s higher level systematic responses to internal changes. 103 common DEGs were identified to find a similar pathway for RA and COVID-19. The top 10 KEGG pathways were: hematopoietic cell lineage, staphylococcus aureus infection, leishmaniasis, Th1 and Th2 cell differentiation, tuberculosis, inflammatory bowel disease, rheumatoid arthritis, asthma, phagosome, Th17 cell differentiation. Evidence suggests that the SARS-CoV-2 virus entry receptor (angiotensin-converting enzyme 2, ACE2) and receptor for angiotensin II (AT1) were expressed and functional on the surface of hematopoietic stem/progenitor cells (HSPCs), and the proinflammatory system also played an important role (55).

DEGs genes were used to create a PPI network to understand the mutual biological functional properties of proteins and predict drug targets. Identification of hub genes that may be essential drug targets or biomarkers for COVID-19 were associated with various pathogeneses. The top 10 hub genes according to the MCC method include: CCR7, CCL5, KLRK1, IL7R, CXCL9, TLR2, CD1C, KLRB1, CD3E, and GZMK identified at RA and COVID-19. CCR7 was a homeostatically expressed chemokine receptor that was known to regulate the homing of various types of immune cells to lymphoid organs (56, 57). Study demonstrated that CCR7 signaling was essential for the induction of collagen-induced arthritis (CIA) and identify CCR7 as a potential therapeutic target in RA (58). Recently, expression of the receptor CCR7 on circulating monocytes was found to be representative of the disease activity score (DAS28) of RA (59). Multisystem inflammatory syndrome in children (MIS-C) presents as an immunopathogenic disease involving multi-organ dysfunction and systemic inflammation. Recent reports indicated a new clinical syndrome in children who had multisystem inflammatory syndrome associated with SARS-CoV-2, in the acute phase of MIS-C, high HLA-DR expression on γδ and CD4+CCR7+ T cells, and these immune cell populations were activated (60–62). Another hub gene CCL5 was an important hub gene involved in RA and COVID-19 and played an important role in inflammation and immune responses. RANTES/CCL5, regulated upon activation, normally expressed and secreted, was involved in the pathogenesis of RA by facilitating leukocyte infiltration (63). Research found that patients with COVID-19 showed higher serum concentrations of RANTES/CCL5 and observed “chemokine signature” (64). Beyond this, a study demonstrated that low level of CCL5 expression was associated with the severity of COVID-19 (65). Furthermore, KLRK1 (NKG2D) was a C-type lectin receptor present on natural killer (NK) cells, γδ, CD8+ and CD4+ T cells, and its gene polymorphisms might modify the risk of development and severity of RA (66). The NKG2D receptor was also a new potential target to regulate the direct interaction between SARS-CoV-2 and NK cells and could be used as a therapeutic target for COVID-19 (11). Single-cell transcriptome analyzes of human inflammatory monocytes from COVID-19 and RA patients showed that a group of CD127 (IL-7 receptor subunit) positive cells induced initially inactive monocytes to respond to IL-7 and negatively affected the inflammatory phenotype of monocytes (67).

Moreover, research data showed that inflammatory macrophages were shared and strikingly abundant in severe COVID-19 bronchoalveolar lavage samples and inflamed RA synovium, with an obvious arrangement of pro-inflammatory genes and interferon response genes such as CXCL10, CXCL9 and so on (68). Severe pathology in SARS-CoV-2 infection was triggered by excessive cytokine release or a cytokine storm. Another hub gene TLR2 was necessary for β-coronavirus-induced inflammatory response (69). Its dependent signaling pathway induced the production of pro-inflammatory cytokines during coronavirus infection and recognizes the SARS-CoV-2 envelope protein as its ligand (69). Regarding the CD1c+ gene, studies had shown that the number of CD1c+ myeloid dendritic cells and plasma dendritic cells decreased in patients infected with SARS-CoV-2 even 7 months later, which contributed to a better understanding of the immunological sequelae of COVID-19 (70). In COVID-19, dendritic cell numbers and IFN-α production were defective, which was related to the severity of the disease (70). KLRB1 hub gene was associated with senescent T cells, which were highly inflammatory and cytotoxic mediators and express natural killer cell receptor (NKR, previous HGNC symbols for KLRB1 gene) to circumvent its antigen specificity. Senescent T cells could damage healthy organs in intense inflammation, and they also applied to COVID-19 and RA (71). Currently, there was not much research on KLRB1 gene, which also offered a new idea for targeted treatment of diseases in COVID-19. A meta-analysis (72)compiled a comprehensive ranking of host genes in human beta coronavirus infection, with high-ranking genes, including CD3E, suggested as prognostic factors for COVID-19. Finally, studies had shown that GZMB, as part of the effector CD8+T cell population with memory precursor-like features, was involved in the immune development of SARS-CoV-2 (73). Finally, we performed a ROC analysis of the top ten hub genes. In our analysis, we could find that the AUC value of most hub genes (80%) is above 0.8 in the cohort RA. This meaned that these genes were more effective in predicting RA. When the 10 genes were included in the logistic regression analysis and the model was constructed, it was found that the AUC value of the model could reach 1. Also, in the cohort COVID-19, the AUC values of the top 10 genes were all above 0.8, which means that the hub gene in this study has a higher predictive power for COVID-19. The ten hub genes were included in the logistic regression analysis and the model was constructed, and the AUC value could also reach 1. If the biological view of COVID-19 was confirmed, these hub genes could be considered as powerful biomarkers and new drug targets.

We also discovered relationships between the diseases in terms of TFs-genes and miRNAs. TFs mainly regulated transcription and expression of target genes. MiRNAs modified post-transcriptional genes and influenced the development of organisms. For example, FOXC1, STAT3, GATA2, JUN, POU2F2, PPARG, JUND, NFKB1, FOXL1, HOXA5, and FOS are among the TFs associated with various types of RA. In the context of visualization of DEG-miRNAs, mir-204-5p, mir-124-3p, mir-26b-5p, mir-16-5p, mir-106b-5p are associated with the pathogenesis of RA. Some studies had shown that mir-204-5p was involved in proliferation and production of inflammatory cytokines in RA fibroblast-like synoviocytes (74). In addition, mir-124-3p was involved in the pathogenicity of Th17 cells in RA (75). Mir-26b-5p might be a novel biomarker for stratification of RA patients and was worth further clinical validation (76). Expression of mir-16-5p was inhibited, vascular endothelial growth factor (VEGF) production in osteoblasts is increased, and angiogenesis of endothelial progenitor cells is inhibited in RA (77). The results of Tao et al. (78) suggested that inhibition of miR-106b-5p could alleviate CIA-associated inflammation and bone destruction, and thus could serve as a potential therapeutic for human RA treatment. It was worth noting that we also predicted three miRNAs (mir-615-3p, mir-93-5p, mir-335-5p) associated with different genes from COVID-19. Prasad et al. (79) identified that mir-615-3p as potential candidates for the treatment of COVID-19 and associated manifestations. The obtained results showed that induction of interleukin-8 by SARS-CoV-2 Spike protein in bronchial epithelial IB3-1 cells was mediated by a miR-93-5p agomiR (80). Bioinformatics analysis revealed that miR-335-5p and miR-26b-5p were modulated by Spike and ACE together with histone deacetylate (HDAC) pathway and that SARS-CoV-2 off-label HDAC inhibitors (HDACis) drugs might be repurposed to limit or block host-virus interactions (81).

In addition, we also conducted gene-disease (GD) analysis to forecast the association between remarkable DEGs with degree lager than 3 and different diseases. The results showed various types of diseases related to COVID-19. For instance, several genes related to liver diseases, such as biliary cirrhosis and experimental cirrhosis, were found in the study. SARS-CoV-2 had a remarkable tropism for the liver and the biliary tract (82), including the infrequent and chronic manifestation of cholangiopathy (83). Second, endometriosis, a disease associated with high levels of chronic stress, had been linked to psychological problems such as post-traumatic stress disorder, psychological distress, depression, and anxiety caused by COVID-19 pandemic (84). Third, the loss-of-function study in autosomal recessive or autosomal dominant defects suggested that congenital immune errors could lead to life-threatening COVID-19 pneumonia in patients who had not been seriously infected in the past (85). As for schizophrenia, the virus hypothesis has attracted more and more attention. In the current pandemic COVID-19 there was evidence of new mental illness after exposure to the virus, implying that neuroinflammation in key areas of the brain may cause psychiatric symptoms (86). Furthermore, COVID-19 was closely related to most neoplasms, such as mammary neoplasms, colonic neoplasms, stomach neoplasms, prostatic neoplasms. Cancer patients had a higher risk of severe COVID-19 disease and associated death due to the severity of the disease and compromised immune system (87).

In the past, several chemicals and drugs had been used as potential therapeutic against COVID-19. Remdesivir, for example, was the first approved treatment for severe COVID-19, a novel nucleoside analog with broad-spectrum antiviral activity in RNA viruses (88). Moreover, favipiravir (FPV), was a novel antiviral drug that acted as a competitive inhibitor of RNA-dependent RNA polymerase and prevented viral transcription and replication, had also been used in the therapy of SARS-CoV-2 infection (89). A phase 2a clinical trial in COVID-19 patients showed that molnupiravir accelerated the elimination of SARS-CoV-2 RNA and infectious virus (90). In addition, a clinical study showed that treatment with hydroxychloroquine in COVID-19 patients had a significant effect, which was enhanced by azithromycin (91). However, a meta-analysis showed that the combination of hydroxychloroquine and azithromycin significantly increased mortality (92). In the context of COVID-19 pandemic, it was an inevitable trend to identify new drugs and/or compounds for the treatment of SARS-CoV-2 infection. Therefore, we identified progesterone and estradiol to be used in diseases related to anti- inflammation, remodeling immune cell competence, stimulating antibody production, and promoting proliferation and repair of respiratory epithelial cells (93). Progesterone and estradiol were endogenous reproductive steroids abundantly produced in the periphery by the adrenal glands and ovaries and *de novo* by the brain. They played an important physiological role by modulating inflammatory processes and behavior (94). Studies found that male COVID-19 patients were twice as likely as female patients to be admitted to the ICU and had a higher mortality rate, suggesting that there is a strong gender disparity in SARS-CoV-2 (95). This evidence suggested that female sex hormones may be an explanation for reducing COVID-19 symptom severity and mortality.

Another extracted drug was calcitriol, which was not only the hormone form of vitamin D, but also the active form. A pilot randomized clinical trial showed that administration of a high dose of calcifediol or 25-hydroxyvitamin D, a major metabolite of the endocrine system of vitamin D, reduced the need for ICU treatment in patients who had been admitted to the hospital because of a proven COVID-19 (96). Retinoic acid had been shown to improve epithelial and endothelial barrier function and development, and even suppress inflammation-related damage to these barriers by regulating immune cell activity (97). Callaghan et al. (98) found that retinoic acid metabolism was impaired in COVID-19 (cytokine storm) septicemia, so retinoic acid supplementation might be a new perspective for the treatment of COVID-19. In addition, data analysis showed that tamibarotene was selected as a candidate for the treatment of triple mutant virus spike protein against SARS-CoV-2 due to its safety (99). A result of a randomized trial showed that intravenous dexamethasone plus standard treatment compared with standard treatment alone significantly increased the number of days without a ventilator in patients with COVID-19 and moderate or severe ARDS, and the use of dexamethasone may attenuate lung injury in these patients (100). Therefore, the above drugs might be used to treat COVID-19.

It was worth noting that the above results mentioned above, including hub genes, regulatory network, and identification of candidate drugs were based on our bioinformatics calculations and analysis, and basic experiments or clinical trials were still needed to validate these biological functions of hub genes and effectiveness and safety of candidate drugs.

## Conclusions

In this study, DEGs analysis was performed to find out the common DEGs and to determine the disease response of RA and SARS-CoV-2 infection based on two datasets of RA and COVID-19 transcriptome. Bioinformatic analysis showed that RA was a high-risk factor for SARS-CoV-2 infection. Therefore, we used DEGs analysis to obtain 103 common DEGs. Based on the DEGs, we further constructed the PPI network to identify the top ten hub genes. The hub genes retrieved from the DSigDB database indicated multiple drug molecules and drug-targets interaction. In the COVID-19 pandemic, many vaccines are constantly being used, but SARS-CoV-2 continues to form mutants. Only the development of effective COVID-19 vaccines and drugs is an effective blow against COVID-19. The top ten hub genes we identified are all related to COVID-19 and play important roles in various functional mutations that can be used as a new target and perspective for COVID-19 vaccine development.

## Data Availability Statement

The datasets presented in this study can be found in online repositories. The names of the repository/repositories and accession number(s) can be found in the article/**Supplementary Material**.

## Ethics Statement

Ethical review and approval was not required for the study on human participants in accordance with the local legislation and institutional requirements. Written informed consent from the participants’ legal guardian/next of kin was not required to participate in this study in accordance with the national legislation and the institutional requirements.

## Author Contributions

HH, NT, FZ, and LiL designed this study. HH, FZ, and NT collected the transcriptome and clinical data. FZ and LiL took part in the data analysis. HH drafted the manuscript. LoL revised the final manuscript. All authors contributed to the article and approved the submitted version.

## Funding

This work was supported by Guizhou Science and Technology Department Plan Supporting Project of China, Grant No: 2021 General 060.

## Conflict of Interest

The authors declare that the research was conducted in the absence of any commercial or financial relationships that could be construed as a potential conflict of interest.

## Publisher’s Note

All claims expressed in this article are solely those of the authors and do not necessarily represent those of their affiliated organizations, or those of the publisher, the editors and the reviewers. Any product that may be evaluated in this article, or claim that may be made by its manufacturer, is not guaranteed or endorsed by the publisher.

## Supplementary Material

Supplementary Figure 1 Validation of the hub gene by ROC analysis. **(A)** was ROC analysis of RA cohort (GSE17755); **(B)** was ROC analysis of COVID-19 cohort (GSE171110).
