# Supplementary figures and images for: Bioinformatics and System Biology Approach to Identify the Influences of COVID-19 on Rheumatoid Arthritis
Source: Front Immunol. 2022 Apr 7;13:860676. doi: 10.3389/fimmu.2022.860676 (PMC9021444; doi:10.3389/fimmu.2022.860676)

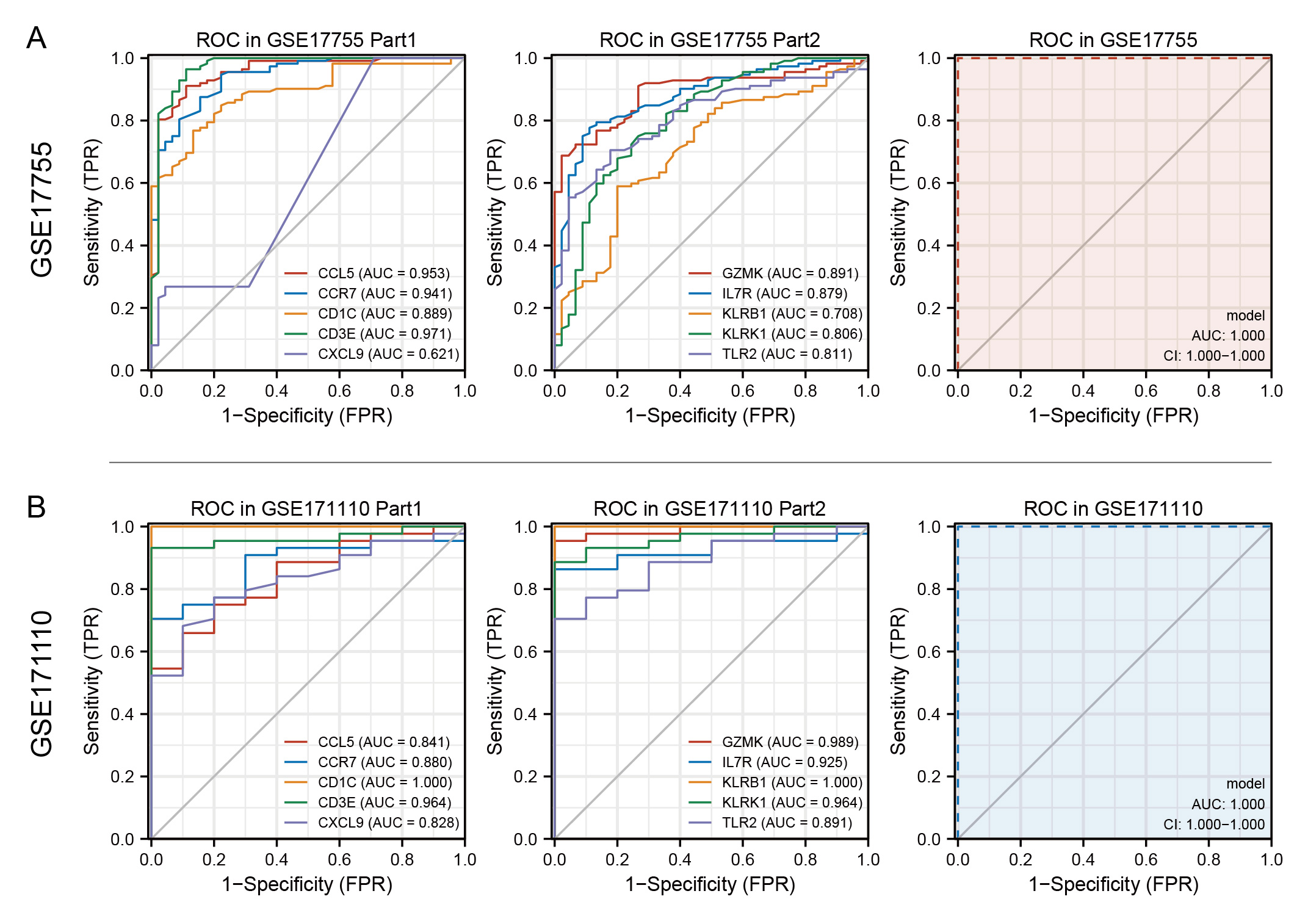

Supplement: Supplementary Figure 1 — Validation of the hub gene by ROC analysis. (A) was ROC analysis of RA cohort (GSE17755); (B) was ROC analysis of COVID-19 cohort (GSE171110). [file Image_1.tif]
