# Supplementary material for: Bioinformatics and System Biology Approach to Identify the Influences of COVID-19 on Rheumatoid Arthritis
Source: Front Immunol. 2022 Apr 7;13:860676. doi: 10.3389/fimmu.2022.860676 (PMC9021444; doi:10.3389/fimmu.2022.860676)
Supplement: Supplementary file 2 [file Table_1.docx]

Table S1. DEGs in GSE17755.

| Genes | logFC | AveExpr | t | P.Value | adj.P.Value | B |
| --- | --- | --- | --- | --- | --- | --- |
| ACTG1 | -1.360975 | -0.99933 | -24.2538 | 2.47E-55 | 3.58E-51 | 115.2004 |
| TXNIP | -2.659873 | -1.88904 | -20.6235 | 8.09E-47 | 5.87E-43 | 95.89378 |
| CFL1 | -1.989655 | -1.24204 | -20.3703 | 3.38E-46 | 1.63E-42 | 94.48361 |
| PFN1 | -1.214272 | -0.82312 | -20.3204 | 4.48E-46 | 1.63E-42 | 94.2046 |
| IL32 | -1.512187 | -0.95854 | -20.25 | 6.68E-46 | 1.94E-42 | 93.81067 |
| PIGL | 0.9137321 | -0.06217 | 19.02403 | 7.71E-43 | 1.87E-39 | 86.84676 |
| CD37 | -0.593977 | -0.38962 | -18.9956 | 9.10E-43 | 1.89E-39 | 86.68324 |
| C22orf30 | 0.8646488 | 0.078153 | 18.34145 | 4.23E-41 | 7.68E-38 | 82.8881 |
| LSP1 | -1.440653 | -0.88395 | -18.3199 | 4.81E-41 | 7.76E-38 | 82.76196 |
| NOC2L | 0.6776349 | 0.373631 | 18.29264 | 5.65E-41 | 8.20E-38 | 82.60288 |
| ARHGDIB | -1.230724 | -0.72019 | -18.2754 | 6.26E-41 | 8.26E-38 | 82.50191 |
| MEOX2 | 0.9335179 | -0.06605 | 18.2272 | 8.32E-41 | 1.01E-37 | 82.21999 |
| LEPRE1 | 0.9840913 | -0.00153 | 17.936 | 4.69E-40 | 5.24E-37 | 80.51007 |
| MRPS10 | 0.9732817 | 0.189427 | 17.69256 | 2.01E-39 | 2.08E-36 | 79.07295 |
| PDE6H | 0.7605873 | 0.09414 | 17.60288 | 3.43E-39 | 3.32E-36 | 78.5418 |
| EDA2R | 0.8914087 | 0.121465 | 17.38148 | 1.30E-38 | 1.18E-35 | 77.22659 |
| PRKD2 | -0.946185 | -0.55032 | -17.2602 | 2.69E-38 | 2.30E-35 | 76.50368 |
| CAPZB | -0.66001 | -0.52306 | -17.1354 | 5.72E-38 | 4.61E-35 | 75.75835 |
| GABRD | 1.0102718 | 0.161592 | 16.94716 | 1.79E-37 | 1.37E-34 | 74.63071 |
| BDKRB1 | 0.7597877 | 0.424459 | 16.88301 | 2.64E-37 | 1.92E-34 | 74.24559 |
| C19orf61 | 0.83825 | 0.245987 | 16.67793 | 9.18E-37 | 6.35E-34 | 73.01132 |
| SLC35A4 | 0.8299206 | 0.254268 | 16.5193 | 2.42E-36 | 1.59E-33 | 72.05361 |
| SLC22A17 | 0.9211052 | 0.11465 | 16.49037 | 2.88E-36 | 1.82E-33 | 71.87866 |
| ATP8 | -0.936361 | -0.76787 | -16.4601 | 3.47E-36 | 2.10E-33 | 71.69533 |
| EEF2 | -0.803177 | -0.60452 | -16.4487 | 3.72E-36 | 2.10E-33 | 71.6263 |
| PARP10 | -0.503448 | -0.35159 | -16.4471 | 3.76E-36 | 2.10E-33 | 71.61713 |
| PPP4C | -1.033022 | -0.59382 | -16.4217 | 4.39E-36 | 2.36E-33 | 71.4631 |
| LCK | -1.080082 | -0.85895 | -16.3734 | 5.90E-36 | 3.06E-33 | 71.17019 |
| NNMT | 0.6837302 | 0.103312 | 16.36226 | 6.31E-36 | 3.16E-33 | 71.10296 |
| C5orf23 | 0.8440992 | 0.230382 | 16.28711 | 1.00E-35 | 4.69E-33 | 70.64714 |
| HTR2B | 0.801506 | 0.109108 | 16.15721 | 2.22E-35 | 1.01E-32 | 69.85796 |
| PRKAA1 | 0.8782123 | 0.086051 | 16.09304 | 3.29E-35 | 1.45E-32 | 69.46751 |
| DNMT3B | 0.7199206 | 0.365796 | 15.98672 | 6.34E-35 | 2.71E-32 | 68.81974 |
| ZNF780B | 0.7854623 | 0.283885 | 15.9182 | 9.67E-35 | 4.01E-32 | 68.40166 |
| NEK6 | 0.9128214 | -0.18682 | 15.88901 | 1.16E-34 | 4.67E-32 | 68.2234 |
| PDK2 | 0.8398472 | 0.364904 | 15.85631 | 1.42E-34 | 5.56E-32 | 68.02369 |
| ARF1 | -0.637976 | -0.50899 | -15.8063 | 1.93E-34 | 7.37E-32 | 67.71803 |
| WDR17 | 0.7443036 | 0.024968 | 15.6856 | 4.07E-34 | 1.51E-31 | 66.97929 |
| HLA-DRA | -1.089867 | -0.93771 | -15.6486 | 5.11E-34 | 1.86E-31 | 66.75265 |
| COTL1 | -1.276871 | -0.66 | -15.6278 | 5.81E-34 | 2.06E-31 | 66.62528 |
| RXRG | 0.8120456 | 0.257516 | 15.54482 | 9.72E-34 | 3.36E-31 | 66.11603 |
| ZYX | -0.903173 | -0.57497 | -15.5236 | 1.11E-33 | 3.72E-31 | 65.98565 |
| EGR1 | 0.6838214 | 0.253822 | 15.52093 | 1.13E-33 | 3.72E-31 | 65.96933 |
| NAGK | 0.5854821 | 0.085669 | 15.47317 | 1.52E-33 | 4.89E-31 | 65.67603 |
| NPIP | -1.213026 | -0.73312 | -15.3989 | 2.40E-33 | 7.59E-31 | 65.21971 |
| TBC1D4 | 0.8102063 | 0.094204 | 15.39474 | 2.47E-33 | 7.62E-31 | 65.19386 |
| CCNE2 | 0.7159524 | 0.174076 | 15.33461 | 3.59E-33 | 1.08E-30 | 64.82384 |
| KLF8 | 0.5324157 | 0.135924 | 15.28743 | 4.81E-33 | 1.42E-30 | 64.53329 |
| IL12RB2 | 0.7979484 | -0.15032 | 15.25553 | 5.86E-33 | 1.70E-30 | 64.33675 |
| CA14 | 0.690629 | 0.263567 | 15.04093 | 2.23E-32 | 6.35E-30 | 63.01249 |
| HSP90AB1 | -1.121704 | -0.85197 | -14.9884 | 3.10E-32 | 8.65E-30 | 62.68789 |
| FAM108A1 | -0.623687 | -0.40085 | -14.9537 | 3.85E-32 | 1.05E-29 | 62.47318 |
| ZNF646 | 0.7952282 | -0.11159 | 14.86991 | 6.50E-32 | 1.71E-29 | 61.95454 |
| TBX19 | 0.8804325 | 0.164968 | 14.77562 | 1.17E-31 | 2.98E-29 | 61.37032 |
| LRPAP1 | -0.907087 | -0.56465 | -14.7658 | 1.25E-31 | 3.12E-29 | 61.30938 |
| HOXA11 | 1.0819563 | 0.210064 | 14.75026 | 1.37E-31 | 3.38E-29 | 61.2131 |
| MFSD2 | 0.5589405 | 0.026401 | 14.69935 | 1.89E-31 | 4.57E-29 | 60.89732 |
| S100A12 | 1.6397421 | 0.971975 | 14.69622 | 1.93E-31 | 4.58E-29 | 60.87788 |
| FAIM3 | -1.198111 | -0.71159 | -14.5875 | 3.81E-31 | 8.77E-29 | 60.20301 |
| HMHA1 | -0.806728 | -0.57261 | -14.5443 | 4.99E-31 | 1.13E-28 | 59.93448 |
| GPR109A | 0.8974821 | 0.076242 | 14.49422 | 6.84E-31 | 1.53E-28 | 59.62315 |
| GUCY1B2 | 0.6962341 | 0.083121 | 14.47554 | 7.69E-31 | 1.69E-28 | 59.50698 |
| NCKAP1L | 0.7263095 | 0.181465 | 14.46171 | 8.38E-31 | 1.82E-28 | 59.42099 |
| IL2RG | -0.979677 | -0.40643 | -14.3683 | 1.51E-30 | 3.22E-28 | 58.83984 |
| MAN1A1 | 0.8930437 | 0.178854 | 14.31487 | 2.11E-30 | 4.44E-28 | 58.5069 |
| C21orf96 | 0.7402083 | 0.214713 | 14.30684 | 2.22E-30 | 4.60E-28 | 58.4569 |
| C22orf24 | 0.7617401 | 0.273185 | 14.26438 | 2.90E-30 | 5.93E-28 | 58.19232 |
| TMEM169 | 0.7933452 | 0.205287 | 14.24654 | 3.24E-30 | 6.54E-28 | 58.08111 |
| KIF25 | 0.6659643 | 0.211083 | 14.23082 | 3.58E-30 | 7.12E-28 | 57.98316 |
| EI24 | 0.853621 | 0.090064 | 14.21705 | 3.90E-30 | 7.66E-28 | 57.89726 |
| CLEC3A | 0.5094067 | 0.26051 | 14.20271 | 4.27E-30 | 8.27E-28 | 57.80785 |
| GNGT2 | 0.6890873 | -0.25287 | 14.18306 | 4.83E-30 | 9.24E-28 | 57.6853 |
| IL10RB | 0.7067738 | 0.011529 | 14.13549 | 6.52E-30 | 1.23E-27 | 57.38854 |
| PSRC1 | 0.8767302 | 0.562994 | 14.1213 | 7.13E-30 | 1.33E-27 | 57.30002 |
| FOXM1 | 0.8029107 | -0.16522 | 14.09911 | 8.20E-30 | 1.49E-27 | 57.16149 |
| PPP1CA | -1.091714 | -0.5728 | -14.0877 | 8.82E-30 | 1.58E-27 | 57.0902 |
| CCNL1 | -0.694475 | -0.37787 | -14.08 | 9.26E-30 | 1.64E-27 | 57.04196 |
| DNMT3L | 0.6948056 | 0.222102 | 14.01865 | 1.36E-29 | 2.38E-27 | 56.65912 |
| BPIL1 | 0.8353393 | -0.17809 | 13.97669 | 1.77E-29 | 3.07E-27 | 56.39702 |
| SERF1A | 0.6548274 | -0.15153 | 13.93573 | 2.30E-29 | 3.88E-27 | 56.14101 |
| CCL13 | 0.571496 | 0.052803 | 13.92295 | 2.49E-29 | 4.16E-27 | 56.06117 |
| ND4L | -1.093319 | -0.5535 | -13.9175 | 2.58E-29 | 4.25E-27 | 56.02709 |
| INPP5F | 0.747129 | 0.139873 | 13.87513 | 3.37E-29 | 5.49E-27 | 55.76218 |
| CPSF4 | 0.76175 | 0.005414 | 13.85387 | 3.85E-29 | 6.21E-27 | 55.6292 |
| CD3E | -0.715349 | -0.34854 | -13.8372 | 4.28E-29 | 6.83E-27 | 55.52482 |
| LRRC3B | 0.5280794 | 0.036497 | 13.82734 | 4.55E-29 | 7.19E-27 | 55.4633 |
| FFAR3 | 0.6577778 | 0.051465 | 13.81556 | 4.91E-29 | 7.66E-27 | 55.38959 |
| FCGR2A | 0.8071448 | 0.311242 | 13.7835 | 6.01E-29 | 9.28E-27 | 55.18898 |
| COX2 | -0.935886 | -0.71608 | -13.7707 | 6.51E-29 | 9.95E-27 | 55.1089 |
| HERPUD1 | 0.6442579 | 0.143376 | 13.74002 | 7.91E-29 | 1.17E-26 | 54.91687 |
| SNRP70 | -0.849317 | -0.52299 | -13.5874 | 2.07E-28 | 3.04E-26 | 53.96111 |
| NPHS2 | 0.9819385 | -0.14529 | 13.54795 | 2.66E-28 | 3.87E-26 | 53.71388 |
| CCL5 | -1.298429 | -0.81427 | -13.5459 | 2.70E-28 | 3.88E-26 | 53.70088 |
| CD74 | -0.81794 | -0.54717 | -13.5062 | 3.47E-28 | 4.94E-26 | 53.45224 |
| TRIM28 | -0.518234 | -0.38414 | -13.4782 | 4.14E-28 | 5.83E-26 | 53.27694 |
| UPB1 | 0.8007341 | 0.475669 | 13.44479 | 5.11E-28 | 7.14E-26 | 53.06716 |
| KCNK3 | -0.614101 | -0.45675 | -13.4374 | 5.36E-28 | 7.41E-26 | 53.021 |
| IARS2 | 0.7811171 | 0.427452 | 13.41599 | 6.14E-28 | 8.41E-26 | 52.88657 |
| ARF3 | 0.8207302 | 0.609045 | 13.3804 | 7.69E-28 | 1.04E-25 | 52.66333 |
| IMPDH1 | -0.5685 | -0.23955 | -13.3592 | 8.79E-28 | 1.18E-25 | 52.53049 |
| HCST | -0.603127 | -0.5907 | -13.329 | 1.06E-27 | 1.42E-25 | 52.34055 |
| RASGRF1 | 0.5533591 | 0.327197 | 13.32619 | 1.08E-27 | 1.43E-25 | 52.32319 |
| LRRC50 | 0.6280635 | 0.124268 | 13.3011 | 1.27E-27 | 1.66E-25 | 52.16573 |
| TDRD1 | 0.5799524 | 0.223057 | 13.29611 | 1.31E-27 | 1.70E-25 | 52.13442 |
| CDK10 | 0.6878393 | 0.126688 | 13.28915 | 1.37E-27 | 1.76E-25 | 52.09072 |
| ENO1 | -0.840875 | -0.57586 | -13.2699 | 1.55E-27 | 1.97E-25 | 51.97009 |
| DHX34 | 0.7786052 | 0.172994 | 13.25715 | 1.68E-27 | 2.12E-25 | 51.8899 |
| CTSW | -0.752762 | -0.57567 | -13.219 | 2.14E-27 | 2.65E-25 | 51.65031 |
| XRCC6 | -0.585141 | -0.64898 | -13.1812 | 2.72E-27 | 3.34E-25 | 51.41306 |
| OLIG2 | -0.515784 | -0.4635 | -13.1688 | 2.94E-27 | 3.58E-25 | 51.33547 |
| EGR4 | 1.0556488 | 0.298408 | 13.15852 | 3.14E-27 | 3.79E-25 | 51.27065 |
| CCND3 | -0.797361 | -0.55771 | -13.1169 | 4.08E-27 | 4.90E-25 | 51.00942 |
| SLC3A2 | 0.7916766 | 0.060318 | 13.07381 | 5.36E-27 | 6.38E-25 | 50.73865 |
| GNAI2 | -0.694318 | -0.66264 | -12.9495 | 1.18E-26 | 1.37E-24 | 49.95743 |
| HSPA8 | -0.741056 | -0.6351 | -12.9234 | 1.39E-26 | 1.60E-24 | 49.79361 |
| C3orf46 | 0.7354365 | 0.18242 | 12.90869 | 1.53E-26 | 1.75E-24 | 49.70118 |
| BAI1 | -0.508605 | -0.23038 | -12.9046 | 1.57E-26 | 1.78E-24 | 49.67544 |
| KIAA1543 | -0.526692 | -0.25484 | -12.873 | 1.92E-26 | 2.14E-24 | 49.47703 |
| OSTM1 | 0.5656111 | 0.230382 | 12.86814 | 1.98E-26 | 2.19E-24 | 49.4463 |
| SERINC1 | -0.85903 | -0.64548 | -12.8403 | 2.36E-26 | 2.60E-24 | 49.27111 |
| GTF2F1 | 0.7727639 | 0.150382 | 12.82463 | 2.61E-26 | 2.83E-24 | 49.17286 |
| CHMP4C | 0.7716488 | 0.099809 | 12.82411 | 2.61E-26 | 2.83E-24 | 49.16958 |
| SFRS3 | -0.971994 | -0.63006 | -12.7603 | 3.92E-26 | 4.21E-24 | 48.76853 |
| TNFSF13B | 1.2931329 | 0.994268 | 12.75654 | 4.01E-26 | 4.28E-24 | 48.74479 |
| CCR7 | -0.750452 | -0.37669 | -12.7412 | 4.43E-26 | 4.69E-24 | 48.64819 |
| SHOX2 | -0.842089 | -0.73873 | -12.7237 | 4.94E-26 | 5.20E-24 | 48.53836 |
| RPN2 | -0.928587 | -0.64599 | -12.6952 | 5.92E-26 | 6.19E-24 | 48.3592 |
| KCTD10 | -0.839673 | -0.56567 | -12.6589 | 7.46E-26 | 7.73E-24 | 48.13122 |
| NCL | -0.637484 | -0.29121 | -12.6463 | 8.08E-26 | 8.32E-24 | 48.0515 |
| RNF167 | -0.670562 | -0.40914 | -12.6081 | 1.03E-25 | 1.05E-23 | 47.81133 |
| AP2M1 | -0.743157 | -0.31726 | -12.5873 | 1.18E-25 | 1.18E-23 | 47.68066 |
| ZFPM2 | 0.6005139 | 0.518503 | 12.57715 | 1.25E-25 | 1.25E-23 | 47.61692 |
| NIN | 0.5442837 | 0.017834 | 12.56118 | 1.39E-25 | 1.38E-23 | 47.51652 |
| IL16 | -0.607163 | -0.54758 | -12.5409 | 1.58E-25 | 1.56E-23 | 47.38929 |
| NDUFV1 | -0.532643 | -0.34531 | -12.5318 | 1.67E-25 | 1.64E-23 | 47.33205 |
| PITX3 | -0.51251 | -0.31783 | -12.5157 | 1.85E-25 | 1.80E-23 | 47.23023 |
| AHNAK2 | 0.5184762 | 0.278535 | 12.50983 | 1.92E-25 | 1.86E-23 | 47.19359 |
| CORO1A | -0.835339 | -0.65191 | -12.4736 | 2.42E-25 | 2.32E-23 | 46.96569 |
| GPR18 | 0.8525496 | 0.609299 | 12.4728 | 2.43E-25 | 2.32E-23 | 46.96075 |
| ENTPD1 | 0.577252 | 0.006242 | 12.44086 | 2.98E-25 | 2.77E-23 | 46.75991 |
| RBM14 | -0.579214 | -0.4272 | -12.3891 | 4.13E-25 | 3.78E-23 | 46.4344 |
| FAM110A | -0.510927 | -0.28204 | -12.3872 | 4.19E-25 | 3.80E-23 | 46.42238 |
| ACTB | -0.736934 | -0.72326 | -12.3833 | 4.29E-25 | 3.84E-23 | 46.39831 |
| DDOST | -0.606246 | -0.48159 | -12.3684 | 4.71E-25 | 4.20E-23 | 46.30446 |
| HSPA9 | -0.964468 | -0.71892 | -12.3553 | 5.12E-25 | 4.54E-23 | 46.22179 |
| CCT7 | -0.594361 | -0.46089 | -12.3442 | 5.50E-25 | 4.84E-23 | 46.15241 |
| SATB1 | -0.755923 | -0.37592 | -12.3422 | 5.57E-25 | 4.87E-23 | 46.13975 |
| SRPR | -0.716028 | -0.41102 | -12.3212 | 6.36E-25 | 5.50E-23 | 46.00771 |
| VEZF1 | -0.545581 | -0.39943 | -12.3212 | 6.36E-25 | 5.50E-23 | 46.00755 |
| SFRS4 | -0.906192 | -0.56357 | -12.3099 | 6.84E-25 | 5.87E-23 | 45.93643 |
| FYN | -1.037556 | -0.54261 | -12.3085 | 6.90E-25 | 5.89E-23 | 45.92774 |
| SLC25A6 | -0.621187 | -0.48392 | -12.2933 | 7.59E-25 | 6.41E-23 | 45.83219 |
| FBLIM1 | 0.5816548 | 0.345605 | 12.22163 | 1.20E-24 | 9.99E-23 | 45.38154 |
| MTCH1 | -0.583413 | -0.44064 | -12.2001 | 1.37E-24 | 1.14E-22 | 45.24646 |
| ADD3 | -1.414071 | -0.94076 | -12.198 | 1.39E-24 | 1.15E-22 | 45.2328 |
| C1orf133 | 0.6940357 | 0.349108 | 12.05205 | 3.51E-24 | 2.86E-22 | 44.31573 |
| RAC2 | -0.571691 | -0.36672 | -12.0458 | 3.65E-24 | 2.96E-22 | 44.27651 |
| CD97 | -1.137054 | -0.87115 | -12.0309 | 4.02E-24 | 3.24E-22 | 44.18255 |
| C5orf28 | 0.579746 | 0.436688 | 12.00707 | 4.67E-24 | 3.72E-22 | 44.03309 |
| UCP2 | -0.711389 | -0.2786 | -12.0014 | 4.84E-24 | 3.84E-22 | 43.99726 |
| TUFM | -0.511909 | -0.32274 | -11.9623 | 6.20E-24 | 4.84E-22 | 43.75211 |
| SNAI1 | 0.5398849 | 0.189363 | 11.94592 | 6.88E-24 | 5.31E-22 | 43.64895 |
| ZNF714 | -0.830026 | -0.5379 | -11.9206 | 8.08E-24 | 6.18E-22 | 43.48974 |
| NDUFS4 | 0.7893472 | 0.776879 | 11.90695 | 8.81E-24 | 6.70E-22 | 43.40418 |
| RPL8 | -0.75671 | -0.61293 | -11.8736 | 1.09E-23 | 8.23E-22 | 43.19461 |
| APOA4 | 0.585506 | 0.111019 | 11.86544 | 1.15E-23 | 8.58E-22 | 43.1435 |
| PSMD8 | -0.731806 | -0.3565 | -11.8593 | 1.19E-23 | 8.88E-22 | 43.10479 |
| GPI | -0.725026 | -0.45299 | -11.8285 | 1.45E-23 | 1.07E-21 | 42.91129 |
| FAM102A | -0.71872 | -0.45605 | -11.7978 | 1.76E-23 | 1.29E-21 | 42.7188 |
| CD79B | -0.690495 | -0.39347 | -11.7601 | 2.24E-23 | 1.61E-21 | 42.48193 |
| C2orf28 | -0.504494 | -0.49156 | -11.7473 | 2.42E-23 | 1.73E-21 | 42.40216 |
| TMEM30A | 0.7180942 | 0.415382 | 11.74337 | 2.49E-23 | 1.77E-21 | 42.37723 |
| BAT1 | -0.801063 | -0.40968 | -11.7266 | 2.76E-23 | 1.96E-21 | 42.27193 |
| ARMCX6 | 0.5101389 | 0.025032 | 11.69216 | 3.44E-23 | 2.42E-21 | 42.05586 |
| PNMA1 | 0.7332044 | 0.520828 | 11.6875 | 3.54E-23 | 2.47E-21 | 42.02663 |
| SOCS2 | 0.743246 | 0.457325 | 11.6492 | 4.51E-23 | 3.11E-21 | 41.78638 |
| ICAM2 | -0.724794 | -0.40083 | -11.5645 | 7.72E-23 | 5.23E-21 | 41.25538 |
| S100A9 | 0.9979444 | 0.581465 | 11.53803 | 9.12E-23 | 6.13E-21 | 41.08938 |
| CCDC9 | -0.562536 | -0.3893 | -11.5324 | 9.45E-23 | 6.32E-21 | 41.05431 |
| SERBP1 | -0.533766 | -0.33233 | -11.4676 | 1.43E-22 | 9.45E-21 | 40.64783 |
| C3orf59 | 0.6520675 | 0.40828 | 11.44566 | 1.64E-22 | 1.08E-20 | 40.51064 |
| HLA-E | -1.221115 | -1.05089 | -11.4449 | 1.64E-22 | 1.08E-20 | 40.50613 |
| ALG10 | 0.658629 | 0.062739 | 11.41348 | 2.01E-22 | 1.30E-20 | 40.30913 |
| BANK1 | -0.7347 | -0.49701 | -11.4082 | 2.07E-22 | 1.33E-20 | 40.27602 |
| TGFB1 | -0.758966 | -0.33987 | -11.3884 | 2.35E-22 | 1.50E-20 | 40.15183 |
| SLC38A3 | 0.6513373 | 0.154204 | 11.36739 | 2.68E-22 | 1.69E-20 | 40.02058 |
| MOBKL2A | 0.5404405 | 0.084204 | 11.36608 | 2.71E-22 | 1.69E-20 | 40.01234 |
| CCDC103 | 0.7276171 | -0.09471 | 11.35814 | 2.85E-22 | 1.77E-20 | 39.96266 |
| ANXA6 | -1.021835 | -0.78006 | -11.3438 | 3.12E-22 | 1.93E-20 | 39.87291 |
| C1orf75 | 0.5390317 | 0.167643 | 11.33987 | 3.19E-22 | 1.97E-20 | 39.84837 |
| CEBPB | 0.7616964 | -0.02662 | 11.33973 | 3.20E-22 | 1.97E-20 | 39.84748 |
| ZSWIM4 | 0.5952877 | 0.119108 | 11.33433 | 3.31E-22 | 2.03E-20 | 39.81366 |
| CAPNS1 | -0.763746 | -0.38395 | -11.3289 | 3.42E-22 | 2.09E-20 | 39.77989 |
| CD27 | -0.834881 | -0.58892 | -11.3164 | 3.71E-22 | 2.25E-20 | 39.70169 |
| ERAF | 1.2960337 | 0.906115 | 11.24541 | 5.80E-22 | 3.47E-20 | 39.25749 |
| CA1 | 1.1569444 | 0.860892 | 11.24427 | 5.84E-22 | 3.47E-20 | 39.25034 |
| PANK4 | 0.8356171 | 0.084331 | 11.24351 | 5.87E-22 | 3.47E-20 | 39.2456 |
| TAS2R1 | 0.6855794 | -0.30115 | 11.2431 | 5.89E-22 | 3.47E-20 | 39.24305 |
| TMEM41B | 0.6231706 | 0.354777 | 11.22726 | 6.51E-22 | 3.82E-20 | 39.14402 |
| SFRS5 | -1.081121 | -0.84236 | -11.2165 | 6.97E-22 | 4.07E-20 | 39.07675 |
| ABTB1 | -0.656375 | -0.44624 | -11.2153 | 7.02E-22 | 4.07E-20 | 39.06919 |
| HS3ST6 | -0.555335 | -0.34127 | -11.2142 | 7.07E-22 | 4.07E-20 | 39.06258 |
| PARVG | -0.643514 | -0.42618 | -11.2125 | 7.14E-22 | 4.10E-20 | 39.05152 |
| RMND5A | 0.6525913 | 0.415987 | 11.20251 | 7.61E-22 | 4.33E-20 | 38.98932 |
| C14orf172 | 0.5478036 | 0.09879 | 11.19339 | 8.06E-22 | 4.57E-20 | 38.93232 |
| MFN1 | 0.6857321 | 0.293185 | 11.19106 | 8.18E-22 | 4.62E-20 | 38.91773 |
| RPL3 | -0.993577 | -0.78013 | -11.1695 | 9.37E-22 | 5.27E-20 | 38.7833 |
| NR4A2 | 0.7261091 | -0.11357 | 11.14375 | 1.10E-21 | 6.18E-20 | 38.62215 |
| ADRA1D | 0.7510159 | 0.113312 | 11.1279 | 1.22E-21 | 6.80E-20 | 38.52318 |
| HLA-DPB1 | -0.723221 | -0.58748 | -11.0632 | 1.83E-21 | 1.01E-19 | 38.11921 |
| LY6G5C | 0.6765536 | 0.250637 | 11.05466 | 1.93E-21 | 1.06E-19 | 38.06598 |
| UBE1 | 0.7943571 | 0.622675 | 11.04986 | 1.99E-21 | 1.09E-19 | 38.03601 |
| CAPN2 | -0.794262 | -0.52127 | -11.0457 | 2.05E-21 | 1.11E-19 | 38.00984 |
| MAT2B | -0.936452 | -0.69338 | -11.0435 | 2.07E-21 | 1.12E-19 | 37.99622 |
| TCL1A | -0.864751 | -0.69452 | -11.0334 | 2.21E-21 | 1.19E-19 | 37.9335 |
| KIAA0174 | -1.016399 | -0.77841 | -10.988 | 2.94E-21 | 1.56E-19 | 37.65017 |
| IRS4 | 0.8203968 | 0.63414 | 10.98636 | 2.97E-21 | 1.58E-19 | 37.63992 |
| HECW2 | 0.5308175 | 0.111783 | 10.96348 | 3.43E-21 | 1.81E-19 | 37.49732 |
| CDKN1C | -0.52306 | -0.23847 | -10.9299 | 4.24E-21 | 2.21E-19 | 37.28805 |
| FXYD5 | -0.629613 | -0.28248 | -10.9267 | 4.33E-21 | 2.24E-19 | 37.26793 |
| AMT | 0.7278135 | 0.399427 | 10.85796 | 6.67E-21 | 3.42E-19 | 36.83997 |
| CCM2 | -0.635002 | -0.44344 | -10.8445 | 7.26E-21 | 3.71E-19 | 36.75616 |
| C20orf118 | 0.6100298 | 0.221847 | 10.84233 | 7.36E-21 | 3.75E-19 | 36.74267 |
| RBL2 | -0.782188 | -0.74822 | -10.8382 | 7.55E-21 | 3.82E-19 | 36.7171 |
| KRT3 | 0.5342956 | 0.026709 | 10.79749 | 9.76E-21 | 4.92E-19 | 36.46373 |
| IGSF8 | 0.6920337 | 0.049236 | 10.78473 | 1.06E-20 | 5.31E-19 | 36.38436 |
| RPL29 | -0.919323 | -0.60427 | -10.7093 | 1.70E-20 | 8.44E-19 | 35.91552 |
| PUM1 | -0.802524 | -0.63783 | -10.6878 | 1.94E-20 | 9.63E-19 | 35.7819 |
| XCR1 | 0.5260298 | -0.02408 | 10.65893 | 2.33E-20 | 1.15E-18 | 35.60271 |
| CLEC4E | 0.7228859 | 0.512134 | 10.61569 | 3.05E-20 | 1.49E-18 | 35.33432 |
| H3F3A | 0.8109067 | 0.111592 | 10.59817 | 3.41E-20 | 1.65E-18 | 35.22569 |
| PDIA3 | -0.707596 | -0.37134 | -10.5771 | 3.89E-20 | 1.87E-18 | 35.09481 |
| CHMP7 | -0.769369 | -0.49752 | -10.5715 | 4.03E-20 | 1.93E-18 | 35.06021 |
| SELPLG | -0.580403 | -0.48427 | -10.5522 | 4.55E-20 | 2.16E-18 | 34.94089 |
| DOC2B | 0.6130317 | 0.636433 | 10.54635 | 4.72E-20 | 2.24E-18 | 34.90442 |
| MAOA | -0.582054 | -0.41522 | -10.5346 | 5.08E-20 | 2.38E-18 | 34.83138 |
| NRF1 | 0.7067222 | 0.459936 | 10.53429 | 5.09E-20 | 2.38E-18 | 34.82966 |
| LRCH4 | 0.5958492 | 0.285287 | 10.51783 | 5.64E-20 | 2.63E-18 | 34.72773 |
| HNRPA2B1 | -0.937065 | -0.69115 | -10.5148 | 5.75E-20 | 2.67E-18 | 34.70901 |
| BTN2A3 | 0.720619 | 0.132739 | 10.49463 | 6.52E-20 | 3.01E-18 | 34.58402 |
| FOXO3 | -0.52274 | -0.15669 | -10.4382 | 9.28E-20 | 4.25E-18 | 34.23452 |
| CHPT1 | 0.9036567 | 0.573758 | 10.40237 | 1.16E-19 | 5.28E-18 | 34.01326 |
| HIGD2A | -0.694867 | -0.39592 | -10.3985 | 1.19E-19 | 5.39E-18 | 33.98937 |
| OR52A1 | 0.6458353 | 0.107834 | 10.37134 | 1.41E-19 | 6.33E-18 | 33.8215 |
| CDK5RAP3 | -0.537304 | -0.3793 | -10.3459 | 1.65E-19 | 7.31E-18 | 33.6644 |
| GNB2 | -0.516242 | -0.3765 | -10.3433 | 1.68E-19 | 7.41E-18 | 33.64837 |
| HLA-DPA1 | -0.586571 | -0.61045 | -10.317 | 1.98E-19 | 8.68E-18 | 33.48567 |
| LIME1 | -0.574329 | -0.42949 | -10.3055 | 2.13E-19 | 9.29E-18 | 33.41501 |
| PARP1 | -0.836671 | -0.66108 | -10.2635 | 2.76E-19 | 1.19E-17 | 33.15589 |
| EIF3S8 | -0.653081 | -0.44611 | -10.2437 | 3.12E-19 | 1.34E-17 | 33.03367 |
| CDIPT | -0.69299 | -0.39414 | -10.1844 | 4.52E-19 | 1.91E-17 | 32.66841 |
| HDAC1 | -0.617091 | -0.37866 | -10.17 | 4.94E-19 | 2.07E-17 | 32.58009 |
| LRRC39 | 0.5500694 | 0.597962 | 10.10245 | 7.52E-19 | 3.12E-17 | 32.16442 |
| ACAT2 | 0.5028631 | 6.37E-05 | 10.07287 | 9.04E-19 | 3.71E-17 | 31.9827 |
| DENND2D | -0.725407 | -0.5686 | -10.0697 | 9.22E-19 | 3.77E-17 | 31.96331 |
| AES | -0.684752 | -0.42293 | -10.0482 | 1.05E-18 | 4.27E-17 | 31.83149 |
| EIF4G2 | -0.631655 | -0.33127 | -10.0438 | 1.08E-18 | 4.38E-17 | 31.80418 |
| IL1R2 | 1.1731091 | 0.723312 | 10.04212 | 1.09E-18 | 4.41E-17 | 31.79389 |
| ATP9B | 0.7784087 | 0.418854 | 10.03895 | 1.12E-18 | 4.49E-17 | 31.7744 |
| SCPEP1 | -0.672355 | -0.3772 | -10.0181 | 1.27E-18 | 5.08E-17 | 31.64628 |
| HBE1 | 0.6014286 | 0.119045 | 10.00752 | 1.36E-18 | 5.40E-17 | 31.58156 |
| PPP2R1A | -0.645742 | -0.30688 | -9.98334 | 1.58E-18 | 6.23E-17 | 31.43332 |
| CCDC101 | -0.535389 | -0.19904 | -9.97699 | 1.64E-18 | 6.47E-17 | 31.39439 |
| LENG4 | -0.72301 | -0.43 | -9.97384 | 1.67E-18 | 6.56E-17 | 31.37508 |
| TXNDC5 | -0.741659 | -0.48064 | -9.97335 | 1.68E-18 | 6.56E-17 | 31.37206 |
| DET1 | 0.5304325 | 0.455287 | 9.972875 | 1.68E-18 | 6.56E-17 | 31.36917 |
| RBBP4 | -0.810077 | -0.47522 | -9.95933 | 1.83E-18 | 7.06E-17 | 31.28615 |
| IL10RA | -0.654444 | -0.41242 | -9.95208 | 1.91E-18 | 7.35E-17 | 31.24175 |
| ACTR3 | -0.930621 | -0.59299 | -9.94508 | 2.00E-18 | 7.63E-17 | 31.19888 |
| HTATIP | -0.50426 | -0.24395 | -9.91733 | 2.37E-18 | 8.92E-17 | 31.02899 |
| WARS | -0.772958 | -0.71408 | -9.9133 | 2.43E-18 | 9.12E-17 | 31.00432 |
| RBBP7 | -0.503026 | -0.20662 | -9.90136 | 2.62E-18 | 9.80E-17 | 30.93124 |
| JAK1 | -0.714685 | -0.70917 | -9.88316 | 2.93E-18 | 1.09E-16 | 30.8199 |
| SNX9 | 0.5504048 | 0.243312 | 9.866666 | 3.25E-18 | 1.20E-16 | 30.71904 |
| IMPDH2 | -0.878373 | -0.60217 | -9.84923 | 3.61E-18 | 1.34E-16 | 30.61249 |
| SFRS2 | -0.746425 | -0.57159 | -9.81517 | 4.46E-18 | 1.63E-16 | 30.40443 |
| POLD2 | -0.609119 | -0.4772 | -9.80733 | 4.68E-18 | 1.70E-16 | 30.35652 |
| HCFC1 | -0.541153 | -0.34427 | -9.80333 | 4.80E-18 | 1.74E-16 | 30.33211 |
| ATRN | 0.5841171 | 0.278917 | 9.798297 | 4.95E-18 | 1.79E-16 | 30.3014 |
| KCNJ13 | 0.5394484 | 0.151274 | 9.78874 | 5.25E-18 | 1.89E-16 | 30.24308 |
| ABCB7 | 0.6410813 | 0.399554 | 9.786465 | 5.33E-18 | 1.91E-16 | 30.2292 |
| DCTN5 | 0.5129048 | 0.286561 | 9.784702 | 5.38E-18 | 1.92E-16 | 30.21844 |
| DBT | 0.5788036 | 0.324904 | 9.783697 | 5.42E-18 | 1.93E-16 | 30.2123 |
| EIF3S7 | -0.803708 | -0.59401 | -9.7584 | 6.33E-18 | 2.24E-16 | 30.058 |
| PTDSS1 | -0.51044 | -0.38889 | -9.75813 | 6.34E-18 | 2.24E-16 | 30.05638 |
| CTSB | -0.836214 | -0.45854 | -9.7577 | 6.36E-18 | 2.24E-16 | 30.05375 |
| ACADVL | -0.511619 | -0.34764 | -9.74825 | 6.74E-18 | 2.36E-16 | 29.99613 |
| SERPINB1 | 0.8009266 | 0.398917 | 9.735212 | 7.31E-18 | 2.55E-16 | 29.91667 |
| CDC2L1 | -0.620415 | -0.33548 | -9.69134 | 9.57E-18 | 3.29E-16 | 29.64946 |
| KIAA1853 | 0.6447917 | 0.043312 | 9.668777 | 1.10E-17 | 3.74E-16 | 29.51217 |
| CSAG2 | 0.5762679 | 0.715096 | 9.665301 | 1.12E-17 | 3.81E-16 | 29.49103 |
| B3GNT8 | 0.6382778 | -0.31045 | 9.660465 | 1.16E-17 | 3.91E-16 | 29.46161 |
| TMEM66 | -0.845597 | -0.78701 | -9.64962 | 1.24E-17 | 4.16E-16 | 29.39569 |
| HVCN1 | -0.88206 | -0.70057 | -9.64702 | 1.26E-17 | 4.22E-16 | 29.37983 |
| RPA1 | -0.673405 | -0.35306 | -9.62335 | 1.45E-17 | 4.87E-16 | 29.236 |
| CPSF3L | -0.522136 | -0.20092 | -9.61923 | 1.49E-17 | 4.98E-16 | 29.21098 |
| EVL | -1.104812 | -0.85592 | -9.61604 | 1.52E-17 | 5.07E-16 | 29.19156 |
| CYTB | -0.725329 | -0.71121 | -9.60648 | 1.61E-17 | 5.35E-16 | 29.13349 |
| MAOB | 0.7743036 | 0.186369 | 9.560917 | 2.13E-17 | 7.01E-16 | 28.85696 |
| PGAM1 | -0.643109 | -0.46522 | -9.55111 | 2.27E-17 | 7.40E-16 | 28.7975 |
| MAMDC4 | 0.5913512 | -0.01548 | 9.540132 | 2.43E-17 | 7.86E-16 | 28.73092 |
| DCPS | -0.510913 | -0.30892 | -9.53935 | 2.44E-17 | 7.88E-16 | 28.72616 |
| RBBP9 | 0.5990883 | 0.413153 | 9.538873 | 2.44E-17 | 7.88E-16 | 28.72329 |
| CLGN | 0.5540337 | 0.48879 | 9.499453 | 3.11E-17 | 9.93E-16 | 28.48446 |
| MYL9 | -0.507401 | -0.21975 | -9.46491 | 3.85E-17 | 1.22E-15 | 28.27539 |
| BPNT1 | 0.5080159 | 0.437962 | 9.432261 | 4.70E-17 | 1.48E-15 | 28.078 |
| MIF | -0.712954 | -0.46038 | -9.41359 | 5.26E-17 | 1.64E-15 | 27.96521 |
| PHB2 | -0.684278 | -0.47637 | -9.41164 | 5.33E-17 | 1.66E-15 | 27.95341 |
| ATP6 | -0.714883 | -0.61376 | -9.38977 | 6.09E-17 | 1.87E-15 | 27.82138 |
| RPS6KA1 | -0.588847 | -0.49185 | -9.38936 | 6.10E-17 | 1.87E-15 | 27.81886 |
| SFRS16 | -0.697119 | -0.58197 | -9.38399 | 6.31E-17 | 1.92E-15 | 27.78646 |
| PDE7A | -0.689524 | -0.50522 | -9.35557 | 7.50E-17 | 2.26E-15 | 27.61503 |
| ARL6IP5 | -0.903496 | -0.73764 | -9.34985 | 7.77E-17 | 2.33E-15 | 27.58059 |
| PPIB | -0.507147 | -0.27268 | -9.33318 | 8.60E-17 | 2.57E-15 | 27.48009 |
| ARHGEF1 | -0.751046 | -0.36 | -9.31689 | 9.50E-17 | 2.84E-15 | 27.38196 |
| ENPP4 | 0.6037718 | 0.325605 | 9.316317 | 9.53E-17 | 2.84E-15 | 27.37852 |
| SEMA3A | 0.6348016 | 0.478408 | 9.303671 | 1.03E-16 | 3.05E-15 | 27.30238 |
| C3orf34 | 0.545875 | 0.365414 | 9.298431 | 1.06E-16 | 3.14E-15 | 27.27083 |
| RPSA | -0.784795 | -0.65385 | -9.29716 | 1.07E-16 | 3.16E-15 | 27.26319 |
| GNG2 | 0.9981905 | 0.746752 | 9.29136 | 1.11E-16 | 3.27E-15 | 27.22828 |
| PDCD4 | -0.555121 | -0.32312 | -9.26032 | 1.34E-16 | 3.91E-15 | 27.04156 |
| ILVBL | 0.583631 | 0.279682 | 9.255648 | 1.38E-16 | 4.01E-15 | 27.0135 |
| ADPGK | -0.919841 | -0.71064 | -9.24529 | 1.47E-16 | 4.25E-15 | 26.95123 |
| P4HB | -0.626433 | -0.37777 | -9.24515 | 1.47E-16 | 4.25E-15 | 26.95042 |
| C11orf2 | -0.591298 | -0.43248 | -9.2406 | 1.51E-16 | 4.35E-15 | 26.92309 |
| C14orf131 | 0.5206558 | 0.228312 | 9.233136 | 1.58E-16 | 4.55E-15 | 26.87823 |
| MAF1 | -0.627135 | -0.36561 | -9.22344 | 1.68E-16 | 4.79E-15 | 26.81999 |
| COX17 | 0.5685615 | 0.493376 | 9.220687 | 1.71E-16 | 4.87E-15 | 26.80347 |
| PTPN22 | 0.5837917 | 0.445796 | 9.202359 | 1.91E-16 | 5.42E-15 | 26.69347 |
| RHAG | 0.5272222 | 0.368885 | 9.194555 | 2.00E-16 | 5.66E-15 | 26.64664 |
| GLRX5 | 0.8581845 | 0.725541 | 9.177371 | 2.22E-16 | 6.22E-15 | 26.54358 |
| C8G | 0.648756 | 0.43414 | 9.167375 | 2.36E-16 | 6.58E-15 | 26.48367 |
| NDUFA10 | -0.689756 | -0.46739 | -9.13722 | 2.83E-16 | 7.86E-15 | 26.30304 |
| RPL18 | -0.756657 | -0.72089 | -9.12325 | 3.08E-16 | 8.52E-15 | 26.21941 |
| SLC22A5 | 0.6050933 | 0.452548 | 9.117458 | 3.19E-16 | 8.81E-15 | 26.18473 |
| TMED10 | -0.563142 | -0.47551 | -9.11263 | 3.29E-16 | 9.00E-15 | 26.15582 |
| GYPA | 0.8031012 | 0.34758 | 9.084903 | 3.89E-16 | 1.06E-14 | 25.99005 |
| RAB9A | 0.5130992 | 0.410255 | 9.082439 | 3.95E-16 | 1.07E-14 | 25.97532 |
| EEF1A1 | -1.149901 | -0.99809 | -9.07401 | 4.15E-16 | 1.12E-14 | 25.92492 |
| KLF10 | 0.5252163 | 0.453121 | 9.060059 | 4.52E-16 | 1.22E-14 | 25.84162 |
| IL7R | -0.75048 | -0.56293 | -9.0234 | 5.64E-16 | 1.50E-14 | 25.62283 |
| IGJ | -1.176794 | -0.80127 | -9.00306 | 6.38E-16 | 1.69E-14 | 25.50158 |
| NOB1 | -0.670734 | -0.50293 | -8.98402 | 7.15E-16 | 1.88E-14 | 25.38813 |
| CALCOCO2 | 0.5373472 | 0.369108 | 8.978786 | 7.38E-16 | 1.94E-14 | 25.35697 |
| RTF1 | 0.6949444 | 0.203312 | 8.971976 | 7.69E-16 | 2.01E-14 | 25.31643 |
| GMFG | 0.6248532 | 0.132866 | 8.971799 | 7.70E-16 | 2.01E-14 | 25.31537 |
| IRF8 | -0.512319 | -0.47503 | -8.96458 | 8.04E-16 | 2.09E-14 | 25.2724 |
| GAPDH | -0.890143 | -0.50066 | -8.96077 | 8.23E-16 | 2.13E-14 | 25.24971 |
| AHSA1 | -0.62446 | -0.48459 | -8.95712 | 8.41E-16 | 2.17E-14 | 25.22801 |
| DNAJB4 | 0.577756 | 0.46949 | 8.956452 | 8.45E-16 | 2.17E-14 | 25.22404 |
| SLC25A5 | -0.824875 | -0.67045 | -8.94558 | 9.02E-16 | 2.32E-14 | 25.15936 |
| BTG1 | -0.854859 | -0.33828 | -8.93938 | 9.36E-16 | 2.40E-14 | 25.12247 |
| TBCB | -0.620714 | -0.4428 | -8.89644 | 1.21E-15 | 3.09E-14 | 24.86737 |
| ID2 | -0.922254 | -0.6428 | -8.87813 | 1.35E-15 | 3.43E-14 | 24.75873 |
| CYP2U1 | 0.5206746 | 0.072548 | 8.866625 | 1.45E-15 | 3.66E-14 | 24.69046 |
| KIAA1279 | 0.6689286 | 0.327197 | 8.81945 | 1.92E-15 | 4.83E-14 | 24.41096 |
| APH1B | 0.611752 | 0.268854 | 8.818182 | 1.94E-15 | 4.85E-14 | 24.40345 |
| TAP1 | -0.562419 | -0.45299 | -8.81496 | 1.98E-15 | 4.94E-14 | 24.38436 |
| GNLY | -1.085746 | -0.84166 | -8.80407 | 2.11E-15 | 5.26E-14 | 24.31993 |
| CD1C | -0.810034 | -0.60541 | -8.77759 | 2.47E-15 | 6.06E-14 | 24.16335 |
| EIF4G3 | 0.6421409 | 0.447643 | 8.764527 | 2.67E-15 | 6.53E-14 | 24.08619 |
| SMARCA4 | -0.811931 | -0.69166 | -8.73584 | 3.18E-15 | 7.70E-14 | 23.91685 |
| RBM5 | -0.530659 | -0.55611 | -8.72937 | 3.30E-15 | 7.97E-14 | 23.87866 |
| CAT | -0.830821 | -0.70169 | -8.72668 | 3.35E-15 | 8.06E-14 | 23.86281 |
| HLA-DMA | -0.555909 | -0.49013 | -8.71207 | 3.66E-15 | 8.78E-14 | 23.77668 |
| HNRPDL | -0.639187 | -0.50376 | -8.67454 | 4.58E-15 | 1.08E-13 | 23.5556 |
| MARCO | 0.6401944 | 0.380255 | 8.662079 | 4.93E-15 | 1.16E-13 | 23.48228 |
| ANKRD50 | 0.544506 | 0.437771 | 8.658953 | 5.03E-15 | 1.18E-13 | 23.46389 |
| ZNF268 | 0.5656022 | 0.473376 | 8.654025 | 5.18E-15 | 1.21E-13 | 23.43491 |
| TRAP1 | -0.640609 | -0.49344 | -8.62122 | 6.29E-15 | 1.46E-13 | 23.24214 |
| THOC3 | -0.811687 | -0.46682 | -8.61367 | 6.58E-15 | 1.52E-13 | 23.1978 |
| LIN7A | 0.5491032 | 0.310828 | 8.599519 | 7.16E-15 | 1.64E-13 | 23.11473 |
| UROD | 0.5740139 | 0.128599 | 8.598249 | 7.21E-15 | 1.65E-13 | 23.10728 |
| SCP2 | -0.691042 | -0.54631 | -8.59634 | 7.30E-15 | 1.67E-13 | 23.0961 |
| PDCD6IP | -0.806337 | -0.59478 | -8.59216 | 7.48E-15 | 1.71E-13 | 23.07159 |
| ATP6AP2 | -0.752144 | -0.64178 | -8.56387 | 8.85E-15 | 2.01E-13 | 22.90575 |
| GZMK | -0.802242 | -0.40452 | -8.54938 | 9.65E-15 | 2.18E-13 | 22.82084 |
| NELL2 | -0.512091 | -0.38376 | -8.54007 | 1.02E-14 | 2.30E-13 | 22.76637 |
| TMEM131 | -0.533639 | -0.4058 | -8.53553 | 1.05E-14 | 2.36E-13 | 22.73981 |
| GNB2L1 | -0.722552 | -0.60701 | -8.53544 | 1.05E-14 | 2.36E-13 | 22.73927 |
| RPA2 | -0.545448 | -0.41955 | -8.53481 | 1.05E-14 | 2.36E-13 | 22.73558 |
| DHX15 | -0.728788 | -0.47834 | -8.51859 | 1.16E-14 | 2.59E-13 | 22.64072 |
| VIL2 | -0.597589 | -0.35631 | -8.49039 | 1.37E-14 | 3.05E-13 | 22.47592 |
| NCOA4 | -0.783812 | -0.82293 | -8.47178 | 1.53E-14 | 3.38E-13 | 22.36728 |
| TLN1 | -0.576413 | -0.39764 | -8.4694 | 1.55E-14 | 3.42E-13 | 22.35337 |
| REPIN1 | 0.5709306 | 0.245732 | 8.458018 | 1.66E-14 | 3.63E-13 | 22.287 |
| CKLF | 0.5710843 | 0.325287 | 8.456767 | 1.67E-14 | 3.66E-13 | 22.27971 |
| THEG | -0.630988 | -0.18146 | -8.45233 | 1.71E-14 | 3.74E-13 | 22.25383 |
| PPT1 | -0.81431 | -0.72624 | -8.41847 | 2.09E-14 | 4.48E-13 | 22.05659 |
| ZNF557 | 0.6138294 | 0.465669 | 8.398196 | 2.36E-14 | 4.99E-13 | 21.93861 |
| YIPF5 | -0.80231 | -0.52968 | -8.38697 | 2.52E-14 | 5.31E-13 | 21.87334 |
| RPS19 | -0.767069 | -0.59076 | -8.38255 | 2.59E-14 | 5.44E-13 | 21.84763 |
| ROPN1L | 0.8867956 | 0.494841 | 8.381288 | 2.61E-14 | 5.48E-13 | 21.84031 |
| EIF3S6IP | -0.560081 | -0.50777 | -8.36972 | 2.79E-14 | 5.82E-13 | 21.77309 |
| QPCT | 0.5886131 | 0.379236 | 8.36361 | 2.89E-14 | 6.00E-13 | 21.73762 |
| ATP6V1F | -0.603312 | -0.35217 | -8.35286 | 3.08E-14 | 6.34E-13 | 21.67518 |
| DDX23 | -0.633071 | -0.63962 | -8.34781 | 3.18E-14 | 6.50E-13 | 21.64592 |
| MED18 | 0.5690417 | 0.411274 | 8.286014 | 4.57E-14 | 9.17E-13 | 21.28785 |
| FNBP1L | 0.542503 | 0.362675 | 8.269256 | 5.04E-14 | 1.00E-12 | 21.19093 |
| MOSPD1 | 0.5301687 | 0.625987 | 8.267231 | 5.10E-14 | 1.02E-12 | 21.17922 |
| SNX17 | -0.533675 | -0.47382 | -8.25887 | 5.35E-14 | 1.06E-12 | 21.1309 |
| GPX4 | -0.59275 | -0.34885 | -8.25692 | 5.42E-14 | 1.07E-12 | 21.11964 |
| RING1 | -0.74324 | -0.40599 | -8.24612 | 5.77E-14 | 1.14E-12 | 21.05724 |
| BAI2 | -0.521056 | -0.11815 | -8.23952 | 6.00E-14 | 1.18E-12 | 21.01914 |
| LSM2 | -0.697323 | -0.2279 | -8.21597 | 6.88E-14 | 1.34E-12 | 20.8833 |
| HNRPM | -0.706294 | -0.42363 | -8.21155 | 7.06E-14 | 1.37E-12 | 20.85779 |
| ISGF3G | -0.51304 | -0.08688 | -8.20948 | 7.15E-14 | 1.39E-12 | 20.84588 |
| STK4 | 0.5123899 | 0.14086 | 8.204787 | 7.35E-14 | 1.42E-12 | 20.81881 |
| SFRS1 | -1.070992 | -0.77624 | -8.19967 | 7.57E-14 | 1.46E-12 | 20.7893 |
| NKX1-1 | -0.564373 | -0.23217 | -8.18468 | 8.27E-14 | 1.59E-12 | 20.70301 |
| LCP1 | -1.420986 | -1.33459 | -8.17809 | 8.59E-14 | 1.64E-12 | 20.66507 |
| EIF3S2 | -0.667234 | -0.48643 | -8.17678 | 8.66E-14 | 1.65E-12 | 20.65752 |
| EIF2B2 | -0.565073 | -0.38955 | -8.1718 | 8.91E-14 | 1.70E-12 | 20.62885 |
| PGK1 | -0.555984 | -0.41207 | -8.1623 | 9.42E-14 | 1.78E-12 | 20.57418 |
| YY1 | -0.739067 | -0.44834 | -8.1586 | 9.63E-14 | 1.81E-12 | 20.55294 |
| LRP10 | -0.613264 | -0.3986 | -8.08938 | 1.44E-13 | 2.65E-12 | 20.15559 |
| KLF4 | -0.522643 | -0.22484 | -8.07958 | 1.53E-13 | 2.79E-12 | 20.09943 |
| TXN | 0.6460843 | 0.34379 | 8.068543 | 1.63E-13 | 2.96E-12 | 20.03627 |
| HNRPU | -0.517157 | -0.43204 | -8.06516 | 1.66E-13 | 3.01E-12 | 20.01693 |
| CPSF1 | -0.548095 | -0.51433 | -8.05302 | 1.78E-13 | 3.22E-12 | 19.94746 |
| COG4 | -0.503692 | -0.45643 | -8.03937 | 1.93E-13 | 3.47E-12 | 19.86942 |
| RPL23AP7 | -0.68381 | -0.48115 | -8.01791 | 2.18E-13 | 3.90E-12 | 19.74681 |
| H1FX | -0.703716 | -0.23446 | -7.9926 | 2.53E-13 | 4.45E-12 | 19.60244 |
| FCGR1A | 0.6163571 | 0.408694 | 7.98509 | 2.64E-13 | 4.64E-12 | 19.55963 |
| MSRB2 | 0.6643175 | 0.621019 | 7.981203 | 2.70E-13 | 4.72E-12 | 19.53748 |
| RASGRP2 | -1.008474 | -0.67764 | -7.94771 | 3.28E-13 | 5.69E-12 | 19.34681 |
| NUDC | -0.541498 | -0.38185 | -7.94384 | 3.35E-13 | 5.81E-12 | 19.3248 |
| RAP1B | -0.800347 | -0.51873 | -7.93839 | 3.46E-13 | 5.99E-12 | 19.2938 |
| C2 | -0.545919 | -0.37522 | -7.92237 | 3.79E-13 | 6.53E-12 | 19.20279 |
| SNRPC | -0.550488 | -0.40204 | -7.90545 | 4.18E-13 | 7.15E-12 | 19.10675 |
| UBE2I | -0.591256 | -0.48312 | -7.89936 | 4.33E-13 | 7.38E-12 | 19.07218 |
| ANGPTL1 | 0.7151905 | 0.482866 | 7.899044 | 4.34E-13 | 7.39E-12 | 19.07039 |
| ANP32B | -0.621573 | -0.45586 | -7.89653 | 4.40E-13 | 7.48E-12 | 19.05611 |
| HNRPK | -0.710167 | -0.48395 | -7.89636 | 4.40E-13 | 7.48E-12 | 19.05518 |
| KRT23 | 0.6119206 | 0.226752 | 7.890586 | 4.55E-13 | 7.70E-12 | 19.02243 |
| RPL36 | -0.645504 | -0.48338 | -7.88713 | 4.65E-13 | 7.84E-12 | 19.00281 |
| FIBP | 0.5779008 | 0.122038 | 7.883076 | 4.75E-13 | 8.01E-12 | 18.97986 |
| ORMDL3 | -0.666956 | -0.38401 | -7.85864 | 5.47E-13 | 9.12E-12 | 18.84147 |
| MATR3 | -0.63383 | -0.37016 | -7.85584 | 5.56E-13 | 9.26E-12 | 18.82561 |
| HSD11B1 | 0.5471984 | 0.132803 | 7.827949 | 6.53E-13 | 1.08E-11 | 18.6679 |
| GAL3ST1 | 0.5198571 | -0.25115 | 7.826537 | 6.58E-13 | 1.08E-11 | 18.65992 |
| CSNK2B | -0.506065 | -0.33968 | -7.82517 | 6.63E-13 | 1.09E-11 | 18.6522 |
| CARD11 | -0.76844 | -0.40885 | -7.82501 | 6.64E-13 | 1.09E-11 | 18.6513 |
| ALAS2 | 0.9478472 | 0.493949 | 7.798542 | 7.73E-13 | 1.26E-11 | 18.50188 |
| DYNLT1 | 0.6197153 | 0.268312 | 7.78392 | 8.40E-13 | 1.37E-11 | 18.41944 |
| RBP7 | 0.6568532 | 0.253694 | 7.780521 | 8.57E-13 | 1.39E-11 | 18.40028 |
| ZDHHC3 | 0.5458175 | 0.162484 | 7.774914 | 8.85E-13 | 1.43E-11 | 18.36869 |
| DIABLO | -0.736873 | -0.43522 | -7.75225 | 1.01E-12 | 1.62E-11 | 18.24109 |
| BRD2 | -0.683286 | -0.61344 | -7.74311 | 1.06E-12 | 1.69E-11 | 18.18968 |
| ACTN1 | -0.682181 | -0.5451 | -7.71951 | 1.21E-12 | 1.92E-11 | 18.0571 |
| AIFM1 | -0.631345 | -0.34172 | -7.70993 | 1.28E-12 | 2.02E-11 | 18.00331 |
| TGFBI | -0.710171 | -0.54484 | -7.6945 | 1.40E-12 | 2.21E-11 | 17.91676 |
| LGALS3 | 0.541002 | 0.370382 | 7.679887 | 1.52E-12 | 2.39E-11 | 17.83487 |
| CLSPN | 0.5061667 | 0.16242 | 7.674948 | 1.57E-12 | 2.45E-11 | 17.8072 |
| LYN | -0.640435 | -0.1842 | -7.67432 | 1.57E-12 | 2.46E-11 | 17.80367 |
| SSR2 | -0.542579 | -0.18484 | -7.66018 | 1.70E-12 | 2.65E-11 | 17.72454 |
| GSDMD | -0.54144 | -0.45892 | -7.65854 | 1.72E-12 | 2.68E-11 | 17.71536 |
| CCDC11 | -0.53403 | -0.26363 | -7.62195 | 2.12E-12 | 3.26E-11 | 17.51087 |
| CDC16 | -0.770839 | -0.4979 | -7.61882 | 2.15E-12 | 3.31E-11 | 17.49341 |
| IFITM1 | 0.633244 | 0.178408 | 7.614252 | 2.21E-12 | 3.39E-11 | 17.4679 |
| HBD | 0.571873 | -0.22248 | 7.612568 | 2.23E-12 | 3.42E-11 | 17.4585 |
| ELF1 | -0.566446 | -0.50809 | -7.60893 | 2.28E-12 | 3.47E-11 | 17.43819 |
| C17orf28 | 0.518375 | -0.1942 | 7.597553 | 2.43E-12 | 3.70E-11 | 17.37477 |
| BTN3A3 | -0.688187 | -0.48471 | -7.59595 | 2.45E-12 | 3.73E-11 | 17.36582 |
| MAT2A | -0.696512 | -0.66554 | -7.57092 | 2.83E-12 | 4.26E-11 | 17.22644 |
| GDI2 | -1.241935 | -1.0993 | -7.56905 | 2.86E-12 | 4.30E-11 | 17.216 |
| CCDC54 | 0.7076964 | 0.538854 | 7.556433 | 3.07E-12 | 4.61E-11 | 17.14584 |
| PIK3C2G | -0.509778 | -0.37389 | -7.54287 | 3.31E-12 | 4.94E-11 | 17.07046 |
| HMOX2 | -0.595476 | -0.56146 | -7.51899 | 3.79E-12 | 5.61E-11 | 16.93787 |
| C6orf62 | -0.618062 | -0.62669 | -7.51793 | 3.81E-12 | 5.64E-11 | 16.93199 |
| DSTN | -0.780637 | -0.40955 | -7.51778 | 3.81E-12 | 5.64E-11 | 16.93121 |
| TPST1 | 0.6190437 | 0.647389 | 7.513364 | 3.91E-12 | 5.78E-11 | 16.90669 |
| RPL13 | -0.60104 | -0.60166 | -7.50818 | 4.03E-12 | 5.92E-11 | 16.87797 |
| BZRPL1 | 0.5097063 | -0.25217 | 7.489426 | 4.48E-12 | 6.54E-11 | 16.77404 |
| RPLP1 | -0.646742 | -0.44159 | -7.43374 | 6.12E-12 | 8.76E-11 | 16.46627 |
| SSPN | -0.509073 | -0.12561 | -7.43166 | 6.19E-12 | 8.85E-11 | 16.4548 |
| PPP6C | -0.609284 | -0.2142 | -7.42206 | 6.54E-12 | 9.33E-11 | 16.40185 |
| ANXA3 | 0.9910972 | 0.812803 | 7.418998 | 6.65E-12 | 9.47E-11 | 16.38497 |
| CEP27 | 0.5948373 | 0.457898 | 7.412938 | 6.88E-12 | 9.77E-11 | 16.35158 |
| ALOX5AP | 0.5059325 | 0.139809 | 7.40449 | 7.21E-12 | 1.02E-10 | 16.30504 |
| EPB49 | -0.607724 | -0.33376 | -7.37783 | 8.37E-12 | 1.18E-10 | 16.15834 |
| TNFRSF14 | -0.602294 | -0.43344 | -7.37567 | 8.48E-12 | 1.19E-10 | 16.14651 |
| SETD5 | -0.500419 | -0.39076 | -7.36901 | 8.80E-12 | 1.24E-10 | 16.10987 |
| ATP5G3 | -0.601097 | -0.51459 | -7.36154 | 9.17E-12 | 1.29E-10 | 16.06888 |
| SDHD | -0.502921 | -0.34299 | -7.35518 | 9.51E-12 | 1.33E-10 | 16.03395 |
| HSP90AA1 | -0.73497 | -0.38764 | -7.3373 | 1.05E-11 | 1.46E-10 | 15.93588 |
| NPM1 | -0.529136 | -0.42823 | -7.27212 | 1.51E-11 | 2.05E-10 | 15.5793 |
| PRPSAP2 | -0.537012 | -0.35376 | -7.27197 | 1.51E-11 | 2.05E-10 | 15.57851 |
| TNFAIP8 | -0.974099 | -0.79312 | -7.27172 | 1.51E-11 | 2.05E-10 | 15.57712 |
| NIPSNAP1 | -0.516659 | -0.30013 | -7.26796 | 1.55E-11 | 2.09E-10 | 15.55663 |
| PARP2 | -0.586571 | -0.34045 | -7.25632 | 1.65E-11 | 2.22E-10 | 15.49314 |
| C19orf10 | -0.603802 | -0.23229 | -7.23121 | 1.89E-11 | 2.53E-10 | 15.35633 |
| CCT6A | -0.619465 | -0.53213 | -7.23086 | 1.90E-11 | 2.53E-10 | 15.35446 |
| LRRC31 | 0.5657996 | 0.612739 | 7.223276 | 1.98E-11 | 2.63E-10 | 15.31318 |
| LMBRD2 | 0.609627 | 0.811338 | 7.202789 | 2.22E-11 | 2.93E-10 | 15.20181 |
| SUMO4 | -0.626603 | -0.40611 | -7.19859 | 2.27E-11 | 2.99E-10 | 15.17902 |
| ACADM | -0.800567 | -0.64822 | -7.1892 | 2.39E-11 | 3.13E-10 | 15.12801 |
| VPREB3 | -0.500804 | -0.39726 | -7.17587 | 2.57E-11 | 3.36E-10 | 15.05571 |
| NP | -0.571562 | -0.41752 | -7.1413 | 3.11E-11 | 4.01E-10 | 14.86851 |
| DEFA3 | 1.6316766 | 0.269554 | 7.140328 | 3.13E-11 | 4.03E-10 | 14.86328 |
| RHOC | -0.731145 | -0.54803 | -7.13666 | 3.19E-11 | 4.10E-10 | 14.84342 |
| BCKDHA | -0.676498 | -0.33815 | -7.12843 | 3.34E-11 | 4.29E-10 | 14.79898 |
| ARG1 | 0.93875 | 0.629682 | 7.127105 | 3.37E-11 | 4.31E-10 | 14.7918 |
| PHOX2A | -0.522613 | -0.13815 | -7.09641 | 3.99E-11 | 5.05E-10 | 14.62616 |
| RPS3 | -0.661996 | -0.59936 | -7.09454 | 4.03E-11 | 5.09E-10 | 14.61607 |
| CNBP | -0.616188 | -0.2958 | -7.09277 | 4.07E-11 | 5.14E-10 | 14.60654 |
| C11orf24 | -0.631825 | -0.39962 | -7.09144 | 4.10E-11 | 5.17E-10 | 14.59937 |
| IL8RB | -0.690722 | -0.56452 | -7.08275 | 4.30E-11 | 5.41E-10 | 14.55254 |
| BHLHB4 | -0.524817 | -0.4335 | -7.06729 | 4.68E-11 | 5.84E-10 | 14.46934 |
| IGF2R | -0.663804 | -0.60554 | -7.04883 | 5.18E-11 | 6.43E-10 | 14.37008 |
| GZMH | -0.992024 | -0.88102 | -7.03421 | 5.61E-11 | 6.91E-10 | 14.2916 |
| RNF157 | 0.6293889 | -0.6079 | 7.031213 | 5.70E-11 | 7.00E-10 | 14.27553 |
| CFD | -0.716234 | -0.48739 | -7.02153 | 6.01E-11 | 7.35E-10 | 14.22359 |
| FGFR2 | 0.5713224 | 0.45879 | 7.016985 | 6.16E-11 | 7.52E-10 | 14.19923 |
| KLHL13 | 0.5180734 | 0.188025 | 7.013897 | 6.26E-11 | 7.64E-10 | 14.18269 |
| C7orf42 | -0.636857 | -0.42032 | -6.97318 | 7.82E-11 | 9.38E-10 | 13.96488 |
| PWP1 | -0.533224 | -0.45261 | -6.96284 | 8.27E-11 | 9.89E-10 | 13.90964 |
| TNFAIP6 | 0.7093571 | 0.522038 | 6.96189 | 8.32E-11 | 9.91E-10 | 13.90458 |
| PPP1CC | -0.674157 | -0.41204 | -6.95913 | 8.44E-11 | 1.00E-09 | 13.88983 |
| MCM5 | -0.600688 | -0.46274 | -6.931 | 9.84E-11 | 1.16E-09 | 13.73991 |
| NHP2L1 | -0.550026 | -0.36815 | -6.91575 | 1.07E-10 | 1.26E-09 | 13.65876 |
| SAT1 | 0.5265188 | 0.355828 | 6.910695 | 1.10E-10 | 1.29E-09 | 13.63188 |
| ZFP91 | -0.510393 | -0.3721 | -6.88952 | 1.23E-10 | 1.43E-09 | 13.51941 |
| PNRC1 | -0.661248 | -0.49127 | -6.8862 | 1.25E-10 | 1.46E-09 | 13.50181 |
| RPN1 | -0.564139 | -0.43955 | -6.86451 | 1.41E-10 | 1.63E-09 | 13.38681 |
| NMI | 0.6567857 | 0.508535 | 6.860879 | 1.44E-10 | 1.66E-09 | 13.36757 |
| ZNF567 | 0.5116925 | 0.19414 | 6.850557 | 1.52E-10 | 1.75E-09 | 13.31293 |
| CD2BP2 | -0.676692 | -0.34185 | -6.82794 | 1.72E-10 | 1.95E-09 | 13.19338 |
| MRPS7 | 0.5207262 | 0.24414 | 6.824296 | 1.75E-10 | 1.99E-09 | 13.17413 |
| C13orf3 | 0.6238155 | 0.219682 | 6.811507 | 1.88E-10 | 2.12E-09 | 13.10663 |
| EIF4A2 | -0.523461 | -0.38376 | -6.80796 | 1.91E-10 | 2.16E-09 | 13.08792 |
| CDK4 | -0.537758 | -0.3614 | -6.79084 | 2.10E-10 | 2.34E-09 | 12.9977 |
| STMN1 | -0.565335 | -0.17841 | -6.78616 | 2.15E-10 | 2.39E-09 | 12.97309 |
| ACSL5 | -0.586131 | -0.30146 | -6.77858 | 2.24E-10 | 2.48E-09 | 12.93319 |
| MRRF | 0.7324444 | 0.450064 | 6.775947 | 2.27E-10 | 2.51E-09 | 12.91933 |
| TRPS1 | 0.6094048 | 0.221401 | 6.772278 | 2.32E-10 | 2.56E-09 | 12.90004 |
| ITGB1 | -0.520905 | -0.48427 | -6.74459 | 2.69E-10 | 2.93E-09 | 12.75462 |
| JMJD1A | -0.699879 | -0.41217 | -6.73472 | 2.83E-10 | 3.08E-09 | 12.70286 |
| CCT3 | -0.504782 | -0.43121 | -6.70725 | 3.28E-10 | 3.54E-09 | 12.55903 |
| KLRK1 | -0.559528 | -0.40338 | -6.66181 | 4.18E-10 | 4.47E-09 | 12.32184 |
| TOB1 | -0.65694 | -0.39331 | -6.63363 | 4.86E-10 | 5.16E-09 | 12.17517 |
| RABGGTB | -0.570208 | -0.49344 | -6.63274 | 4.88E-10 | 5.17E-09 | 12.17053 |
| NUBP1 | -0.536417 | -0.38 | -6.62536 | 5.08E-10 | 5.36E-09 | 12.1322 |
| SLC20A1 | -0.51251 | -0.55783 | -6.58322 | 6.35E-10 | 6.58E-09 | 11.91373 |
| HPN | -0.520661 | -0.33943 | -6.56798 | 6.88E-10 | 7.11E-09 | 11.8349 |
| LY96 | 0.8280377 | 0.581146 | 6.55695 | 7.29E-10 | 7.49E-09 | 11.77791 |
| REG4 | 0.5117937 | 0.696879 | 6.52121 | 8.80E-10 | 8.93E-09 | 11.59366 |
| PTPRC | -0.647228 | -0.68083 | -6.51532 | 9.08E-10 | 9.20E-09 | 11.56337 |
| ERRFI1 | 0.5591111 | 0.149745 | 6.505551 | 9.56E-10 | 9.66E-09 | 11.51311 |
| NAP1L1 | -0.561822 | -0.39401 | -6.50216 | 9.73E-10 | 9.81E-09 | 11.49568 |
| MORF4L2 | -0.555143 | -0.35803 | -6.502 | 9.74E-10 | 9.81E-09 | 11.49483 |
| ANXA2 | -0.602854 | -0.45439 | -6.50145 | 9.76E-10 | 9.83E-09 | 11.49202 |
| KNG1 | 0.5497421 | 0.394395 | 6.499645 | 9.86E-10 | 9.91E-09 | 11.48276 |
| CCDC14 | 0.570879 | 0.15414 | 6.469141 | 1.16E-09 | 1.15E-08 | 11.32626 |
| HSPD1 | -0.570752 | -0.45561 | -6.46555 | 1.18E-09 | 1.17E-08 | 11.30785 |
| YTHDC1 | 0.5691865 | 0.363822 | 6.439724 | 1.35E-09 | 1.33E-08 | 11.17573 |
| FPR1 | 0.5653651 | 0.105096 | 6.406994 | 1.60E-09 | 1.56E-08 | 11.00873 |
| EFTUD2 | -0.523839 | -0.45369 | -6.40065 | 1.65E-09 | 1.61E-08 | 10.97643 |
| FBXO8 | -0.535113 | -0.43707 | -6.40007 | 1.66E-09 | 1.61E-08 | 10.97348 |
| ADI1 | -0.686143 | -0.54548 | -6.39266 | 1.72E-09 | 1.66E-08 | 10.93575 |
| PLEK2 | 0.8145298 | 0.625732 | 6.377533 | 1.87E-09 | 1.79E-08 | 10.85884 |
| AP4B1 | -0.580073 | -0.43025 | -6.36537 | 1.99E-09 | 1.89E-08 | 10.79708 |
| C1orf213 | 0.529748 | 0.301465 | 6.333007 | 2.35E-09 | 2.21E-08 | 10.63307 |
| GBP2 | 0.5227063 | 0.179108 | 6.328749 | 2.40E-09 | 2.25E-08 | 10.61153 |
| GRIN2C | 0.5340675 | 0.342102 | 6.316942 | 2.55E-09 | 2.38E-08 | 10.55185 |
| ABCA12 | 0.7561369 | 0.434076 | 6.307093 | 2.69E-09 | 2.49E-08 | 10.50211 |
| ZNF544 | -0.562895 | -0.39 | -6.30179 | 2.76E-09 | 2.55E-08 | 10.47537 |
| KRT2 | 0.5023433 | 0.363248 | 6.289655 | 2.94E-09 | 2.71E-08 | 10.41417 |
| PEX7 | -0.543722 | -0.49166 | -6.2749 | 3.17E-09 | 2.91E-08 | 10.33988 |
| HP1BP3 | -0.559599 | -0.35943 | -6.24269 | 3.74E-09 | 3.41E-08 | 10.17802 |
| CAMP | 0.5541409 | 0.202866 | 6.240006 | 3.80E-09 | 3.46E-08 | 10.16457 |
| ZNF138 | 0.7360853 | 0.348217 | 6.233047 | 3.93E-09 | 3.57E-08 | 10.12968 |
| LDHB | -0.679984 | -0.41153 | -6.22243 | 4.15E-09 | 3.75E-08 | 10.0765 |
| PRKCSH | -0.645704 | -0.38841 | -6.20156 | 4.62E-09 | 4.13E-08 | 9.972122 |
| F13A1 | -0.535421 | -0.46618 | -6.1967 | 4.74E-09 | 4.21E-08 | 9.947842 |
| STK24 | -0.562385 | -0.28541 | -6.19347 | 4.82E-09 | 4.28E-08 | 9.931733 |
| TLR2 | 0.5551012 | 0.168662 | 6.182406 | 5.10E-09 | 4.50E-08 | 9.876503 |
| ATP6V1B2 | -0.550341 | -0.53904 | -6.16015 | 5.71E-09 | 4.99E-08 | 9.765634 |
| CYB561D2 | -0.527248 | -0.44968 | -6.15896 | 5.75E-09 | 5.02E-08 | 9.759736 |
| SLC4A7 | 0.5231792 | 0.222038 | 6.154699 | 5.87E-09 | 5.11E-08 | 9.738517 |
| STAT4 | -0.585933 | -0.54688 | -6.15155 | 5.97E-09 | 5.19E-08 | 9.722872 |
| RNF213 | -0.660629 | -0.56217 | -6.13617 | 6.45E-09 | 5.58E-08 | 9.646471 |
| AREG | 0.6623988 | 0.459873 | 6.129796 | 6.67E-09 | 5.76E-08 | 9.61482 |
| AKT2 | 0.7275159 | 0.582548 | 6.115564 | 7.17E-09 | 6.16E-08 | 9.544267 |
| OFD1 | -0.59301 | -0.51726 | -6.09729 | 7.86E-09 | 6.71E-08 | 9.453827 |
| EIF4A3 | -0.503938 | -0.35172 | -6.09305 | 8.03E-09 | 6.84E-08 | 9.432869 |
| COQ10B | -0.523355 | -0.3549 | -6.08196 | 8.50E-09 | 7.21E-08 | 9.378068 |
| FREM1 | 0.5032629 | 0.364904 | 6.055128 | 9.73E-09 | 8.21E-08 | 9.245792 |
| AP1S2 | -0.618753 | -0.25707 | -6.03652 | 1.07E-08 | 8.97E-08 | 9.154244 |
| IQGAP1 | -0.916942 | -0.90924 | -6.03529 | 1.08E-08 | 9.02E-08 | 9.148207 |
| HIP1 | 0.6977837 | 0.461338 | 6.010684 | 1.22E-08 | 1.01E-07 | 9.02748 |
| ELP2 | -0.591012 | -0.49828 | -5.99793 | 1.30E-08 | 1.07E-07 | 8.965027 |
| C14orf65 | -0.546425 | -0.25892 | -5.99462 | 1.32E-08 | 1.09E-07 | 8.948824 |
| THOC1 | -0.567794 | -0.49083 | -5.9876 | 1.37E-08 | 1.13E-07 | 8.91448 |
| PRMT1 | -0.748333 | -0.35051 | -5.98646 | 1.37E-08 | 1.13E-07 | 8.908922 |
| MPG | -0.501345 | -0.33898 | -5.97628 | 1.45E-08 | 1.18E-07 | 8.859194 |
| RAB7L1 | -0.513423 | -0.37293 | -5.96443 | 1.54E-08 | 1.25E-07 | 8.801327 |
| PROK2 | -0.873069 | -0.76038 | -5.93755 | 1.76E-08 | 1.42E-07 | 8.670433 |
| SLC22A1 | 0.5078631 | 0.263631 | 5.935909 | 1.77E-08 | 1.43E-07 | 8.662461 |
| CCT4 | -0.564669 | -0.5086 | -5.93347 | 1.79E-08 | 1.45E-07 | 8.65062 |
| IDH1 | -0.596298 | -0.40605 | -5.9176 | 1.94E-08 | 1.56E-07 | 8.573512 |
| SEMA6D | 0.5193681 | 0.333949 | 5.896239 | 2.16E-08 | 1.72E-07 | 8.469989 |
| PRDX1 | -0.553306 | -0.37516 | -5.88309 | 2.30E-08 | 1.82E-07 | 8.406389 |
| CCDC109B | -0.689032 | -0.46465 | -5.87074 | 2.45E-08 | 1.93E-07 | 8.346699 |
| CLINT1 | -0.501083 | -0.18013 | -5.8684 | 2.48E-08 | 1.95E-07 | 8.335419 |
| AASS | 0.6113353 | 0.485223 | 5.845824 | 2.77E-08 | 2.17E-07 | 8.226579 |
| HESX1 | 0.5925774 | 0.460064 | 5.84301 | 2.81E-08 | 2.19E-07 | 8.213035 |
| MND1 | 0.5850258 | 0.483121 | 5.83998 | 2.85E-08 | 2.22E-07 | 8.198453 |
| ASXL3 | 0.5207391 | 0.412038 | 5.832517 | 2.96E-08 | 2.30E-07 | 8.162556 |
| PF4 | 0.5706806 | 0.419554 | 5.828886 | 3.01E-08 | 2.34E-07 | 8.145102 |
| GNGT1 | 0.6537718 | 0.511274 | 5.80437 | 3.40E-08 | 2.61E-07 | 8.027442 |
| GUSB | -0.510978 | -0.41363 | -5.77574 | 3.91E-08 | 2.97E-07 | 7.890462 |
| RPL13A | -0.542718 | -0.30605 | -5.77537 | 3.92E-08 | 2.97E-07 | 7.888681 |
| MAGEL2 | 0.6010357 | 0.520764 | 5.730316 | 4.89E-08 | 3.66E-07 | 7.673999 |
| ZDHHC2 | 0.6183948 | 0.361592 | 5.725361 | 5.01E-08 | 3.74E-07 | 7.650456 |
| ZNF624 | 0.516631 | 0.293885 | 5.713025 | 5.32E-08 | 3.95E-07 | 7.5919 |
| AZIN1 | -0.561302 | -0.29197 | -5.68392 | 6.13E-08 | 4.51E-07 | 7.454101 |
| MIB1 | 0.5412798 | 0.392803 | 5.668188 | 6.61E-08 | 4.84E-07 | 7.379776 |
| RANBP5 | -0.501681 | -0.13433 | -5.66224 | 6.81E-08 | 4.98E-07 | 7.351722 |
| SIAH2 | 0.5074762 | 0.596688 | 5.657249 | 6.97E-08 | 5.10E-07 | 7.328188 |
| TNFSF10 | 0.589879 | 0.403694 | 5.654777 | 7.06E-08 | 5.15E-07 | 7.316542 |
| SMNDC1 | -0.618956 | -0.44777 | -5.64527 | 7.39E-08 | 5.37E-07 | 7.271778 |
| PCDHB8 | 0.5500972 | 0.394204 | 5.638233 | 7.65E-08 | 5.55E-07 | 7.238674 |
| GKN1 | 0.6559841 | 0.318408 | 5.636562 | 7.71E-08 | 5.59E-07 | 7.230819 |
| CTSH | -0.68978 | -0.35274 | -5.62865 | 8.01E-08 | 5.79E-07 | 7.193647 |
| NQO2 | 0.5330099 | 0.184459 | 5.608534 | 8.82E-08 | 6.33E-07 | 7.099268 |
| CUL1 | -0.515837 | -0.39554 | -5.60252 | 9.08E-08 | 6.49E-07 | 7.071118 |
| PPP2CA | -0.664006 | -0.54102 | -5.60045 | 9.18E-08 | 6.55E-07 | 7.061401 |
| ACP5 | -0.576935 | -0.3249 | -5.59891 | 9.24E-08 | 6.58E-07 | 7.054207 |
| USP47 | -0.573341 | -0.37745 | -5.59223 | 9.55E-08 | 6.79E-07 | 7.022953 |
| EBI2 | -0.710288 | -0.28115 | -5.52372 | 1.33E-07 | 9.20E-07 | 6.703904 |
| CLC | -0.592929 | -0.57898 | -5.49953 | 1.49E-07 | 1.02E-06 | 6.591846 |
| MMP9 | 0.6971964 | 0.429363 | 5.493564 | 1.53E-07 | 1.05E-06 | 6.564281 |
| C1QTNF7 | 0.6819385 | 0.480701 | 5.487303 | 1.58E-07 | 1.08E-06 | 6.53536 |
| CDA | 0.5000516 | 0.48828 | 5.486295 | 1.58E-07 | 1.08E-06 | 6.530709 |
| PPBP | 0.5572143 | 0.543503 | 5.454613 | 1.84E-07 | 1.24E-06 | 6.384736 |
| SLC7A1 | 0.5757619 | 0.471401 | 5.394647 | 2.45E-07 | 1.61E-06 | 6.110032 |
| ZIM2 | 0.5524147 | 0.384968 | 5.379102 | 2.63E-07 | 1.73E-06 | 6.03916 |
| PTPN12 | -0.52298 | -0.29064 | -5.37573 | 2.67E-07 | 1.75E-06 | 6.02382 |
| LGI1 | 0.5904762 | 0.327898 | 5.354665 | 2.95E-07 | 1.92E-06 | 5.928035 |
| PPHLN1 | -0.576371 | -0.18828 | -5.32662 | 3.36E-07 | 2.16E-06 | 5.800947 |
| ADH5 | -0.505145 | -0.3528 | -5.32341 | 3.41E-07 | 2.19E-06 | 5.786422 |
| REL | -0.587244 | -0.33159 | -5.31983 | 3.47E-07 | 2.23E-06 | 5.770242 |
| BMPR1B | 0.598248 | 0.424331 | 5.314089 | 3.57E-07 | 2.28E-06 | 5.7443 |
| RHCE | 0.5399841 | 0.481656 | 5.305468 | 3.71E-07 | 2.36E-06 | 5.705387 |
| ALDH1A1 | -0.50202 | -0.45057 | -5.29849 | 3.83E-07 | 2.43E-06 | 5.67393 |
| HABP2 | 0.542377 | 0.439363 | 5.293437 | 3.93E-07 | 2.48E-06 | 5.651153 |
| GZMB | -0.673599 | -0.65675 | -5.29328 | 3.93E-07 | 2.48E-06 | 5.650438 |
| RCBTB1 | 0.6947242 | 0.513822 | 5.289675 | 4.00E-07 | 2.52E-06 | 5.634216 |
| LASP1 | 0.5479663 | 0.35535 | 5.264979 | 4.48E-07 | 2.80E-06 | 5.523223 |
| PBEF1 | 0.53075 | 0.126624 | 5.258608 | 4.61E-07 | 2.88E-06 | 5.494644 |
| INTS10 | -0.558669 | -0.45032 | -5.23699 | 5.10E-07 | 3.16E-06 | 5.397863 |
| ROBO1 | 0.6435317 | 0.590191 | 5.225897 | 5.37E-07 | 3.31E-06 | 5.348315 |
| SAMHD1 | -0.559855 | -0.39694 | -5.17915 | 6.66E-07 | 4.03E-06 | 5.140335 |
| ZNF510 | 0.5963968 | 0.468344 | 5.16573 | 7.08E-07 | 4.26E-06 | 5.080851 |
| DOCK3 | 0.5710694 | 0.406943 | 5.13866 | 8.01E-07 | 4.77E-06 | 4.961236 |
| CLK1 | -0.54622 | -0.33299 | -5.13571 | 8.12E-07 | 4.83E-06 | 4.948212 |
| SEC24C | -0.644052 | -0.49701 | -5.13432 | 8.17E-07 | 4.86E-06 | 4.942085 |
| SPC25 | 0.5785575 | 0.429618 | 5.127535 | 8.43E-07 | 4.99E-06 | 4.912207 |
| GTF2IP1 | -0.501437 | -0.18949 | -5.12349 | 8.58E-07 | 5.07E-06 | 4.894405 |
| RRM1 | -0.505371 | -0.29363 | -5.11964 | 8.73E-07 | 5.15E-06 | 4.877471 |
| ID3 | -0.506889 | -0.33471 | -5.10611 | 9.29E-07 | 5.44E-06 | 4.818012 |
| RANBP1 | -0.540621 | -0.38478 | -5.10489 | 9.34E-07 | 5.47E-06 | 4.812629 |
| DDX5 | -0.534363 | -0.46854 | -5.07917 | 1.05E-06 | 6.08E-06 | 4.699924 |
| SKIL | 0.5353165 | 0.462771 | 5.061946 | 1.13E-06 | 6.54E-06 | 4.624694 |
| CCDC56 | -0.504254 | -0.34261 | -5.03575 | 1.28E-06 | 7.29E-06 | 4.510607 |
| C1orf54 | 0.6514504 | 0.569618 | 4.989482 | 1.57E-06 | 8.85E-06 | 4.31016 |
| GPR89A | -0.545192 | -0.36204 | -4.98938 | 1.57E-06 | 8.85E-06 | 4.309701 |
| SF3B1 | -0.577675 | -0.48121 | -4.97786 | 1.66E-06 | 9.25E-06 | 4.260012 |
| CTSC | -0.510179 | -0.46395 | -4.9619 | 1.78E-06 | 9.88E-06 | 4.191294 |
| KIAA1586 | 0.6837202 | 0.331083 | 4.937722 | 1.98E-06 | 1.09E-05 | 4.087511 |
| GLOD4 | -0.605012 | -0.47427 | -4.92955 | 2.05E-06 | 1.13E-05 | 4.052537 |
| BIRC3 | -0.566466 | -0.42255 | -4.9279 | 2.07E-06 | 1.14E-05 | 4.045448 |
| IFIT3 | 0.5798413 | 0.298089 | 4.90835 | 2.25E-06 | 1.23E-05 | 3.961927 |
| GSTCD | 0.5058056 | 0.381274 | 4.893724 | 2.41E-06 | 1.30E-05 | 3.899595 |
| SAMM50 | -0.569877 | -0.45898 | -4.88842 | 2.46E-06 | 1.33E-05 | 3.877016 |
| MRPS24 | -0.535728 | -0.37529 | -4.88208 | 2.53E-06 | 1.37E-05 | 3.850051 |
| FBN1 | 0.503748 | 0.388917 | 4.879512 | 2.56E-06 | 1.38E-05 | 3.839159 |
| TEX15 | 0.5308631 | 0.392038 | 4.864886 | 2.73E-06 | 1.47E-05 | 3.7771 |
| C20orf86 | 0.5355714 | 0.350064 | 4.840258 | 3.04E-06 | 1.62E-05 | 3.67291 |
| OR2J2 | 0.5383175 | -0.04287 | 4.824278 | 3.26E-06 | 1.72E-05 | 3.605514 |
| OR6C3 | 0.5144722 | 0.43879 | 4.803918 | 3.57E-06 | 1.87E-05 | 3.519889 |
| MAK | 0.5296627 | 0.462293 | 4.800523 | 3.62E-06 | 1.89E-05 | 3.505636 |
| SOX18 | -0.509405 | 0.019936 | -4.78699 | 3.84E-06 | 2.00E-05 | 3.44891 |
| IFI44L | 0.6051766 | 0.281274 | 4.772018 | 4.10E-06 | 2.12E-05 | 3.386277 |
| MMP12 | 0.5899821 | 0.466879 | 4.732248 | 4.87E-06 | 2.48E-05 | 3.220628 |
| MYOM2 | -0.508671 | -0.5051 | -4.72601 | 5.00E-06 | 2.54E-05 | 3.194737 |
| FAM29A | 0.506621 | 0.284522 | 4.71192 | 5.32E-06 | 2.68E-05 | 3.136368 |
| STK33 | 0.5050198 | 0.294713 | 4.707718 | 5.41E-06 | 2.72E-05 | 3.118983 |
| OCIAD1 | -0.558147 | -0.23306 | -4.70618 | 5.45E-06 | 2.74E-05 | 3.112642 |
| PAGE4 | 0.5395099 | 0.385096 | 4.701465 | 5.56E-06 | 2.79E-05 | 3.093134 |
| PRSS35 | 0.5515337 | 0.367006 | 4.69709 | 5.67E-06 | 2.84E-05 | 3.075063 |
| OR51B6 | 0.5053988 | 0.199873 | 4.695317 | 5.71E-06 | 2.85E-05 | 3.067747 |
| HS3ST1 | 0.5055853 | 0.231783 | 4.651021 | 6.90E-06 | 3.38E-05 | 2.885566 |
| FSTL5 | 0.5339206 | 0.439108 | 4.649941 | 6.93E-06 | 3.40E-05 | 2.881141 |
| DAP | -0.569232 | -0.51408 | -4.63393 | 7.42E-06 | 3.61E-05 | 2.815607 |
| ZNF623 | 0.5724762 | 0.513057 | 4.630731 | 7.53E-06 | 3.65E-05 | 2.802553 |
| SLC16A7 | 0.5850198 | 0.411783 | 4.602278 | 8.49E-06 | 4.08E-05 | 2.686613 |
| PPP1R14C | 0.5137202 | 0.369809 | 4.600779 | 8.55E-06 | 4.10E-05 | 2.680519 |
| SULT2A1 | 0.7423829 | 0.503376 | 4.599441 | 8.59E-06 | 4.12E-05 | 2.675084 |
| DPYS | 0.5118512 | 0.369809 | 4.586108 | 9.09E-06 | 4.33E-05 | 2.620969 |
| SLC25A2 | 0.5087421 | 0.331146 | 4.58108 | 9.29E-06 | 4.41E-05 | 2.600592 |
| ZNF665 | 0.6048393 | 0.525478 | 4.580892 | 9.30E-06 | 4.41E-05 | 2.599829 |
| CCL17 | 0.5004623 | 0.240573 | 4.569857 | 9.74E-06 | 4.60E-05 | 2.555171 |
| OSBPL6 | 0.5197619 | 0.397452 | 4.553037 | 1.05E-05 | 4.91E-05 | 2.487258 |
| MTTP | 0.5352063 | 0.358025 | 4.545072 | 1.08E-05 | 5.07E-05 | 2.455164 |
| PICALM | -0.574105 | -0.42911 | -4.54275 | 1.09E-05 | 5.11E-05 | 2.445803 |
| C1QTNF6 | 0.5206171 | 0.379618 | 4.54139 | 1.10E-05 | 5.14E-05 | 2.440346 |
| PDC | 0.507627 | 0.450573 | 4.535758 | 1.12E-05 | 5.25E-05 | 2.41769 |
| MRPS30 | -0.517607 | -0.25325 | -4.50259 | 1.29E-05 | 5.94E-05 | 2.284748 |
| NTS | 0.5570357 | 0.333376 | 4.482498 | 1.40E-05 | 6.41E-05 | 2.204553 |
| TMEM132C | 0.6007698 | 0.321019 | 4.480318 | 1.42E-05 | 6.46E-05 | 2.195871 |
| PROM1 | 0.5689623 | 0.373439 | 4.452263 | 1.59E-05 | 7.17E-05 | 2.084424 |
| MGAT1 | -0.570659 | -0.39465 | -4.4512 | 1.60E-05 | 7.20E-05 | 2.08023 |
| PTPRM | 0.5690278 | 0.423153 | 4.353671 | 2.39E-05 | 0.000103 | 1.697108 |
| PANK3 | 0.5260893 | 0.459299 | 4.339458 | 2.53E-05 | 0.000108 | 1.641835 |
| RGS18 | -0.63451 | -0.65287 | -4.33493 | 2.58E-05 | 0.00011 | 1.624245 |
| C16orf70 | 0.5615992 | 0.478854 | 4.331897 | 2.61E-05 | 0.000112 | 1.61249 |
| DEFA4 | 0.5694663 | 0.296688 | 4.262928 | 3.45E-05 | 0.000144 | 1.346668 |
| PCDHGA5 | 0.5629683 | 0.496497 | 4.247407 | 3.67E-05 | 0.000152 | 1.287314 |
| KHDRBS3 | 0.535129 | 0.290637 | 4.239011 | 3.79E-05 | 0.000157 | 1.255277 |
| ACCN5 | 0.5304821 | 0.306433 | 4.233118 | 3.88E-05 | 0.00016 | 1.232822 |
| ZNF702 | 0.5358016 | 0.341783 | 4.214489 | 4.18E-05 | 0.000171 | 1.162001 |
| GABRB3 | 0.8593115 | 0.62879 | 4.211485 | 4.23E-05 | 0.000173 | 1.150604 |
| KLRB1 | -0.5187 | -0.26892 | -4.20214 | 4.39E-05 | 0.000179 | 1.115193 |
| RUNX2 | 0.5546508 | 0.397452 | 4.195215 | 4.52E-05 | 0.000183 | 1.088987 |
| PIWIL1 | 0.5392917 | 0.386051 | 4.158702 | 5.22E-05 | 0.000209 | 0.951406 |
| ZNF664 | 0.5003016 | 0.234459 | 4.148748 | 5.43E-05 | 0.000216 | 0.91407 |
| IFNA8 | 0.5883472 | 0.44949 | 4.123169 | 6.00E-05 | 0.000237 | 0.818448 |
| GRB14 | 0.5553968 | 0.455096 | 4.089286 | 6.85E-05 | 0.000267 | 0.692519 |
| HSD3B1 | 0.5530734 | 0.492994 | 4.060152 | 7.67E-05 | 0.000295 | 0.584915 |
| RAI14 | 0.5506111 | 0.549682 | 4.038572 | 8.34E-05 | 0.000318 | 0.505611 |
| SIGIRR | -0.517839 | -0.36541 | -4.02181 | 8.90E-05 | 0.000337 | 0.444263 |
| ZBTB26 | 0.5251865 | 0.236433 | 4.013974 | 9.17E-05 | 0.000347 | 0.415637 |
| C9orf24 | 0.5374742 | 0.427643 | 3.973594 | 0.000107 | 0.000397 | 0.268911 |
| HAVCR1 | 0.5067401 | 0.371274 | 3.945915 | 0.000119 | 0.000436 | 0.169036 |
| KL | 0.5451607 | 0.384904 | 3.901065 | 0.000141 | 0.000507 | 0.008422 |
| RTN4IP1 | 0.5752401 | 0.64414 | 3.89691 | 0.000143 | 0.000515 | -0.00638 |
| CALML4 | 0.638504 | 0.500382 | 3.826892 | 0.000186 | 0.000649 | -0.25387 |
| DGKE | 0.5300099 | 0.470318 | 3.577921 | 0.000459 | 0.001451 | -1.10342 |
| MAGEA11 | 0.503996 | 0.54465 | 3.396337 | 0.000863 | 0.002539 | -1.69232 |
| RAB39 | 0.5700179 | 0.450637 | 3.348645 | 0.001014 | 0.002923 | -1.8426 |
| CXCL9 | 0.5088016 | 0.394522 | 3.295015 | 0.001213 | 0.003434 | -2.0094 |
| TM4SF20 | 0.7855317 | 0.60949 | 3.251196 | 0.001403 | 0.0039 | -2.14393 |
| SLC25A31 | 0.6472024 | 0.245032 | 3.097837 | 0.002305 | 0.00603 | -2.60231 |
| NUFIP2 | 0.615248 | 0.574459 | 3.075137 | 0.002477 | 0.006428 | -2.6685 |
| DLX2 | 0.5834603 | 0.271338 | 2.819374 | 0.005423 | 0.012819 | -3.3841 |
| CFHR2 | 0.6261111 | 0.525541 | 2.77543 | 0.006174 | 0.014369 | -3.50142 |
| SPACA1 | 0.5914901 | 0.495732 | 2.773262 | 0.006213 | 0.014442 | -3.50716 |
| NCAPG | 0.6441905 | 0.468217 | 2.707183 | 0.007527 | 0.01703 | -3.68029 |
| PHF7 | 0.6189345 | 0.532866 | 2.488525 | 0.013856 | 0.028947 | -4.22589 |
| TMC5 | 0.6217381 | 0.572866 | 2.461894 | 0.014887 | 0.030772 | -4.28944 |
| DKK1 | 0.5239187 | 0.421529 | 2.428792 | 0.016263 | 0.033186 | -4.36756 |
| RALGPS1 | 0.503381 | 0.356433 | 2.410132 | 0.017087 | 0.034639 | -4.41116 |
| ZNF549 | 0.5770972 | 0.191465 | 2.407777 | 0.017194 | 0.034816 | -4.41665 |
| ARL6IP2 | 0.605375 | 0.48586 | 2.371871 | 0.018893 | 0.037722 | -4.49959 |
| ICA1L | 0.5488155 | 0.396178 | 2.251496 | 0.025722 | 0.049289 | -4.76917 |
