# Supplementary material for: Bioinformatics and System Biology Approach to Identify the Influences of COVID-19 on Rheumatoid Arthritis
Source: Front Immunol. 2022 Apr 7;13:860676. doi: 10.3389/fimmu.2022.860676 (PMC9021444; doi:10.3389/fimmu.2022.860676)
Supplement: Supplementary file 3 [file Table_2.docx]

Table S2. DEGs in GSE171110.

| Genes | logFC | AveExpr | t | P.Value | adj.P.Value | B |
| --- | --- | --- | --- | --- | --- | --- |
| HJURP | -2.79896 | 12.6112 | -11.5698 | 4.08E-16 | 3.67E-12 | 26.33202 |
| SKA3 | -3.04243 | 12.93225 | -11.5196 | 4.82E-16 | 3.67E-12 | 26.17238 |
| CDC25A | -3.45979 | 12.84824 | -11.4764 | 5.56E-16 | 3.67E-12 | 26.03465 |
| RAD54L | -2.82671 | 12.90523 | -11.4559 | 5.95E-16 | 3.67E-12 | 25.9691 |
| GTSE1 | -2.62133 | 13.00766 | -11.4498 | 6.07E-16 | 3.67E-12 | 25.94961 |
| KIF18B | -2.72157 | 12.76043 | -11.3943 | 7.30E-16 | 3.67E-12 | 25.77217 |
| SDC1 | -5.7516 | 12.30262 | -11.2857 | 1.05E-15 | 4.24E-12 | 25.42349 |
| RPL21P16 | 1.731459 | 13.03888 | 11.26483 | 1.12E-15 | 4.24E-12 | 25.3563 |
| NCAPG | -2.99499 | 12.81247 | -11.175 | 1.52E-15 | 5.09E-12 | 25.06646 |
| MCM10 | -2.95138 | 13.03011 | -11.0358 | 2.42E-15 | 7.26E-12 | 24.61515 |
| MKI67 | -2.85233 | 12.802 | -11.0099 | 2.65E-15 | 7.26E-12 | 24.53074 |
| RPS3A | 1.861239 | 13.10828 | 10.87657 | 4.15E-15 | 1.04E-11 | 24.09571 |
| RPS14 | 1.627182 | 13.15644 | 10.81525 | 5.11E-15 | 1.15E-11 | 23.89476 |
| RPS27A | 1.495316 | 13.18954 | 10.80254 | 5.34E-15 | 1.15E-11 | 23.85305 |
| KIF4A | -3.0616 | 12.74399 | -10.7732 | 5.90E-15 | 1.19E-11 | 23.75656 |
| TICRR | -2.99837 | 12.81208 | -10.6606 | 8.66E-15 | 1.63E-11 | 23.38591 |
| CDC6 | -3.02344 | 12.97333 | -10.4909 | 1.55E-14 | 2.75E-11 | 22.82373 |
| RPL21 | 1.622475 | 13.00843 | 10.4406 | 1.84E-14 | 3.09E-11 | 22.65629 |
| CLSPN | -2.5791 | 13.09021 | -10.3769 | 2.29E-14 | 3.65E-11 | 22.44388 |
| KIF14 | -2.69235 | 12.97022 | -10.3237 | 2.76E-14 | 4.00E-11 | 22.26629 |
| RPS15A | 1.432649 | 13.18815 | 10.32123 | 2.78E-14 | 4.00E-11 | 22.25786 |
| H2AC7 | -3.32094 | 13.16928 | -10.2788 | 3.22E-14 | 4.42E-11 | 22.11598 |
| ESPL1 | -3.05085 | 12.80626 | -10.2481 | 3.58E-14 | 4.70E-11 | 22.01278 |
| CDC45 | -2.72801 | 12.95875 | -10.1875 | 4.42E-14 | 5.47E-11 | 21.80925 |
| CDCA5 | -2.71016 | 12.96029 | -10.1688 | 4.72E-14 | 5.47E-11 | 21.74662 |
| CDCA2 | -2.8463 | 12.88814 | -10.1629 | 4.81E-14 | 5.47E-11 | 21.72662 |
| RPL39 | 1.53887 | 13.16134 | 10.15777 | 4.90E-14 | 5.47E-11 | 21.70937 |
| RPL34 | 1.455625 | 13.13569 | 10.14762 | 5.08E-14 | 5.47E-11 | 21.6752 |
| RPS6 | 1.578688 | 13.16111 | 10.13248 | 5.35E-14 | 5.49E-11 | 21.62421 |
| RRM2 | -2.96993 | 12.92761 | -10.1269 | 5.46E-14 | 5.49E-11 | 21.60536 |
| KIF20A | -2.9125 | 12.68537 | -10.1007 | 5.98E-14 | 5.64E-11 | 21.51699 |
| BUB1 | -2.88343 | 13.02147 | -10.1005 | 5.98E-14 | 5.64E-11 | 21.5164 |
| TPT1 | 1.325273 | 13.12776 | 10.08471 | 6.32E-14 | 5.78E-11 | 21.46313 |
| DLGAP5 | -2.53245 | 12.86167 | -10.0431 | 7.31E-14 | 6.49E-11 | 21.32241 |
| ASPM | -2.84205 | 12.86011 | -9.9875 | 8.87E-14 | 7.65E-11 | 21.13446 |
| HMMR | -2.42651 | 12.90736 | -9.95608 | 9.91E-14 | 8.31E-11 | 21.02799 |
| BUB1B | -2.60712 | 12.9344 | -9.94008 | 1.05E-13 | 8.55E-11 | 20.97372 |
| H2AC13 | -3.23583 | 12.81299 | -9.9213 | 1.12E-13 | 8.89E-11 | 20.90997 |
| E2F8 | -2.87242 | 12.81339 | -9.83637 | 1.51E-13 | 1.12E-10 | 20.62122 |
| H3C14 | -2.44453 | 13.33803 | -9.83372 | 1.52E-13 | 1.12E-10 | 20.6122 |
| H3C15 | -2.44453 | 13.33803 | -9.83372 | 1.52E-13 | 1.12E-10 | 20.6122 |
| FOXM1 | -2.58599 | 12.90127 | -9.81891 | 1.60E-13 | 1.15E-10 | 20.56173 |
| MYBL2 | -3.3795 | 12.76306 | -9.78715 | 1.79E-13 | 1.24E-10 | 20.4535 |
| SHCBP1 | -2.81904 | 13.01058 | -9.78492 | 1.81E-13 | 1.24E-10 | 20.44589 |
| H3C4 | -2.82446 | 13.24839 | -9.75298 | 2.02E-13 | 1.36E-10 | 20.33685 |
| BIRC5 | -2.85761 | 12.78921 | -9.73558 | 2.15E-13 | 1.37E-10 | 20.27741 |
| CCNA2 | -2.15567 | 12.96133 | -9.73259 | 2.17E-13 | 1.37E-10 | 20.2672 |
| UBE2C | -2.26371 | 12.96608 | -9.73096 | 2.18E-13 | 1.37E-10 | 20.26162 |
| EXO1 | -2.54798 | 13.00297 | -9.70611 | 2.38E-13 | 1.45E-10 | 20.17667 |
| ARHGAP23 | -2.07935 | 12.62801 | -9.70335 | 2.41E-13 | 1.45E-10 | 20.16722 |
| POLQ | -2.50401 | 12.98735 | -9.65371 | 2.87E-13 | 1.70E-10 | 19.9973 |
| TOMM7 | 1.317495 | 13.18714 | 9.620179 | 3.23E-13 | 1.87E-10 | 19.88233 |
| H3C8 | -2.72932 | 12.9887 | -9.57722 | 3.76E-13 | 2.14E-10 | 19.73487 |
| KLHDC7A | 2.885833 | 12.33103 | 9.51798 | 4.64E-13 | 2.59E-10 | 19.53116 |
| DIAPH3 | -2.54889 | 12.87003 | -9.49597 | 5.02E-13 | 2.73E-10 | 19.45538 |
| PLK1 | -2.50136 | 12.91927 | -9.49293 | 5.07E-13 | 2.73E-10 | 19.4449 |
| CDC20 | -3.00076 | 12.80928 | -9.48812 | 5.16E-13 | 2.73E-10 | 19.42835 |
| AC034236.1 | 1.743863 | 13.1909 | 9.482752 | 5.26E-13 | 2.74E-10 | 19.40984 |
| TPX2 | -2.39598 | 12.82 | -9.47659 | 5.37E-13 | 2.75E-10 | 19.3886 |
| DEPDC1 | -2.67467 | 12.75639 | -9.46738 | 5.55E-13 | 2.75E-10 | 19.35687 |
| DTL | -2.47603 | 13.03985 | -9.4671 | 5.56E-13 | 2.75E-10 | 19.35589 |
| CCNB2 | -2.41118 | 12.84909 | -9.45767 | 5.75E-13 | 2.80E-10 | 19.3234 |
| RPS7 | 1.278795 | 13.26405 | 9.448933 | 5.93E-13 | 2.84E-10 | 19.29325 |
| ORC1 | -2.27485 | 12.96042 | -9.42593 | 6.43E-13 | 3.03E-10 | 19.21387 |
| KIF15 | -2.28854 | 12.90911 | -9.39734 | 7.12E-13 | 3.31E-10 | 19.11512 |
| SPC24 | -2.07542 | 12.77483 | -9.39209 | 7.26E-13 | 3.32E-10 | 19.09699 |
| CKAP2L | -2.36903 | 12.8585 | -9.34646 | 8.54E-13 | 3.84E-10 | 18.9392 |
| RPS20 | 1.433728 | 13.14429 | 9.342976 | 8.64E-13 | 3.84E-10 | 18.92713 |
| RPL30 | 1.297767 | 13.20532 | 9.336753 | 8.84E-13 | 3.87E-10 | 18.90558 |
| KNL1 | -2.0807 | 13.06844 | -9.31629 | 9.51E-13 | 4.10E-10 | 18.83473 |
| TOP2A | -2.41195 | 12.9336 | -9.30136 | 1.00E-12 | 4.26E-10 | 18.78301 |
| RPS12 | 1.766498 | 12.90836 | 9.292731 | 1.03E-12 | 4.34E-10 | 18.75309 |
| PCLAF | -1.93491 | 13.06972 | -9.26523 | 1.14E-12 | 4.72E-10 | 18.6577 |
| RPS10 | 1.684222 | 13.17655 | 9.260748 | 1.16E-12 | 4.73E-10 | 18.64216 |
| SPC25 | -2.3263 | 12.99706 | -9.23674 | 1.26E-12 | 5.08E-10 | 18.55883 |
| H1-12P | -2.72356 | 13.26731 | -9.21021 | 1.39E-12 | 5.45E-10 | 18.46665 |
| SKA1 | -2.11589 | 13.01941 | -9.20999 | 1.39E-12 | 5.45E-10 | 18.46591 |
| TTK | -2.04111 | 13.00741 | -9.20407 | 1.42E-12 | 5.50E-10 | 18.44531 |
| KIF23 | -1.58932 | 13.05026 | -9.17383 | 1.58E-12 | 6.05E-10 | 18.34017 |
| E2F7 | -2.73456 | 12.87407 | -9.16686 | 1.62E-12 | 6.12E-10 | 18.31591 |
| PKMYT1 | -2.97844 | 12.57911 | -9.15925 | 1.67E-12 | 6.21E-10 | 18.28943 |
| RPS23 | 1.342104 | 13.20333 | 9.127455 | 1.87E-12 | 6.79E-10 | 18.17872 |
| LMNB1 | -1.55788 | 13.39328 | -9.12552 | 1.88E-12 | 6.79E-10 | 18.17197 |
| UHRF1 | -1.93656 | 13.06992 | -9.12439 | 1.89E-12 | 6.79E-10 | 18.16806 |
| BRCA1 | -1.37798 | 13.3369 | -9.11558 | 1.95E-12 | 6.92E-10 | 18.13733 |
| RPL5 | 1.357706 | 13.2245 | 9.094225 | 2.11E-12 | 7.39E-10 | 18.0629 |
| F5 | -2.08331 | 13.22457 | -9.06977 | 2.30E-12 | 7.97E-10 | 17.97758 |
| TYMS | -2.40664 | 12.86493 | -9.0486 | 2.48E-12 | 8.51E-10 | 17.9037 |
| RPL6 | 1.24025 | 13.23778 | 9.031634 | 2.64E-12 | 8.94E-10 | 17.84444 |
| RPL7 | 1.276584 | 13.22399 | 9.022496 | 2.72E-12 | 9.11E-10 | 17.81252 |
| MELK | -2.32757 | 13.0151 | -9.02034 | 2.75E-12 | 9.11E-10 | 17.80499 |
| HASPIN | -2.30791 | 13.137 | -8.99436 | 3.01E-12 | 9.85E-10 | 17.71417 |
| EEF1A1 | 1.412201 | 13.22191 | 8.99241 | 3.04E-12 | 9.85E-10 | 17.70735 |
| UBE2J1 | -1.45259 | 13.39477 | -8.97286 | 3.26E-12 | 1.05E-09 | 17.63896 |
| CENPE | -2.01324 | 12.96047 | -8.93964 | 3.67E-12 | 1.17E-09 | 17.52267 |
| E2F2 | -1.92451 | 12.66219 | -8.92633 | 3.85E-12 | 1.21E-09 | 17.47602 |
| CDT1 | -2.45057 | 12.81868 | -8.91768 | 3.97E-12 | 1.23E-09 | 17.44574 |
| H4C4 | -2.6695 | 13.22367 | -8.91303 | 4.04E-12 | 1.23E-09 | 17.42943 |
| AURKB | -2.29398 | 12.9278 | -8.91142 | 4.06E-12 | 1.23E-09 | 17.42378 |
| CDK1 | -2.23204 | 12.88296 | -8.91113 | 4.07E-12 | 1.23E-09 | 17.42279 |
| ITGA7 | -4.11927 | 12.61085 | -8.89687 | 4.28E-12 | 1.28E-09 | 17.37276 |
| KIF2C | -2.30574 | 12.82672 | -8.88987 | 4.39E-12 | 1.30E-09 | 17.34822 |
| SLC26A8 | -2.90998 | 13.14422 | -8.86184 | 4.86E-12 | 1.42E-09 | 17.24989 |
| AC103563.5 | -5.5825 | 12.25262 | -8.84324 | 5.20E-12 | 1.51E-09 | 17.18461 |
| KIFC1 | -2.15924 | 12.91911 | -8.82657 | 5.52E-12 | 1.59E-09 | 17.12603 |
| CDC25C | -2.45707 | 12.68418 | -8.81612 | 5.73E-12 | 1.63E-09 | 17.08934 |
| RPS23P8 | 1.763231 | 13.19919 | 8.80885 | 5.89E-12 | 1.66E-09 | 17.06378 |
| RPS3 | 1.278872 | 13.22986 | 8.798102 | 6.12E-12 | 1.71E-09 | 17.02601 |
| CENPF | -1.9839 | 12.87686 | -8.77742 | 6.60E-12 | 1.83E-09 | 16.95329 |
| RPL23A | 1.36878 | 13.25534 | 8.766851 | 6.85E-12 | 1.88E-09 | 16.91609 |
| PYCR1 | -3.10728 | 12.89494 | -8.74096 | 7.53E-12 | 2.05E-09 | 16.82498 |
| CEP55 | -2.28275 | 13.0712 | -8.7301 | 7.83E-12 | 2.11E-09 | 16.78674 |
| SHISA4 | 2.702463 | 12.08589 | 8.720393 | 8.11E-12 | 2.17E-09 | 16.75254 |
| KCNN3 | -3.27302 | 12.85418 | -8.70431 | 8.60E-12 | 2.26E-09 | 16.69589 |
| RPS13 | 1.242955 | 13.21887 | 8.704268 | 8.60E-12 | 2.26E-09 | 16.69572 |
| KLRB1 | 2.978888 | 12.77713 | 8.694317 | 8.91E-12 | 2.32E-09 | 16.66065 |
| TK1 | -2.48349 | 12.83546 | -8.6808 | 9.36E-12 | 2.42E-09 | 16.61301 |
| RRBP1 | -1.80556 | 13.35408 | -8.66951 | 9.75E-12 | 2.49E-09 | 16.57318 |
| NES | -4.13134 | 12.46292 | -8.65906 | 1.01E-11 | 2.57E-09 | 16.53634 |
| RPL32 | 1.36762 | 13.18762 | 8.649944 | 1.05E-11 | 2.63E-09 | 16.50416 |
| NUSAP1 | -1.81773 | 12.93093 | -8.64302 | 1.07E-11 | 2.68E-09 | 16.47973 |
| GGH | -2.22455 | 13.12645 | -8.63767 | 1.09E-11 | 2.71E-09 | 16.46082 |
| SLCO4A1 | -2.69866 | 13.07428 | -8.63239 | 1.12E-11 | 2.73E-09 | 16.44221 |
| TROAP | -2.56258 | 12.38126 | -8.63086 | 1.12E-11 | 2.73E-09 | 16.43681 |
| AL022722.1 | -2.69173 | 12.66064 | -8.62548 | 1.14E-11 | 2.75E-09 | 16.41782 |
| NCKAP5 | 2.93992 | 12.31245 | 8.624255 | 1.15E-11 | 2.75E-09 | 16.41348 |
| RPS17 | 1.214317 | 13.19961 | 8.603451 | 1.24E-11 | 2.95E-09 | 16.34 |
| RPL17 | 1.175585 | 13.2325 | 8.595211 | 1.28E-11 | 3.01E-09 | 16.31089 |
| FAM20A | -3.53064 | 12.72458 | -8.57027 | 1.40E-11 | 3.27E-09 | 16.22275 |
| NEK2 | -2.21509 | 12.82726 | -8.54303 | 1.54E-11 | 3.59E-09 | 16.12639 |
| CD207 | 3.577087 | 12.13852 | 8.519244 | 1.68E-11 | 3.88E-09 | 16.04223 |
| RPL19 | 1.204901 | 13.25407 | 8.516934 | 1.70E-11 | 3.88E-09 | 16.03406 |
| RPL13A | 1.342532 | 13.2065 | 8.511774 | 1.73E-11 | 3.93E-09 | 16.01579 |
| KIF11 | -1.99417 | 13.00517 | -8.50941 | 1.75E-11 | 3.93E-09 | 16.00743 |
| SPAG4 | -2.55639 | 13.00498 | -8.50671 | 1.76E-11 | 3.94E-09 | 15.99787 |
| RPL31 | 1.235391 | 13.13567 | 8.50097 | 1.80E-11 | 3.99E-09 | 15.97753 |
| FER1L4 | -2.84741 | 12.94509 | -8.48648 | 1.90E-11 | 4.16E-09 | 15.92621 |
| AC099560.2 | 1.467307 | 13.18846 | 8.485722 | 1.90E-11 | 4.16E-09 | 15.92353 |
| CENPW | -1.83417 | 12.97334 | -8.47872 | 1.95E-11 | 4.24E-09 | 15.89871 |
| RPS18 | 1.443314 | 13.19373 | 8.475861 | 1.97E-11 | 4.25E-09 | 15.88859 |
| CD1C | 2.416285 | 12.76377 | 8.459636 | 2.09E-11 | 4.45E-09 | 15.8311 |
| EEF1A1P19 | 1.715691 | 13.21652 | 8.459248 | 2.10E-11 | 4.45E-09 | 15.82972 |
| CCNE2 | -1.55876 | 13.10649 | -8.45448 | 2.13E-11 | 4.50E-09 | 15.81283 |
| CENPA | -2.37769 | 12.85574 | -8.45227 | 2.15E-11 | 4.51E-09 | 15.80499 |
| RPL15 | 1.106199 | 13.2556 | 8.44442 | 2.21E-11 | 4.60E-09 | 15.77716 |
| CROCC2 | 4.315046 | 10.63183 | 8.439743 | 2.25E-11 | 4.65E-09 | 15.76057 |
| DEPDC1B | -2.06526 | 12.99932 | -8.43724 | 2.27E-11 | 4.66E-09 | 15.75171 |
| RPS8 | 1.226866 | 13.18912 | 8.435384 | 2.29E-11 | 4.66E-09 | 15.74512 |
| ALDH1L2 | -2.8293 | 13.05397 | -8.42559 | 2.37E-11 | 4.80E-09 | 15.71036 |
| TRIB1 | -1.20667 | 13.40945 | -8.41047 | 2.50E-11 | 5.04E-09 | 15.65675 |
| ANLN | -2.50105 | 12.62767 | -8.37736 | 2.82E-11 | 5.63E-09 | 15.5392 |
| RPL35A | 1.173414 | 13.17082 | 8.374318 | 2.86E-11 | 5.63E-09 | 15.52841 |
| TRIP13 | -2.45568 | 13.04891 | -8.37361 | 2.86E-11 | 5.63E-09 | 15.52591 |
| HBB | 5.352281 | 11.05848 | 8.372545 | 2.87E-11 | 5.63E-09 | 15.52212 |
| ASS1 | -2.94946 | 13.05668 | -8.37018 | 2.90E-11 | 5.65E-09 | 15.51371 |
| BHLHA15 | -3.79117 | 12.70205 | -8.35948 | 3.02E-11 | 5.83E-09 | 15.47571 |
| SPAG5 | -1.96878 | 13.03855 | -8.34412 | 3.19E-11 | 6.13E-09 | 15.42114 |
| NCAPH | -2.06566 | 13.03315 | -8.31109 | 3.60E-11 | 6.87E-09 | 15.30373 |
| H2BC8 | -2.46304 | 13.06708 | -8.30375 | 3.70E-11 | 7.01E-09 | 15.27765 |
| CYP19A1 | -4.70007 | 12.22362 | -8.30218 | 3.72E-11 | 7.01E-09 | 15.27206 |
| RPS11 | 1.042925 | 13.20547 | 8.293733 | 3.83E-11 | 7.19E-09 | 15.24202 |
| EEF1B2 | 1.32477 | 13.2242 | 8.283126 | 3.99E-11 | 7.43E-09 | 15.20429 |
| DYSF | -2.10422 | 13.1651 | -8.26338 | 4.28E-11 | 7.92E-09 | 15.13401 |
| NTNG2 | -2.56953 | 13.1151 | -8.261 | 4.32E-11 | 7.92E-09 | 15.12557 |
| GINS1 | -1.65906 | 13.22825 | -8.26063 | 4.33E-11 | 7.92E-09 | 15.12424 |
| METTL7B | -4.79042 | 12.3005 | -8.24923 | 4.51E-11 | 8.20E-09 | 15.08366 |
| MTF1 | -1.41571 | 13.40611 | -8.23846 | 4.69E-11 | 8.48E-09 | 15.0453 |
| GINS2 | -2.06121 | 13.03636 | -8.22946 | 4.85E-11 | 8.69E-09 | 15.01328 |
| PLPPR4 | 1.851771 | 13.12428 | 8.228644 | 4.87E-11 | 8.69E-09 | 15.01036 |
| TEDC2 | -2.54756 | 13.00532 | -8.21282 | 5.16E-11 | 9.15E-09 | 14.95399 |
| RPS25 | 1.1507 | 13.19292 | 8.203137 | 5.34E-11 | 9.43E-09 | 14.91949 |
| FOSL1 | -2.3283 | 12.86084 | -8.18809 | 5.64E-11 | 9.91E-09 | 14.86589 |
| PFDN5 | 1.089066 | 13.15956 | 8.167271 | 6.09E-11 | 1.06E-08 | 14.79166 |
| CDCA3 | -1.8229 | 12.99233 | -8.13527 | 6.85E-11 | 1.18E-08 | 14.67752 |
| ZWINT | -2.13888 | 13.05832 | -8.1343 | 6.87E-11 | 1.18E-08 | 14.67405 |
| LY6G6C | -3.24777 | 13.00417 | -8.12934 | 7.00E-11 | 1.19E-08 | 14.65638 |
| NOS1AP | -2.84557 | 12.95582 | -8.10249 | 7.73E-11 | 1.31E-08 | 14.56056 |
| AC008984.5 | -2.51388 | 13.19984 | -8.07507 | 8.54E-11 | 1.42E-08 | 14.46263 |
| AC245884.2 | -2.51388 | 13.19984 | -8.07507 | 8.54E-11 | 1.42E-08 | 14.46263 |
| ARNTL2 | -1.51433 | 13.3823 | -8.06498 | 8.87E-11 | 1.47E-08 | 14.4266 |
| RPL26 | 1.587118 | 13.20825 | 8.059593 | 9.04E-11 | 1.49E-08 | 14.40736 |
| AC115223.1 | 1.161057 | 13.24541 | 8.048577 | 9.42E-11 | 1.54E-08 | 14.368 |
| RNASE1 | -4.8291 | 12.31794 | -8.0471 | 9.47E-11 | 1.54E-08 | 14.36272 |
| RPL4 | 1.206792 | 13.23283 | 8.034385 | 9.92E-11 | 1.61E-08 | 14.31729 |
| GALNT14 | -3.11941 | 12.94134 | -8.033 | 9.97E-11 | 1.61E-08 | 14.31235 |
| CENPM | -2.24639 | 12.93959 | -8.0285 | 1.01E-10 | 1.62E-08 | 14.29626 |
| RPL10 | 1.3646 | 13.1792 | 8.027754 | 1.02E-10 | 1.62E-08 | 14.29359 |
| RPL11 | 1.140351 | 13.29912 | 8.026227 | 1.02E-10 | 1.62E-08 | 14.28813 |
| H2BC5 | -2.69984 | 13.15878 | -8.02518 | 1.03E-10 | 1.62E-08 | 14.28439 |
| PBK | -2.67585 | 12.90511 | -8.02194 | 1.04E-10 | 1.63E-08 | 14.27282 |
| RPL24 | 1.065619 | 13.27424 | 7.998453 | 1.13E-10 | 1.77E-08 | 14.18884 |
| B3GALT2 | 3.080639 | 12.75096 | 7.972473 | 1.25E-10 | 1.94E-08 | 14.09591 |
| NUF2 | -1.74673 | 12.9947 | -7.96393 | 1.29E-10 | 1.99E-08 | 14.06536 |
| NDRG2 | 1.914698 | 13.03047 | 7.953751 | 1.33E-10 | 2.05E-08 | 14.02893 |
| ENHO | 3.576792 | 11.89567 | 7.935908 | 1.42E-10 | 2.18E-08 | 13.96507 |
| APOBR | -1.79088 | 13.36829 | -7.91686 | 1.53E-10 | 2.33E-08 | 13.89689 |
| CACNA2D3 | 3.849469 | 12.00675 | 7.914259 | 1.54E-10 | 2.34E-08 | 13.88757 |
| RPL37 | 1.067763 | 13.21022 | 7.913454 | 1.55E-10 | 2.34E-08 | 13.88468 |
| ORC6 | -1.40762 | 13.23911 | -7.90288 | 1.61E-10 | 2.42E-08 | 13.84682 |
| SLC4A10 | 4.068419 | 12.23447 | 7.88102 | 1.74E-10 | 2.61E-08 | 13.76852 |
| ESCO2 | -1.92785 | 13.15252 | -7.85857 | 1.89E-10 | 2.82E-08 | 13.68808 |
| CKAP4 | -1.80719 | 13.17642 | -7.85517 | 1.92E-10 | 2.84E-08 | 13.6759 |
| CLU | -2.12487 | 13.0736 | -7.85124 | 1.95E-10 | 2.86E-08 | 13.66182 |
| EEF1A1P6 | 1.685978 | 13.18454 | 7.849685 | 1.96E-10 | 2.87E-08 | 13.65625 |
| IL10 | -2.0853 | 13.32449 | -7.84351 | 2.00E-10 | 2.92E-08 | 13.63412 |
| OIP5 | -1.52108 | 13.17533 | -7.8365 | 2.05E-10 | 2.98E-08 | 13.609 |
| UCHL1 | -4.32221 | 12.49844 | -7.8046 | 2.31E-10 | 3.34E-08 | 13.49463 |
| APOBEC3B | -2.36299 | 13.25589 | -7.79173 | 2.42E-10 | 3.47E-08 | 13.44845 |
| CCNB1 | -1.89474 | 13.00767 | -7.76711 | 2.65E-10 | 3.77E-08 | 13.36016 |
| MZB1 | -3.06972 | 12.88126 | -7.76662 | 2.66E-10 | 3.77E-08 | 13.35838 |
| H2BC6 | -2.52831 | 13.30365 | -7.76451 | 2.68E-10 | 3.78E-08 | 13.35083 |
| B4GALT5 | -1.65585 | 13.24159 | -7.76278 | 2.70E-10 | 3.78E-08 | 13.34459 |
| CIT | -1.96665 | 12.69022 | -7.76214 | 2.70E-10 | 3.78E-08 | 13.3423 |
| TTC9 | 1.66156 | 13.14571 | 7.759355 | 2.73E-10 | 3.80E-08 | 13.33232 |
| LTK | 1.916562 | 13.16718 | 7.753539 | 2.79E-10 | 3.85E-08 | 13.31145 |
| RPL27 | 1.114405 | 13.19658 | 7.7533 | 2.79E-10 | 3.85E-08 | 13.3106 |
| C1orf226 | -3.02053 | 12.78812 | -7.75171 | 2.81E-10 | 3.85E-08 | 13.30489 |
| MUC1 | -1.87771 | 13.12527 | -7.72168 | 3.14E-10 | 4.28E-08 | 13.19712 |
| RPS29 | 1.335421 | 13.14249 | 7.715387 | 3.21E-10 | 4.37E-08 | 13.17452 |
| IGHG1 | -4.62425 | 12.13316 | -7.70747 | 3.31E-10 | 4.47E-08 | 13.14611 |
| PIF1 | -1.47996 | 13.19515 | -7.69507 | 3.46E-10 | 4.66E-08 | 13.10157 |
| MAFG | -1.51292 | 13.10149 | -7.68703 | 3.56E-10 | 4.78E-08 | 13.07271 |
| HBA2 | 4.582948 | 11.13739 | 7.678602 | 3.68E-10 | 4.91E-08 | 13.04243 |
| RPL10A | 1.241243 | 13.23746 | 7.673095 | 3.75E-10 | 4.99E-08 | 13.02265 |
| RPL23 | 1.050371 | 13.28535 | 7.671873 | 3.77E-10 | 4.99E-08 | 13.01826 |
| NOL3 | -1.65562 | 13.29234 | -7.66875 | 3.81E-10 | 5.03E-08 | 13.00703 |
| DUSP13 | -3.91587 | 12.21808 | -7.64674 | 4.14E-10 | 5.43E-08 | 12.92796 |
| RPL12 | 1.238502 | 13.18877 | 7.644239 | 4.17E-10 | 5.43E-08 | 12.91899 |
| RPLP2 | 1.256871 | 13.10852 | 7.643526 | 4.19E-10 | 5.43E-08 | 12.91643 |
| RNASE2 | -2.36856 | 12.97049 | -7.6425 | 4.20E-10 | 5.43E-08 | 12.91273 |
| EEF1G | 1.134978 | 13.23724 | 7.641825 | 4.21E-10 | 5.43E-08 | 12.91032 |
| UBA52 | 1.617354 | 12.75925 | 7.638584 | 4.26E-10 | 5.48E-08 | 12.89867 |
| POC1A | -1.51157 | 13.15922 | -7.63477 | 4.32E-10 | 5.53E-08 | 12.88495 |
| RPL3P4 | 1.520697 | 13.1698 | 7.620423 | 4.56E-10 | 5.81E-08 | 12.83341 |
| RPL37A | 1.06617 | 13.25705 | 7.614491 | 4.66E-10 | 5.91E-08 | 12.81209 |
| SEMA3B | -2.21966 | 13.28277 | -7.60961 | 4.74E-10 | 5.99E-08 | 12.79457 |
| IL17RE | 1.62275 | 13.2259 | 7.598113 | 4.95E-10 | 6.22E-08 | 12.75323 |
| RAD51 | -1.80735 | 12.93792 | -7.59712 | 4.97E-10 | 6.22E-08 | 12.74965 |
| ACOXL | -2.03409 | 13.22159 | -7.59439 | 5.02E-10 | 6.26E-08 | 12.73986 |
| IGLC3 | -3.30906 | 12.78028 | -7.58161 | 5.26E-10 | 6.54E-08 | 12.69392 |
| MND1 | -2.11027 | 12.91088 | -7.58007 | 5.29E-10 | 6.55E-08 | 12.68837 |
| SGO1 | -1.41157 | 13.05841 | -7.5727 | 5.44E-10 | 6.70E-08 | 12.66187 |
| NRCAM | 3.717757 | 12.35422 | 7.559034 | 5.72E-10 | 7.02E-08 | 12.61273 |
| HP | -3.50324 | 12.29584 | -7.55031 | 5.91E-10 | 7.22E-08 | 12.58134 |
| KRT8P26 | -2.51901 | 12.88374 | -7.52899 | 6.39E-10 | 7.78E-08 | 12.5047 |
| IGLV3-1 | -3.95481 | 12.58065 | -7.52722 | 6.43E-10 | 7.80E-08 | 12.49831 |
| IGHJ3 | -3.06948 | 12.7417 | -7.5231 | 6.53E-10 | 7.89E-08 | 12.48349 |
| EIF3L | 1.134179 | 13.20325 | 7.519928 | 6.61E-10 | 7.95E-08 | 12.47209 |
| ELL2 | -1.96563 | 13.16702 | -7.50807 | 6.90E-10 | 8.27E-08 | 12.42943 |
| PNPLA1 | -2.03699 | 13.1946 | -7.49371 | 7.28E-10 | 8.69E-08 | 12.37777 |
| PPARG | -3.01953 | 12.76931 | -7.47612 | 7.77E-10 | 9.23E-08 | 12.31447 |
| IL36A | 2.43457 | 13.13363 | 7.471423 | 7.91E-10 | 9.36E-08 | 12.29758 |
| CHEK1 | -1.7212 | 13.18602 | -7.46481 | 8.10E-10 | 9.55E-08 | 12.27379 |
| RPL13 | 1.277959 | 13.14004 | 7.456253 | 8.36E-10 | 9.82E-08 | 12.24299 |
| TRAJ33 | 2.694094 | 12.9748 | 7.452376 | 8.48E-10 | 9.92E-08 | 12.22903 |
| RORC | 2.48979 | 12.93963 | 7.433599 | 9.09E-10 | 1.06E-07 | 12.16145 |
| RPL7A | 1.040799 | 13.27168 | 7.429915 | 9.22E-10 | 1.07E-07 | 12.14819 |
| SLC18A1 | 2.364019 | 12.7982 | 7.425561 | 9.37E-10 | 1.08E-07 | 12.13252 |
| G0S2 | -2.7485 | 13.01187 | -7.4125 | 9.83E-10 | 1.13E-07 | 12.08548 |
| RANBP6 | 1.113257 | 13.37992 | 7.408657 | 9.97E-10 | 1.14E-07 | 12.07167 |
| ADAT2 | 1.147009 | 13.26566 | 7.399415 | 1.03E-09 | 1.18E-07 | 12.03839 |
| AURKA | -1.51873 | 12.94361 | -7.39894 | 1.03E-09 | 1.18E-07 | 12.03669 |
| AL683842.1 | -2.2782 | 13.20995 | -7.39331 | 1.06E-09 | 1.20E-07 | 12.01641 |
| AP5B1 | -1.65948 | 13.33189 | -7.39082 | 1.07E-09 | 1.20E-07 | 12.00743 |
| CTSD | -1.56175 | 13.23327 | -7.382 | 1.10E-09 | 1.24E-07 | 11.97569 |
| E2F1 | -2.34212 | 12.8986 | -7.38168 | 1.10E-09 | 1.24E-07 | 11.97455 |
| HMGB2 | -1.59733 | 13.14587 | -7.37338 | 1.14E-09 | 1.27E-07 | 11.94465 |
| MFSD14C | -1.37387 | 13.36257 | -7.37032 | 1.15E-09 | 1.28E-07 | 11.93365 |
| CR1 | -2.01686 | 13.2302 | -7.35174 | 1.23E-09 | 1.37E-07 | 11.86672 |
| MTFR2 | -1.20058 | 13.24578 | -7.33138 | 1.33E-09 | 1.47E-07 | 11.7934 |
| ITGAM | -1.36166 | 13.27525 | -7.32883 | 1.34E-09 | 1.48E-07 | 11.78422 |
| STIL | -1.64084 | 13.22809 | -7.32561 | 1.36E-09 | 1.48E-07 | 11.77263 |
| ID3 | 1.924914 | 13.0576 | 7.325598 | 1.36E-09 | 1.48E-07 | 11.77258 |
| RPL22P1 | 1.151721 | 13.27352 | 7.321847 | 1.38E-09 | 1.50E-07 | 11.75907 |
| RPL22 | 1.19623 | 13.26822 | 7.32056 | 1.38E-09 | 1.50E-07 | 11.75443 |
| CDCA8 | -1.5236 | 13.07698 | -7.29795 | 1.50E-09 | 1.62E-07 | 11.673 |
| MXD3 | -1.88714 | 13.19724 | -7.27801 | 1.62E-09 | 1.74E-07 | 11.60117 |
| LY6G6E | -2.43589 | 12.98921 | -7.27695 | 1.62E-09 | 1.74E-07 | 11.59736 |
| ERCC6L | -1.69623 | 13.11988 | -7.25449 | 1.76E-09 | 1.88E-07 | 11.51642 |
| UBE2S | -1.32989 | 13.11418 | -7.2472 | 1.81E-09 | 1.93E-07 | 11.49017 |
| RPL27A | 1.128456 | 13.21776 | 7.236594 | 1.89E-09 | 2.00E-07 | 11.45195 |
| HTR3A | 2.824746 | 12.83782 | 7.224946 | 1.97E-09 | 2.08E-07 | 11.40998 |
| ELOVL4 | 2.839398 | 12.91578 | 7.219503 | 2.01E-09 | 2.11E-07 | 11.39037 |
| CDKN3 | -1.75941 | 12.91988 | -7.21906 | 2.01E-09 | 2.11E-07 | 11.38876 |
| EME1 | -1.5032 | 13.23371 | -7.21157 | 2.07E-09 | 2.16E-07 | 11.36179 |
| AC107075.1 | 1.154418 | 13.28002 | 7.210432 | 2.08E-09 | 2.16E-07 | 11.35769 |
| RPL38 | 1.042223 | 13.16271 | 7.209961 | 2.08E-09 | 2.16E-07 | 11.35599 |
| MCEMP1 | -2.71674 | 12.86559 | -7.2073 | 2.10E-09 | 2.17E-07 | 11.34639 |
| H2BC7 | -2.68098 | 13.1751 | -7.20366 | 2.13E-09 | 2.19E-07 | 11.33327 |
| AUNIP | -1.64105 | 12.97691 | -7.20277 | 2.14E-09 | 2.19E-07 | 11.33006 |
| RPL21P131 | 1.388448 | 14.6582 | 7.201895 | 2.14E-09 | 2.19E-07 | 11.32693 |
| NCR3 | 1.922914 | 13.12436 | 7.187949 | 2.26E-09 | 2.30E-07 | 11.27667 |
| DMC1 | -2.02929 | 13.07673 | -7.1772 | 2.35E-09 | 2.37E-07 | 11.23794 |
| KIF18A | -1.39536 | 13.09258 | -7.17522 | 2.37E-09 | 2.38E-07 | 11.23081 |
| ADM | -2.04881 | 13.23723 | -7.16262 | 2.48E-09 | 2.48E-07 | 11.1854 |
| SLC36A1 | -1.39777 | 13.21505 | -7.16256 | 2.48E-09 | 2.48E-07 | 11.18518 |
| IGHV4-59 | -3.07624 | 12.82648 | -7.15429 | 2.56E-09 | 2.55E-07 | 11.1554 |
| WDR62 | -1.53673 | 13.05411 | -7.15303 | 2.57E-09 | 2.55E-07 | 11.15084 |
| EEF1A1P9 | 2.192333 | 13.13235 | 7.150895 | 2.59E-09 | 2.56E-07 | 11.14315 |
| RPL18A | 1.189642 | 13.19475 | 7.133226 | 2.76E-09 | 2.73E-07 | 11.07948 |
| SHROOM2P1 | 1.739973 | 14.35738 | 7.126739 | 2.83E-09 | 2.78E-07 | 11.0561 |
| AL031777.1 | -2.44868 | 13.2146 | -7.12019 | 2.90E-09 | 2.84E-07 | 11.03248 |
| MET | -2.60959 | 13.02269 | -7.11825 | 2.92E-09 | 2.85E-07 | 11.0255 |
| OPRL1 | -1.28544 | 13.3666 | -7.0905 | 3.24E-09 | 3.15E-07 | 10.92551 |
| FAM72B | -1.44527 | 13.18365 | -7.07859 | 3.39E-09 | 3.29E-07 | 10.88258 |
| KCND1 | -1.92104 | 13.22746 | -7.0689 | 3.51E-09 | 3.39E-07 | 10.84766 |
| FAM72A | -1.40571 | 13.22775 | -7.06606 | 3.55E-09 | 3.42E-07 | 10.83741 |
| PLBD1 | -1.65382 | 13.2014 | -7.04727 | 3.80E-09 | 3.65E-07 | 10.7697 |
| IGHV1-24 | -5.56861 | 11.43594 | -7.03589 | 3.97E-09 | 3.80E-07 | 10.7287 |
| RPSA | 1.177188 | 13.23248 | 7.030271 | 4.05E-09 | 3.87E-07 | 10.70844 |
| PITPNC1 | 1.034256 | 13.32888 | 7.027898 | 4.08E-09 | 3.89E-07 | 10.69989 |
| POLE2 | -1.37065 | 13.21205 | -7.02334 | 4.15E-09 | 3.94E-07 | 10.68347 |
| RPL3 | 1.243584 | 13.24627 | 7.020378 | 4.20E-09 | 3.96E-07 | 10.67279 |
| WNT10B | 1.824068 | 12.96105 | 7.020296 | 4.20E-09 | 3.96E-07 | 10.6725 |
| EEF1A1P12 | 1.735241 | 13.14081 | 7.018633 | 4.23E-09 | 3.97E-07 | 10.6665 |
| RPS16 | 1.137967 | 13.21286 | 7.009589 | 4.37E-09 | 4.10E-07 | 10.63392 |
| GALNT12 | 1.629215 | 13.21069 | 7.004608 | 4.45E-09 | 4.15E-07 | 10.61597 |
| CARD6 | -1.3023 | 13.35044 | -7.00434 | 4.46E-09 | 4.15E-07 | 10.61502 |
| CRISPLD2 | -1.5989 | 13.3032 | -6.9953 | 4.61E-09 | 4.27E-07 | 10.58242 |
| HMGB3 | -1.95662 | 12.95876 | -6.98868 | 4.72E-09 | 4.36E-07 | 10.55858 |
| IGLC2 | -3.14197 | 12.79991 | -6.98025 | 4.87E-09 | 4.48E-07 | 10.52818 |
| IGLV2-14 | -2.91773 | 12.803 | -6.97423 | 4.98E-09 | 4.57E-07 | 10.5065 |
| IGLV1-40 | -3.06833 | 12.75482 | -6.95939 | 5.26E-09 | 4.80E-07 | 10.45302 |
| TXNDC5 | -2.92845 | 12.87432 | -6.95913 | 5.27E-09 | 4.80E-07 | 10.4521 |
| CMTM1 | -1.717 | 13.33023 | -6.95883 | 5.28E-09 | 4.80E-07 | 10.45102 |
| H2BC12 | -1.55829 | 13.28306 | -6.94995 | 5.45E-09 | 4.94E-07 | 10.419 |
| DPH5 | 1.021991 | 13.37133 | 6.941476 | 5.63E-09 | 5.07E-07 | 10.38848 |
| CFAP36 | 1.18701 | 13.29541 | 6.930722 | 5.85E-09 | 5.26E-07 | 10.34973 |
| MDS2 | 1.669771 | 13.06125 | 6.918776 | 6.12E-09 | 5.48E-07 | 10.30669 |
| TNFAIP8L3 | -3.21553 | 12.74633 | -6.91512 | 6.20E-09 | 5.54E-07 | 10.29352 |
| TMEM42 | 1.062897 | 13.23658 | 6.908174 | 6.36E-09 | 5.65E-07 | 10.2685 |
| UNC5A | -1.90781 | 13.23969 | -6.89979 | 6.57E-09 | 5.79E-07 | 10.23828 |
| SLC1A3 | -3.20032 | 12.62513 | -6.89849 | 6.60E-09 | 5.81E-07 | 10.23362 |
| INAVA | -2.26197 | 12.7969 | -6.88708 | 6.88E-09 | 6.02E-07 | 10.19249 |
| SCNN1B | -2.89414 | 12.89241 | -6.88694 | 6.89E-09 | 6.02E-07 | 10.19202 |
| IGLJ2 | -3.19092 | 12.7778 | -6.87979 | 7.07E-09 | 6.17E-07 | 10.16623 |
| FKBP9 | -1.68502 | 13.02229 | -6.87759 | 7.13E-09 | 6.20E-07 | 10.15833 |
| IGLV1-36 | -4.28182 | 12.21304 | -6.8712 | 7.30E-09 | 6.33E-07 | 10.1353 |
| H2AC17 | -3.05229 | 12.8662 | -6.86976 | 7.34E-09 | 6.35E-07 | 10.1301 |
| ADAMTS2 | -5.69831 | 11.42074 | -6.86597 | 7.44E-09 | 6.42E-07 | 10.11647 |
| GPR84 | -2.96723 | 12.55759 | -6.86491 | 7.47E-09 | 6.42E-07 | 10.11264 |
| LYPD1 | 2.160027 | 13.23409 | 6.859657 | 7.62E-09 | 6.53E-07 | 10.09373 |
| IGKV4-1 | -2.76035 | 12.549 | -6.84754 | 7.97E-09 | 6.81E-07 | 10.05007 |
| EEF1A1P16 | 1.477143 | 13.18362 | 6.823597 | 8.71E-09 | 7.38E-07 | 9.963855 |
| JUN | -1.4519 | 13.32483 | -6.81806 | 8.89E-09 | 7.50E-07 | 9.943913 |
| GRN | -1.18423 | 13.3101 | -6.81738 | 8.91E-09 | 7.50E-07 | 9.94145 |
| MCM4 | -1.71321 | 13.19986 | -6.81696 | 8.92E-09 | 7.50E-07 | 9.939952 |
| LRRN3 | 3.596939 | 12.45559 | 6.813504 | 9.04E-09 | 7.58E-07 | 9.927508 |
| ADCK1 | 1.007656 | 13.26957 | 6.80487 | 9.33E-09 | 7.79E-07 | 9.896418 |
| RPL14 | 1.025493 | 13.27314 | 6.801468 | 9.45E-09 | 7.86E-07 | 9.884171 |
| MAOB | -3.43349 | 12.44072 | -6.79509 | 9.68E-09 | 8.01E-07 | 9.861205 |
| GZMK | 2.382528 | 12.8592 | 6.794863 | 9.68E-09 | 8.01E-07 | 9.860386 |
| PLPP3 | -1.83495 | 13.13989 | -6.77655 | 1.04E-08 | 8.53E-07 | 9.794449 |
| DNASE1L3 | 1.712172 | 13.21427 | 6.776059 | 1.04E-08 | 8.53E-07 | 9.792689 |
| PKHD1L1 | -2.2911 | 12.93862 | -6.77568 | 1.04E-08 | 8.53E-07 | 9.791318 |
| IGHV3-43 | -3.43896 | 12.49863 | -6.7734 | 1.05E-08 | 8.57E-07 | 9.783119 |
| AC116533.1 | 1.039287 | 13.21586 | 6.772511 | 1.05E-08 | 8.57E-07 | 9.779915 |
| H2BW2 | 1.602589 | 13.27714 | 6.772214 | 1.05E-08 | 8.57E-07 | 9.778845 |
| DPRXP2 | -2.86296 | 12.9879 | -6.77151 | 1.06E-08 | 8.57E-07 | 9.776294 |
| GPRIN1 | -1.44676 | 13.32029 | -6.76682 | 1.07E-08 | 8.69E-07 | 9.759412 |
| RPL36A | 1.03385 | 13.22404 | 6.76192 | 1.09E-08 | 8.83E-07 | 9.741787 |
| AC108161.1 | 1.984233 | 13.87218 | 6.760521 | 1.10E-08 | 8.85E-07 | 9.736752 |
| S100A9 | -1.85599 | 13.08849 | -6.75865 | 1.11E-08 | 8.87E-07 | 9.730034 |
| PRSS35 | 3.67364 | 12.3037 | 6.758603 | 1.11E-08 | 8.87E-07 | 9.729849 |
| KIF1B | -1.39091 | 13.31337 | -6.75787 | 1.11E-08 | 8.87E-07 | 9.727223 |
| IGLV4-69 | -3.86077 | 12.10629 | -6.75524 | 1.12E-08 | 8.93E-07 | 9.717744 |
| OPLAH | -2.55893 | 12.88544 | -6.75277 | 1.13E-08 | 8.99E-07 | 9.708864 |
| SCN1B | -1.79523 | 13.28969 | -6.75063 | 1.14E-08 | 9.04E-07 | 9.701152 |
| AL162615.1 | -2.00958 | 13.3425 | -6.74978 | 1.14E-08 | 9.04E-07 | 9.6981 |
| DRAM1 | -1.09542 | 13.37042 | -6.74227 | 1.18E-08 | 9.25E-07 | 9.671067 |
| RPS3AP5 | 2.448571 | 12.99111 | 6.728684 | 1.24E-08 | 9.70E-07 | 9.622164 |
| FAM72C | -3.16971 | 12.52868 | -6.72645 | 1.25E-08 | 9.76E-07 | 9.614106 |
| IGHV3-23 | -3.41861 | 12.68105 | -6.72512 | 1.25E-08 | 9.78E-07 | 9.609348 |
| DHFRP1 | -5.76891 | 11.77908 | -6.72092 | 1.27E-08 | 9.91E-07 | 9.594214 |
| IL17RA | -1.17891 | 13.36333 | -6.71974 | 1.28E-08 | 9.92E-07 | 9.589987 |
| IFI27L1 | -1.56538 | 13.21972 | -6.71864 | 1.28E-08 | 9.94E-07 | 9.586027 |
| SPATA20P1 | -2.92877 | 12.8948 | -6.71729 | 1.29E-08 | 9.96E-07 | 9.581164 |
| FAM92B | -2.77502 | 12.87478 | -6.70816 | 1.33E-08 | 1.03E-06 | 9.548317 |
| IGHV3-74 | -2.51135 | 12.98649 | -6.70245 | 1.36E-08 | 1.05E-06 | 9.527761 |
| IGLV3-25 | -4.02293 | 12.42014 | -6.70054 | 1.37E-08 | 1.05E-06 | 9.520886 |
| AC090498.1 | 1.664678 | 13.14327 | 6.696118 | 1.40E-08 | 1.07E-06 | 9.504978 |
| IGLV3-10 | -4.41029 | 11.88332 | -6.69377 | 1.41E-08 | 1.07E-06 | 9.496516 |
| CEP295NL | -1.70316 | 13.38227 | -6.69249 | 1.41E-08 | 1.08E-06 | 9.491918 |
| IGLV1-51 | -3.36822 | 12.45996 | -6.68958 | 1.43E-08 | 1.08E-06 | 9.481443 |
| IGHV1-18 | -3.79026 | 12.52896 | -6.67797 | 1.49E-08 | 1.12E-06 | 9.439705 |
| H4C9 | -1.4468 | 13.2913 | -6.67782 | 1.49E-08 | 1.12E-06 | 9.439136 |
| EAF2 | -1.45282 | 13.24461 | -6.66981 | 1.54E-08 | 1.15E-06 | 9.410345 |
| SLC2A3 | -1.55331 | 13.29132 | -6.66426 | 1.57E-08 | 1.17E-06 | 9.39036 |
| CYB561 | 1.173555 | 13.26964 | 6.654406 | 1.63E-08 | 1.21E-06 | 9.354934 |
| SLC7A11 | -1.36715 | 13.20288 | -6.65053 | 1.65E-08 | 1.23E-06 | 9.34098 |
| FZD5 | -1.6807 | 12.85251 | -6.64781 | 1.67E-08 | 1.24E-06 | 9.331202 |
| IGHA1 | -2.74916 | 12.89949 | -6.64636 | 1.68E-08 | 1.24E-06 | 9.325994 |
| CPE | -3.78615 | 12.31502 | -6.63822 | 1.73E-08 | 1.28E-06 | 9.296746 |
| DPH6 | 1.113133 | 13.2969 | 6.637098 | 1.74E-08 | 1.28E-06 | 9.292692 |
| ZFAND1 | 1.032123 | 13.36873 | 6.637062 | 1.74E-08 | 1.28E-06 | 9.292563 |
| GEM | 2.793998 | 12.69317 | 6.631589 | 1.77E-08 | 1.30E-06 | 9.272884 |
| AC090013.1 | 1.995064 | 13.27653 | 6.628989 | 1.79E-08 | 1.31E-06 | 9.263535 |
| SCARA5 | 3.333198 | 12.42958 | 6.62345 | 1.83E-08 | 1.33E-06 | 9.24362 |
| RPL18 | 1.069248 | 13.22253 | 6.619194 | 1.86E-08 | 1.35E-06 | 9.228319 |
| INSL3 | -1.82684 | 13.27296 | -6.61808 | 1.86E-08 | 1.35E-06 | 9.224332 |
| CDC42BPA | -1.69253 | 12.8625 | -6.61454 | 1.89E-08 | 1.37E-06 | 9.211584 |
| AC009245.1 | 1.87291 | 13.196 | 6.612313 | 1.90E-08 | 1.37E-06 | 9.203584 |
| FAM72D | -1.5217 | 13.19067 | -6.60618 | 1.95E-08 | 1.40E-06 | 9.181552 |
| AC116353.5 | -1.87642 | 13.17041 | -6.60085 | 1.99E-08 | 1.42E-06 | 9.162373 |
| MAPK14 | -1.44767 | 13.34999 | -6.5972 | 2.01E-08 | 1.44E-06 | 9.149276 |
| MAP2K6 | -1.56095 | 13.39929 | -6.5935 | 2.04E-08 | 1.45E-06 | 9.135955 |
| HLA-DPB2 | 2.164199 | 13.02251 | 6.583093 | 2.12E-08 | 1.50E-06 | 9.098558 |
| RTEL1P1 | -2.64868 | 13.12521 | -6.58255 | 2.12E-08 | 1.50E-06 | 9.096596 |
| ARID3A | -1.19886 | 13.29043 | -6.57954 | 2.15E-08 | 1.51E-06 | 9.085776 |
| RPS3AP21 | 2.337989 | 12.89852 | 6.575931 | 2.18E-08 | 1.53E-06 | 9.072822 |
| EEF1A1P4 | 1.7518 | 13.28 | 6.571264 | 2.21E-08 | 1.55E-06 | 9.05605 |
| AC006946.1 | -2.66166 | 13.04568 | -6.56972 | 2.23E-08 | 1.55E-06 | 9.050498 |
| COL5A1 | 2.150552 | 13.01812 | 6.569542 | 2.23E-08 | 1.55E-06 | 9.049866 |
| HLA-DPB1 | 1.634845 | 13.08414 | 6.566831 | 2.25E-08 | 1.57E-06 | 9.040123 |
| KREMEN1 | -2.30115 | 13.11097 | -6.56506 | 2.27E-08 | 1.57E-06 | 9.033778 |
| GYG1 | -1.48703 | 13.2028 | -6.5639 | 2.28E-08 | 1.58E-06 | 9.029584 |
| NEIL3 | -1.94783 | 12.64869 | -6.56049 | 2.30E-08 | 1.59E-06 | 9.017335 |
| SPATA7 | 1.142061 | 13.25179 | 6.557213 | 2.33E-08 | 1.61E-06 | 9.00557 |
| ELOVL3 | -1.91283 | 12.87788 | -6.55306 | 2.37E-08 | 1.63E-06 | 8.990639 |
| OSCAR | -1.31813 | 13.23173 | -6.55247 | 2.37E-08 | 1.63E-06 | 8.988515 |
| IGLV1-47 | -3.02392 | 12.71019 | -6.54912 | 2.40E-08 | 1.65E-06 | 8.976478 |
| EEF1A1P5 | 1.758218 | 13.19186 | 6.547722 | 2.42E-08 | 1.65E-06 | 8.971474 |
| IGKV2-28 | -3.00334 | 12.47778 | -6.54505 | 2.44E-08 | 1.66E-06 | 8.96186 |
| ZNF37CP | 1.891431 | 13.92836 | 6.544588 | 2.44E-08 | 1.66E-06 | 8.960216 |
| CTSA | -1.12364 | 13.31984 | -6.53916 | 2.49E-08 | 1.69E-06 | 8.940706 |
| QPCT | -1.29867 | 13.34806 | -6.5371 | 2.51E-08 | 1.70E-06 | 8.933309 |
| SIGLEC17P | 1.765819 | 12.9803 | 6.53232 | 2.56E-08 | 1.72E-06 | 8.916154 |
| IL23A | 1.625059 | 13.17855 | 6.532057 | 2.56E-08 | 1.72E-06 | 8.915208 |
| MMP9 | -3.46009 | 12.3532 | -6.52556 | 2.62E-08 | 1.76E-06 | 8.891891 |
| ADAM12 | 2.149182 | 13.08511 | 6.521732 | 2.66E-08 | 1.78E-06 | 8.878129 |
| PFN2 | 1.887674 | 13.14194 | 6.516566 | 2.71E-08 | 1.81E-06 | 8.859579 |
| MCMDC2 | 1.084513 | 13.31323 | 6.514509 | 2.73E-08 | 1.82E-06 | 8.852193 |
| AC244157.2 | 2.795183 | 12.50053 | 6.508905 | 2.79E-08 | 1.85E-06 | 8.832074 |
| IGLV2-11 | -2.72611 | 12.8236 | -6.50539 | 2.83E-08 | 1.87E-06 | 8.819449 |
| INSC | -2.13315 | 13.07952 | -6.50175 | 2.86E-08 | 1.89E-06 | 8.806369 |
| TERT | -2.87636 | 12.95346 | -6.49851 | 2.90E-08 | 1.90E-06 | 8.794752 |
| LAMB1 | -1.61519 | 13.2062 | -6.49838 | 2.90E-08 | 1.90E-06 | 8.794299 |
| RPS5 | 1.133505 | 13.2325 | 6.49622 | 2.92E-08 | 1.91E-06 | 8.786533 |
| ECE1 | -1.24713 | 13.36509 | -6.496 | 2.93E-08 | 1.91E-06 | 8.785758 |
| MIXL1 | -2.17709 | 13.17421 | -6.49313 | 2.96E-08 | 1.92E-06 | 8.775426 |
| FAM151B | -1.25169 | 13.26926 | -6.49145 | 2.97E-08 | 1.93E-06 | 8.769397 |
| MT-ND6 | 1.034922 | 13.28123 | 6.489945 | 2.99E-08 | 1.94E-06 | 8.764011 |
| RPL36AP13 | 1.744918 | 13.7777 | 6.485142 | 3.05E-08 | 1.97E-06 | 8.746772 |
| COQ10A | 1.241473 | 13.26422 | 6.480572 | 3.10E-08 | 1.99E-06 | 8.730369 |
| OVGP1 | 1.456619 | 13.20491 | 6.480337 | 3.10E-08 | 1.99E-06 | 8.729524 |
| IGHJ5 | -2.97289 | 12.77253 | -6.47977 | 3.11E-08 | 1.99E-06 | 8.727504 |
| PRC1 | -1.44918 | 12.80338 | -6.47925 | 3.11E-08 | 1.99E-06 | 8.72563 |
| CAV1 | -2.76044 | 12.82378 | -6.47341 | 3.18E-08 | 2.03E-06 | 8.704654 |
| SLC7A5 | -1.66084 | 12.5806 | -6.47094 | 3.21E-08 | 2.04E-06 | 8.695818 |
| IGHV1-46 | -3.51906 | 12.43807 | -6.47038 | 3.22E-08 | 2.04E-06 | 8.693794 |
| STMN3 | 1.586196 | 13.01608 | 6.46979 | 3.22E-08 | 2.04E-06 | 8.691678 |
| SIL1 | -1.04445 | 13.3743 | -6.46767 | 3.25E-08 | 2.06E-06 | 8.684071 |
| HLA-DRA | 1.568752 | 13.09065 | 6.46343 | 3.30E-08 | 2.08E-06 | 8.668857 |
| AC022034.2 | 2.695372 | 13.05571 | 6.463042 | 3.30E-08 | 2.08E-06 | 8.667463 |
| AZIN2 | 1.545399 | 13.1922 | 6.457836 | 3.37E-08 | 2.12E-06 | 8.648784 |
| IGKV3-15 | -2.76766 | 12.88537 | -6.4572 | 3.38E-08 | 2.12E-06 | 8.646511 |
| RPL11P3 | -2.4983 | 13.15286 | -6.45399 | 3.42E-08 | 2.14E-06 | 8.634998 |
| C1QC | -2.61235 | 12.79291 | -6.44304 | 3.56E-08 | 2.22E-06 | 8.595725 |
| AC007325.1 | -3.09621 | 12.86184 | -6.43456 | 3.67E-08 | 2.28E-06 | 8.565288 |
| CD177 | -5.50596 | 11.05228 | -6.43256 | 3.70E-08 | 2.30E-06 | 8.558115 |
| GMNN | -1.15249 | 13.30895 | -6.43196 | 3.71E-08 | 2.30E-06 | 8.555985 |
| RPS21 | 1.075289 | 13.1973 | 6.428915 | 3.75E-08 | 2.32E-06 | 8.545048 |
| RPS4X | 1.22086 | 13.20396 | 6.427598 | 3.77E-08 | 2.32E-06 | 8.540325 |
| NT5DC2 | -2.07677 | 13.07593 | -6.42758 | 3.77E-08 | 2.32E-06 | 8.540254 |
| COL7A1 | -1.92686 | 13.18518 | -6.42452 | 3.81E-08 | 2.34E-06 | 8.529271 |
| BCL6 | -2.00026 | 13.08089 | -6.42321 | 3.83E-08 | 2.35E-06 | 8.524576 |
| GBA | -1.13852 | 13.2166 | -6.41905 | 3.89E-08 | 2.38E-06 | 8.509689 |
| MARCHF9 | 1.110866 | 13.29244 | 6.418806 | 3.89E-08 | 2.38E-06 | 8.508798 |
| PLEC | -1.12574 | 13.33083 | -6.41712 | 3.91E-08 | 2.39E-06 | 8.502755 |
| EIF3E | 1.104043 | 13.3035 | 6.410242 | 4.02E-08 | 2.44E-06 | 8.478093 |
| RGMA | -1.35385 | 13.29362 | -6.40407 | 4.11E-08 | 2.49E-06 | 8.455958 |
| H4C8 | -2.17417 | 13.25431 | -6.39768 | 4.21E-08 | 2.54E-06 | 8.433048 |
| IFITM3P6 | 1.681722 | 13.19218 | 6.397123 | 4.21E-08 | 2.54E-06 | 8.431067 |
| C1QTNF3 | 1.268973 | 13.24001 | 6.394849 | 4.25E-08 | 2.56E-06 | 8.422918 |
| APBA1 | 2.090348 | 12.91424 | 6.393485 | 4.27E-08 | 2.56E-06 | 8.418027 |
| CENPI | -1.33702 | 13.2295 | -6.39178 | 4.30E-08 | 2.57E-06 | 8.411919 |
| AL109918.1 | -2.90669 | 12.83668 | -6.38524 | 4.40E-08 | 2.63E-06 | 8.388486 |
| MIR1244-1 | 4.313355 | 11.29116 | 6.384525 | 4.41E-08 | 2.63E-06 | 8.385919 |
| H2BS1 | -1.65532 | 13.26803 | -6.3711 | 4.64E-08 | 2.76E-06 | 8.337825 |
| CCNB1IP1 | 1.032132 | 13.32999 | 6.368002 | 4.69E-08 | 2.78E-06 | 8.326717 |
| IGLV6-57 | -3.63249 | 12.47891 | -6.36796 | 4.69E-08 | 2.78E-06 | 8.326583 |
| H2AC18 | -2.42489 | 12.99098 | -6.36193 | 4.80E-08 | 2.83E-06 | 8.304973 |
| IGHV4-39 | -3.57479 | 12.50418 | -6.36053 | 4.82E-08 | 2.84E-06 | 8.299966 |
| CDK14 | -1.03729 | 13.41683 | -6.35856 | 4.86E-08 | 2.85E-06 | 8.292908 |
| HK3 | -1.70262 | 13.08892 | -6.34684 | 5.07E-08 | 2.97E-06 | 8.250904 |
| NTSR1 | -2.44476 | 12.88433 | -6.3462 | 5.09E-08 | 2.97E-06 | 8.24862 |
| SLCO2B1 | -1.90658 | 13.02538 | -6.34386 | 5.13E-08 | 2.98E-06 | 8.240252 |
| PHKA1 | -2.09687 | 13.16272 | -6.34361 | 5.13E-08 | 2.98E-06 | 8.239343 |
| RPS3AP36 | 1.302675 | 14.69667 | 6.342194 | 5.16E-08 | 2.99E-06 | 8.234279 |
| PSMC3IP | -1.17874 | 13.21944 | -6.34121 | 5.18E-08 | 3.00E-06 | 8.230766 |
| ZNF223 | 1.207372 | 13.29596 | 6.335824 | 5.28E-08 | 3.05E-06 | 8.211472 |
| RGS16 | -1.69779 | 12.30831 | -6.32418 | 5.52E-08 | 3.18E-06 | 8.169779 |
| NOG | 3.241979 | 12.29424 | 6.322993 | 5.54E-08 | 3.18E-06 | 8.165537 |
| FCAR | -1.80455 | 13.08696 | -6.32271 | 5.55E-08 | 3.18E-06 | 8.164509 |
| ZNF502 | 1.677157 | 13.21519 | 6.320708 | 5.59E-08 | 3.20E-06 | 8.157358 |
| TC2N | 1.992487 | 13.08492 | 6.315367 | 5.70E-08 | 3.25E-06 | 8.13824 |
| SH2B2 | -1.01172 | 13.40167 | -6.3153 | 5.70E-08 | 3.25E-06 | 8.138018 |
| ANXA2R | 1.330762 | 13.22385 | 6.313516 | 5.74E-08 | 3.27E-06 | 8.131614 |
| RPS28 | 1.13345 | 13.20122 | 6.312905 | 5.75E-08 | 3.27E-06 | 8.129429 |
| IGSF23 | 2.902414 | 11.98699 | 6.311599 | 5.78E-08 | 3.28E-06 | 8.124756 |
| SAMD15 | -1.99496 | 13.24068 | -6.30967 | 5.82E-08 | 3.29E-06 | 8.117846 |
| TEF | 1.539903 | 13.20851 | 6.307243 | 5.87E-08 | 3.31E-06 | 8.109168 |
| GPR183 | 1.742449 | 13.14834 | 6.306267 | 5.89E-08 | 3.32E-06 | 8.105674 |
| NR1D1 | 1.377528 | 13.25358 | 6.304747 | 5.92E-08 | 3.32E-06 | 8.100235 |
| PLXNB3 | -2.992 | 12.49475 | -6.30446 | 5.93E-08 | 3.32E-06 | 8.099225 |
| IGLV2-23 | -2.95226 | 12.48171 | -6.2995 | 6.04E-08 | 3.37E-06 | 8.081474 |
| MXRA8 | 1.377998 | 13.118 | 6.299419 | 6.04E-08 | 3.37E-06 | 8.081169 |
| CCDC150 | -1.13161 | 13.24065 | -6.29876 | 6.06E-08 | 3.37E-06 | 8.078796 |
| CADM1 | -2.01864 | 13.09787 | -6.29726 | 6.09E-08 | 3.38E-06 | 8.073443 |
| HLA-DMB | 1.49654 | 13.12848 | 6.28839 | 6.29E-08 | 3.49E-06 | 8.041712 |
| LRRC75A | 1.059145 | 13.04608 | 6.281455 | 6.46E-08 | 3.56E-06 | 8.016905 |
| IGHV3-15 | -3.52245 | 12.17245 | -6.27897 | 6.52E-08 | 3.59E-06 | 8.008015 |
| IGHV2-26 | -3.43699 | 12.55905 | -6.27753 | 6.55E-08 | 3.59E-06 | 8.002873 |
| GLDC | -3.09557 | 12.63696 | -6.27744 | 6.55E-08 | 3.59E-06 | 8.00255 |
| TRAV1-2 | 2.752689 | 12.7567 | 6.267971 | 6.78E-08 | 3.70E-06 | 7.968684 |
| H2AC20 | -1.74551 | 13.09132 | -6.25787 | 7.04E-08 | 3.84E-06 | 7.932573 |
| IFI27 | -4.98361 | 12.24677 | -6.25449 | 7.13E-08 | 3.87E-06 | 7.920499 |
| CLIC5 | 1.880172 | 13.14069 | 6.25363 | 7.15E-08 | 3.87E-06 | 7.917413 |
| MYBPC3 | -1.75834 | 13.27687 | -6.24894 | 7.28E-08 | 3.93E-06 | 7.900663 |
| LPO | -1.84173 | 13.04504 | -6.24181 | 7.47E-08 | 4.02E-06 | 7.875154 |
| SEMA4A | -1.34375 | 13.31304 | -6.24145 | 7.48E-08 | 4.02E-06 | 7.873889 |
| CXCR6 | 2.173955 | 12.99188 | 6.226142 | 7.91E-08 | 4.25E-06 | 7.819178 |
| IGLV3-27 | -4.02978 | 11.96672 | -6.22401 | 7.98E-08 | 4.27E-06 | 7.811575 |
| AL671986.1 | -2.1508 | 13.0105 | -6.22255 | 8.02E-08 | 4.28E-06 | 7.806354 |
| MTARC1 | -1.62857 | 13.26803 | -6.22022 | 8.09E-08 | 4.31E-06 | 7.798035 |
| ARHGEF11 | -1.02792 | 13.35696 | -6.21528 | 8.24E-08 | 4.38E-06 | 7.780393 |
| ARPP21 | 1.883156 | 13.31129 | 6.209991 | 8.40E-08 | 4.45E-06 | 7.76149 |
| HLA-DPA1 | 1.516371 | 13.14378 | 6.209577 | 8.41E-08 | 4.45E-06 | 7.760012 |
| FOLR2 | 1.70978 | 12.87943 | 6.204179 | 8.58E-08 | 4.54E-06 | 7.740732 |
| IGHV5-51 | -3.03115 | 12.67091 | -6.20337 | 8.61E-08 | 4.54E-06 | 7.737834 |
| IGHJ4 | -2.79032 | 12.79 | -6.19252 | 8.96E-08 | 4.71E-06 | 7.699105 |
| IGIP | 1.421657 | 13.25499 | 6.186478 | 9.16E-08 | 4.80E-06 | 7.677539 |
| DHFR | -1.08929 | 13.21951 | -6.18313 | 9.27E-08 | 4.84E-06 | 7.66558 |
| LMAN1L | -2.20937 | 13.14987 | -6.17697 | 9.48E-08 | 4.94E-06 | 7.643586 |
| SLC25A26 | 1.033449 | 13.33135 | 6.175342 | 9.54E-08 | 4.96E-06 | 7.637791 |
| MGAM2 | -2.84345 | 12.85857 | -6.17308 | 9.62E-08 | 4.99E-06 | 7.629737 |
| EEF1A1P14 | 1.991818 | 13.15867 | 6.1729 | 9.63E-08 | 4.99E-06 | 7.62908 |
| TRAV12-1 | 2.032797 | 12.90823 | 6.172245 | 9.65E-08 | 5.00E-06 | 7.626743 |
| RPL12P6 | 1.66441 | 14.32337 | 6.170159 | 9.72E-08 | 5.03E-06 | 7.619296 |
| PLXDC1 | 2.003248 | 12.88273 | 6.166098 | 9.87E-08 | 5.09E-06 | 7.604808 |
| CLRN1 | -2.87204 | 12.81975 | -6.1653 | 9.90E-08 | 5.10E-06 | 7.601955 |
| PLIN3 | -1.05932 | 13.3183 | -6.16037 | 1.01E-07 | 5.17E-06 | 7.584391 |
| AC068946.2 | -2.99695 | 12.38243 | -6.15783 | 1.02E-07 | 5.21E-06 | 7.575303 |
| TRBV6-4 | 2.984489 | 12.62685 | 6.148466 | 1.05E-07 | 5.38E-06 | 7.541914 |
| HPDL | -2.32658 | 13.08724 | -6.14498 | 1.07E-07 | 5.44E-06 | 7.529481 |
| IGHV3-33 | -3.5512 | 12.51 | -6.14413 | 1.07E-07 | 5.45E-06 | 7.526436 |
| PGD | -1.39828 | 13.14152 | -6.14121 | 1.08E-07 | 5.49E-06 | 7.516047 |
| FCGR1A | -1.90456 | 13.20826 | -6.13597 | 1.10E-07 | 5.58E-06 | 7.497342 |
| BAMBI | -2.62873 | 12.96067 | -6.13093 | 1.12E-07 | 5.68E-06 | 7.479371 |
| NTN4 | 2.844787 | 12.69812 | 6.129923 | 1.13E-07 | 5.69E-06 | 7.475797 |
| RAD51AP1 | -1.24476 | 13.13084 | -6.12056 | 1.17E-07 | 5.88E-06 | 7.442437 |
| CETP | -1.70214 | 13.22563 | -6.11991 | 1.17E-07 | 5.88E-06 | 7.440104 |
| CENPU | -1.32002 | 13.1797 | -6.11675 | 1.18E-07 | 5.94E-06 | 7.428856 |
| CREBL2 | 1.088048 | 13.27049 | 6.115229 | 1.19E-07 | 5.97E-06 | 7.423427 |
| H2BC13 | -2.80414 | 12.69533 | -6.11366 | 1.20E-07 | 5.99E-06 | 7.417831 |
| CYSLTR2 | 2.230477 | 12.74044 | 6.111487 | 1.21E-07 | 6.03E-06 | 7.410093 |
| MT-CYB | 1.177573 | 13.19273 | 6.109775 | 1.21E-07 | 6.06E-06 | 7.403994 |
| H2AC19 | -2.69898 | 13.09942 | -6.10423 | 1.24E-07 | 6.17E-06 | 7.384243 |
| AC084871.1 | -2.66166 | 13.00388 | -6.10116 | 1.25E-07 | 6.23E-06 | 7.373302 |
| IGKV6-21 | -2.53128 | 12.55835 | -6.09936 | 1.26E-07 | 6.26E-06 | 7.366904 |
| FABP5P7 | -1.40085 | 13.31586 | -6.0979 | 1.27E-07 | 6.28E-06 | 7.361677 |
| IGKV3-20 | -2.67039 | 12.88537 | -6.09776 | 1.27E-07 | 6.28E-06 | 7.361196 |
| IGKC | -2.41349 | 12.87485 | -6.08422 | 1.33E-07 | 6.58E-06 | 7.312984 |
| TPPP3 | 2.964336 | 12.11715 | 6.080689 | 1.35E-07 | 6.65E-06 | 7.300399 |
| DDN | -2.83387 | 13.02459 | -6.08015 | 1.35E-07 | 6.65E-06 | 7.298497 |
| LY6G6D | -3.11651 | 12.67335 | -6.0776 | 1.37E-07 | 6.70E-06 | 7.289405 |
| IGKV1-33 | -3.1031 | 12.6599 | -6.07173 | 1.40E-07 | 6.84E-06 | 7.268501 |
| CRIP2 | 2.269272 | 12.44924 | 6.06955 | 1.41E-07 | 6.88E-06 | 7.260747 |
| RPL3P3 | 1.531759 | 14.29554 | 6.064817 | 1.43E-07 | 6.98E-06 | 7.243904 |
| SEZ6L | 3.763543 | 11.79332 | 6.064055 | 1.44E-07 | 6.98E-06 | 7.24119 |
| TECPR2 | -1.37315 | 13.3456 | -6.06396 | 1.44E-07 | 6.98E-06 | 7.240845 |
| IGF1 | -2.58805 | 12.93064 | -6.0614 | 1.45E-07 | 7.04E-06 | 7.231736 |
| PARPBP | -1.12493 | 13.2402 | -6.05468 | 1.49E-07 | 7.20E-06 | 7.207836 |
| SAPCD2 | -1.83844 | 13.05812 | -6.05121 | 1.50E-07 | 7.25E-06 | 7.195493 |
| RPS4XP20 | 1.123973 | 14.72342 | 6.051118 | 1.51E-07 | 7.25E-06 | 7.19516 |
| SIGLEC11 | -2.37364 | 13.03559 | -6.05039 | 1.51E-07 | 7.26E-06 | 7.192558 |
| TMEM38A | -1.27066 | 13.32869 | -6.04942 | 1.51E-07 | 7.27E-06 | 7.189127 |
| ZNF32 | 1.337903 | 13.26965 | 6.046253 | 1.53E-07 | 7.33E-06 | 7.177853 |
| ACSL1 | -1.76771 | 13.25534 | -6.04332 | 1.55E-07 | 7.40E-06 | 7.167404 |
| H4C11 | -1.72469 | 13.24533 | -6.03847 | 1.58E-07 | 7.52E-06 | 7.150161 |
| ZNF529 | 1.226939 | 13.27936 | 6.035302 | 1.60E-07 | 7.60E-06 | 7.138906 |
| PGAP3 | 1.035713 | 13.22907 | 6.034572 | 1.60E-07 | 7.60E-06 | 7.136309 |
| PER3 | 1.52579 | 13.23259 | 6.02953 | 1.63E-07 | 7.73E-06 | 7.118379 |
| AJAP1 | 2.372394 | 12.73261 | 6.027917 | 1.64E-07 | 7.75E-06 | 7.112646 |
| SBNO2 | -1.32228 | 13.31342 | -6.02432 | 1.66E-07 | 7.84E-06 | 7.099853 |
| SCART1 | 1.408019 | 13.19541 | 6.024174 | 1.66E-07 | 7.84E-06 | 7.099339 |
| PID1 | 3.538993 | 11.9927 | 6.015711 | 1.71E-07 | 8.07E-06 | 7.069259 |
| ABCB9 | -1.52717 | 13.23474 | -6.01323 | 1.73E-07 | 8.13E-06 | 7.06043 |
| ITGA8 | -2.54421 | 12.80745 | -6.01246 | 1.73E-07 | 8.13E-06 | 7.057714 |
| TENT5B | 2.63043 | 12.23233 | 6.011191 | 1.74E-07 | 8.16E-06 | 7.053194 |
| ST3GAL2 | -1.03791 | 13.3571 | -6.00642 | 1.77E-07 | 8.29E-06 | 7.036241 |
| FCGR1CP | -2.2145 | 13.1785 | -6.00287 | 1.80E-07 | 8.38E-06 | 7.023634 |
| ADAM9 | -1.3564 | 13.28596 | -6.00251 | 1.80E-07 | 8.38E-06 | 7.022336 |
| NMRAL2P | -2.87371 | 12.82385 | -5.99882 | 1.82E-07 | 8.48E-06 | 7.009227 |
| ADAMTS5 | 3.750107 | 11.62084 | 5.98899 | 1.89E-07 | 8.72E-06 | 6.974325 |
| H2AC15 | -2.07727 | 13.06293 | -5.98745 | 1.90E-07 | 8.76E-06 | 6.968871 |
| HRH2 | -1.74968 | 13.25193 | -5.98585 | 1.91E-07 | 8.80E-06 | 6.963189 |
| JCHAIN | -2.43889 | 12.94158 | -5.97804 | 1.97E-07 | 9.04E-06 | 6.935453 |
| C2orf15 | 1.278803 | 13.2437 | 5.977707 | 1.97E-07 | 9.04E-06 | 6.934262 |
| HROB | -2.02075 | 12.9613 | -5.9769 | 1.98E-07 | 9.04E-06 | 6.93141 |
| FO393411.1 | 2.064786 | 13.43183 | 5.975134 | 1.99E-07 | 9.08E-06 | 6.925127 |
| PADI4 | -1.7044 | 13.01996 | -5.96672 | 2.05E-07 | 9.35E-06 | 6.89525 |
| IGHV4-34 | -3.58087 | 12.23525 | -5.96365 | 2.07E-07 | 9.44E-06 | 6.884368 |
| AC131392.2 | -2.68133 | 12.91193 | -5.9628 | 2.08E-07 | 9.46E-06 | 6.881342 |
| AK1 | 1.998606 | 11.9127 | 5.960935 | 2.09E-07 | 9.49E-06 | 6.874732 |
| IGLC1 | -2.81635 | 12.75643 | -5.95955 | 2.11E-07 | 9.53E-06 | 6.86981 |
| LY6G6F-LY6G6D | -2.49433 | 12.90894 | -5.95236 | 2.16E-07 | 9.77E-06 | 6.844323 |
| AC026403.1 | 1.061055 | 13.19713 | 5.949689 | 2.18E-07 | 9.85E-06 | 6.834833 |
| IGLV1-44 | -3.19988 | 12.63053 | -5.94305 | 2.24E-07 | 1.01E-05 | 6.811286 |
| DCANP1 | 4.011186 | 11.98246 | 5.941932 | 2.25E-07 | 1.01E-05 | 6.807321 |
| HLF | 2.01783 | 13.01228 | 5.939877 | 2.26E-07 | 1.01E-05 | 6.800032 |
| SH2D1A | 1.908849 | 13.03764 | 5.934435 | 2.31E-07 | 1.03E-05 | 6.780736 |
| DHCR24 | -1.56452 | 13.35442 | -5.93389 | 2.31E-07 | 1.03E-05 | 6.778788 |
| SNHG32 | 1.0592 | 13.29962 | 5.933143 | 2.32E-07 | 1.03E-05 | 6.776155 |
| PLEKHH3 | -1.55492 | 12.43841 | -5.93013 | 2.34E-07 | 1.04E-05 | 6.765468 |
| XIRP2 | -2.75614 | 12.82652 | -5.9286 | 2.36E-07 | 1.05E-05 | 6.760043 |
| TMC5 | 2.222226 | 12.22006 | 5.927557 | 2.37E-07 | 1.05E-05 | 6.756354 |
| GPR27 | -1.41719 | 13.38187 | -5.92678 | 2.37E-07 | 1.05E-05 | 6.753585 |
| SPON1 | 2.370664 | 12.87919 | 5.917439 | 2.46E-07 | 1.08E-05 | 6.72049 |
| DOK3 | -1.36467 | 13.28841 | -5.91518 | 2.48E-07 | 1.09E-05 | 6.712485 |
| CDC14A | 1.192469 | 13.29845 | 5.911914 | 2.51E-07 | 1.10E-05 | 6.700914 |
| AL133517.1 | -1.98807 | 13.21758 | -5.91157 | 2.51E-07 | 1.10E-05 | 6.69968 |
| FAAHP1 | 1.774233 | 13.06925 | 5.906548 | 2.56E-07 | 1.12E-05 | 6.681903 |
| RNF144A | 1.234952 | 13.18174 | 5.906113 | 2.56E-07 | 1.12E-05 | 6.680361 |
| RPL41P2 | 2.588528 | 12.87604 | 5.897792 | 2.64E-07 | 1.15E-05 | 6.650888 |
| PIR | -2.5354 | 12.51193 | -5.89164 | 2.70E-07 | 1.17E-05 | 6.629089 |
| RCAN3 | 1.682057 | 13.1399 | 5.891033 | 2.70E-07 | 1.17E-05 | 6.626955 |
| C12orf57 | 1.25506 | 13.17082 | 5.886427 | 2.75E-07 | 1.19E-05 | 6.610647 |
| DGCR6 | 1.054354 | 13.20537 | 5.885297 | 2.76E-07 | 1.20E-05 | 6.606647 |
| SPATC1 | -3.14276 | 12.69268 | -5.88467 | 2.77E-07 | 1.20E-05 | 6.604429 |
| CCDC88B | -1.01818 | 13.34978 | -5.88337 | 2.78E-07 | 1.20E-05 | 6.599808 |
| CD52 | 1.180257 | 13.1692 | 5.873053 | 2.89E-07 | 1.24E-05 | 6.563311 |
| CLEC10A | 2.40789 | 12.55826 | 5.872938 | 2.89E-07 | 1.24E-05 | 6.562902 |
| SRPK1 | -1.27699 | 13.27654 | -5.87291 | 2.89E-07 | 1.24E-05 | 6.562815 |
| TMEM267 | 1.150145 | 13.38559 | 5.87169 | 2.90E-07 | 1.25E-05 | 6.558488 |
| GATA6 | 2.685092 | 12.60496 | 5.869546 | 2.93E-07 | 1.25E-05 | 6.550899 |
| AC116353.6 | -1.46571 | 13.25439 | -5.86657 | 2.96E-07 | 1.26E-05 | 6.540368 |
| CD38 | -1.61628 | 13.27885 | -5.86575 | 2.97E-07 | 1.27E-05 | 6.537452 |
| GPR141 | -1.55928 | 13.24872 | -5.86425 | 2.98E-07 | 1.27E-05 | 6.532178 |
| NSG1 | 2.224229 | 13.01469 | 5.861882 | 3.01E-07 | 1.28E-05 | 6.523784 |
| FAHD2CP | 1.332944 | 13.22542 | 5.860682 | 3.02E-07 | 1.28E-05 | 6.519541 |
| GP1BA | -1.77879 | 13.09159 | -5.85923 | 3.04E-07 | 1.29E-05 | 6.514407 |
| AC087343.1 | 2.188034 | 12.95007 | 5.856422 | 3.07E-07 | 1.30E-05 | 6.504474 |
| FAM124A | -1.37981 | 13.38064 | -5.85048 | 3.14E-07 | 1.33E-05 | 6.483445 |
| NDC80 | -1.00316 | 13.21681 | -5.84557 | 3.19E-07 | 1.35E-05 | 6.466082 |
| SLC7A6 | 1.370146 | 13.16213 | 5.844389 | 3.21E-07 | 1.35E-05 | 6.461924 |
| PTTG1 | -1.45515 | 12.8194 | -5.84404 | 3.21E-07 | 1.35E-05 | 6.460701 |
| TUBG1 | -1.21402 | 12.83207 | -5.84221 | 3.23E-07 | 1.36E-05 | 6.454205 |
| MS4A4A | -1.99287 | 13.05285 | -5.84028 | 3.26E-07 | 1.36E-05 | 6.447398 |
| OSGEPL1 | 1.146361 | 13.32351 | 5.839066 | 3.27E-07 | 1.37E-05 | 6.443107 |
| ZNF10 | 1.223075 | 13.33117 | 5.83427 | 3.33E-07 | 1.39E-05 | 6.426158 |
| KIT | 1.843634 | 13.03895 | 5.832983 | 3.34E-07 | 1.39E-05 | 6.42161 |
| UNC13B | -1.61669 | 13.23097 | -5.83159 | 3.36E-07 | 1.40E-05 | 6.416682 |
| DKK3 | 2.512576 | 12.81011 | 5.830543 | 3.37E-07 | 1.40E-05 | 6.412987 |
| IL24 | 1.691993 | 13.20327 | 5.830433 | 3.37E-07 | 1.40E-05 | 6.412597 |
| SELP | -1.81074 | 12.9848 | -5.82957 | 3.39E-07 | 1.40E-05 | 6.409565 |
| C3AR1 | -1.95076 | 13.17165 | -5.82872 | 3.40E-07 | 1.40E-05 | 6.40653 |
| PROCR | 1.607938 | 13.22503 | 5.828166 | 3.40E-07 | 1.40E-05 | 6.404588 |
| ZBTB32 | -1.73575 | 13.24214 | -5.82657 | 3.42E-07 | 1.41E-05 | 6.398942 |
| HLA-DMA | 1.139931 | 13.21722 | 5.82583 | 3.43E-07 | 1.41E-05 | 6.396332 |
| OLFM2 | 1.69997 | 13.03758 | 5.82559 | 3.43E-07 | 1.41E-05 | 6.395484 |
| HNRNPUL2-BSCL2 | -1.12251 | 13.48528 | -5.82364 | 3.46E-07 | 1.42E-05 | 6.388589 |
| HBA1 | 3.513763 | 11.42561 | 5.823496 | 3.46E-07 | 1.42E-05 | 6.388086 |
| TRBC2 | 1.911942 | 12.99494 | 5.822778 | 3.47E-07 | 1.42E-05 | 6.385552 |
| S100A8 | -1.90258 | 12.87683 | -5.82191 | 3.48E-07 | 1.42E-05 | 6.382501 |
| IPO5P1 | 1.252308 | 13.28139 | 5.817514 | 3.54E-07 | 1.44E-05 | 6.366956 |
| RPL4P1 | 2.351375 | 12.81208 | 5.816849 | 3.55E-07 | 1.44E-05 | 6.364609 |
| TG | -1.77781 | 13.28637 | -5.80844 | 3.66E-07 | 1.48E-05 | 6.334896 |
| NUDT13 | 1.11751 | 13.2817 | 5.808233 | 3.66E-07 | 1.48E-05 | 6.334179 |
| IQGAP3 | -2.27668 | 12.06035 | -5.80572 | 3.69E-07 | 1.49E-05 | 6.325296 |
| ATF7IP2 | 1.131555 | 13.28938 | 5.805517 | 3.70E-07 | 1.49E-05 | 6.324591 |
| SCML1 | 1.567846 | 13.22184 | 5.804251 | 3.71E-07 | 1.49E-05 | 6.32012 |
| IGHV3-11 | -3.56351 | 12.49479 | -5.80414 | 3.71E-07 | 1.49E-05 | 6.319731 |
| SLC1A4 | -1.25318 | 13.25602 | -5.80086 | 3.76E-07 | 1.51E-05 | 6.308142 |
| IGHV3-21 | -3.80128 | 12.60547 | -5.7985 | 3.79E-07 | 1.52E-05 | 6.299805 |
| TASP1 | 1.094111 | 13.35011 | 5.791367 | 3.89E-07 | 1.56E-05 | 6.274644 |
| ZBTB46 | 1.349818 | 12.98082 | 5.786537 | 3.96E-07 | 1.58E-05 | 6.257602 |
| RALGAPA2 | -1.28609 | 13.30338 | -5.78635 | 3.96E-07 | 1.58E-05 | 6.256946 |
| MT-ATP6 | 1.053558 | 13.27898 | 5.784712 | 3.99E-07 | 1.59E-05 | 6.251166 |
| AC104619.3 | 2.188228 | 13.09193 | 5.781978 | 4.03E-07 | 1.60E-05 | 6.241519 |
| CA12 | -3.16174 | 12.88752 | -5.77758 | 4.09E-07 | 1.62E-05 | 6.225995 |
| RASA4 | 1.259593 | 13.17162 | 5.771062 | 4.19E-07 | 1.66E-05 | 6.20302 |
| PAIP2B | 1.223422 | 13.32835 | 5.769036 | 4.22E-07 | 1.67E-05 | 6.195874 |
| ALDH1A2 | -1.72048 | 13.32719 | -5.76487 | 4.28E-07 | 1.69E-05 | 6.181202 |
| IGLV3-19 | -3.85267 | 11.95972 | -5.75989 | 4.36E-07 | 1.72E-05 | 6.163617 |
| HAUS1P1 | -2.84188 | 12.96816 | -5.75896 | 4.38E-07 | 1.73E-05 | 6.160347 |
| NLRC4 | -1.20634 | 13.21529 | -5.75544 | 4.43E-07 | 1.75E-05 | 6.147964 |
| IGLJ3 | -2.54404 | 12.96854 | -5.75218 | 4.49E-07 | 1.76E-05 | 6.136474 |
| ZNF582 | 1.083659 | 13.34002 | 5.751727 | 4.49E-07 | 1.76E-05 | 6.134862 |
| RPSAP8 | 1.990106 | 13.69585 | 5.750594 | 4.51E-07 | 1.77E-05 | 6.130872 |
| CENPN | -1.02339 | 13.27497 | -5.75048 | 4.52E-07 | 1.77E-05 | 6.130462 |
| FGFR2 | 2.62673 | 12.46322 | 5.746216 | 4.59E-07 | 1.79E-05 | 6.115445 |
| AC079250.1 | 1.8174 | 13.18696 | 5.744169 | 4.62E-07 | 1.80E-05 | 6.108233 |
| FAM102A | 1.671331 | 13.07349 | 5.736385 | 4.75E-07 | 1.84E-05 | 6.080819 |
| AK5 | 2.103274 | 12.91403 | 5.736372 | 4.75E-07 | 1.84E-05 | 6.080774 |
| EID3 | 1.431625 | 13.22558 | 5.729374 | 4.88E-07 | 1.87E-05 | 6.056131 |
| QSOX1 | -1.19655 | 13.18813 | -5.72906 | 4.88E-07 | 1.87E-05 | 6.055028 |
| RPL10P3 | 2.07018 | 13.48855 | 5.728981 | 4.88E-07 | 1.87E-05 | 6.054745 |
| WDFY3 | -1.3993 | 13.33861 | -5.72506 | 4.95E-07 | 1.90E-05 | 6.040939 |
| TDRD9 | -1.93375 | 13.0544 | -5.72484 | 4.96E-07 | 1.90E-05 | 6.040166 |
| ASS1P1 | -2.16085 | 13.08524 | -5.72365 | 4.98E-07 | 1.90E-05 | 6.035962 |
| H2BC21 | -1.56922 | 13.27068 | -5.7194 | 5.06E-07 | 1.93E-05 | 6.021005 |
| GAS6 | -1.50653 | 13.25594 | -5.71349 | 5.17E-07 | 1.97E-05 | 6.000239 |
| AC246787.1 | 1.644752 | 13.35788 | 5.709262 | 5.25E-07 | 1.99E-05 | 5.985347 |
| AC246787.3 | 1.644752 | 13.35788 | 5.709262 | 5.25E-07 | 1.99E-05 | 5.985347 |
| ACSM3 | 1.039921 | 12.99429 | 5.707813 | 5.27E-07 | 2.00E-05 | 5.98025 |
| PPP1R2P6 | -2.29965 | 13.3503 | -5.70724 | 5.28E-07 | 2.00E-05 | 5.978221 |
| PRSS22 | 2.643736 | 12.85338 | 5.706938 | 5.29E-07 | 2.00E-05 | 5.977173 |
| IL23R | 2.673481 | 12.88087 | 5.70017 | 5.42E-07 | 2.05E-05 | 5.953367 |
| RTN1 | 1.793913 | 12.69324 | 5.697803 | 5.47E-07 | 2.06E-05 | 5.945041 |
| IL34 | 2.616819 | 12.10031 | 5.695719 | 5.51E-07 | 2.07E-05 | 5.937713 |
| SPEF2 | 1.480297 | 13.16614 | 5.695241 | 5.52E-07 | 2.07E-05 | 5.936033 |
| IGKV1-5 | -2.62668 | 12.76212 | -5.69378 | 5.55E-07 | 2.08E-05 | 5.930914 |
| IFITM3 | -2.41127 | 13.06114 | -5.6923 | 5.58E-07 | 2.09E-05 | 5.925701 |
| NELL2 | 2.718266 | 12.71559 | 5.692023 | 5.58E-07 | 2.09E-05 | 5.924719 |
| ANKRD22 | -2.18966 | 13.04052 | -5.69175 | 5.59E-07 | 2.09E-05 | 5.923772 |
| IGLV9-49 | -2.93282 | 12.70683 | -5.6894 | 5.64E-07 | 2.10E-05 | 5.915499 |
| CRY1 | 1.026274 | 13.3386 | 5.689114 | 5.64E-07 | 2.10E-05 | 5.914493 |
| PMP22 | 3.030782 | 12.13863 | 5.684462 | 5.74E-07 | 2.14E-05 | 5.89814 |
| ASIC4 | -1.97744 | 13.21957 | -5.68403 | 5.75E-07 | 2.14E-05 | 5.896635 |
| IGLV8-61 | -2.29157 | 12.78026 | -5.68024 | 5.83E-07 | 2.16E-05 | 5.88331 |
| OXCT2 | -3.26476 | 12.71913 | -5.68017 | 5.83E-07 | 2.16E-05 | 5.883044 |
| RECQL4 | -1.49732 | 13.00664 | -5.67818 | 5.87E-07 | 2.17E-05 | 5.87608 |
| AC139495.1 | -2.75328 | 12.81735 | -5.67687 | 5.90E-07 | 2.18E-05 | 5.871469 |
| NOP53 | 1.100648 | 13.20583 | 5.674726 | 5.95E-07 | 2.19E-05 | 5.86393 |
| NEK6 | -1.1399 | 13.2877 | -5.67232 | 6.00E-07 | 2.21E-05 | 5.855472 |
| ANO2 | -2.30954 | 12.90526 | -5.67225 | 6.00E-07 | 2.21E-05 | 5.855245 |
| BAG1 | 1.763683 | 12.09207 | 5.671187 | 6.02E-07 | 2.21E-05 | 5.851496 |
| FNDC3B | -1.03133 | 13.46243 | -5.66919 | 6.07E-07 | 2.23E-05 | 5.84449 |
| BMX | -2.4118 | 12.78693 | -5.66769 | 6.10E-07 | 2.23E-05 | 5.839206 |
| NLRP4 | 2.286802 | 12.97387 | 5.667634 | 6.10E-07 | 2.23E-05 | 5.839017 |
| DBP | 1.01047 | 13.18998 | 5.66391 | 6.18E-07 | 2.26E-05 | 5.825936 |
| NMT2 | 1.644783 | 13.16194 | 5.663205 | 6.20E-07 | 2.26E-05 | 5.823461 |
| TMEM117 | 1.446926 | 13.29645 | 5.658122 | 6.32E-07 | 2.30E-05 | 5.805613 |
| PLK4 | -1.20476 | 13.18925 | -5.65217 | 6.45E-07 | 2.35E-05 | 5.784716 |
| ADGRA3 | 1.348027 | 13.24864 | 5.651046 | 6.48E-07 | 2.35E-05 | 5.780774 |
| ARL14EP | 1.16035 | 13.33865 | 5.6482 | 6.55E-07 | 2.38E-05 | 5.770785 |
| PASK | 1.366222 | 13.19473 | 5.645814 | 6.60E-07 | 2.39E-05 | 5.76241 |
| DDIAS | -1.12163 | 13.30609 | -5.64365 | 6.66E-07 | 2.41E-05 | 5.754815 |
| IGLL5 | -2.5062 | 12.80768 | -5.64329 | 6.66E-07 | 2.41E-05 | 5.753556 |
| AL031432.2 | -2.6088 | 12.61231 | -5.64231 | 6.69E-07 | 2.41E-05 | 5.750133 |
| IGHEP2 | -1.81299 | 13.25532 | -5.63916 | 6.76E-07 | 2.44E-05 | 5.739074 |
| GABRR2 | -2.27085 | 12.98675 | -5.6335 | 6.90E-07 | 2.49E-05 | 5.71922 |
| CCDC65 | 1.344099 | 13.26102 | 5.633109 | 6.91E-07 | 2.49E-05 | 5.717837 |
| AL078604.1 | 1.967979 | 13.33857 | 5.632868 | 6.92E-07 | 2.49E-05 | 5.716993 |
| ZNF662 | 1.453248 | 13.22045 | 5.631193 | 6.96E-07 | 2.50E-05 | 5.711118 |
| SYTL5 | 1.427383 | 12.96411 | 5.629396 | 7.01E-07 | 2.51E-05 | 5.704818 |
| SRPX | 2.326935 | 12.95081 | 5.627579 | 7.05E-07 | 2.53E-05 | 5.698444 |
| CLYBL | 1.188978 | 13.26113 | 5.626366 | 7.09E-07 | 2.53E-05 | 5.69419 |
| MCM2 | -1.23855 | 13.17265 | -5.62226 | 7.19E-07 | 2.57E-05 | 5.679788 |
| MTND5P32 | -2.41602 | 13.21331 | -5.62 | 7.25E-07 | 2.58E-05 | 5.671865 |
| ANO9 | 1.481642 | 12.99773 | 5.618072 | 7.30E-07 | 2.59E-05 | 5.665113 |
| CA4 | -2.4326 | 12.74956 | -5.61716 | 7.33E-07 | 2.60E-05 | 5.661914 |
| FXYD7 | 1.494943 | 13.01181 | 5.613914 | 7.41E-07 | 2.62E-05 | 5.65054 |
| IGHG3 | -3.52357 | 11.73033 | -5.61272 | 7.44E-07 | 2.63E-05 | 5.646366 |
| SOAT2 | 2.032456 | 13.09974 | 5.610541 | 7.50E-07 | 2.65E-05 | 5.63872 |
| WNK4 | -1.8003 | 13.32893 | -5.6092 | 7.54E-07 | 2.66E-05 | 5.634025 |
| IGF2BP3 | -1.32405 | 13.36589 | -5.60825 | 7.57E-07 | 2.66E-05 | 5.630684 |
| KLHL32 | 1.743095 | 13.2473 | 5.605941 | 7.63E-07 | 2.68E-05 | 5.622604 |
| SLC16A3 | -1.30146 | 13.21094 | -5.60228 | 7.73E-07 | 2.72E-05 | 5.609781 |
| DDX10P1 | -1.70579 | 13.32083 | -5.59953 | 7.81E-07 | 2.74E-05 | 5.60016 |
| LBH | 1.613106 | 13.1346 | 5.593936 | 7.97E-07 | 2.79E-05 | 5.580556 |
| IGKV1-6 | -2.28994 | 13.0792 | -5.59368 | 7.98E-07 | 2.79E-05 | 5.579676 |
| CTSF | 1.587171 | 13.12185 | 5.593446 | 7.98E-07 | 2.79E-05 | 5.578842 |
| AC025459.1 | 1.169278 | 14.75587 | 5.59336 | 7.99E-07 | 2.79E-05 | 5.578541 |
| DDAH2 | -1.35516 | 13.27263 | -5.59055 | 8.07E-07 | 2.81E-05 | 5.568717 |
| GPR17 | -2.51303 | 13.02844 | -5.58876 | 8.12E-07 | 2.83E-05 | 5.56244 |
| ZNF608 | -3.3816 | 12.30415 | -5.58747 | 8.16E-07 | 2.84E-05 | 5.557904 |
| CNGA4 | -2.20747 | 13.18001 | -5.58722 | 8.16E-07 | 2.84E-05 | 5.557033 |
| PLEKHA1 | 1.367224 | 13.29504 | 5.587206 | 8.16E-07 | 2.84E-05 | 5.556996 |
| ANKRD33B | -1.15325 | 13.26268 | -5.58374 | 8.27E-07 | 2.87E-05 | 5.544876 |
| OR2B6 | -2.75583 | 12.83088 | -5.57975 | 8.39E-07 | 2.91E-05 | 5.530908 |
| NT5E | 2.107518 | 13.0818 | 5.579615 | 8.39E-07 | 2.91E-05 | 5.530426 |
| ZNF181 | 1.115724 | 13.34887 | 5.577953 | 8.44E-07 | 2.92E-05 | 5.524609 |
| KLHL3 | 1.601897 | 13.14144 | 5.575722 | 8.51E-07 | 2.94E-05 | 5.516804 |
| ZNF438 | -1.22248 | 13.37774 | -5.57251 | 8.61E-07 | 2.96E-05 | 5.50557 |
| MYO10 | -1.53654 | 13.26463 | -5.57017 | 8.68E-07 | 2.99E-05 | 5.497391 |
| TUBB3 | -2.03386 | 13.16019 | -5.56956 | 8.70E-07 | 2.99E-05 | 5.495253 |
| HOXB13 | -1.74227 | 13.14623 | -5.56895 | 8.72E-07 | 2.99E-05 | 5.49312 |
| CERKL | 1.115677 | 13.27906 | 5.567617 | 8.76E-07 | 3.00E-05 | 5.488452 |
| EEF1B2P3 | 1.828578 | 13.00455 | 5.564503 | 8.86E-07 | 3.03E-05 | 5.477561 |
| MCTP2 | -1.2317 | 13.37577 | -5.56153 | 8.96E-07 | 3.05E-05 | 5.467176 |
| IL11RA | 1.328314 | 13.05837 | 5.559154 | 9.04E-07 | 3.07E-05 | 5.458858 |
| FANCI | -1.04401 | 13.17296 | -5.55715 | 9.10E-07 | 3.08E-05 | 5.451834 |
| NFIL3 | -1.30856 | 13.342 | -5.55711 | 9.10E-07 | 3.08E-05 | 5.451711 |
| SYBU | 1.478736 | 13.11908 | 5.553377 | 9.23E-07 | 3.12E-05 | 5.438661 |
| TMEM92 | -2.49341 | 12.48528 | -5.55224 | 9.27E-07 | 3.13E-05 | 5.434694 |
| DSCC1 | -1.35204 | 13.17321 | -5.55201 | 9.27E-07 | 3.13E-05 | 5.433873 |
| IGKV1-9 | -2.43692 | 12.61925 | -5.55189 | 9.28E-07 | 3.13E-05 | 5.433447 |
| TRBJ1-1 | 1.872435 | 12.98645 | 5.548673 | 9.39E-07 | 3.16E-05 | 5.422222 |
| TNFRSF25 | 1.730251 | 12.91115 | 5.546098 | 9.47E-07 | 3.18E-05 | 5.413222 |
| RPL19P21 | 1.479138 | 14.30771 | 5.545859 | 9.48E-07 | 3.18E-05 | 5.412386 |
| SMARCD3 | -1.32907 | 13.31224 | -5.54511 | 9.51E-07 | 3.19E-05 | 5.409758 |
| CYP2U1 | 1.597118 | 13.19623 | 5.543467 | 9.56E-07 | 3.20E-05 | 5.40403 |
| EPHX2 | 1.426986 | 13.15658 | 5.540357 | 9.67E-07 | 3.23E-05 | 5.393164 |
| IGHV4-61 | -2.77537 | 12.68637 | -5.53543 | 9.85E-07 | 3.27E-05 | 5.37596 |
| CLEC4F | 4.192063 | 11.03997 | 5.535133 | 9.86E-07 | 3.27E-05 | 5.374918 |
| BVES | -2.09175 | 13.12652 | -5.53507 | 9.86E-07 | 3.27E-05 | 5.374685 |
| IL4 | 2.917387 | 12.26827 | 5.532801 | 9.94E-07 | 3.29E-05 | 5.366773 |
| CCNF | -1.2443 | 12.98845 | -5.53005 | 1.00E-06 | 3.32E-05 | 5.357165 |
| SLC16A10 | 1.123665 | 13.3071 | 5.529011 | 1.01E-06 | 3.33E-05 | 5.353539 |
| CNIH3 | 1.088884 | 12.95886 | 5.528108 | 1.01E-06 | 3.33E-05 | 5.350386 |
| FCER1A | 4.65691 | 11.29917 | 5.527078 | 1.01E-06 | 3.34E-05 | 5.34679 |
| PLD4 | 2.094036 | 12.76023 | 5.52652 | 1.02E-06 | 3.34E-05 | 5.344842 |
| RPL23AP43 | 1.343802 | 14.31848 | 5.525164 | 1.02E-06 | 3.36E-05 | 5.340109 |
| IGLV10-54 | -3.06124 | 12.38177 | -5.5244 | 1.02E-06 | 3.36E-05 | 5.337434 |
| RPS27AP11 | 1.935495 | 13.36019 | 5.523421 | 1.03E-06 | 3.37E-05 | 5.334025 |
| DYRK2 | 1.650038 | 13.1883 | 5.517775 | 1.05E-06 | 3.43E-05 | 5.31432 |
| NBEAL2 | -1.21013 | 13.25196 | -5.51716 | 1.05E-06 | 3.43E-05 | 5.31218 |
| RPL21P92 | 1.026124 | 15.25061 | 5.515431 | 1.06E-06 | 3.45E-05 | 5.30614 |
| VSIG1 | 2.074016 | 12.96543 | 5.511587 | 1.07E-06 | 3.49E-05 | 5.292728 |
| WASF1 | -1.58948 | 13.23129 | -5.50728 | 1.09E-06 | 3.54E-05 | 5.277716 |
| AC006059.2 | -3.73878 | 12.14506 | -5.50652 | 1.09E-06 | 3.54E-05 | 5.275066 |
| XRCC2 | -1.03884 | 13.28447 | -5.5017 | 1.11E-06 | 3.59E-05 | 5.258241 |
| ZNF599 | 1.100483 | 13.32415 | 5.501313 | 1.11E-06 | 3.59E-05 | 5.256895 |
| AC139494.1 | -1.87257 | 13.13729 | -5.4993 | 1.12E-06 | 3.61E-05 | 5.249889 |
| TRAC | 1.923987 | 13.01439 | 5.499221 | 1.12E-06 | 3.61E-05 | 5.249599 |
| FREM3 | 1.781716 | 13.21828 | 5.498504 | 1.12E-06 | 3.62E-05 | 5.2471 |
| IL27 | -1.72515 | 13.32041 | -5.49518 | 1.14E-06 | 3.66E-05 | 5.235525 |
| PCP4L1 | -1.87502 | 13.26232 | -5.49454 | 1.14E-06 | 3.66E-05 | 5.233266 |
| TRAF1 | 1.159346 | 13.14757 | 5.492234 | 1.15E-06 | 3.68E-05 | 5.22524 |
| GPR173 | 1.328917 | 13.35987 | 5.490681 | 1.16E-06 | 3.69E-05 | 5.219828 |
| GP6 | -1.87128 | 12.96523 | -5.49067 | 1.16E-06 | 3.69E-05 | 5.219781 |
| CD1E | 3.057357 | 12.28678 | 5.489678 | 1.16E-06 | 3.70E-05 | 5.216331 |
| CHPF | -1.8808 | 13.13964 | -5.48709 | 1.17E-06 | 3.72E-05 | 5.207309 |
| ZDHHC4P1 | -3.24757 | 12.83163 | -5.47904 | 1.21E-06 | 3.83E-05 | 5.179265 |
| DUX4L50 | -1.34534 | 13.29205 | -5.47622 | 1.22E-06 | 3.86E-05 | 5.169451 |
| LRFN1 | -1.30558 | 13.31285 | -5.47491 | 1.22E-06 | 3.88E-05 | 5.164891 |
| OIT3 | -2.04729 | 13.14955 | -5.4746 | 1.23E-06 | 3.88E-05 | 5.163804 |
| SH3YL1 | 1.436401 | 13.21694 | 5.47058 | 1.24E-06 | 3.93E-05 | 5.149801 |
| SERPINE3 | -1.69497 | 13.28891 | -5.46794 | 1.26E-06 | 3.97E-05 | 5.140608 |
| RPS27AP16 | 1.769807 | 13.23619 | 5.467631 | 1.26E-06 | 3.97E-05 | 5.139533 |
| AC136632.1 | 1.93177 | 13.02507 | 5.467067 | 1.26E-06 | 3.97E-05 | 5.137569 |
| SGPP1 | 1.141207 | 13.33844 | 5.466286 | 1.26E-06 | 3.98E-05 | 5.13485 |
| GUSBP1 | 1.055627 | 13.2977 | 5.465131 | 1.27E-06 | 3.99E-05 | 5.130831 |
| CAMK2N1 | 1.920092 | 13.0504 | 5.460796 | 1.29E-06 | 4.04E-05 | 5.115742 |
| KRT5 | 3.81847 | 11.8204 | 5.456077 | 1.31E-06 | 4.10E-05 | 5.099321 |
| KLRG1 | 2.038218 | 12.97817 | 5.454523 | 1.32E-06 | 4.12E-05 | 5.093913 |
| AC022916.4 | 2.893569 | 12.68268 | 5.454259 | 1.32E-06 | 4.12E-05 | 5.092994 |
| HLA-DRB1 | 1.470345 | 12.99354 | 5.453873 | 1.32E-06 | 4.12E-05 | 5.091649 |
| RPL21P11 | 2.271985 | 13.15581 | 5.451779 | 1.33E-06 | 4.15E-05 | 5.084367 |
| PHF21A | -1.0403 | 13.34552 | -5.45086 | 1.34E-06 | 4.15E-05 | 5.081174 |
| DUSP2 | 1.580907 | 13.0333 | 5.449697 | 1.34E-06 | 4.16E-05 | 5.077123 |
| IGLV2-5 | -2.40153 | 12.77037 | -5.44759 | 1.35E-06 | 4.18E-05 | 5.069793 |
| IGKV1-27 | -2.75052 | 12.20918 | -5.44544 | 1.36E-06 | 4.21E-05 | 5.062308 |
| TRIML2 | -1.16062 | 13.36746 | -5.4434 | 1.37E-06 | 4.24E-05 | 5.055235 |
| S100A12 | -2.10472 | 12.76854 | -5.44191 | 1.38E-06 | 4.24E-05 | 5.050048 |
| TP73 | -1.74405 | 13.17528 | -5.44173 | 1.38E-06 | 4.24E-05 | 5.0494 |
| RPS3AP20 | 2.003032 | 12.88976 | 5.440022 | 1.39E-06 | 4.26E-05 | 5.043477 |
| TMEM8B | 1.155552 | 13.20518 | 5.438768 | 1.39E-06 | 4.27E-05 | 5.039115 |
| ADAM19 | -1.01829 | 13.44048 | -5.43512 | 1.41E-06 | 4.31E-05 | 5.02642 |
| ARL6 | 1.272471 | 13.32082 | 5.434325 | 1.42E-06 | 4.32E-05 | 5.023671 |
| IGKV1-17 | -2.51633 | 12.49385 | -5.4325 | 1.43E-06 | 4.34E-05 | 5.017319 |
| CDKN1A | -1.1235 | 13.30944 | -5.42586 | 1.46E-06 | 4.44E-05 | 4.994243 |
| ATP5F1AP8 | 1.494899 | 12.81053 | 5.424844 | 1.47E-06 | 4.44E-05 | 4.990726 |
| RPL3P9 | 1.534292 | 14.08449 | 5.424546 | 1.47E-06 | 4.44E-05 | 4.989688 |
| ZNF559 | 1.012137 | 13.36666 | 5.424308 | 1.47E-06 | 4.44E-05 | 4.988862 |
| ABCB6 | -1.20837 | 12.38986 | -5.42353 | 1.47E-06 | 4.45E-05 | 4.986149 |
| IGHM | -2.3729 | 12.69246 | -5.42265 | 1.48E-06 | 4.46E-05 | 4.983101 |
| HSD3B7 | -1.49935 | 13.2246 | -5.42237 | 1.48E-06 | 4.46E-05 | 4.982138 |
| CD74 | 1.053449 | 13.25797 | 5.422081 | 1.48E-06 | 4.46E-05 | 4.981126 |
| LARP1P1 | -2.1324 | 13.27987 | -5.42029 | 1.49E-06 | 4.48E-05 | 4.974917 |
| ZNF823 | 1.028843 | 13.32885 | 5.419725 | 1.49E-06 | 4.48E-05 | 4.972943 |
| ZNF853 | 1.581839 | 13.18704 | 5.419265 | 1.50E-06 | 4.48E-05 | 4.971343 |
| EGF | -1.99223 | 13.0032 | -5.418 | 1.50E-06 | 4.50E-05 | 4.966957 |
| CLEC4D | -1.94822 | 13.02105 | -5.41563 | 1.52E-06 | 4.53E-05 | 4.958725 |
| CLEC9A | 1.437691 | 12.75701 | 5.41404 | 1.52E-06 | 4.55E-05 | 4.953197 |
| IGHV3-20 | -3.51212 | 11.54015 | -5.41249 | 1.53E-06 | 4.55E-05 | 4.947802 |
| RPL4P5 | 1.752149 | 13.12422 | 5.412402 | 1.53E-06 | 4.55E-05 | 4.947509 |
| PAQR8 | 1.037596 | 13.24971 | 5.412083 | 1.54E-06 | 4.55E-05 | 4.946404 |
| UPP1 | -1.50426 | 13.12597 | -5.41202 | 1.54E-06 | 4.55E-05 | 4.946198 |
| CDK20 | 1.375582 | 13.13553 | 5.407007 | 1.56E-06 | 4.61E-05 | 4.928782 |
| HSPB9 | -1.48964 | 13.34174 | -5.40665 | 1.57E-06 | 4.61E-05 | 4.927553 |
| ZIK1 | 1.348091 | 13.29494 | 5.405201 | 1.57E-06 | 4.63E-05 | 4.922513 |
| SPACA7 | -1.56107 | 13.36378 | -5.40104 | 1.60E-06 | 4.69E-05 | 4.908086 |
| HYAL1 | -1.19183 | 13.28352 | -5.40075 | 1.60E-06 | 4.69E-05 | 4.907069 |
| AC011979.1 | 2.88784 | 12.70117 | 5.39776 | 1.62E-06 | 4.73E-05 | 4.89669 |
| RAB13 | -1.53525 | 12.77193 | -5.39713 | 1.62E-06 | 4.73E-05 | 4.894515 |
| MGAM | -1.92028 | 13.05277 | -5.39535 | 1.63E-06 | 4.75E-05 | 4.888329 |
| ZNF329 | 1.038339 | 13.35366 | 5.394575 | 1.63E-06 | 4.76E-05 | 4.88564 |
| ENPP3 | 2.014963 | 12.69539 | 5.387233 | 1.68E-06 | 4.87E-05 | 4.860178 |
| AC004453.1 | 2.020312 | 13.12132 | 5.37594 | 1.75E-06 | 5.05E-05 | 4.821028 |
| SKAP1 | 1.604399 | 13.1255 | 5.374814 | 1.76E-06 | 5.06E-05 | 4.817125 |
| RPL9 | 1.733813 | 12.92572 | 5.372968 | 1.77E-06 | 5.09E-05 | 4.810728 |
| CLDN9 | -1.4706 | 13.16911 | -5.37131 | 1.78E-06 | 5.11E-05 | 4.804973 |
| CACNB4 | 1.530729 | 13.07091 | 5.369597 | 1.79E-06 | 5.14E-05 | 4.799049 |
| RPL7P19 | 2.461606 | 12.9359 | 5.367898 | 1.80E-06 | 5.17E-05 | 4.793162 |
| B4GALT2 | -1.42636 | 13.17812 | -5.36676 | 1.81E-06 | 5.18E-05 | 4.789224 |
| SPAG16 | 1.443171 | 13.29726 | 5.364499 | 1.82E-06 | 5.22E-05 | 4.781388 |
| B9D1 | -2.54284 | 12.89435 | -5.36442 | 1.82E-06 | 5.22E-05 | 4.781102 |
| SYT5 | -1.5848 | 13.11986 | -5.36416 | 1.82E-06 | 5.22E-05 | 4.780214 |
| SHROOM4 | -1.7667 | 12.93334 | -5.36238 | 1.84E-06 | 5.24E-05 | 4.774033 |
| APOBEC3A | -1.4075 | 13.3766 | -5.36086 | 1.85E-06 | 5.26E-05 | 4.768786 |
| RPS3AP47 | 2.147904 | 13.14119 | 5.358773 | 1.86E-06 | 5.29E-05 | 4.761559 |
| AL590004.2 | 1.001206 | 14.75996 | 5.355603 | 1.88E-06 | 5.33E-05 | 4.750586 |
| NR1D2 | 1.603535 | 13.25289 | 5.355076 | 1.88E-06 | 5.34E-05 | 4.748761 |
| AK4 | -1.06568 | 13.36706 | -5.35469 | 1.89E-06 | 5.34E-05 | 4.747435 |
| BIK | -1.33251 | 13.26574 | -5.35309 | 1.90E-06 | 5.37E-05 | 4.74189 |
| CD200R1 | 1.416732 | 13.10671 | 5.352711 | 1.90E-06 | 5.37E-05 | 4.740572 |
| GCNT4 | 2.350054 | 12.88112 | 5.352283 | 1.90E-06 | 5.37E-05 | 4.739092 |
| RPL5P32 | 1.064019 | 15.04303 | 5.349975 | 1.92E-06 | 5.41E-05 | 4.731103 |
| PHLDA3 | -1.13899 | 13.31006 | -5.34884 | 1.93E-06 | 5.42E-05 | 4.727188 |
| NEURL1 | 1.880483 | 12.51221 | 5.340841 | 1.98E-06 | 5.57E-05 | 4.699499 |
| TBC1D4 | 1.843231 | 13.01672 | 5.340146 | 1.99E-06 | 5.58E-05 | 4.697095 |
| PIM3 | -1.14668 | 13.28902 | -5.34009 | 1.99E-06 | 5.58E-05 | 4.696887 |
| SHKBP1 | -1.10703 | 13.26096 | -5.33982 | 1.99E-06 | 5.58E-05 | 4.695967 |
| TRIM60P18 | 1.467449 | 13.14777 | 5.338116 | 2.00E-06 | 5.59E-05 | 4.690072 |
| TRBV7-9 | 2.003008 | 12.8857 | 5.33794 | 2.00E-06 | 5.59E-05 | 4.689463 |
| GSTA4 | 1.229665 | 13.08656 | 5.337361 | 2.01E-06 | 5.60E-05 | 4.687461 |
| TARM1 | -2.54936 | 12.11891 | -5.33566 | 2.02E-06 | 5.63E-05 | 4.681573 |
| RPL7P32 | 1.797102 | 13.1677 | 5.334979 | 2.02E-06 | 5.64E-05 | 4.679224 |
| IGHV1-17 | -2.6441 | 12.14803 | -5.33428 | 2.03E-06 | 5.64E-05 | 4.676802 |
| IGHV3-13 | -3.16478 | 11.9046 | -5.33408 | 2.03E-06 | 5.64E-05 | 4.676101 |
| SUCNR1 | -2.05207 | 12.45975 | -5.33322 | 2.04E-06 | 5.65E-05 | 4.673127 |
| RSPH9 | -1.47326 | 13.15066 | -5.32894 | 2.07E-06 | 5.73E-05 | 4.658327 |
| AC009570.2 | -1.80847 | 13.12004 | -5.32868 | 2.07E-06 | 5.73E-05 | 4.657453 |
| HOPX | 1.475975 | 13.12659 | 5.328476 | 2.07E-06 | 5.73E-05 | 4.656739 |
| LIPM | -2.49498 | 12.93188 | -5.3283 | 2.07E-06 | 5.73E-05 | 4.65614 |
| TLR5 | -1.57812 | 13.30476 | -5.32786 | 2.08E-06 | 5.73E-05 | 4.654604 |
| EEF1A1P29 | 2.15308 | 13.26199 | 5.327123 | 2.08E-06 | 5.74E-05 | 4.652063 |
| IL2 | 1.688939 | 13.75239 | 5.322256 | 2.12E-06 | 5.81E-05 | 4.63524 |
| AL645568.2 | -1.89909 | 13.26695 | -5.32031 | 2.13E-06 | 5.85E-05 | 4.628503 |
| SOCS3 | -1.77348 | 13.27037 | -5.31789 | 2.15E-06 | 5.89E-05 | 4.620148 |
| MYDGF | -1.23381 | 13.30429 | -5.31773 | 2.15E-06 | 5.89E-05 | 4.619617 |
| CDHR3 | 1.387203 | 13.14374 | 5.31495 | 2.17E-06 | 5.95E-05 | 4.609995 |
| DHRS9 | -1.56474 | 13.22584 | -5.31471 | 2.18E-06 | 5.95E-05 | 4.609176 |
| CD96 | 1.633217 | 13.1278 | 5.313651 | 2.19E-06 | 5.96E-05 | 4.60551 |
| SIPA1L2 | -1.62435 | 13.25082 | -5.31324 | 2.19E-06 | 5.96E-05 | 4.604079 |
| COLQ | 1.569251 | 13.14759 | 5.313146 | 2.19E-06 | 5.96E-05 | 4.603766 |
| CDC42EP3 | -1.02773 | 13.41876 | -5.31035 | 2.21E-06 | 6.02E-05 | 4.594108 |
| AC012569.1 | -1.74348 | 13.35151 | -5.30893 | 2.22E-06 | 6.03E-05 | 4.589197 |
| AC103810.9 | -1.26836 | 13.26487 | -5.30869 | 2.22E-06 | 6.03E-05 | 4.588364 |
| SEPTIN1 | 1.150522 | 13.22322 | 5.308646 | 2.22E-06 | 6.03E-05 | 4.588223 |
| MATK | 1.513592 | 13.02594 | 5.308023 | 2.23E-06 | 6.04E-05 | 4.586072 |
| CROT | 1.108322 | 13.33607 | 5.306532 | 2.24E-06 | 6.06E-05 | 4.580925 |
| CACNG6 | 3.627879 | 11.3325 | 5.305903 | 2.25E-06 | 6.07E-05 | 4.57875 |
| IGKV3-11 | -2.60636 | 12.64662 | -5.30459 | 2.26E-06 | 6.09E-05 | 4.574212 |
| CLEC5A | -1.67578 | 12.94477 | -5.30132 | 2.28E-06 | 6.16E-05 | 4.562947 |
| DCC | -1.76897 | 13.07583 | -5.30031 | 2.29E-06 | 6.18E-05 | 4.559431 |
| BTBD11 | 1.320731 | 13.22927 | 5.296435 | 2.32E-06 | 6.25E-05 | 4.546071 |
| FAP | -3.18088 | 12.7418 | -5.29483 | 2.34E-06 | 6.28E-05 | 4.540532 |
| FAM111B | -1.40108 | 13.30758 | -5.29338 | 2.35E-06 | 6.31E-05 | 4.535526 |
| SNED1 | 1.461026 | 13.0433 | 5.291994 | 2.36E-06 | 6.33E-05 | 4.530745 |
| ICAM5 | -1.70115 | 13.02616 | -5.29128 | 2.37E-06 | 6.34E-05 | 4.528281 |
| RPL5P24 | 1.237039 | 14.42187 | 5.290451 | 2.37E-06 | 6.35E-05 | 4.525423 |
| IGLV3-21 | -2.8603 | 12.74644 | -5.29025 | 2.38E-06 | 6.35E-05 | 4.524719 |
| TRBV6-6 | 1.892338 | 12.94818 | 5.290021 | 2.38E-06 | 6.35E-05 | 4.523939 |
| LAMA2 | 1.792594 | 13.11538 | 5.289133 | 2.39E-06 | 6.36E-05 | 4.520877 |
| ATG9B | 1.711985 | 12.97224 | 5.288627 | 2.39E-06 | 6.36E-05 | 4.51913 |
| CHRNB2 | -1.79433 | 13.38551 | -5.28844 | 2.39E-06 | 6.36E-05 | 4.518478 |
| RNF24 | -1.17112 | 13.34428 | -5.28704 | 2.40E-06 | 6.39E-05 | 4.513658 |
| WNT7A | 2.147506 | 12.8366 | 5.286575 | 2.41E-06 | 6.39E-05 | 4.512052 |
| LTB4R | -1.16194 | 13.27141 | -5.28615 | 2.41E-06 | 6.39E-05 | 4.510595 |
| TXNDC11 | -1.20306 | 13.36577 | -5.28526 | 2.42E-06 | 6.41E-05 | 4.507534 |
| ZNF547 | 1.099921 | 13.35286 | 5.283888 | 2.43E-06 | 6.42E-05 | 4.502784 |
| TNK1 | 1.545889 | 13.08806 | 5.281991 | 2.45E-06 | 6.46E-05 | 4.496246 |
| H2BC4 | -1.55832 | 13.36261 | -5.2803 | 2.46E-06 | 6.48E-05 | 4.490411 |
| EEF1A1P28 | 1.947537 | 13.51959 | 5.277627 | 2.49E-06 | 6.53E-05 | 4.481199 |
| GINS4 | -1.10743 | 13.29901 | -5.27731 | 2.49E-06 | 6.54E-05 | 4.480109 |
| CCR6 | 1.730414 | 13.21518 | 5.27666 | 2.49E-06 | 6.55E-05 | 4.477865 |
| COL26A1 | 3.600252 | 11.50799 | 5.275635 | 2.50E-06 | 6.56E-05 | 4.474331 |
| MANSC1 | -1.49039 | 13.26146 | -5.27439 | 2.51E-06 | 6.58E-05 | 4.470034 |
| GLI1 | -1.91693 | 13.26773 | -5.27409 | 2.52E-06 | 6.58E-05 | 4.468992 |
| FMNL3 | 1.140308 | 13.21444 | 5.271679 | 2.54E-06 | 6.63E-05 | 4.460697 |
| CD3D | 1.731044 | 13.03248 | 5.270119 | 2.55E-06 | 6.67E-05 | 4.455321 |
| AP001324.1 | 2.159743 | 12.91808 | 5.265675 | 2.59E-06 | 6.77E-05 | 4.44001 |
| ANXA3 | -2.28205 | 12.7046 | -5.26522 | 2.60E-06 | 6.77E-05 | 4.438454 |
| GADD45A | -1.86833 | 12.71086 | -5.26501 | 2.60E-06 | 6.77E-05 | 4.437717 |
| BRCA2 | -1.18687 | 13.27755 | -5.26202 | 2.63E-06 | 6.83E-05 | 4.427429 |
| VNN1 | -2.43495 | 12.73601 | -5.26052 | 2.64E-06 | 6.86E-05 | 4.422267 |
| AC138827.1 | -2.38154 | 13.13717 | -5.25937 | 2.65E-06 | 6.88E-05 | 4.418281 |
| NAIPP1 | -1.8321 | 13.27788 | -5.25767 | 2.67E-06 | 6.91E-05 | 4.412441 |
| SMKR1 | 1.636659 | 13.18833 | 5.257232 | 2.67E-06 | 6.91E-05 | 4.41093 |
| SLC2A5 | -1.7426 | 12.65026 | -5.25703 | 2.67E-06 | 6.91E-05 | 4.410219 |
| SSC4D | -1.42385 | 13.352 | -5.25558 | 2.69E-06 | 6.94E-05 | 4.405258 |
| GAL3ST4 | 1.232691 | 13.19004 | 5.252095 | 2.72E-06 | 7.01E-05 | 4.393244 |
| LILRA5 | -1.38304 | 13.20005 | -5.25176 | 2.73E-06 | 7.01E-05 | 4.392081 |
| H2BC20P | -1.37026 | 13.33367 | -5.25022 | 2.74E-06 | 7.05E-05 | 4.386789 |
| EPHA4 | 1.72607 | 13.12163 | 5.249271 | 2.75E-06 | 7.06E-05 | 4.383523 |
| CD8B | 2.062092 | 12.88547 | 5.245631 | 2.79E-06 | 7.15E-05 | 4.370997 |
| ZNF302 | 1.117869 | 13.32034 | 5.244813 | 2.79E-06 | 7.16E-05 | 4.368181 |
| PLA2G4F | -1.61894 | 13.41382 | -5.24464 | 2.80E-06 | 7.16E-05 | 4.367585 |
| KLRK1 | 1.7837 | 13.05108 | 5.243642 | 2.81E-06 | 7.18E-05 | 4.364152 |
| TRBV14 | 1.948075 | 12.78232 | 5.242352 | 2.82E-06 | 7.21E-05 | 4.359716 |
| TP53I3 | -1.3949 | 13.15098 | -5.24177 | 2.82E-06 | 7.21E-05 | 4.357722 |
| KCNJ2 | -1.35754 | 13.36893 | -5.23851 | 2.86E-06 | 7.27E-05 | 4.346503 |
| IGHV3-49 | -3.03404 | 11.54865 | -5.2385 | 2.86E-06 | 7.27E-05 | 4.346457 |
| MAN1C1 | 1.422384 | 13.16899 | 5.238161 | 2.86E-06 | 7.28E-05 | 4.345296 |
| ZNF248 | 1.16746 | 13.27504 | 5.23738 | 2.87E-06 | 7.29E-05 | 4.34261 |
| AC018755.3 | -1.85357 | 13.26728 | -5.23482 | 2.90E-06 | 7.34E-05 | 4.333816 |
| MSANTD2 | 1.124101 | 13.27792 | 5.234336 | 2.90E-06 | 7.34E-05 | 4.332142 |
| MYLK4 | 1.04969 | 13.16858 | 5.233311 | 2.91E-06 | 7.37E-05 | 4.328617 |
| RPL21P28 | 1.782337 | 12.91326 | 5.233005 | 2.91E-06 | 7.37E-05 | 4.327564 |
| RPS3AP26 | 2.472698 | 12.68148 | 5.230407 | 2.94E-06 | 7.42E-05 | 4.318631 |
| ZFHX2 | -1.46492 | 13.20726 | -5.22988 | 2.95E-06 | 7.43E-05 | 4.316828 |
| EGFL8 | 1.341215 | 12.56526 | 5.229604 | 2.95E-06 | 7.43E-05 | 4.315871 |
| NFE2 | -1.21897 | 13.15011 | -5.22938 | 2.95E-06 | 7.43E-05 | 4.315091 |
| ZNF256 | 1.226 | 13.33093 | 5.228875 | 2.96E-06 | 7.43E-05 | 4.313366 |
| TRAV8-3 | 2.137036 | 12.9132 | 5.225657 | 2.99E-06 | 7.50E-05 | 4.302303 |
| FCGR1B | -1.60157 | 13.29759 | -5.22425 | 3.01E-06 | 7.52E-05 | 4.297473 |
| RPS3AP44 | 1.748238 | 13.69836 | 5.22346 | 3.01E-06 | 7.54E-05 | 4.294755 |
| AC104561.2 | -1.96554 | 12.14276 | -5.22256 | 3.02E-06 | 7.55E-05 | 4.291674 |
| TRAF5 | 1.355555 | 13.25098 | 5.217475 | 3.08E-06 | 7.67E-05 | 4.274189 |
| CD3E | 1.741473 | 13.06638 | 5.216891 | 3.09E-06 | 7.68E-05 | 4.272181 |
| HNRNPA3P6 | -2.22594 | 12.92039 | -5.21571 | 3.10E-06 | 7.70E-05 | 4.268116 |
| AGTRAP | -1.07391 | 13.30463 | -5.21454 | 3.11E-06 | 7.73E-05 | 4.26409 |
| DEPP1 | -2.4826 | 12.79527 | -5.21276 | 3.13E-06 | 7.77E-05 | 4.257977 |
| COL6A2 | 1.947945 | 12.88183 | 5.212231 | 3.14E-06 | 7.78E-05 | 4.256176 |
| ACKR3 | 1.441535 | 13.27085 | 5.2117 | 3.14E-06 | 7.78E-05 | 4.254351 |
| PDIA5 | -1.16898 | 13.33024 | -5.21083 | 3.15E-06 | 7.80E-05 | 4.251354 |
| GP1BB | -4.32698 | 11.92515 | -5.21031 | 3.16E-06 | 7.81E-05 | 4.249579 |
| OR5K2 | 2.176368 | 13.02978 | 5.209059 | 3.17E-06 | 7.84E-05 | 4.245283 |
| AC006042.2 | -1.67886 | 12.923 | -5.20447 | 3.23E-06 | 7.95E-05 | 4.229541 |
| SDF2L1 | -1.40849 | 13.26842 | -5.20327 | 3.24E-06 | 7.96E-05 | 4.22542 |
| FLT1 | -1.11794 | 13.31485 | -5.2019 | 3.26E-06 | 8.00E-05 | 4.220696 |
| CYP1A1 | -2.39398 | 13.14605 | -5.20087 | 3.27E-06 | 8.02E-05 | 4.217155 |
| AC008507.1 | 1.415451 | 13.21574 | 5.199693 | 3.28E-06 | 8.05E-05 | 4.213131 |
| SORBS3 | 1.481788 | 13.06913 | 5.19903 | 3.29E-06 | 8.06E-05 | 4.210856 |
| PEAR1 | -1.71903 | 12.92756 | -5.19283 | 3.36E-06 | 8.22E-05 | 4.189589 |
| TRBV6-2 | 2.090369 | 12.79228 | 5.192007 | 3.37E-06 | 8.23E-05 | 4.186761 |
| TRBV29-1 | 2.141678 | 12.71899 | 5.191871 | 3.37E-06 | 8.23E-05 | 4.186292 |
| MYBL1 | 2.093946 | 12.92999 | 5.191702 | 3.38E-06 | 8.23E-05 | 4.185714 |
| H2BC11 | -1.42092 | 13.1588 | -5.18742 | 3.43E-06 | 8.34E-05 | 4.171038 |
| GCSAM | 1.539003 | 13.15757 | 5.186565 | 3.44E-06 | 8.36E-05 | 4.168093 |
| BEX2 | 1.681312 | 13.16554 | 5.183395 | 3.48E-06 | 8.44E-05 | 4.157224 |
| EXOSC4 | -1.19966 | 13.24535 | -5.18249 | 3.49E-06 | 8.45E-05 | 4.154137 |
| LTBP3 | 1.263241 | 13.1514 | 5.181933 | 3.50E-06 | 8.46E-05 | 4.152215 |
| CLDN6 | -1.79464 | 13.41812 | -5.18151 | 3.50E-06 | 8.46E-05 | 4.150774 |
| NIPSNAP3B | 1.073276 | 13.27287 | 5.180929 | 3.51E-06 | 8.46E-05 | 4.14877 |
| RPL14P1 | 1.167386 | 13.18926 | 5.179988 | 3.52E-06 | 8.48E-05 | 4.145546 |
| MMRN1 | -1.64889 | 12.96928 | -5.17793 | 3.55E-06 | 8.53E-05 | 4.138499 |
| RBFOX2 | 1.20447 | 13.2956 | 5.176779 | 3.56E-06 | 8.55E-05 | 4.134547 |
| WNT5B | -1.51429 | 13.21709 | -5.17605 | 3.57E-06 | 8.56E-05 | 4.132035 |
| HPN | -1.98915 | 13.27031 | -5.17484 | 3.58E-06 | 8.58E-05 | 4.127894 |
| LAMC1 | -1.83216 | 13.16255 | -5.17471 | 3.59E-06 | 8.58E-05 | 4.127462 |
| RPL21P6 | 1.417842 | 14.08615 | 5.173603 | 3.60E-06 | 8.60E-05 | 4.123665 |
| PRR11 | -1.11664 | 13.19471 | -5.17123 | 3.63E-06 | 8.66E-05 | 4.115534 |
| CCNE1 | -1.46991 | 13.13797 | -5.17088 | 3.64E-06 | 8.67E-05 | 4.114337 |
| AC125807.1 | 1.797598 | 13.17459 | 5.166684 | 3.69E-06 | 8.78E-05 | 4.099959 |
| FAT2 | -1.4514 | 13.03755 | -5.16645 | 3.69E-06 | 8.78E-05 | 4.099169 |
| CRACD | -1.92404 | 13.02351 | -5.16563 | 3.70E-06 | 8.80E-05 | 4.096358 |
| TPT1P3 | 1.076762 | 15.14115 | 5.165261 | 3.71E-06 | 8.80E-05 | 4.095087 |
| SEMA4C | 1.140009 | 13.24917 | 5.164888 | 3.71E-06 | 8.80E-05 | 4.09381 |
| SHOC1 | -1.86866 | 12.94487 | -5.16339 | 3.73E-06 | 8.84E-05 | 4.088693 |
| OTUD7A | 1.642022 | 13.13311 | 5.163287 | 3.73E-06 | 8.84E-05 | 4.088327 |
| BCL11B | 1.832991 | 13.04023 | 5.157089 | 3.82E-06 | 9.02E-05 | 4.067107 |
| NOTCH2NLA | -1.17812 | 13.3025 | -5.15604 | 3.83E-06 | 9.04E-05 | 4.063506 |
| DYNC2H1 | 2.2185 | 12.72152 | 5.154947 | 3.85E-06 | 9.06E-05 | 4.059777 |
| FBLN5 | 1.881035 | 12.91847 | 5.15492 | 3.85E-06 | 9.06E-05 | 4.059684 |
| FFAR3 | -2.38049 | 13.05737 | -5.15333 | 3.87E-06 | 9.11E-05 | 4.054228 |
| ECRG4 | 2.637501 | 12.66051 | 5.152208 | 3.88E-06 | 9.14E-05 | 4.050401 |
| TMEM30B | 1.908074 | 13.05041 | 5.148533 | 3.94E-06 | 9.24E-05 | 4.037829 |
| SARDH | 1.734543 | 12.96115 | 5.148118 | 3.94E-06 | 9.25E-05 | 4.036409 |
| TRAV1-1 | 1.818663 | 13.02172 | 5.14684 | 3.96E-06 | 9.27E-05 | 4.032034 |
| RPS27AP5 | 1.860683 | 13.14415 | 5.145601 | 3.98E-06 | 9.30E-05 | 4.027798 |
| GNPDA2 | 1.045102 | 13.3423 | 5.145522 | 3.98E-06 | 9.30E-05 | 4.027527 |
| CPEB4 | -1.05209 | 13.23655 | -5.14541 | 3.98E-06 | 9.30E-05 | 4.027154 |
| APBB1 | 1.428971 | 13.16363 | 5.143049 | 4.01E-06 | 9.37E-05 | 4.01907 |
| RPL21P93 | 2.219873 | 12.89707 | 5.142766 | 4.02E-06 | 9.37E-05 | 4.018101 |
| DBNDD1 | 1.991508 | 12.91029 | 5.140867 | 4.04E-06 | 9.43E-05 | 4.011605 |
| GFPT2 | 2.489455 | 12.64574 | 5.140024 | 4.06E-06 | 9.44E-05 | 4.008725 |
| HABP4 | 1.176156 | 13.2714 | 5.139473 | 4.06E-06 | 9.44E-05 | 4.00684 |
| HSD17B14 | 2.01542 | 13.0349 | 5.139444 | 4.06E-06 | 9.44E-05 | 4.006741 |
| MSRB3 | -1.15631 | 13.01334 | -5.13907 | 4.07E-06 | 9.45E-05 | 4.005473 |
| PKIA | 1.354116 | 13.30207 | 5.138914 | 4.07E-06 | 9.45E-05 | 4.00493 |
| AC002075.2 | 1.789963 | 13.31752 | 5.137996 | 4.09E-06 | 9.46E-05 | 4.001789 |
| ADORA1 | 2.636735 | 12.5034 | 5.13632 | 4.11E-06 | 9.51E-05 | 3.99606 |
| AC091304.3 | -1.70298 | 13.24419 | -5.13484 | 4.13E-06 | 9.55E-05 | 3.990994 |
| AC243913.10 | -1.70298 | 13.24419 | -5.13484 | 4.13E-06 | 9.55E-05 | 3.990994 |
| PLSCR4 | -1.4615 | 12.688 | -5.1343 | 4.14E-06 | 9.56E-05 | 3.989161 |
| H4C5 | -2.27241 | 13.13442 | -5.13329 | 4.15E-06 | 9.59E-05 | 3.985698 |
| TRIM51EP | 3.001849 | 12.00402 | 5.132554 | 4.16E-06 | 9.60E-05 | 3.983188 |
| RPL30P7 | -2.25127 | 12.97927 | -5.13135 | 4.18E-06 | 9.64E-05 | 3.979076 |
| PKMP3 | 1.512747 | 13.21366 | 5.129944 | 4.20E-06 | 9.67E-05 | 3.974266 |
| GPR68 | 1.296467 | 13.14934 | 5.129142 | 4.22E-06 | 9.68E-05 | 3.971526 |
| EHF | 2.300805 | 12.86339 | 5.128738 | 4.22E-06 | 9.69E-05 | 3.970146 |
| TESPA1 | 1.558668 | 13.13155 | 5.128637 | 4.22E-06 | 9.69E-05 | 3.9698 |
| ARHGAP11A | -1.54444 | 13.04603 | -5.12768 | 4.24E-06 | 9.71E-05 | 3.966539 |
| HSD17B3 | 2.032992 | 12.91639 | 5.126706 | 4.25E-06 | 9.74E-05 | 3.963202 |
| PHTF1 | -1.07134 | 13.36205 | -5.12492 | 4.28E-06 | 9.79E-05 | 3.95711 |
| CASD1 | 1.133199 | 13.34964 | 5.124796 | 4.28E-06 | 9.79E-05 | 3.956677 |
| ARG1 | -3.27114 | 11.84569 | -5.12278 | 4.31E-06 | 9.85E-05 | 3.949799 |
| ZNF501 | 1.192203 | 13.34822 | 5.122394 | 4.32E-06 | 9.85E-05 | 3.948473 |
| NCALD | 1.455144 | 13.21014 | 5.122339 | 4.32E-06 | 9.85E-05 | 3.948284 |
| IMMP2L | 1.253664 | 13.2228 | 5.12167 | 4.33E-06 | 9.87E-05 | 3.946001 |
| Z95152.1 | -2.49643 | 12.97353 | -5.11945 | 4.36E-06 | 9.94E-05 | 3.938419 |
| SLC35F3 | 2.945063 | 12.4618 | 5.119199 | 4.37E-06 | 9.94E-05 | 3.93756 |
| EXTL2 | 1.003727 | 13.33487 | 5.11907 | 4.37E-06 | 9.94E-05 | 3.937122 |
| IGLV3-22 | -3.14887 | 12.3784 | -5.11793 | 4.39E-06 | 9.96E-05 | 3.933239 |
| ZNF337 | 1.024404 | 13.28807 | 5.114854 | 4.43E-06 | 0.000101 | 3.922724 |
| IGHV3-73 | -2.85146 | 11.14686 | -5.11447 | 4.44E-06 | 0.000101 | 3.921401 |
| LRRN1 | -2.01563 | 13.11131 | -5.11431 | 4.44E-06 | 0.000101 | 3.920877 |
| OR2W6P | -2.05797 | 12.88103 | -5.11329 | 4.46E-06 | 0.000101 | 3.917388 |
| GRAP | 1.184905 | 13.28125 | 5.113198 | 4.46E-06 | 0.000101 | 3.917073 |
| POU2AF1 | -1.50159 | 13.29457 | -5.11239 | 4.47E-06 | 0.000101 | 3.914326 |
| NBL1 | 1.511365 | 12.79057 | 5.111615 | 4.49E-06 | 0.000101 | 3.911668 |
| DPP4 | 1.724957 | 13.10117 | 5.11145 | 4.49E-06 | 0.000101 | 3.911106 |
| AC000095.1 | -1.61556 | 13.18615 | -5.10891 | 4.53E-06 | 0.000102 | 3.90243 |
| ZNF649 | 1.045024 | 13.37157 | 5.104032 | 4.61E-06 | 0.000103 | 3.885793 |
| GPR18 | 1.537396 | 13.21037 | 5.103108 | 4.62E-06 | 0.000104 | 3.88264 |
| TSHR | -2.08694 | 12.97011 | -5.10263 | 4.63E-06 | 0.000104 | 3.881022 |
| TMEM119 | -2.36734 | 12.71455 | -5.10234 | 4.64E-06 | 0.000104 | 3.880021 |
| IGHV4-28 | -2.75029 | 12.73133 | -5.10072 | 4.66E-06 | 0.000104 | 3.874508 |
| GDF10 | 2.515968 | 12.43703 | 5.099393 | 4.68E-06 | 0.000105 | 3.869968 |
| RNF125 | 1.274263 | 13.25712 | 5.098722 | 4.69E-06 | 0.000105 | 3.867678 |
| TIFAB | 3.448188 | 12.03038 | 5.098608 | 4.70E-06 | 0.000105 | 3.867289 |
| CMTM5 | -1.94936 | 12.90947 | -5.09717 | 4.72E-06 | 0.000105 | 3.86238 |
| TRGC2 | 1.744774 | 12.96514 | 5.096198 | 4.74E-06 | 0.000105 | 3.859072 |
| ABLIM2 | 1.376009 | 13.25768 | 5.095367 | 4.75E-06 | 0.000106 | 3.856237 |
| MTCO2P12 | 1.142776 | 13.22614 | 5.094257 | 4.77E-06 | 0.000106 | 3.852452 |
| CLCF1 | 1.08444 | 13.10607 | 5.092865 | 4.79E-06 | 0.000106 | 3.847707 |
| KCNE1 | -1.85845 | 13.02914 | -5.09275 | 4.80E-06 | 0.000106 | 3.847318 |
| LHFPL2 | -1.31593 | 13.3568 | -5.09131 | 4.82E-06 | 0.000107 | 3.842392 |
| HSF2 | 1.062683 | 13.28763 | 5.087006 | 4.89E-06 | 0.000108 | 3.827735 |
| AP1S2 | 1.066568 | 13.23681 | 5.08674 | 4.90E-06 | 0.000108 | 3.826829 |
| ITGA2B | -2.29827 | 12.57581 | -5.08639 | 4.90E-06 | 0.000108 | 3.825646 |
| FHIT | 1.661136 | 12.91458 | 5.085707 | 4.92E-06 | 0.000108 | 3.82331 |
| IGHV1-12 | -3.82552 | 11.83902 | -5.08473 | 4.93E-06 | 0.000109 | 3.819996 |
| MB21D2 | 1.562293 | 13.15961 | 5.0838 | 4.95E-06 | 0.000109 | 3.81681 |
| LOXHD1 | -1.61805 | 13.15198 | -5.08364 | 4.95E-06 | 0.000109 | 3.816266 |
| NSG2 | -2.5902 | 12.85678 | -5.07986 | 5.02E-06 | 0.00011 | 3.803379 |
| IGLV3-9 | -2.84286 | 12.5391 | -5.07936 | 5.03E-06 | 0.00011 | 3.801685 |
| TRIM25 | -1.16225 | 13.40561 | -5.07936 | 5.03E-06 | 0.00011 | 3.801682 |
| CD40LG | 1.782186 | 13.01391 | 5.075339 | 5.10E-06 | 0.000112 | 3.787992 |
| H2BC17 | -2.92447 | 12.71709 | -5.07337 | 5.14E-06 | 0.000112 | 3.781273 |
| MTRNR2L4 | 1.545264 | 13.03605 | 5.073183 | 5.14E-06 | 0.000112 | 3.780651 |
| GCKR | -2.53542 | 12.3814 | -5.0723 | 5.15E-06 | 0.000112 | 3.777631 |
| CTTNBP2 | 2.890192 | 12.30409 | 5.072222 | 5.16E-06 | 0.000112 | 3.777377 |
| RPL7AP31 | 1.670599 | 13.24056 | 5.072086 | 5.16E-06 | 0.000112 | 3.776915 |
| SIRPG | 1.664981 | 13.05457 | 5.067857 | 5.24E-06 | 0.000114 | 3.762519 |
| MLF1 | 1.311951 | 13.1154 | 5.067356 | 5.25E-06 | 0.000114 | 3.760813 |
| ZNF287 | 1.40465 | 13.2543 | 5.0664 | 5.26E-06 | 0.000114 | 3.757559 |
| SPATS2 | -1.21651 | 13.34357 | -5.06624 | 5.27E-06 | 0.000114 | 3.757002 |
| RPL10P16 | 1.034811 | 13.36924 | 5.06418 | 5.30E-06 | 0.000115 | 3.750006 |
| CAPN13 | -3.20273 | 12.51412 | -5.06331 | 5.32E-06 | 0.000115 | 3.747048 |
| SNTA1 | 1.055138 | 13.29136 | 5.062645 | 5.33E-06 | 0.000115 | 3.744782 |
| CRTAM | 1.606654 | 13.15597 | 5.061495 | 5.36E-06 | 0.000115 | 3.74087 |
| CD4 | 1.496659 | 13.07133 | 5.061368 | 5.36E-06 | 0.000115 | 3.740438 |
| IGKV3OR2-268 | -3.21354 | 12.60165 | -5.06135 | 5.36E-06 | 0.000115 | 3.740386 |
| CADPS2 | -2.3184 | 12.62259 | -5.06106 | 5.36E-06 | 0.000115 | 3.739382 |
| C4orf17 | -2.08096 | 13.00353 | -5.06075 | 5.37E-06 | 0.000115 | 3.73835 |
| CDKN2A | -1.42123 | 13.15498 | -5.05897 | 5.40E-06 | 0.000116 | 3.73229 |
| AC139495.2 | -2.07752 | 13.08066 | -5.05619 | 5.46E-06 | 0.000117 | 3.722839 |
| CXCL8 | 2.234168 | 12.78491 | 5.055308 | 5.47E-06 | 0.000117 | 3.719825 |
| KIF5C | 1.631735 | 13.23954 | 5.055197 | 5.48E-06 | 0.000117 | 3.719447 |
| SPOCK2 | 1.516033 | 13.15977 | 5.054328 | 5.49E-06 | 0.000117 | 3.716492 |
| AL353803.3 | -2.25556 | 13.25876 | -5.05429 | 5.49E-06 | 0.000117 | 3.716374 |
| SPTSSB | 2.142978 | 12.94944 | 5.053293 | 5.51E-06 | 0.000117 | 3.712972 |
| ADGRG6 | 1.783239 | 12.87268 | 5.052913 | 5.52E-06 | 0.000117 | 3.711678 |
| GATA3 | 1.548991 | 13.09602 | 5.052608 | 5.53E-06 | 0.000117 | 3.710644 |
| ADGRF5P1 | -1.63186 | 13.19501 | -5.05203 | 5.54E-06 | 0.000118 | 3.708663 |
| DNM1 | -1.38829 | 13.1609 | -5.05018 | 5.57E-06 | 0.000118 | 3.702387 |
| AC124312.1 | 1.728585 | 13.07935 | 5.050166 | 5.57E-06 | 0.000118 | 3.702342 |
| OPTN | 1.074518 | 12.72821 | 5.049742 | 5.58E-06 | 0.000118 | 3.700897 |
| IGHJ3P | -1.75296 | 13.16214 | -5.04604 | 5.66E-06 | 0.00012 | 3.688299 |
| MGST1 | -1.04056 | 13.20177 | -5.04565 | 5.66E-06 | 0.00012 | 3.686983 |
| IGKV1-16 | -2.37603 | 12.53001 | -5.04372 | 5.70E-06 | 0.00012 | 3.680419 |
| LILRA6 | -1.29021 | 13.26702 | -5.04368 | 5.70E-06 | 0.00012 | 3.680304 |
| MBLAC2 | 1.297287 | 13.31174 | 5.041054 | 5.76E-06 | 0.000121 | 3.671371 |
| UST | 1.350801 | 13.24681 | 5.039726 | 5.78E-06 | 0.000122 | 3.666859 |
| BCAS4 | 1.07326 | 13.31636 | 5.039316 | 5.79E-06 | 0.000122 | 3.665467 |
| IGLV2-28 | -2.46165 | 12.9079 | -5.03543 | 5.87E-06 | 0.000123 | 3.652251 |
| NRN1 | -2.09645 | 12.88223 | -5.03502 | 5.88E-06 | 0.000124 | 3.650887 |
| IL26 | 2.012349 | 13.18346 | 5.034723 | 5.89E-06 | 0.000124 | 3.649867 |
| CD244 | 1.166949 | 13.14548 | 5.034634 | 5.89E-06 | 0.000124 | 3.649563 |
| CIP2A | -1.02992 | 13.28118 | -5.03267 | 5.93E-06 | 0.000124 | 3.642877 |
| TP53I11 | -1.28773 | 13.19852 | -5.03175 | 5.95E-06 | 0.000125 | 3.639758 |
| ZNF542P | 1.176241 | 13.25409 | 5.031435 | 5.95E-06 | 0.000125 | 3.638701 |
| MRPS31P5 | 1.014822 | 13.29691 | 5.030364 | 5.98E-06 | 0.000125 | 3.635063 |
| IGHJ1 | -2.92211 | 12.31088 | -5.02753 | 6.04E-06 | 0.000126 | 3.625435 |
| EEF1A1P22 | 1.499195 | 13.20519 | 5.025984 | 6.07E-06 | 0.000127 | 3.620196 |
| TRPM2 | -1.21875 | 13.2083 | -5.02406 | 6.11E-06 | 0.000127 | 3.613667 |
| REXO1L1P | -1.01543 | 13.33822 | -5.02123 | 6.17E-06 | 0.000129 | 3.604073 |
| MTRNR2L10 | 1.955799 | 12.89702 | 5.020727 | 6.18E-06 | 0.000129 | 3.602357 |
| CCM2L | -2.50328 | 13.06014 | -5.01935 | 6.21E-06 | 0.000129 | 3.597678 |
| H1-2 | -1.46219 | 12.54405 | -5.01852 | 6.23E-06 | 0.000129 | 3.59488 |
| RPL7P47 | 1.798998 | 13.41278 | 5.01684 | 6.27E-06 | 0.00013 | 3.589167 |
| SMIM10L2A | 1.876619 | 12.92189 | 5.016626 | 6.27E-06 | 0.00013 | 3.588442 |
| KDM6B | -1.03108 | 13.32579 | -5.01567 | 6.29E-06 | 0.00013 | 3.585204 |
| TTLL7 | -1.28175 | 13.17922 | -5.01537 | 6.30E-06 | 0.00013 | 3.584187 |
| VN1R1 | 1.362915 | 13.19491 | 5.014979 | 6.31E-06 | 0.00013 | 3.582856 |
| XIAPP3 | -1.69362 | 13.25034 | -5.01455 | 6.32E-06 | 0.00013 | 3.581411 |
| PSTPIP2 | -1.2258 | 13.28862 | -5.01452 | 6.32E-06 | 0.00013 | 3.581286 |
| PPP1R17 | 3.425229 | 11.48523 | 5.013405 | 6.35E-06 | 0.000131 | 3.577519 |
| SAMD10 | 1.111154 | 13.16401 | 5.009787 | 6.43E-06 | 0.000132 | 3.56525 |
| IGHV3OR16-12 | -2.7944 | 11.51478 | -5.00794 | 6.47E-06 | 0.000133 | 3.558972 |
| CMC1 | 1.025234 | 13.28263 | 5.006915 | 6.49E-06 | 0.000133 | 3.555515 |
| EFHD1 | 1.449723 | 13.34029 | 5.006497 | 6.50E-06 | 0.000133 | 3.554096 |
| AC025449.1 | 1.336796 | 13.28505 | 5.00605 | 6.51E-06 | 0.000134 | 3.552581 |
| ADAM23 | 1.853541 | 13.06441 | 5.00595 | 6.51E-06 | 0.000134 | 3.552243 |
| NAV3 | -1.31405 | 13.26499 | -5.00433 | 6.55E-06 | 0.000134 | 3.546756 |
| CTNND2 | 2.068972 | 12.93124 | 5.004305 | 6.55E-06 | 0.000134 | 3.546668 |
| LRCOL1 | 1.50969 | 13.66924 | 5.003702 | 6.57E-06 | 0.000134 | 3.544624 |
| ZNF30 | 1.530174 | 13.26687 | 5.003443 | 6.57E-06 | 0.000134 | 3.543746 |
| SPI1 | -1.09906 | 13.27798 | -5.00072 | 6.63E-06 | 0.000136 | 3.534528 |
| YBX2P1 | -2.18226 | 13.10041 | -5.00009 | 6.65E-06 | 0.000136 | 3.532386 |
| ZNF540 | 1.674059 | 13.18342 | 4.999987 | 6.65E-06 | 0.000136 | 3.532035 |
| RFPL4A | 3.200706 | 10.91414 | 4.999877 | 6.65E-06 | 0.000136 | 3.531662 |
| ZNF461 | 1.167301 | 13.3305 | 4.99935 | 6.67E-06 | 0.000136 | 3.529876 |
| PEAK3 | -1.43754 | 13.19655 | -4.99903 | 6.67E-06 | 0.000136 | 3.528783 |
| ADCY4 | -1.1828 | 13.27984 | -4.99838 | 6.69E-06 | 0.000136 | 3.526587 |
| MMP8 | -3.78727 | 11.19712 | -4.99808 | 6.70E-06 | 0.000136 | 3.525578 |
| ACVR2A | 1.203822 | 13.22813 | 4.994692 | 6.78E-06 | 0.000137 | 3.514098 |
| ST8SIA5 | -1.39441 | 13.12995 | -4.99465 | 6.78E-06 | 0.000137 | 3.513966 |
| PLAAT4 | 1.267587 | 13.22132 | 4.993877 | 6.80E-06 | 0.000138 | 3.511337 |
| KCNE1B | -2.73674 | 12.7635 | -4.9887 | 6.92E-06 | 0.00014 | 3.493802 |
| MARCKSL1 | 1.032138 | 13.14075 | 4.988109 | 6.94E-06 | 0.00014 | 3.491807 |
| CCDC36 | -1.35136 | 13.20712 | -4.98282 | 7.07E-06 | 0.000142 | 3.47392 |
| ZNF74 | 1.011364 | 13.29525 | 4.980309 | 7.13E-06 | 0.000143 | 3.465409 |
| FGFR4 | -1.27287 | 13.09588 | -4.97805 | 7.19E-06 | 0.000144 | 3.457765 |
| AC009779.4 | -1.00033 | 13.2984 | -4.97715 | 7.21E-06 | 0.000145 | 3.454711 |
| SNAP91 | 1.715937 | 13.40789 | 4.977098 | 7.21E-06 | 0.000145 | 3.454546 |
| IGHV4-55 | -2.49528 | 12.75264 | -4.97692 | 7.21E-06 | 0.000145 | 3.453947 |
| TSEN54 | 1.030465 | 13.24346 | 4.975625 | 7.25E-06 | 0.000145 | 3.449564 |
| YBX1P1 | 1.041191 | 13.25114 | 4.975017 | 7.26E-06 | 0.000145 | 3.447506 |
| TRIM7 | -1.21066 | 13.21867 | -4.97457 | 7.27E-06 | 0.000146 | 3.44599 |
| OLFM1 | 3.995713 | 11.04817 | 4.974358 | 7.28E-06 | 0.000146 | 3.445277 |
| CBLB | 1.128986 | 13.29111 | 4.973418 | 7.30E-06 | 0.000146 | 3.442099 |
| FBLN2 | 2.05213 | 12.94694 | 4.97132 | 7.36E-06 | 0.000147 | 3.435006 |
| PPP4R1L | -1.06301 | 13.3571 | -4.97096 | 7.37E-06 | 0.000147 | 3.4338 |
| ABHD2 | -1.52736 | 13.05384 | -4.96961 | 7.40E-06 | 0.000148 | 3.42921 |
| DEPDC7 | 1.510879 | 13.04154 | 4.968904 | 7.42E-06 | 0.000148 | 3.426837 |
| RPL7P18 | -2.45999 | 12.65035 | -4.96826 | 7.44E-06 | 0.000148 | 3.424651 |
| MT-ND3 | 1.053595 | 13.19769 | 4.966912 | 7.47E-06 | 0.000149 | 3.420102 |
| ZDHHC1 | 1.5073 | 12.83445 | 4.964255 | 7.54E-06 | 0.00015 | 3.411121 |
| WDR89 | 1.098482 | 13.36386 | 4.964057 | 7.55E-06 | 0.00015 | 3.410451 |
| AC008026.1 | 2.23578 | 13.08035 | 4.96356 | 7.56E-06 | 0.00015 | 3.408773 |
| IL1RN | -1.32507 | 13.39296 | -4.96261 | 7.59E-06 | 0.00015 | 3.405576 |
| RPL7P1 | 1.686234 | 13.14082 | 4.959894 | 7.66E-06 | 0.000151 | 3.396386 |
| ASPH | -1.61336 | 13.11553 | -4.9587 | 7.69E-06 | 0.000152 | 3.392357 |
| NLRP12 | -1.01671 | 13.30831 | -4.95738 | 7.73E-06 | 0.000153 | 3.387895 |
| TRAV12-3 | 1.652848 | 13.05326 | 4.956124 | 7.76E-06 | 0.000153 | 3.383649 |
| TTC39C | 1.154709 | 13.25127 | 4.953454 | 7.83E-06 | 0.000154 | 3.374632 |
| ENTPD7 | -1.04286 | 13.28039 | -4.95229 | 7.87E-06 | 0.000155 | 3.370691 |
| NUDT11 | 1.682516 | 13.27713 | 4.951006 | 7.90E-06 | 0.000155 | 3.366365 |
| ADAMTSL4 | -1.20684 | 13.35175 | -4.95064 | 7.91E-06 | 0.000155 | 3.365112 |
| RPL22P24 | -1.37702 | 13.29092 | -4.95055 | 7.91E-06 | 0.000155 | 3.364837 |
| ZNF787 | -1.05178 | 13.24504 | -4.94993 | 7.93E-06 | 0.000155 | 3.36274 |
| RPS7P10 | 1.632128 | 13.62647 | 4.948328 | 7.98E-06 | 0.000156 | 3.357323 |
| DMKN | 1.25163 | 13.29634 | 4.947935 | 7.99E-06 | 0.000156 | 3.355996 |
| AL031985.2 | 1.973676 | 13.59299 | 4.947161 | 8.01E-06 | 0.000156 | 3.353384 |
| GPRC5D | -2.30447 | 12.84469 | -4.94713 | 8.01E-06 | 0.000156 | 3.353276 |
| AL645941.2 | 1.715375 | 13.10699 | 4.945555 | 8.05E-06 | 0.000157 | 3.347962 |
| AC009927.1 | -2.22679 | 13.03799 | -4.94306 | 8.12E-06 | 0.000158 | 3.339531 |
| LRATD2 | 1.557476 | 13.15499 | 4.94163 | 8.17E-06 | 0.000159 | 3.334718 |
| IGHV3OR16-9 | -1.98303 | 12.97062 | -4.94145 | 8.17E-06 | 0.000159 | 3.334118 |
| MST1L | 1.368453 | 13.06829 | 4.94143 | 8.17E-06 | 0.000159 | 3.334041 |
| ACVR2B | 1.329351 | 13.20118 | 4.940848 | 8.19E-06 | 0.000159 | 3.33208 |
| TRBV20-1 | 1.731122 | 12.89143 | 4.940438 | 8.20E-06 | 0.000159 | 3.330697 |
| LDHB | 1.070154 | 13.35656 | 4.937955 | 8.27E-06 | 0.00016 | 3.322319 |
| MTRNR2L11 | 1.467487 | 13.0373 | 4.936579 | 8.31E-06 | 0.000161 | 3.317676 |
| IGHV1OR15-9 | -2.5952 | 12.36968 | -4.93573 | 8.34E-06 | 0.000161 | 3.31483 |
| EEF1A1P17 | 1.843425 | 13.33773 | 4.935521 | 8.34E-06 | 0.000161 | 3.314108 |
| CALB1 | -1.11473 | 13.19801 | -4.93347 | 8.40E-06 | 0.000162 | 3.307185 |
| IGHV3-19 | -2.53973 | 12.72318 | -4.93312 | 8.41E-06 | 0.000162 | 3.30601 |
| IGKV2D-28 | -2.13433 | 12.75024 | -4.93033 | 8.50E-06 | 0.000163 | 3.29662 |
| TMEM204 | 1.582637 | 12.95363 | 4.930089 | 8.50E-06 | 0.000163 | 3.295793 |
| CAMK4 | 1.913241 | 13.04017 | 4.929231 | 8.53E-06 | 0.000163 | 3.2929 |
| RPS3AP25 | 1.890676 | 13.45831 | 4.928221 | 8.56E-06 | 0.000164 | 3.289493 |
| SLC7A4 | 1.917085 | 13.48502 | 4.925615 | 8.64E-06 | 0.000165 | 3.280709 |
| EIF4A2P1 | 1.921661 | 13.14035 | 4.924488 | 8.67E-06 | 0.000165 | 3.276912 |
| ZNF566 | 1.102663 | 13.33009 | 4.924335 | 8.68E-06 | 0.000165 | 3.276395 |
| SH3D19 | 1.211661 | 13.27706 | 4.923081 | 8.71E-06 | 0.000166 | 3.27217 |
| OXNAD1 | 1.252991 | 13.26435 | 4.922038 | 8.75E-06 | 0.000166 | 3.268657 |
| AC136616.2 | -2.47625 | 12.62548 | -4.9217 | 8.76E-06 | 0.000166 | 3.267533 |
| TNNT3 | -1.65209 | 13.36654 | -4.91965 | 8.82E-06 | 0.000167 | 3.260612 |
| C1QB | -2.29592 | 12.71612 | -4.91876 | 8.85E-06 | 0.000168 | 3.257597 |
| BCL11A | 1.01439 | 13.24558 | 4.917996 | 8.87E-06 | 0.000168 | 3.255039 |
| IGSF22 | 1.494313 | 13.09989 | 4.916616 | 8.91E-06 | 0.000169 | 3.250391 |
| ZNF548 | 1.151552 | 13.25503 | 4.916314 | 8.92E-06 | 0.000169 | 3.249374 |
| FEN1 | -1.08377 | 13.26053 | -4.9155 | 8.95E-06 | 0.000169 | 3.246622 |
| NPTXR | 1.492986 | 13.18664 | 4.914829 | 8.97E-06 | 0.000169 | 3.244374 |
| H3C3 | -1.95209 | 13.32789 | -4.9148 | 8.97E-06 | 0.000169 | 3.24428 |
| CLTCL1 | -1.4224 | 12.82826 | -4.91341 | 9.01E-06 | 0.00017 | 3.23958 |
| SMTNL1 | -2.88026 | 12.81933 | -4.91204 | 9.06E-06 | 0.00017 | 3.23497 |
| DHRS3 | 1.413107 | 13.11484 | 4.911714 | 9.07E-06 | 0.00017 | 3.233885 |
| MGLL | -1.3178 | 13.07563 | -4.9117 | 9.07E-06 | 0.00017 | 3.23382 |
| FFAR1 | -1.70267 | 13.23844 | -4.90928 | 9.15E-06 | 0.000171 | 3.225684 |
| ABCA1 | -1.6566 | 13.20175 | -4.90565 | 9.26E-06 | 0.000173 | 3.213474 |
| IGKV5-2 | -2.37758 | 12.37674 | -4.90558 | 9.26E-06 | 0.000173 | 3.213234 |
| INO80B-WBP1 | -5.03494 | 11.58001 | -4.90553 | 9.27E-06 | 0.000173 | 3.213068 |
| CGN | -1.45634 | 13.29044 | -4.90476 | 9.29E-06 | 0.000174 | 3.210464 |
| ESAM | -1.5528 | 13.12425 | -4.90454 | 9.30E-06 | 0.000174 | 3.20973 |
| PTX3 | -1.74753 | 12.60841 | -4.90327 | 9.34E-06 | 0.000174 | 3.205468 |
| STAB1 | -1.30377 | 13.25013 | -4.90321 | 9.34E-06 | 0.000174 | 3.205272 |
| IL5RA | 2.450519 | 12.21389 | 4.900303 | 9.44E-06 | 0.000176 | 3.195481 |
| PLA2G7 | 2.175691 | 12.68392 | 4.897553 | 9.53E-06 | 0.000177 | 3.186228 |
| ATP9A | -1.54881 | 13.05327 | -4.89387 | 9.65E-06 | 0.000179 | 3.173839 |
| LIMK2 | -1.19746 | 13.3948 | -4.89264 | 9.69E-06 | 0.000179 | 3.16971 |
| PFKFB3 | -1.57944 | 13.12219 | -4.89253 | 9.70E-06 | 0.000179 | 3.169355 |
| RPL13AP7 | 1.760796 | 13.1001 | 4.891957 | 9.72E-06 | 0.00018 | 3.167414 |
| DUSP14 | 1.165597 | 13.28775 | 4.891893 | 9.72E-06 | 0.00018 | 3.167198 |
| THEM4 | 1.065574 | 13.28887 | 4.890313 | 9.77E-06 | 0.00018 | 3.161885 |
| TRBV5-5 | 2.028135 | 12.80551 | 4.889949 | 9.79E-06 | 0.00018 | 3.160662 |
| IGKV1D-33 | -2.5818 | 12.71043 | -4.88994 | 9.79E-06 | 0.00018 | 3.160622 |
| TNFAIP6 | -1.72792 | 13.25162 | -4.88956 | 9.80E-06 | 0.000181 | 3.15936 |
| SLC9A8 | -1.16336 | 13.26942 | -4.88811 | 9.85E-06 | 0.000181 | 3.154492 |
| VEGFC | -1.63887 | 13.11122 | -4.88806 | 9.85E-06 | 0.000181 | 3.154318 |
| ABLIM3 | -1.61297 | 13.02319 | -4.88733 | 9.88E-06 | 0.000181 | 3.151858 |
| AC114728.1 | 2.176638 | 12.93911 | 4.887154 | 9.88E-06 | 0.000181 | 3.151267 |
| RPS4XP6 | 1.596834 | 13.659 | 4.884393 | 9.98E-06 | 0.000183 | 3.141988 |
| EEF1A1P24 | 1.815392 | 13.28926 | 4.884364 | 9.98E-06 | 0.000183 | 3.141891 |
| GOLGA7B | 1.482949 | 13.00271 | 4.883349 | 1.00E-05 | 0.000183 | 3.138481 |
| TRABD2A | 1.57541 | 13.05139 | 4.879873 | 1.01E-05 | 0.000185 | 3.126805 |
| ROBO3 | 1.574498 | 13.00873 | 4.879644 | 1.01E-05 | 0.000185 | 3.126036 |
| IGKV1D-16 | -3.19114 | 11.81547 | -4.87814 | 1.02E-05 | 0.000186 | 3.120969 |
| IGKV2D-30 | -3.2711 | 12.07277 | -4.87454 | 1.03E-05 | 0.000188 | 3.108896 |
| OR52W1 | -1.93433 | 13.35331 | -4.87396 | 1.03E-05 | 0.000188 | 3.106944 |
| CD3G | 1.715206 | 13.05866 | 4.870766 | 1.05E-05 | 0.00019 | 3.096224 |
| KPNA5 | 1.406153 | 13.27469 | 4.869286 | 1.05E-05 | 0.000191 | 3.091257 |
| AL158823.1 | 1.884188 | 13.22422 | 4.868749 | 1.05E-05 | 0.000191 | 3.089456 |
| SYT17 | 1.047789 | 13.09296 | 4.868374 | 1.06E-05 | 0.000191 | 3.088198 |
| UGT3A2 | -2.5812 | 12.23333 | -4.86694 | 1.06E-05 | 0.000192 | 3.083382 |
| MICU3 | 2.011405 | 13.12745 | 4.866862 | 1.06E-05 | 0.000192 | 3.083122 |
| MPZ | -1.13685 | 13.32042 | -4.86615 | 1.06E-05 | 0.000192 | 3.080732 |
| COL24A1 | -1.47415 | 13.1842 | -4.86443 | 1.07E-05 | 0.000193 | 3.074954 |
| CCNI2 | 1.304091 | 13.23047 | 4.861678 | 1.08E-05 | 0.000195 | 3.065732 |
| TRBC1 | 1.726436 | 12.98464 | 4.858619 | 1.09E-05 | 0.000196 | 3.055472 |
| AL592114.1 | 1.858253 | 13.17672 | 4.858299 | 1.09E-05 | 0.000196 | 3.054399 |
| SMTN | -1.33631 | 13.12926 | -4.85669 | 1.10E-05 | 0.000197 | 3.049003 |
| KLHDC1 | 1.27024 | 13.2574 | 4.856498 | 1.10E-05 | 0.000197 | 3.048359 |
| PHEX | 1.996483 | 12.83523 | 4.855782 | 1.10E-05 | 0.000198 | 3.045957 |
| TRBV3-1 | 1.871145 | 12.86338 | 4.853115 | 1.11E-05 | 0.000199 | 3.037018 |
| IGHV1OR15-2 | -2.48763 | 12.59701 | -4.85252 | 1.12E-05 | 0.000199 | 3.035032 |
| MSX2 | 1.987648 | 13.01835 | 4.852211 | 1.12E-05 | 0.0002 | 3.033986 |
| KCNK7 | -1.20764 | 13.33928 | -4.84987 | 1.13E-05 | 0.000201 | 3.026154 |
| MEF2B | -1.19596 | 13.18662 | -4.84976 | 1.13E-05 | 0.000201 | 3.025771 |
| IGHV3-71 | -2.32917 | 12.82553 | -4.84958 | 1.13E-05 | 0.000201 | 3.025175 |
| FAUP1 | 1.729689 | 13.17446 | 4.849065 | 1.13E-05 | 0.000201 | 3.023444 |
| RORA | 1.759012 | 13.07182 | 4.848829 | 1.13E-05 | 0.000201 | 3.022653 |
| KIFC3 | -1.29503 | 13.2793 | -4.8474 | 1.14E-05 | 0.000202 | 3.017856 |
| CCL28 | 1.205327 | 13.27967 | 4.845445 | 1.14E-05 | 0.000203 | 3.011314 |
| UBTD1 | -1.29389 | 13.1102 | -4.84391 | 1.15E-05 | 0.000204 | 3.006187 |
| PRKACB | 1.318401 | 13.28195 | 4.842134 | 1.16E-05 | 0.000205 | 3.000221 |
| CHCHD6 | 1.001343 | 13.2771 | 4.838882 | 1.17E-05 | 0.000207 | 2.989332 |
| DERL3 | -1.75935 | 13.05652 | -4.83843 | 1.17E-05 | 0.000207 | 2.987822 |
| SLC4A3 | 2.539196 | 12.40717 | 4.835949 | 1.18E-05 | 0.000209 | 2.979513 |
| PTGDR2 | 2.801432 | 11.95163 | 4.834853 | 1.19E-05 | 0.00021 | 2.975845 |
| ZNF549 | 1.07225 | 13.30491 | 4.834383 | 1.19E-05 | 0.00021 | 2.974271 |
| BEAN1 | -1.46741 | 13.25166 | -4.83383 | 1.19E-05 | 0.00021 | 2.972416 |
| SYCP2L | 1.857342 | 12.7575 | 4.83218 | 1.20E-05 | 0.000211 | 2.966896 |
| ZNF485 | 1.223144 | 13.29733 | 4.831258 | 1.20E-05 | 0.000212 | 2.963811 |
| EOMES | 1.585555 | 13.07913 | 4.830654 | 1.20E-05 | 0.000212 | 2.961792 |
| EPSTI1 | -1.85149 | 13.25893 | -4.83054 | 1.20E-05 | 0.000212 | 2.961404 |
| FAM110C | 1.448019 | 13.1867 | 4.829821 | 1.21E-05 | 0.000212 | 2.959002 |
| UPB1 | -1.72261 | 13.17234 | -4.82729 | 1.22E-05 | 0.000214 | 2.950538 |
| MTND4P30 | -1.26293 | 13.35385 | -4.82625 | 1.22E-05 | 0.000214 | 2.947046 |
| OCIAD2 | 1.074044 | 13.31331 | 4.826089 | 1.22E-05 | 0.000214 | 2.946518 |
| AL136295.5 | -1.23642 | 13.29 | -4.82191 | 1.24E-05 | 0.000217 | 2.932532 |
| SUSD4 | 1.871866 | 12.83807 | 4.820891 | 1.25E-05 | 0.000218 | 2.929134 |
| CALHM5 | -1.64205 | 13.10617 | -4.82074 | 1.25E-05 | 0.000218 | 2.928611 |
| ESRP1 | 1.817993 | 13.47092 | 4.818683 | 1.25E-05 | 0.000219 | 2.921749 |
| ZFP37 | 1.514638 | 13.24205 | 4.815103 | 1.27E-05 | 0.000221 | 2.909783 |
| GLS2 | 1.946978 | 13.02276 | 4.815024 | 1.27E-05 | 0.000221 | 2.909519 |
| CD8A | 1.710681 | 12.96433 | 4.814691 | 1.27E-05 | 0.000221 | 2.908405 |
| SCN9A | -1.2894 | 13.17794 | -4.81384 | 1.28E-05 | 0.000222 | 2.905548 |
| MMP1 | -2.97448 | 11.81229 | -4.81351 | 1.28E-05 | 0.000222 | 2.904471 |
| TRAV29DV5 | 1.921487 | 12.94536 | 4.811004 | 1.29E-05 | 0.000224 | 2.896084 |
| ZNF677 | 1.174246 | 13.33183 | 4.810605 | 1.29E-05 | 0.000224 | 2.894753 |
| ZNF420 | 1.138986 | 13.34871 | 4.809456 | 1.30E-05 | 0.000224 | 2.890915 |
| NPR2 | 1.280118 | 13.20068 | 4.807194 | 1.31E-05 | 0.000226 | 2.883357 |
| CD28 | 1.693537 | 13.07872 | 4.806569 | 1.31E-05 | 0.000226 | 2.881271 |
| RPL13AP25 | 1.778313 | 13.12144 | 4.806311 | 1.31E-05 | 0.000226 | 2.880407 |
| IGHV1-14 | -3.14362 | 11.85696 | -4.80472 | 1.32E-05 | 0.000227 | 2.875082 |
| UBE2Q2L | 2.149759 | 12.9076 | 4.804665 | 1.32E-05 | 0.000227 | 2.87491 |
| FUNDC2P2 | 1.395031 | 13.74837 | 4.803783 | 1.32E-05 | 0.000228 | 2.871966 |
| GLDN | -3.5369 | 11.56978 | -4.80303 | 1.32E-05 | 0.000228 | 2.869463 |
| ABLIM1 | 1.480126 | 13.18705 | 4.799114 | 1.34E-05 | 0.000231 | 2.856379 |
| VPS9D1 | -1.23053 | 13.27971 | -4.7988 | 1.34E-05 | 0.000231 | 2.855331 |
| ZNF491 | 1.161483 | 13.29703 | 4.798302 | 1.35E-05 | 0.000231 | 2.853667 |
| TMEM14A | 1.133144 | 13.30933 | 4.796037 | 1.36E-05 | 0.000233 | 2.846108 |
| POLR1E | 1.056555 | 13.3122 | 4.793191 | 1.37E-05 | 0.000235 | 2.83661 |
| CT75 | -1.55382 | 13.13369 | -4.79177 | 1.38E-05 | 0.000236 | 2.831883 |
| PRRT1 | 1.526752 | 12.98777 | 4.791513 | 1.38E-05 | 0.000236 | 2.831013 |
| AL365273.2 | -2.55847 | 12.9545 | -4.79062 | 1.38E-05 | 0.000236 | 2.828033 |
| NLGN2 | 1.471597 | 13.16647 | 4.789103 | 1.39E-05 | 0.000237 | 2.822973 |
| DPRXP4 | -1.62737 | 13.23811 | -4.78892 | 1.39E-05 | 0.000237 | 2.822365 |
| TRAJ36 | 2.01904 | 13.15569 | 4.787913 | 1.40E-05 | 0.000238 | 2.819006 |
| AKAP7 | 1.140629 | 13.25033 | 4.786082 | 1.41E-05 | 0.000239 | 2.812899 |
| AXIN2 | 1.834947 | 12.92472 | 4.784886 | 1.41E-05 | 0.000239 | 2.808911 |
| NOTCH1 | -1.42511 | 13.12592 | -4.78487 | 1.41E-05 | 0.000239 | 2.808874 |
| HLA-DOA | 1.415794 | 13.2053 | 4.782107 | 1.42E-05 | 0.000241 | 2.799648 |
| SORT1 | -1.22398 | 13.22618 | -4.78115 | 1.43E-05 | 0.000242 | 2.796472 |
| AL157938.1 | -1.73427 | 13.33991 | -4.78062 | 1.43E-05 | 0.000242 | 2.794693 |
| RPS4Y2 | 2.600915 | 12.6086 | 4.779989 | 1.44E-05 | 0.000242 | 2.792586 |
| TRBV11-1 | 2.284946 | 12.69309 | 4.778893 | 1.44E-05 | 0.000243 | 2.788934 |
| HEPH | -2.29252 | 12.68691 | -4.77833 | 1.44E-05 | 0.000243 | 2.787059 |
| PRKCH | 1.256607 | 13.2673 | 4.777025 | 1.45E-05 | 0.000244 | 2.78271 |
| CFLAR | -1.03174 | 13.40019 | -4.77445 | 1.46E-05 | 0.000246 | 2.774124 |
| NAIP | -1.20019 | 13.42834 | -4.77418 | 1.46E-05 | 0.000246 | 2.773239 |
| SPINK2 | 1.742325 | 12.94913 | 4.773581 | 1.47E-05 | 0.000247 | 2.771236 |
| ZDHHC19 | -3.90967 | 11.34982 | -4.7734 | 1.47E-05 | 0.000247 | 2.770644 |
| CHAC1 | -2.81209 | 12.40172 | -4.7698 | 1.49E-05 | 0.000249 | 2.758641 |
| ZFP14 | 1.024972 | 13.33521 | 4.768664 | 1.49E-05 | 0.000249 | 2.754858 |
| CD160 | 2.016983 | 12.86127 | 4.768252 | 1.49E-05 | 0.00025 | 2.753486 |
| FBXO16 | -1.90976 | 13.03173 | -4.76733 | 1.50E-05 | 0.00025 | 2.750425 |
| LCK | 1.452746 | 13.14092 | 4.766134 | 1.51E-05 | 0.000251 | 2.746435 |
| AUTS2 | 1.529414 | 13.13716 | 4.765814 | 1.51E-05 | 0.000251 | 2.745369 |
| RPL10P6 | 1.722026 | 13.55734 | 4.764269 | 1.52E-05 | 0.000252 | 2.740227 |
| KCNG2 | -2.53722 | 12.90133 | -4.7636 | 1.52E-05 | 0.000252 | 2.737983 |
| CAV2 | -1.44029 | 13.17541 | -4.76219 | 1.53E-05 | 0.000253 | 2.733303 |
| MYCL | 1.41118 | 13.07314 | 4.761142 | 1.53E-05 | 0.000254 | 2.72982 |
| PKIB | 1.318654 | 13.20716 | 4.760475 | 1.54E-05 | 0.000254 | 2.7276 |
| AMIGO1 | 1.802932 | 13.02716 | 4.759323 | 1.54E-05 | 0.000255 | 2.723766 |
| PPP1R3B | -1.07697 | 13.40554 | -4.75743 | 1.55E-05 | 0.000256 | 2.717451 |
| FAM3D | 2.048949 | 12.8184 | 4.756516 | 1.56E-05 | 0.000257 | 2.714427 |
| TRAV13-2 | 1.882103 | 12.96861 | 4.755456 | 1.56E-05 | 0.000258 | 2.710902 |
| B3GNT8 | -1.24357 | 13.26015 | -4.75476 | 1.57E-05 | 0.000258 | 2.708596 |
| TRIM6 | -1.88745 | 13.24153 | -4.75225 | 1.58E-05 | 0.00026 | 2.700254 |
| CCNA1 | -2.89114 | 12.30042 | -4.7498 | 1.59E-05 | 0.000262 | 2.692102 |
| AC008763.3 | -2.13484 | 12.71826 | -4.74925 | 1.60E-05 | 0.000262 | 2.690247 |
| ANKS6 | 1.207464 | 13.28403 | 4.749218 | 1.60E-05 | 0.000262 | 2.690158 |
| FCMR | 1.37362 | 13.2369 | 4.74742 | 1.61E-05 | 0.000263 | 2.68418 |
| WDR34 | -1.15346 | 12.93062 | -4.74666 | 1.61E-05 | 0.000264 | 2.68165 |
| IRAK1BP1 | 1.14058 | 13.31899 | 4.746634 | 1.61E-05 | 0.000264 | 2.681569 |
| CHURC1-FNTB | -2.60863 | 12.55931 | -4.746 | 1.61E-05 | 0.000264 | 2.679477 |
| AL662899.3 | -1.74239 | 13.01466 | -4.74371 | 1.63E-05 | 0.000266 | 2.671865 |
| TRAM2 | -1.2101 | 13.33835 | -4.74087 | 1.64E-05 | 0.000268 | 2.662424 |
| AC217785.1 | -1.01108 | 13.33784 | -4.73639 | 1.67E-05 | 0.000272 | 2.647543 |
| EEF1A1P1 | 1.53751 | 13.58474 | 4.735347 | 1.68E-05 | 0.000272 | 2.644069 |
| CEACAM1 | -1.67575 | 12.89872 | -4.73451 | 1.68E-05 | 0.000273 | 2.641299 |
| IGLV3-16 | -2.54581 | 12.68986 | -4.73331 | 1.69E-05 | 0.000274 | 2.637303 |
| RIPOR3 | -1.92458 | 11.27691 | -4.73252 | 1.69E-05 | 0.000275 | 2.634685 |
| UBASH3A | 1.599234 | 13.03138 | 4.732383 | 1.69E-05 | 0.000275 | 2.634228 |
| DENND11 | 1.342008 | 13.25377 | 4.730629 | 1.70E-05 | 0.000276 | 2.628403 |
| CD300LG | -1.56227 | 13.2819 | -4.73031 | 1.70E-05 | 0.000276 | 2.627335 |
| CKLF-CMTM1 | -1.28946 | 13.3425 | -4.72908 | 1.71E-05 | 0.000277 | 2.623269 |
| HAPLN3 | 1.447109 | 13.01651 | 4.728137 | 1.72E-05 | 0.000278 | 2.620133 |
| ZNF215 | -2.02081 | 12.99658 | -4.7279 | 1.72E-05 | 0.000278 | 2.619346 |
| ZFP82 | 1.090177 | 13.35198 | 4.727594 | 1.72E-05 | 0.000278 | 2.618332 |
| AC024940.3 | 1.370231 | 13.58315 | 4.726767 | 1.73E-05 | 0.000278 | 2.615588 |
| AC024940.9 | 1.370231 | 13.58315 | 4.726767 | 1.73E-05 | 0.000278 | 2.615588 |
| FAM27B | -1.96545 | 13.01562 | -4.72648 | 1.73E-05 | 0.000278 | 2.614624 |
| SYNPO | 1.178409 | 13.18207 | 4.726065 | 1.73E-05 | 0.000279 | 2.613255 |
| PSAT1 | -1.12487 | 13.26752 | -4.72595 | 1.73E-05 | 0.000279 | 2.612873 |
| ETS1 | 1.264689 | 13.2787 | 4.725591 | 1.73E-05 | 0.000279 | 2.611682 |
| STAT4 | 1.297309 | 13.26033 | 4.72394 | 1.74E-05 | 0.00028 | 2.606205 |
| TRAV22 | 1.571622 | 13.08886 | 4.72274 | 1.75E-05 | 0.000281 | 2.602226 |
| AC093512.2 | -1.67649 | 13.09234 | -4.7216 | 1.76E-05 | 0.000282 | 2.598445 |
| ZNF541 | 1.962046 | 13.10884 | 4.720739 | 1.76E-05 | 0.000282 | 2.595587 |
| MYL9 | -2.4724 | 12.47774 | -4.71879 | 1.77E-05 | 0.000284 | 2.589113 |
| AL590556.1 | 1.582379 | 13.74258 | 4.718475 | 1.78E-05 | 0.000284 | 2.58808 |
| PCSK9 | -4.93616 | 10.52472 | -4.71808 | 1.78E-05 | 0.000284 | 2.586757 |
| TNFRSF17 | -2.01676 | 13.00145 | -4.71739 | 1.78E-05 | 0.000285 | 2.584496 |
| FLT3LG | 1.306709 | 13.06377 | 4.715221 | 1.80E-05 | 0.000287 | 2.577292 |
| PTGR1 | -1.02444 | 13.06 | -4.71445 | 1.80E-05 | 0.000287 | 2.574745 |
| DAB1 | 2.337004 | 12.93489 | 4.713714 | 1.81E-05 | 0.000288 | 2.572294 |
| RPL13AP5 | 1.609931 | 13.11316 | 4.712667 | 1.81E-05 | 0.000289 | 2.568823 |
| ZXDB | 1.215811 | 13.30088 | 4.711899 | 1.82E-05 | 0.000289 | 2.566277 |
| TBC1D3P5 | -1.40926 | 13.14654 | -4.70913 | 1.83E-05 | 0.000292 | 2.557109 |
| IGLV2-18 | -2.43094 | 12.73929 | -4.70831 | 1.84E-05 | 0.000292 | 2.554398 |
| TRPC1 | 1.777507 | 13.05556 | 4.707269 | 1.85E-05 | 0.000293 | 2.550936 |
| IGKV3D-15 | -2.50382 | 12.68045 | -4.70629 | 1.85E-05 | 0.000294 | 2.547685 |
| EEF1A1P8 | 2.047035 | 12.99864 | 4.705611 | 1.86E-05 | 0.000294 | 2.545443 |
| IKZF2 | 1.812079 | 13.04566 | 4.70455 | 1.86E-05 | 0.000295 | 2.54193 |
| KRT18P34 | -1.11953 | 13.3484 | -4.70303 | 1.87E-05 | 0.000296 | 2.536892 |
| GPR15 | 2.207156 | 12.87346 | 4.701779 | 1.88E-05 | 0.000297 | 2.532754 |
| TARBP1 | 1.036114 | 13.29652 | 4.701624 | 1.88E-05 | 0.000297 | 2.53224 |
| ALPK3 | -1.6985 | 13.19101 | -4.70038 | 1.89E-05 | 0.000298 | 2.528131 |
| PLEKHG4 | 1.036134 | 13.21845 | 4.698553 | 1.90E-05 | 0.0003 | 2.522071 |
| TRAV4 | 1.747688 | 12.9122 | 4.695935 | 1.92E-05 | 0.000302 | 2.513407 |
| ADHFE1 | 1.13336 | 13.147 | 4.694014 | 1.93E-05 | 0.000303 | 2.507047 |
| EIF1AXP1 | -1.56191 | 13.16139 | -4.69283 | 1.94E-05 | 0.000305 | 2.503125 |
| TMEM132D | -1.66015 | 13.22297 | -4.69243 | 1.94E-05 | 0.000305 | 2.501796 |
| JUNB | -1.10948 | 13.24287 | -4.69115 | 1.95E-05 | 0.000306 | 2.497563 |
| ANPEP | -1.09126 | 13.2713 | -4.69021 | 1.96E-05 | 0.000307 | 2.494477 |
| ZC3H12B | 1.126785 | 13.27484 | 4.689862 | 1.96E-05 | 0.000307 | 2.493309 |
| REXO5 | -1.02006 | 12.8563 | -4.6895 | 1.96E-05 | 0.000307 | 2.492101 |
| IGLV7-46 | -2.40218 | 12.4899 | -4.68858 | 1.97E-05 | 0.000308 | 2.489057 |
| SMAD1 | -1.24132 | 13.25409 | -4.6883 | 1.97E-05 | 0.000308 | 2.488136 |
| LRWD1 | -1.01328 | 13.3524 | -4.68511 | 1.99E-05 | 0.000311 | 2.477592 |
| RHOBTB1 | -1.46039 | 13.1392 | -4.68355 | 2.00E-05 | 0.000312 | 2.472421 |
| CLEC1B | -1.36142 | 13.19266 | -4.68339 | 2.00E-05 | 0.000312 | 2.4719 |
| TSPYL5 | 1.401281 | 13.19253 | 4.682277 | 2.01E-05 | 0.000313 | 2.468226 |
| CNR2 | 1.290846 | 13.1845 | 4.682126 | 2.01E-05 | 0.000313 | 2.467726 |
| AL161909.1 | 1.273309 | 14.20946 | 4.680683 | 2.02E-05 | 0.000315 | 2.462956 |
| HELZ2 | -1.4095 | 13.30148 | -4.6794 | 2.03E-05 | 0.000316 | 2.458719 |
| LDB2 | 1.80351 | 13.06962 | 4.678025 | 2.04E-05 | 0.000317 | 2.454171 |
| TTC39B | 1.082012 | 13.30045 | 4.677448 | 2.05E-05 | 0.000317 | 2.452265 |
| DENND3 | -1.07674 | 13.30259 | -4.67649 | 2.05E-05 | 0.000317 | 2.449103 |
| SIGLEC8 | 2.763002 | 11.91973 | 4.674985 | 2.06E-05 | 0.000319 | 2.444127 |
| GAS7 | -1.07844 | 13.31016 | -4.67487 | 2.06E-05 | 0.000319 | 2.443733 |
| EGFL7 | -1.80321 | 12.96596 | -4.67413 | 2.07E-05 | 0.000319 | 2.441318 |
| LILRB5 | -1.02261 | 13.23149 | -4.67288 | 2.08E-05 | 0.000321 | 2.437169 |
| KRT80 | -1.26308 | 13.33834 | -4.67193 | 2.09E-05 | 0.000321 | 2.434024 |
| KIAA1958 | -1.51402 | 13.34189 | -4.67089 | 2.09E-05 | 0.000322 | 2.430594 |
| FAM117B | 1.157278 | 13.31672 | 4.670444 | 2.10E-05 | 0.000322 | 2.429126 |
| TRAJ26 | 2.208329 | 12.71293 | 4.669475 | 2.10E-05 | 0.000323 | 2.425926 |
| SMOX | -1.49635 | 12.71018 | -4.668 | 2.11E-05 | 0.000325 | 2.421063 |
| TRAV5 | 1.940665 | 12.8134 | 4.666563 | 2.12E-05 | 0.000326 | 2.416312 |
| KLHL30 | -1.20103 | 13.41262 | -4.66654 | 2.12E-05 | 0.000326 | 2.416232 |
| CCR9 | 1.649798 | 13.0976 | 4.66352 | 2.15E-05 | 0.000329 | 2.406268 |
| TRAV36DV7 | 1.625482 | 12.94784 | 4.663137 | 2.15E-05 | 0.000329 | 2.405002 |
| AC104619.1 | 1.665937 | 13.78976 | 4.663117 | 2.15E-05 | 0.000329 | 2.404938 |
| EHD2 | -1.10464 | 13.06355 | -4.66263 | 2.15E-05 | 0.000329 | 2.403325 |
| EIF5A2 | 1.033446 | 13.27335 | 4.661032 | 2.17E-05 | 0.00033 | 2.398056 |
| AC139491.5 | 1.109592 | 13.23261 | 4.658026 | 2.19E-05 | 0.000333 | 2.388139 |
| IGHV7-81 | -3.1869 | 11.25882 | -4.65683 | 2.20E-05 | 0.000334 | 2.384186 |
| KLHL31 | 1.544337 | 13.20498 | 4.655755 | 2.20E-05 | 0.000335 | 2.380646 |
| DTX3 | 1.268748 | 13.19771 | 4.655721 | 2.20E-05 | 0.000335 | 2.380535 |
| AC090589.1 | 2.085336 | 13.11166 | 4.650122 | 2.25E-05 | 0.00034 | 2.362074 |
| TRAJ30 | 2.341563 | 12.9168 | 4.649654 | 2.25E-05 | 0.000341 | 2.36053 |
| IGKV3D-20 | -2.38745 | 12.26461 | -4.64764 | 2.27E-05 | 0.000343 | 2.353878 |
| GGT7 | 1.055444 | 13.20763 | 4.645465 | 2.28E-05 | 0.000345 | 2.346721 |
| TBC1D19 | 1.083715 | 13.31918 | 4.645062 | 2.29E-05 | 0.000345 | 2.345395 |
| GIMAP7 | 1.210863 | 13.30653 | 4.642909 | 2.30E-05 | 0.000347 | 2.338299 |
| OMG | -1.13528 | 13.18974 | -4.64227 | 2.31E-05 | 0.000348 | 2.336201 |
| AC069431.2 | -2.40862 | 12.99935 | -4.64071 | 2.32E-05 | 0.000349 | 2.331057 |
| TIGD3 | 1.537518 | 12.75026 | 4.640543 | 2.32E-05 | 0.000349 | 2.330508 |
| LY6G6F | -1.61514 | 12.93637 | -4.64034 | 2.32E-05 | 0.000349 | 2.329836 |
| LAT | 1.000861 | 13.22897 | 4.640262 | 2.33E-05 | 0.000349 | 2.329579 |
| DDX60L | -1.31939 | 13.35964 | -4.63841 | 2.34E-05 | 0.000351 | 2.323485 |
| TNFRSF13B | -1.74017 | 13.11553 | -4.63349 | 2.38E-05 | 0.000356 | 2.307283 |
| CAMK2D | 1.142615 | 13.32768 | 4.633484 | 2.38E-05 | 0.000356 | 2.307262 |
| TRBV15 | 1.682079 | 12.94604 | 4.632149 | 2.39E-05 | 0.000357 | 2.302868 |
| AC068050.1 | 2.714363 | 12.3295 | 4.63169 | 2.39E-05 | 0.000358 | 2.301355 |
| LRRC4 | -1.06869 | 13.27238 | -4.63131 | 2.40E-05 | 0.000358 | 2.30012 |
| IGKV1D-37 | -2.20896 | 12.50573 | -4.63126 | 2.40E-05 | 0.000358 | 2.299942 |
| GADD45G | -1.88774 | 12.78661 | -4.63092 | 2.40E-05 | 0.000358 | 2.298839 |
| STRIP2 | -1.01612 | 12.94335 | -4.63021 | 2.41E-05 | 0.000358 | 2.29647 |
| MAP1LC3B2 | -1.36769 | 13.2256 | -4.6302 | 2.41E-05 | 0.000358 | 2.296439 |
| KIAA1614 | -1.39254 | 13.34662 | -4.62955 | 2.41E-05 | 0.000358 | 2.294321 |
| AC114744.1 | -1.29451 | 13.07447 | -4.62737 | 2.43E-05 | 0.00036 | 2.28714 |
| RPL9P28 | 1.698623 | 13.33249 | 4.626983 | 2.43E-05 | 0.000361 | 2.285867 |
| RPL23AP74 | 1.115575 | 13.17663 | 4.626869 | 2.43E-05 | 0.000361 | 2.285493 |
| TRPM1 | -1.53356 | 13.42717 | -4.62209 | 2.48E-05 | 0.000365 | 2.26979 |
| XKR4 | -1.51404 | 13.31079 | -4.62189 | 2.48E-05 | 0.000365 | 2.269131 |
| TRAJ16 | 2.118644 | 12.83937 | 4.621813 | 2.48E-05 | 0.000365 | 2.268862 |
| TDRD6 | 2.334855 | 12.57001 | 4.619747 | 2.50E-05 | 0.000367 | 2.262069 |
| MAL | 1.669406 | 12.83182 | 4.619421 | 2.50E-05 | 0.000367 | 2.260998 |
| ALDH1A1 | 1.769839 | 12.93296 | 4.618714 | 2.50E-05 | 0.000368 | 2.258674 |
| GJB5 | -1.85879 | 13.11016 | -4.61831 | 2.51E-05 | 0.000368 | 2.25734 |
| TCN2 | -1.94351 | 13.0259 | -4.61794 | 2.51E-05 | 0.000368 | 2.256129 |
| PRDM6 | -1.61905 | 13.05887 | -4.61573 | 2.53E-05 | 0.000371 | 2.248877 |
| PAQR6 | -1.65014 | 12.9699 | -4.61415 | 2.54E-05 | 0.000373 | 2.243675 |
| KRTAP29-1 | 1.850038 | 13.15721 | 4.613565 | 2.55E-05 | 0.000373 | 2.241749 |
| AC010469.2 | 1.320959 | 14.38209 | 4.613195 | 2.55E-05 | 0.000374 | 2.240535 |
| ARID3BP1 | -2.54715 | 12.32372 | -4.61266 | 2.56E-05 | 0.000374 | 2.238763 |
| HMGB1P9 | -1.75261 | 13.31391 | -4.61092 | 2.57E-05 | 0.000376 | 2.233071 |
| MTCO1P11 | -1.86978 | 13.09261 | -4.6107 | 2.57E-05 | 0.000376 | 2.23233 |
| DLG2 | 1.262697 | 13.34462 | 4.609142 | 2.59E-05 | 0.000377 | 2.227218 |
| RPL21P75 | 3.753825 | 11.92661 | 4.609106 | 2.59E-05 | 0.000377 | 2.227101 |
| ASIP | 1.854754 | 13.25603 | 4.607829 | 2.60E-05 | 0.000379 | 2.222904 |
| PRSS33 | 5.072791 | 10.07994 | 4.605251 | 2.62E-05 | 0.000382 | 2.214441 |
| CTSO | 1.170042 | 13.29352 | 4.603718 | 2.64E-05 | 0.000383 | 2.209406 |
| CD209 | 1.547166 | 12.95092 | 4.60354 | 2.64E-05 | 0.000384 | 2.208822 |
| S1PR1 | 1.171392 | 13.32015 | 4.603074 | 2.64E-05 | 0.000384 | 2.207292 |
| RPS27 | 1.124139 | 13.19008 | 4.602205 | 2.65E-05 | 0.000385 | 2.204439 |
| ARFGEF3 | -1.37595 | 12.94845 | -4.59816 | 2.69E-05 | 0.00039 | 2.191175 |
| AC010332.3 | 1.314842 | 13.11242 | 4.598055 | 2.69E-05 | 0.00039 | 2.19082 |
| AC015911.7 | 1.532018 | 13.10905 | 4.597978 | 2.69E-05 | 0.00039 | 2.190568 |
| NQO2 | -1.17899 | 13.09137 | -4.59649 | 2.70E-05 | 0.000392 | 2.185686 |
| WFDC1 | -1.87984 | 12.80532 | -4.59622 | 2.70E-05 | 0.000392 | 2.184809 |
| CD2 | 1.492898 | 13.13557 | 4.595956 | 2.71E-05 | 0.000392 | 2.183932 |
| ARHGEF4 | 1.310913 | 13.1146 | 4.59422 | 2.72E-05 | 0.000393 | 2.178239 |
| IGKV2-24 | -2.38847 | 12.58691 | -4.59421 | 2.72E-05 | 0.000393 | 2.178202 |
| LINGO3 | -1.62054 | 13.06168 | -4.59405 | 2.72E-05 | 0.000393 | 2.177687 |
| GPT2 | -1.10584 | 13.41634 | -4.59324 | 2.73E-05 | 0.000394 | 2.175007 |
| GLT1D1 | -1.07574 | 13.2516 | -4.59121 | 2.75E-05 | 0.000396 | 2.168379 |
| USP44 | 1.855149 | 13.05439 | 4.591069 | 2.75E-05 | 0.000396 | 2.167903 |
| APLP1 | -1.49889 | 13.26384 | -4.59091 | 2.75E-05 | 0.000396 | 2.167394 |
| ZNF550 | 1.160853 | 13.29301 | 4.589547 | 2.77E-05 | 0.000397 | 2.162913 |
| TRAV8-2 | 1.963473 | 12.85429 | 4.583934 | 2.82E-05 | 0.000404 | 2.144513 |
| TCTEX1D4 | -2.16742 | 12.94486 | -4.58364 | 2.82E-05 | 0.000404 | 2.143538 |
| RBMS1P1 | -2.29715 | 12.87206 | -4.58269 | 2.83E-05 | 0.000405 | 2.140438 |
| ZCCHC18 | 1.223494 | 13.26278 | 4.582547 | 2.83E-05 | 0.000405 | 2.139969 |
| FBXO7 | 1.504122 | 11.95778 | 4.580305 | 2.86E-05 | 0.000407 | 2.132621 |
| SIAH3 | 1.352628 | 13.12862 | 4.580261 | 2.86E-05 | 0.000407 | 2.132478 |
| AL353625.1 | -1.11379 | 13.33934 | -4.5799 | 2.86E-05 | 0.000408 | 2.131306 |
| TLR2 | -1.1369 | 13.36745 | -4.57625 | 2.90E-05 | 0.000412 | 2.119328 |
| PTPN4 | 1.36551 | 13.18389 | 4.575914 | 2.90E-05 | 0.000412 | 2.11824 |
| GJD3 | -1.81076 | 13.17233 | -4.57533 | 2.90E-05 | 0.000413 | 2.116337 |
| FLT4 | 1.480131 | 13.13082 | 4.574932 | 2.91E-05 | 0.000413 | 2.115023 |
| AC098583.1 | 1.655786 | 13.36537 | 4.573505 | 2.92E-05 | 0.000415 | 2.110351 |
| GLULP5 | -1.73806 | 13.15794 | -4.57254 | 2.93E-05 | 0.000416 | 2.107208 |
| ETNK2 | 1.534057 | 13.19381 | 4.57075 | 2.95E-05 | 0.000418 | 2.101331 |
| CMYA5 | -1.28275 | 13.32971 | -4.57029 | 2.96E-05 | 0.000418 | 2.099813 |
| AGGF1P8 | -2.15367 | 13.17141 | -4.56859 | 2.97E-05 | 0.00042 | 2.094265 |
| ZNF418 | 1.028278 | 13.33428 | 4.568253 | 2.98E-05 | 0.000421 | 2.093159 |
| PARD3 | -1.40986 | 13.09873 | -4.56757 | 2.98E-05 | 0.000421 | 2.090917 |
| NMU | 1.806715 | 12.74152 | 4.567149 | 2.99E-05 | 0.000422 | 2.089547 |
| ZNF703 | 2.288607 | 12.23047 | 4.566366 | 3.00E-05 | 0.000423 | 2.086986 |
| RPL7AP30 | 1.498164 | 13.21503 | 4.563005 | 3.03E-05 | 0.000427 | 2.075992 |
| ADGRE4P | 2.616585 | 12.27431 | 4.562863 | 3.03E-05 | 0.000427 | 2.075527 |
| CD70 | -1.43028 | 13.27545 | -4.56286 | 3.03E-05 | 0.000427 | 2.075508 |
| A2MP1 | 1.689735 | 12.99097 | 4.56088 | 3.05E-05 | 0.000429 | 2.06904 |
| TRBV4-2 | 1.92832 | 12.81056 | 4.558883 | 3.07E-05 | 0.000431 | 2.062512 |
| GRIP1 | 1.257659 | 13.23274 | 4.558726 | 3.07E-05 | 0.000431 | 2.061997 |
| PABPC1P7 | 1.839842 | 13.03619 | 4.558388 | 3.08E-05 | 0.000431 | 2.060892 |
| CNTNAP2 | 2.050408 | 12.80957 | 4.558047 | 3.08E-05 | 0.000432 | 2.059777 |
| RPSAP54 | 2.230429 | 13.00768 | 4.556294 | 3.10E-05 | 0.000434 | 2.054046 |
| BTG1P1 | 2.418168 | 12.61615 | 4.556153 | 3.10E-05 | 0.000434 | 2.053585 |
| RPS7P11 | 1.693074 | 13.14084 | 4.553787 | 3.13E-05 | 0.000437 | 2.045853 |
| CPNE5 | -1.10086 | 13.35378 | -4.55319 | 3.13E-05 | 0.000437 | 2.04391 |
| TRBV7-7 | 2.416913 | 12.62847 | 4.553051 | 3.13E-05 | 0.000437 | 2.043449 |
| IGLVI-70 | -3.05105 | 11.62972 | -4.55175 | 3.15E-05 | 0.000439 | 2.039205 |
| TRAJ39 | 2.194415 | 12.8903 | 4.5483 | 3.19E-05 | 0.000444 | 2.027928 |
| TRAV27 | 1.733604 | 12.84321 | 4.548193 | 3.19E-05 | 0.000444 | 2.027576 |
| RASGRF2 | 1.747669 | 13.08387 | 4.547693 | 3.19E-05 | 0.000444 | 2.025943 |
| EPHA2 | 2.952061 | 12.2753 | 4.545926 | 3.21E-05 | 0.000446 | 2.020171 |
| ESYT3 | -1.60279 | 13.24124 | -4.54513 | 3.22E-05 | 0.000447 | 2.017575 |
| PYGL | -1.30942 | 13.0897 | -4.54485 | 3.22E-05 | 0.000447 | 2.01666 |
| NPAS2 | 1.487699 | 13.16795 | 4.542584 | 3.25E-05 | 0.00045 | 2.00926 |
| TRAJ6 | 1.918757 | 13.10758 | 4.542157 | 3.25E-05 | 0.00045 | 2.007866 |
| C1QTNF7 | -1.77158 | 13.26205 | -4.54105 | 3.27E-05 | 0.000451 | 2.004253 |
| WDR49 | 1.531556 | 12.88667 | 4.539576 | 3.28E-05 | 0.000453 | 1.999442 |
| SPTLC2 | -1.01388 | 13.39455 | -4.53909 | 3.29E-05 | 0.000454 | 1.997848 |
| LY9 | 1.346548 | 13.21412 | 4.537172 | 3.31E-05 | 0.000457 | 1.991596 |
| TCF7 | 1.835385 | 12.93342 | 4.536555 | 3.32E-05 | 0.000457 | 1.989583 |
| TRIM71 | -1.6694 | 13.18424 | -4.53649 | 3.32E-05 | 0.000457 | 1.989373 |
| GIMAP5 | 1.300415 | 13.20605 | 4.536016 | 3.32E-05 | 0.000458 | 1.987824 |
| NAP1L3 | 1.650141 | 13.0105 | 4.535266 | 3.33E-05 | 0.000459 | 1.985378 |
| CU151838.2 | -1.88846 | 13.2106 | -4.53086 | 3.38E-05 | 0.000465 | 1.971016 |
| MMP28 | 1.775011 | 13.02786 | 4.530435 | 3.39E-05 | 0.000465 | 1.969623 |
| EEF1A1P38 | 1.527698 | 13.32576 | 4.528531 | 3.41E-05 | 0.000468 | 1.963414 |
| MIOX | -1.40894 | 13.2005 | -4.52618 | 3.44E-05 | 0.000471 | 1.95575 |
| PDLIM7 | -1.18132 | 13.16101 | -4.52575 | 3.44E-05 | 0.000471 | 1.954346 |
| PI16 | 1.755347 | 12.82905 | 4.523689 | 3.46E-05 | 0.000473 | 1.94763 |
| CABLES1 | -1.1338 | 13.22018 | -4.52246 | 3.48E-05 | 0.000475 | 1.943618 |
| ITGB3 | -1.69284 | 12.88221 | -4.52243 | 3.48E-05 | 0.000475 | 1.94354 |
| GRB10 | -2.24566 | 12.59091 | -4.52165 | 3.49E-05 | 0.000475 | 1.940981 |
| CD5 | 1.502748 | 13.05347 | 4.520673 | 3.50E-05 | 0.000477 | 1.937805 |
| ACVRL1 | -1.21468 | 13.35216 | -4.51968 | 3.51E-05 | 0.000478 | 1.934584 |
| TRBV10-3 | 2.06564 | 12.7276 | 4.518986 | 3.52E-05 | 0.000479 | 1.93231 |
| RASGRP1 | 1.433053 | 13.1775 | 4.517816 | 3.53E-05 | 0.000481 | 1.928498 |
| NACA4P | 1.057849 | 13.3823 | 4.517131 | 3.54E-05 | 0.000482 | 1.926268 |
| GALNT8 | 1.482486 | 13.0541 | 4.516887 | 3.55E-05 | 0.000482 | 1.925471 |
| CHIC1 | 1.437324 | 13.26431 | 4.516019 | 3.56E-05 | 0.000483 | 1.922647 |
| FCER1G | -1.0071 | 13.22855 | -4.51428 | 3.58E-05 | 0.000485 | 1.916978 |
| IGLV2-8 | -2.93573 | 12.0602 | -4.51351 | 3.59E-05 | 0.000486 | 1.91449 |
| LEF1 | 1.720666 | 13.04528 | 4.512017 | 3.61E-05 | 0.000488 | 1.909617 |
| AMPD1 | -2.37856 | 12.8413 | -4.50861 | 3.65E-05 | 0.000492 | 1.898529 |
| CHI3L2 | 1.2492 | 13.20078 | 4.508486 | 3.65E-05 | 0.000492 | 1.898124 |
| FGFBP1 | 1.230235 | 14.13103 | 4.504875 | 3.69E-05 | 0.000498 | 1.886378 |
| LAMB3 | -1.33665 | 12.78111 | -4.50473 | 3.70E-05 | 0.000498 | 1.885903 |
| FAM83D | -1.17413 | 12.7499 | -4.50409 | 3.70E-05 | 0.000498 | 1.883824 |
| THEMIS | 1.798435 | 13.07246 | 4.501842 | 3.73E-05 | 0.000502 | 1.876511 |
| TRBV7-2 | 1.833716 | 12.84263 | 4.501484 | 3.74E-05 | 0.000502 | 1.875348 |
| GJA3 | 1.720287 | 13.02687 | 4.500554 | 3.75E-05 | 0.000503 | 1.872324 |
| ZNF887P | -1.73466 | 12.88971 | -4.49977 | 3.76E-05 | 0.000504 | 1.86978 |
| IGLV4-3 | -2.52607 | 12.64041 | -4.49941 | 3.76E-05 | 0.000504 | 1.868603 |
| HIP1 | -1.07581 | 13.27066 | -4.49881 | 3.77E-05 | 0.000505 | 1.866664 |
| PMCHL2 | -1.33771 | 13.35491 | -4.49839 | 3.78E-05 | 0.000505 | 1.865296 |
| TPT1P9 | 2.006545 | 12.91703 | 4.496548 | 3.80E-05 | 0.000508 | 1.859302 |
| SOX8 | 2.097592 | 12.84763 | 4.495614 | 3.81E-05 | 0.000509 | 1.856266 |
| WNT11 | -1.55244 | 13.24261 | -4.49536 | 3.82E-05 | 0.000509 | 1.855434 |
| TRBV5-6 | 1.715755 | 12.98702 | 4.495324 | 3.82E-05 | 0.000509 | 1.855324 |
| ACE2 | -1.88458 | 13.20256 | -4.4953 | 3.82E-05 | 0.000509 | 1.85525 |
| EMILIN1 | -1.16018 | 13.12683 | -4.49525 | 3.82E-05 | 0.000509 | 1.855092 |
| IGHV2-5 | -2.92216 | 11.91771 | -4.49442 | 3.83E-05 | 0.00051 | 1.852393 |
| BNIP5 | -2.21197 | 13.10239 | -4.49423 | 3.83E-05 | 0.00051 | 1.851758 |
| HLA-DQA1 | 1.156109 | 13.12408 | 4.494128 | 3.83E-05 | 0.00051 | 1.851436 |
| SUGCT | 1.263157 | 13.32894 | 4.493838 | 3.83E-05 | 0.000511 | 1.850494 |
| FGF13 | -1.08965 | 13.2944 | -4.49354 | 3.84E-05 | 0.000511 | 1.849523 |
| ARSJ | 1.963731 | 13.31988 | 4.493221 | 3.84E-05 | 0.000511 | 1.84849 |
| ZBTB25 | 1.067233 | 13.29091 | 4.492783 | 3.85E-05 | 0.000512 | 1.847065 |
| IGHV3-41 | -2.31942 | 12.4451 | -4.49252 | 3.85E-05 | 0.000512 | 1.846203 |
| AKR1C1 | -1.26893 | 12.97493 | -4.49161 | 3.86E-05 | 0.000513 | 1.843253 |
| AC010627.1 | -1.81055 | 13.03573 | -4.4895 | 3.89E-05 | 0.000515 | 1.836414 |
| KLF5 | -1.29316 | 12.96414 | -4.48943 | 3.89E-05 | 0.000515 | 1.836163 |
| MAF | 1.145738 | 13.23251 | 4.488448 | 3.91E-05 | 0.000516 | 1.832983 |
| AC209007.1 | 1.677542 | 13.61934 | 4.488169 | 3.91E-05 | 0.000517 | 1.83208 |
| C11orf45 | 1.668074 | 13.0022 | 4.487095 | 3.92E-05 | 0.000518 | 1.828591 |
| PRICKLE4 | -1.27344 | 13.08332 | -4.48638 | 3.93E-05 | 0.000518 | 1.826282 |
| AC099489.1 | -1.52747 | 13.1829 | -4.48632 | 3.93E-05 | 0.000518 | 1.826088 |
| CLIC6 | 1.682862 | 13.269 | 4.486176 | 3.94E-05 | 0.000518 | 1.825605 |
| RAB42 | -1.30751 | 13.27266 | -4.48607 | 3.94E-05 | 0.000518 | 1.825275 |
| IGHV3-60 | -2.16436 | 12.36978 | -4.48603 | 3.94E-05 | 0.000518 | 1.825128 |
| KLHL2 | -1.15796 | 13.31001 | -4.48479 | 3.95E-05 | 0.00052 | 1.8211 |
| FEZ1 | 1.394469 | 13.24971 | 4.483322 | 3.97E-05 | 0.000522 | 1.81634 |
| ALOX5 | -1.16825 | 13.23369 | -4.48263 | 3.98E-05 | 0.000523 | 1.814103 |
| ZNF354C | 1.356047 | 13.27336 | 4.480249 | 4.02E-05 | 0.000526 | 1.806368 |
| THEM5 | 1.211476 | 12.78963 | 4.479912 | 4.02E-05 | 0.000527 | 1.805273 |
| RAB19 | -1.62667 | 12.89098 | -4.4785 | 4.04E-05 | 0.000529 | 1.800703 |
| CSNK1G2P1 | -1.56428 | 13.35323 | -4.47834 | 4.04E-05 | 0.000529 | 1.800164 |
| DMRTC1B | 1.84549 | 12.96825 | 4.475326 | 4.08E-05 | 0.000533 | 1.790397 |
| KL | -1.47481 | 13.25219 | -4.47491 | 4.09E-05 | 0.000533 | 1.789036 |
| TRAV8-6 | 1.762619 | 12.91961 | 4.473989 | 4.10E-05 | 0.000534 | 1.786059 |
| RPSAP61 | 1.836314 | 13.26892 | 4.472683 | 4.12E-05 | 0.000536 | 1.781825 |
| TRAV14DV4 | 1.641028 | 13.03275 | 4.47163 | 4.14E-05 | 0.000537 | 1.778409 |
| CNBD1 | -1.29219 | 13.3136 | -4.4715 | 4.14E-05 | 0.000537 | 1.777973 |
| PROB1 | -1.16409 | 13.32913 | -4.46605 | 4.21E-05 | 0.000546 | 1.760333 |
| IL32 | 1.238328 | 13.1242 | 4.466042 | 4.21E-05 | 0.000546 | 1.760298 |
| GPR171 | 1.495362 | 13.13999 | 4.465985 | 4.22E-05 | 0.000546 | 1.760111 |
| IGHG4 | -2.17034 | 12.50051 | -4.46546 | 4.22E-05 | 0.000546 | 1.758425 |
| JDP2 | -1.35666 | 13.12669 | -4.46458 | 4.24E-05 | 0.000547 | 1.755543 |
| PRR5L | 1.181003 | 13.13311 | 4.463391 | 4.25E-05 | 0.000549 | 1.751707 |
| ISM1 | 2.280583 | 12.57303 | 4.463061 | 4.26E-05 | 0.000549 | 1.750637 |
| TRAJ5 | 2.151804 | 12.95159 | 4.460721 | 4.29E-05 | 0.000553 | 1.743058 |
| AC138866.1 | 4.097633 | 10.43877 | 4.45812 | 4.33E-05 | 0.000556 | 1.734634 |
| NR3C2 | 1.805199 | 13.07377 | 4.457968 | 4.33E-05 | 0.000556 | 1.734144 |
| TRIM55 | -1.84102 | 13.25443 | -4.45461 | 4.38E-05 | 0.000562 | 1.723264 |
| TRBV6-1 | 1.870274 | 12.88378 | 4.451914 | 4.42E-05 | 0.000566 | 1.714545 |
| PRR26 | -1.93955 | 13.0744 | -4.45166 | 4.42E-05 | 0.000566 | 1.713736 |
| NOS2P3 | -1.43194 | 13.05507 | -4.45112 | 4.43E-05 | 0.000567 | 1.711967 |
| TENT5C | -1.33665 | 12.74815 | -4.45091 | 4.44E-05 | 0.000567 | 1.711301 |
| SH3TC2 | -1.45528 | 13.11669 | -4.45029 | 4.45E-05 | 0.000568 | 1.709288 |
| RPS3AP6 | 2.714899 | 12.48154 | 4.449545 | 4.46E-05 | 0.000569 | 1.70688 |
| TRAV20 | 1.700688 | 12.97783 | 4.449124 | 4.46E-05 | 0.000569 | 1.705519 |
| EVL | 1.152141 | 13.17212 | 4.443359 | 4.55E-05 | 0.000578 | 1.686875 |
| H1-4 | -1.62586 | 13.31913 | -4.44335 | 4.55E-05 | 0.000578 | 1.68684 |
| ITGA1 | -1.29057 | 13.27702 | -4.4421 | 4.57E-05 | 0.00058 | 1.682793 |
| PLOD2 | -1.23818 | 13.06391 | -4.44151 | 4.58E-05 | 0.000581 | 1.680881 |
| BEND7 | -1.64913 | 13.09775 | -4.44051 | 4.59E-05 | 0.000582 | 1.677673 |
| DNMT3B | -1.00208 | 13.31807 | -4.4387 | 4.62E-05 | 0.000585 | 1.671821 |
| TRAV16 | 1.751442 | 12.95267 | 4.438463 | 4.63E-05 | 0.000585 | 1.671048 |
| TRAJ31 | 2.030576 | 12.99854 | 4.438119 | 4.63E-05 | 0.000585 | 1.669938 |
| RTL6 | 1.01533 | 13.28519 | 4.437884 | 4.64E-05 | 0.000586 | 1.669176 |
| IGLV5-45 | -2.36237 | 12.25762 | -4.43533 | 4.68E-05 | 0.000591 | 1.660927 |
| RPL7AP66 | 1.7386 | 13.3564 | 4.434384 | 4.69E-05 | 0.000592 | 1.657869 |
| RPL29P33 | 1.668627 | 13.07755 | 4.433201 | 4.71E-05 | 0.000594 | 1.654048 |
| OLAH | -4.37657 | 10.69423 | -4.43164 | 4.73E-05 | 0.000597 | 1.649002 |
| SHOX | -1.12667 | 13.32859 | -4.4286 | 4.78E-05 | 0.000602 | 1.639181 |
| AC000041.1 | -1.01543 | 13.27863 | -4.42641 | 4.82E-05 | 0.000606 | 1.632134 |
| ZBTB10 | 1.24328 | 13.31325 | 4.426227 | 4.82E-05 | 0.000606 | 1.631532 |
| JSRP1 | -1.52944 | 13.0617 | -4.42606 | 4.82E-05 | 0.000606 | 1.631002 |
| KDF1 | 1.872736 | 12.97855 | 4.425796 | 4.83E-05 | 0.000606 | 1.630139 |
| PCOLCE2 | -2.96124 | 11.64155 | -4.4258 | 4.83E-05 | 0.000606 | 1.630138 |
| SIRPD | -1.02781 | 13.32639 | -4.42494 | 4.84E-05 | 0.000607 | 1.627369 |
| OASL | -1.8062 | 13.19435 | -4.42457 | 4.85E-05 | 0.000608 | 1.626175 |
| PTGFR | -2.48642 | 12.4148 | -4.42282 | 4.88E-05 | 0.000611 | 1.62055 |
| CYP4F22 | 1.803847 | 12.40912 | 4.42253 | 4.88E-05 | 0.000611 | 1.619601 |
| TRAV18 | 2.207135 | 12.77765 | 4.422373 | 4.89E-05 | 0.000611 | 1.619092 |
| IGHV3-72 | -2.18973 | 12.60168 | -4.4212 | 4.90E-05 | 0.000614 | 1.615306 |
| ZMAT4 | 2.411024 | 12.67118 | 4.420224 | 4.92E-05 | 0.000615 | 1.612159 |
| ITM2A | 1.277309 | 13.24255 | 4.41949 | 4.93E-05 | 0.000616 | 1.609793 |
| SERPINE1 | -1.57757 | 13.07596 | -4.41734 | 4.97E-05 | 0.00062 | 1.602852 |
| TRBJ1-4 | 1.593014 | 12.92825 | 4.416106 | 4.99E-05 | 0.000623 | 1.598881 |
| CA15P1 | -1.32935 | 13.26193 | -4.41575 | 5.00E-05 | 0.000623 | 1.597731 |
| PRODH | 1.135682 | 13.18471 | 4.415023 | 5.01E-05 | 0.000624 | 1.59539 |
| STAB2 | -1.74691 | 12.63541 | -4.41416 | 5.02E-05 | 0.000626 | 1.592603 |
| CDH12P2 | 1.013594 | 13.24278 | 4.414108 | 5.02E-05 | 0.000626 | 1.59244 |
| LYPD4 | -1.63039 | 13.16096 | -4.41249 | 5.05E-05 | 0.000628 | 1.587226 |
| RPL21P120 | 1.682303 | 13.0923 | 4.41237 | 5.05E-05 | 0.000628 | 1.586838 |
| MARCHF10 | 1.97956 | 13.0997 | 4.410111 | 5.09E-05 | 0.000632 | 1.579557 |
| IGHV1-58 | -2.67893 | 12.18791 | -4.40973 | 5.10E-05 | 0.000632 | 1.578332 |
| P2RX1 | -1.24299 | 13.10002 | -4.40964 | 5.10E-05 | 0.000632 | 1.578025 |
| AC005682.1 | 1.274334 | 13.314 | 4.408199 | 5.12E-05 | 0.000635 | 1.573396 |
| ASAH2B | 1.01777 | 13.35431 | 4.407201 | 5.14E-05 | 0.000637 | 1.570179 |
| PIMREG | -1.33417 | 13.08953 | -4.40651 | 5.15E-05 | 0.000638 | 1.567941 |
| TIMP1 | -1.03208 | 13.21526 | -4.40627 | 5.16E-05 | 0.000638 | 1.567185 |
| TIMP4 | -3.89814 | 11.36537 | -4.4047 | 5.19E-05 | 0.00064 | 1.562139 |
| AC234301.3 | -2.88416 | 12.49434 | -4.40462 | 5.19E-05 | 0.00064 | 1.561855 |
| PLEKHM3 | -1.02896 | 13.30992 | -4.40438 | 5.19E-05 | 0.00064 | 1.561088 |
| SLAMF8 | 1.097997 | 13.16279 | 4.404103 | 5.20E-05 | 0.00064 | 1.560204 |
| RBP5 | -1.29159 | 13.20749 | -4.40395 | 5.20E-05 | 0.00064 | 1.559694 |
| AC108463.1 | -1.26284 | 13.33008 | -4.40292 | 5.22E-05 | 0.000642 | 1.556393 |
| HLX | -1.04684 | 13.2262 | -4.40246 | 5.22E-05 | 0.000643 | 1.554908 |
| TRAJ44 | 1.78778 | 13.06452 | 4.402168 | 5.23E-05 | 0.000643 | 1.55397 |
| PATJ | 1.338832 | 13.22088 | 4.401484 | 5.24E-05 | 0.000644 | 1.55177 |
| SEPTIN7P8 | -1.59722 | 13.16644 | -4.4009 | 5.25E-05 | 0.000645 | 1.549875 |
| IGHV3OR16-13 | -3.3585 | 10.51289 | -4.40002 | 5.27E-05 | 0.000646 | 1.547047 |
| ZNF286A | 1.158253 | 13.32081 | 4.398968 | 5.29E-05 | 0.000648 | 1.543672 |
| N4BP3 | 1.173693 | 13.29601 | 4.397927 | 5.30E-05 | 0.00065 | 1.54032 |
| TRAJ47 | 2.070435 | 12.84119 | 4.39792 | 5.31E-05 | 0.00065 | 1.540298 |
| CA11 | 1.150289 | 13.28799 | 4.397625 | 5.31E-05 | 0.00065 | 1.539348 |
| ST20-MTHFS | -1.34993 | 13.11435 | -4.39669 | 5.33E-05 | 0.000652 | 1.536333 |
| HGF | -1.16924 | 13.28077 | -4.39664 | 5.33E-05 | 0.000652 | 1.536192 |
| IGLV7-43 | -1.8348 | 12.92997 | -4.39577 | 5.34E-05 | 0.000653 | 1.533389 |
| AC104837.2 | -2.8393 | 12.68697 | -4.39496 | 5.36E-05 | 0.000654 | 1.530769 |
| P2RY14 | 1.719727 | 12.95524 | 4.394936 | 5.36E-05 | 0.000654 | 1.530696 |
| RPL17P34 | 1.428845 | 13.607 | 4.394651 | 5.36E-05 | 0.000654 | 1.529778 |
| ATP2C2 | -2.22476 | 12.45283 | -4.3945 | 5.37E-05 | 0.000654 | 1.529308 |
| SLC39A10 | 1.042692 | 13.32301 | 4.392916 | 5.40E-05 | 0.000658 | 1.524196 |
| ZNF204P | 1.753106 | 13.07895 | 4.392449 | 5.40E-05 | 0.000658 | 1.522696 |
| GPA33 | 1.54627 | 13.0616 | 4.388173 | 5.48E-05 | 0.000667 | 1.508945 |
| PRKCQ | 1.171411 | 13.24988 | 4.387477 | 5.49E-05 | 0.000668 | 1.506708 |
| KLF12 | 1.472631 | 13.1736 | 4.386646 | 5.51E-05 | 0.000669 | 1.504035 |
| AC112777.1 | -3.02767 | 12.06358 | -4.38509 | 5.54E-05 | 0.000672 | 1.499028 |
| TRAV10 | 1.781104 | 12.97383 | 4.384313 | 5.55E-05 | 0.000673 | 1.496537 |
| TRBV19 | 1.809825 | 12.88277 | 4.382842 | 5.58E-05 | 0.000675 | 1.491811 |
| TRAV38-1 | 1.944966 | 12.80381 | 4.380635 | 5.62E-05 | 0.000679 | 1.48472 |
| RPL7AP6 | 1.885714 | 13.04182 | 4.379974 | 5.64E-05 | 0.00068 | 1.482597 |
| NMNAT2 | -1.68149 | 13.0894 | -4.37996 | 5.64E-05 | 0.00068 | 1.482545 |
| AC018638.8 | -1.42272 | 13.17979 | -4.37961 | 5.64E-05 | 0.000681 | 1.48143 |
| TRAJ40 | 1.980374 | 12.93505 | 4.379361 | 5.65E-05 | 0.000681 | 1.480626 |
| LMOD1 | 1.965449 | 11.88907 | 4.379071 | 5.65E-05 | 0.000681 | 1.479694 |
| DGKI | -2.31304 | 12.27094 | -4.37772 | 5.68E-05 | 0.000684 | 1.475344 |
| MRGPRX2 | 1.576776 | 13.3272 | 4.377103 | 5.69E-05 | 0.000685 | 1.473374 |
| MAGEE1 | 1.419849 | 13.17223 | 4.375681 | 5.72E-05 | 0.000688 | 1.46881 |
| TRPV5 | -1.86069 | 13.09513 | -4.37447 | 5.74E-05 | 0.00069 | 1.464932 |
| AC091959.3 | -2.19209 | 12.83439 | -4.37294 | 5.77E-05 | 0.000693 | 1.460025 |
| PHGDH | -1.4531 | 13.18879 | -4.37237 | 5.78E-05 | 0.000693 | 1.458194 |
| USP53 | 1.116313 | 13.36722 | 4.371931 | 5.79E-05 | 0.000694 | 1.456769 |
| IGKV1-12 | -2.84453 | 11.33759 | -4.37103 | 5.81E-05 | 0.000696 | 1.453884 |
| COL6A1 | 1.709111 | 12.93104 | 4.369588 | 5.84E-05 | 0.000699 | 1.449251 |
| AC090114.3 | -1.29317 | 13.15698 | -4.36828 | 5.86E-05 | 0.000701 | 1.445041 |
| CYP7A1 | -1.77575 | 13.45515 | -4.36703 | 5.89E-05 | 0.000703 | 1.441038 |
| RFPL4AL1 | 1.974519 | 12.94522 | 4.366342 | 5.90E-05 | 0.000705 | 1.438838 |
| CCR3 | 2.614451 | 12.36571 | 4.365835 | 5.91E-05 | 0.000705 | 1.43721 |
| AIM2 | -1.42837 | 13.33058 | -4.36492 | 5.93E-05 | 0.000707 | 1.434262 |
| SELENOM | 1.147444 | 13.19884 | 4.363536 | 5.96E-05 | 0.00071 | 1.429838 |
| KCNK5 | -1.45001 | 12.65679 | -4.36312 | 5.96E-05 | 0.000711 | 1.428495 |
| IGHV3-35 | -2.34585 | 12.6087 | -4.36263 | 5.97E-05 | 0.000712 | 1.426948 |
| RRN3P1 | 1.068334 | 13.19147 | 4.362113 | 5.98E-05 | 0.000713 | 1.425273 |
| BCL3 | -1.06533 | 13.28667 | -4.36197 | 5.99E-05 | 0.000713 | 1.42482 |
| LTBP1 | -1.49847 | 12.91206 | -4.36159 | 5.99E-05 | 0.000713 | 1.423604 |
| TRAJ45 | 2.305848 | 12.73507 | 4.359078 | 6.05E-05 | 0.000717 | 1.415544 |
| ZNF781 | 1.193559 | 13.32189 | 4.359065 | 6.05E-05 | 0.000717 | 1.415504 |
| DOCK4 | -1.1803 | 13.22405 | -4.35863 | 6.05E-05 | 0.000718 | 1.414097 |
| AQP9 | -1.0634 | 13.38458 | -4.35666 | 6.09E-05 | 0.000722 | 1.407799 |
| GAS2L1 | -1.58424 | 13.01522 | -4.35382 | 6.15E-05 | 0.000728 | 1.398699 |
| SLC37A3 | -1.13397 | 13.24593 | -4.35331 | 6.16E-05 | 0.000729 | 1.397069 |
| ALPK1 | -1.06152 | 13.28114 | -4.35313 | 6.17E-05 | 0.000729 | 1.396496 |
| AC244226.1 | -3.75107 | 9.665013 | -4.35232 | 6.18E-05 | 0.000731 | 1.39389 |
| IGHV7-4-1 | -3.7511 | 9.664885 | -4.35223 | 6.19E-05 | 0.000731 | 1.393605 |
| TSPAN15 | 1.59679 | 13.14363 | 4.35079 | 6.22E-05 | 0.000733 | 1.388991 |
| FABP2 | -2.01264 | 12.88575 | -4.35025 | 6.23E-05 | 0.000734 | 1.387275 |
| ZNF831 | 1.462229 | 13.16338 | 4.34842 | 6.27E-05 | 0.000738 | 1.381403 |
| LMO7 | 1.189105 | 13.26978 | 4.348196 | 6.27E-05 | 0.000738 | 1.380686 |
| TMEM132C | 2.172257 | 12.84934 | 4.345666 | 6.32E-05 | 0.000743 | 1.372587 |
| KRT8P43 | -1.9921 | 13.07248 | -4.3456 | 6.33E-05 | 0.000743 | 1.372385 |
| ZNF239 | 1.545966 | 13.18991 | 4.344387 | 6.35E-05 | 0.000745 | 1.368493 |
| LRIG1 | 1.039913 | 13.18538 | 4.344265 | 6.35E-05 | 0.000745 | 1.368105 |
| PLAAT5 | 2.204798 | 12.55535 | 4.343352 | 6.37E-05 | 0.000747 | 1.365183 |
| METTL24 | 1.508355 | 13.67117 | 4.343288 | 6.37E-05 | 0.000747 | 1.364977 |
| IGLV1-50 | -2.57439 | 12.31413 | -4.34207 | 6.40E-05 | 0.000749 | 1.361074 |
| CDK2AP2P2 | -2.26704 | 12.93903 | -4.34205 | 6.40E-05 | 0.000749 | 1.361029 |
| FKBP9P1 | -1.07048 | 13.23068 | -4.34155 | 6.41E-05 | 0.00075 | 1.359423 |
| CD6 | 1.366864 | 13.08574 | 4.341402 | 6.41E-05 | 0.000751 | 1.358943 |
| SLC16A14 | -1.76148 | 12.94794 | -4.34041 | 6.44E-05 | 0.000753 | 1.355771 |
| TRBV18 | 1.590224 | 12.91037 | 4.339854 | 6.45E-05 | 0.000754 | 1.353991 |
| SYNC | -2.59365 | 12.48534 | -4.3377 | 6.49E-05 | 0.000758 | 1.347095 |
| AP004607.7 | 1.620344 | 13.48642 | 4.337243 | 6.50E-05 | 0.000759 | 1.34564 |
| HSP90B1 | -1.21234 | 13.29697 | -4.3372 | 6.51E-05 | 0.000759 | 1.345501 |
| CHST8 | -2.21307 | 12.77008 | -4.33631 | 6.53E-05 | 0.00076 | 1.342666 |
| TRBV10-2 | 1.895604 | 12.82285 | 4.335811 | 6.54E-05 | 0.000761 | 1.34106 |
| CASS4 | 1.199711 | 13.01369 | 4.335589 | 6.54E-05 | 0.000762 | 1.340352 |
| ZNF229 | 1.328456 | 13.22662 | 4.335256 | 6.55E-05 | 0.000762 | 1.339288 |
| VMO1 | 2.0422 | 11.9172 | 4.334662 | 6.56E-05 | 0.000764 | 1.337387 |
| VIPR2 | 1.250106 | 13.2503 | 4.330313 | 6.66E-05 | 0.000773 | 1.323488 |
| SHF | 1.01943 | 13.30728 | 4.330053 | 6.66E-05 | 0.000773 | 1.322657 |
| GP9 | -1.89834 | 12.73676 | -4.32965 | 6.67E-05 | 0.000773 | 1.321382 |
| NLRP9P1 | -1.19735 | 13.30165 | -4.32934 | 6.68E-05 | 0.000773 | 1.320373 |
| REM2 | -1.26618 | 13.26256 | -4.32929 | 6.68E-05 | 0.000773 | 1.320211 |
| ARL4C | 1.301144 | 13.21766 | 4.327709 | 6.72E-05 | 0.000777 | 1.315168 |
| AC114781.1 | -1.86571 | 12.94652 | -4.32541 | 6.77E-05 | 0.000782 | 1.307835 |
| NPTX2 | 1.914252 | 12.72634 | 4.324203 | 6.80E-05 | 0.000785 | 1.30397 |
| TRAV2 | 1.686928 | 13.01226 | 4.323704 | 6.81E-05 | 0.000786 | 1.302377 |
| CFH | 1.565285 | 13.13748 | 4.323677 | 6.81E-05 | 0.000786 | 1.302289 |
| DQX1 | -1.64098 | 13.05793 | -4.32231 | 6.84E-05 | 0.000789 | 1.297915 |
| DUXAP7 | -1.71206 | 13.31271 | -4.32027 | 6.89E-05 | 0.000794 | 1.291412 |
| RPL18AP3 | 1.276903 | 13.11416 | 4.319344 | 6.91E-05 | 0.000795 | 1.288458 |
| NEXN | -1.68717 | 13.18087 | -4.31924 | 6.91E-05 | 0.000795 | 1.288129 |
| CPNE6 | -1.79663 | 12.75828 | -4.31732 | 6.95E-05 | 0.0008 | 1.281988 |
| RPSAP12 | 1.88966 | 13.0714 | 4.314494 | 7.02E-05 | 0.000806 | 1.272982 |
| C12orf42 | 1.432672 | 13.21694 | 4.313326 | 7.05E-05 | 0.000808 | 1.269258 |
| TRGV3 | 1.606055 | 13.03448 | 4.312823 | 7.06E-05 | 0.000809 | 1.267655 |
| CXCL3 | -1.58408 | 12.85396 | -4.31257 | 7.06E-05 | 0.00081 | 1.266845 |
| AC090686.1 | 1.467027 | 13.722 | 4.311633 | 7.09E-05 | 0.000812 | 1.263858 |
| AC073135.6 | -1.2916 | 13.2736 | -4.31135 | 7.09E-05 | 0.000812 | 1.262962 |
| FAM157A | -1.2916 | 13.2736 | -4.31135 | 7.09E-05 | 0.000812 | 1.262962 |
| CTNNAL1 | -1.04049 | 12.70531 | -4.31107 | 7.10E-05 | 0.000812 | 1.262047 |
| PLCG1 | 1.26007 | 13.20355 | 4.307574 | 7.18E-05 | 0.00082 | 1.250918 |
| PROK2 | -1.29012 | 13.32705 | -4.30658 | 7.21E-05 | 0.000823 | 1.247756 |
| IGKV1D-8 | -1.77444 | 13.07283 | -4.30401 | 7.27E-05 | 0.000829 | 1.239573 |
| AC006386.2 | 1.207102 | 13.56562 | 4.303059 | 7.29E-05 | 0.00083 | 1.23653 |
| AC012005.1 | 1.207102 | 13.56562 | 4.303059 | 7.29E-05 | 0.00083 | 1.23653 |
| IGLV5-37 | -2.86832 | 12.17726 | -4.30304 | 7.29E-05 | 0.00083 | 1.236477 |
| ZNF732 | 1.884906 | 13.12879 | 4.300895 | 7.35E-05 | 0.000835 | 1.229637 |
| NHLH1 | -1.1237 | 13.3046 | -4.30002 | 7.37E-05 | 0.000837 | 1.226857 |
| TRAV8-4 | 1.510522 | 13.06144 | 4.297772 | 7.42E-05 | 0.000841 | 1.219689 |
| UBBP4 | 1.506903 | 12.4312 | 4.296969 | 7.44E-05 | 0.000843 | 1.217134 |
| AATK | -1.33321 | 13.19942 | -4.29663 | 7.45E-05 | 0.000843 | 1.216048 |
| ALOX5AP | -1.17612 | 13.18305 | -4.29587 | 7.47E-05 | 0.000845 | 1.213647 |
| TRIB2 | 1.146788 | 13.26219 | 4.295782 | 7.47E-05 | 0.000845 | 1.213353 |
| TRAJ37 | 1.753789 | 13.09676 | 4.295771 | 7.47E-05 | 0.000845 | 1.213319 |
| AL117187.1 | -1.83831 | 13.16887 | -4.29531 | 7.48E-05 | 0.000845 | 1.211838 |
| TRBV6-5 | 1.682119 | 12.92046 | 4.294128 | 7.51E-05 | 0.000849 | 1.208089 |
| BICDL1 | 1.27557 | 13.10728 | 4.293833 | 7.52E-05 | 0.000849 | 1.207149 |
| EEF1A1P3 | 1.272095 | 13.39853 | 4.293749 | 7.52E-05 | 0.000849 | 1.206883 |
| PPM1J | 1.182509 | 13.27932 | 4.293227 | 7.54E-05 | 0.00085 | 1.205221 |
| RPS27P29 | 1.908292 | 13.13131 | 4.29321 | 7.54E-05 | 0.00085 | 1.205166 |
| PTGES3P2 | 1.536404 | 13.12128 | 4.292812 | 7.55E-05 | 0.00085 | 1.203902 |
| SLC25A10 | -1.15655 | 13.17253 | -4.29279 | 7.55E-05 | 0.00085 | 1.203815 |
| ZFYVE9 | 1.144397 | 13.35811 | 4.291994 | 7.57E-05 | 0.000852 | 1.201297 |
| CACNA1E | -1.63029 | 13.16245 | -4.29076 | 7.60E-05 | 0.000855 | 1.197381 |
| C3orf18 | 1.179952 | 13.24044 | 4.288735 | 7.65E-05 | 0.000859 | 1.190928 |
| TRBV5-1 | 1.620566 | 12.86683 | 4.288276 | 7.66E-05 | 0.00086 | 1.18947 |
| ABCD2 | 1.647336 | 13.1281 | 4.287564 | 7.68E-05 | 0.000862 | 1.187203 |
| ZNF607 | 1.161538 | 13.31141 | 4.287467 | 7.68E-05 | 0.000862 | 1.186898 |
| RHBG | -1.41744 | 13.31855 | -4.28694 | 7.70E-05 | 0.000862 | 1.185224 |
| MEIS3 | -1.71386 | 13.01667 | -4.28685 | 7.70E-05 | 0.000862 | 1.184922 |
| TRAV41 | 1.976305 | 12.8223 | 4.286809 | 7.70E-05 | 0.000862 | 1.184804 |
| BATF2 | -1.7764 | 13.17389 | -4.2868 | 7.70E-05 | 0.000862 | 1.184767 |
| RAET1E | -1.59462 | 13.17294 | -4.28621 | 7.72E-05 | 0.000864 | 1.182907 |
| VCL | -1.04653 | 13.1934 | -4.28587 | 7.72E-05 | 0.000864 | 1.181806 |
| SLC22A4 | -1.20152 | 13.11055 | -4.28508 | 7.74E-05 | 0.000866 | 1.179307 |
| CUX2 | 2.638563 | 12.51449 | 4.284873 | 7.75E-05 | 0.000866 | 1.178646 |
| FOSB | 1.235443 | 12.84955 | 4.284606 | 7.76E-05 | 0.000866 | 1.177798 |
| AC097065.1 | -2.05904 | 12.88453 | -4.28393 | 7.77E-05 | 0.000868 | 1.175645 |
| TRAJ3 | 2.17561 | 12.9337 | 4.281358 | 7.84E-05 | 0.000874 | 1.167474 |
| BX679664.1 | 1.631294 | 13.23655 | 4.280832 | 7.85E-05 | 0.000875 | 1.165801 |
| RPS29P14 | -1.8376 | 13.1052 | -4.28058 | 7.86E-05 | 0.000876 | 1.165005 |
| MMP2 | -2.40087 | 12.45912 | -4.28022 | 7.87E-05 | 0.000876 | 1.163847 |
| TSPAN9 | -1.41526 | 13.04017 | -4.27995 | 7.88E-05 | 0.000877 | 1.162993 |
| CCL25 | -2.41693 | 12.80372 | -4.2792 | 7.90E-05 | 0.000879 | 1.160621 |
| NLRP9 | -1.73581 | 13.39009 | -4.27897 | 7.90E-05 | 0.000879 | 1.159896 |
| RETN | -2.51753 | 11.78375 | -4.27894 | 7.90E-05 | 0.000879 | 1.159786 |
| GYPC | 1.91683 | 11.93616 | 4.27858 | 7.91E-05 | 0.00088 | 1.158644 |
| TRBV23-1 | 2.163492 | 12.55907 | 4.278039 | 7.93E-05 | 0.000881 | 1.156927 |
| MUC21 | -1.62922 | 12.91966 | -4.27785 | 7.93E-05 | 0.000881 | 1.15632 |
| RPL23AP65 | 1.627041 | 13.34775 | 4.277645 | 7.94E-05 | 0.000881 | 1.155676 |
| DPY19L2P3 | -1.11772 | 13.14414 | -4.27631 | 7.97E-05 | 0.000885 | 1.151423 |
| ME3 | 1.457465 | 12.99302 | 4.276048 | 7.98E-05 | 0.000885 | 1.1506 |
| BIN1 | 1.004109 | 13.2887 | 4.273776 | 8.04E-05 | 0.000892 | 1.143385 |
| AL360294.1 | -1.42243 | 12.91517 | -4.27367 | 8.04E-05 | 0.000892 | 1.143047 |
| LRG1 | -1.39446 | 13.16988 | -4.2736 | 8.05E-05 | 0.000892 | 1.142816 |
| STBD1 | -1.47854 | 12.79876 | -4.2718 | 8.09E-05 | 0.000895 | 1.137102 |
| CNKSR1 | -1.34983 | 13.18045 | -4.27171 | 8.10E-05 | 0.000895 | 1.136837 |
| IL7R | 1.799007 | 13.04042 | 4.269536 | 8.16E-05 | 0.0009 | 1.129924 |
| CYP1B1 | -1.25887 | 13.3017 | -4.26797 | 8.20E-05 | 0.000904 | 1.12496 |
| AD000671.2 | -1.28329 | 13.11426 | -4.26792 | 8.20E-05 | 0.000904 | 1.124794 |
| H2BC18 | -1.50831 | 13.26029 | -4.26781 | 8.20E-05 | 0.000904 | 1.124451 |
| MEIS2 | -1.77874 | 13.04604 | -4.267 | 8.23E-05 | 0.000906 | 1.121865 |
| ST14 | -1.10707 | 13.37501 | -4.2666 | 8.24E-05 | 0.000907 | 1.120605 |
| SLC29A2 | 1.07584 | 13.19263 | 4.265181 | 8.28E-05 | 0.00091 | 1.116102 |
| TPI1P2 | -1.70983 | 13.12543 | -4.26481 | 8.29E-05 | 0.000911 | 1.114913 |
| RELN | -1.99958 | 10.95549 | -4.26469 | 8.29E-05 | 0.000911 | 1.114549 |
| AC135068.2 | -2.78265 | 12.35382 | -4.26301 | 8.34E-05 | 0.000916 | 1.109206 |
| ABCB1 | 1.495252 | 13.21844 | 4.26212 | 8.36E-05 | 0.000917 | 1.106394 |
| RAD23BP1 | 1.746903 | 13.41991 | 4.259874 | 8.42E-05 | 0.000923 | 1.099271 |
| THNSL1 | 1.249039 | 13.27811 | 4.259689 | 8.43E-05 | 0.000923 | 1.098687 |
| TRBV2 | 1.598325 | 12.96856 | 4.259612 | 8.43E-05 | 0.000923 | 1.09844 |
| RPLP0P6 | 1.9418 | 12.96745 | 4.259381 | 8.44E-05 | 0.000923 | 1.09771 |
| IGHJ2 | -2.43262 | 12.57152 | -4.25685 | 8.51E-05 | 0.000929 | 1.089672 |
| TRIM51BP | 3.296111 | 11.66163 | 4.256415 | 8.52E-05 | 0.00093 | 1.088308 |
| TAFA1 | 1.819928 | 12.88662 | 4.255372 | 8.55E-05 | 0.000933 | 1.085001 |
| VDAC1P1 | 1.569365 | 13.49047 | 4.254112 | 8.59E-05 | 0.000937 | 1.081009 |
| AC008072.1 | -1.0403 | 13.28576 | -4.25378 | 8.60E-05 | 0.000937 | 1.079944 |
| EPAS1 | -1.37657 | 13.1254 | -4.25166 | 8.66E-05 | 0.000942 | 1.073226 |
| TRAV19 | 1.82282 | 12.87598 | 4.251257 | 8.67E-05 | 0.000942 | 1.071965 |
| JCAD | -1.22383 | 13.14927 | -4.25003 | 8.70E-05 | 0.000945 | 1.068066 |
| SAMD5 | -1.37231 | 13.08086 | -4.24951 | 8.72E-05 | 0.000946 | 1.066424 |
| ATP8A2 | 1.45124 | 13.22612 | 4.249242 | 8.73E-05 | 0.000947 | 1.065583 |
| PLIN4 | -1.76992 | 12.9846 | -4.24883 | 8.74E-05 | 0.000948 | 1.064289 |
| ADGRD1 | 1.059217 | 13.0311 | 4.247292 | 8.78E-05 | 0.000952 | 1.059407 |
| P2RX6 | 1.461129 | 13.09287 | 4.24723 | 8.78E-05 | 0.000952 | 1.059211 |
| HNRNPA1P4 | 1.682844 | 13.39033 | 4.245853 | 8.82E-05 | 0.000955 | 1.054852 |
| ARHGEF25 | 1.421508 | 13.22537 | 4.245781 | 8.83E-05 | 0.000955 | 1.054622 |
| HSF5 | 1.691119 | 13.15324 | 4.245324 | 8.84E-05 | 0.000956 | 1.053175 |
| LTA | 1.116352 | 13.26347 | 4.244353 | 8.87E-05 | 0.000958 | 1.050103 |
| MTND5P28 | -1.48379 | 13.35615 | -4.24327 | 8.90E-05 | 0.000961 | 1.046674 |
| SULT1B1 | -1.1823 | 13.26929 | -4.243 | 8.91E-05 | 0.000962 | 1.045812 |
| VGF | -1.31988 | 13.82567 | -4.23932 | 9.02E-05 | 0.000971 | 1.034177 |
| PPP2R2B | 1.381705 | 13.10989 | 4.238983 | 9.03E-05 | 0.000972 | 1.033113 |
| FBXL16 | 1.28013 | 13.12444 | 4.237733 | 9.07E-05 | 0.000975 | 1.029158 |
| RPL17P10 | -1.78472 | 13.18131 | -4.23541 | 9.14E-05 | 0.000981 | 1.021813 |
| LYNX1 | 1.707155 | 12.26275 | 4.235317 | 9.14E-05 | 0.000981 | 1.021519 |
| RPL7P23 | 1.431559 | 13.20426 | 4.234603 | 9.16E-05 | 0.000983 | 1.019258 |
| GOLGA8A | 1.196079 | 13.1099 | 4.233384 | 9.20E-05 | 0.000986 | 1.015406 |
| ITM2C | -1.37926 | 13.18121 | -4.23319 | 9.20E-05 | 0.000986 | 1.014778 |
| SLC16A8 | -1.93303 | 12.98049 | -4.23311 | 9.21E-05 | 0.000986 | 1.014554 |
| CACNA1A | -1.18567 | 13.34169 | -4.23094 | 9.27E-05 | 0.000991 | 1.007695 |
| CCSER1 | -1.27709 | 13.30245 | -4.23039 | 9.29E-05 | 0.000992 | 1.005927 |
| PTGIS | -1.38058 | 13.29275 | -4.22991 | 9.30E-05 | 0.000993 | 1.004417 |
| ACVR1C | 1.678169 | 13.01256 | 4.228218 | 9.36E-05 | 0.000998 | 0.999081 |
| CEBPD | -1.21599 | 13.16118 | -4.22566 | 9.44E-05 | 0.001006 | 0.991002 |
| KCTD15 | 1.05524 | 13.0601 | 4.22484 | 9.46E-05 | 0.001008 | 0.988411 |
| NAALAD2 | 1.043053 | 13.24306 | 4.224428 | 9.47E-05 | 0.001008 | 0.987111 |
| IGHV3-64 | -2.55449 | 12.43164 | -4.22423 | 9.48E-05 | 0.001008 | 0.986478 |
| MROH6 | -1.39623 | 12.89534 | -4.22396 | 9.49E-05 | 0.001009 | 0.985636 |
| SYN2 | -1.87931 | 12.94986 | -4.22287 | 9.52E-05 | 0.001012 | 0.982182 |
| PKDCC | 2.102223 | 11.3624 | 4.222011 | 9.55E-05 | 0.001014 | 0.979476 |
| ALPL | -1.93931 | 12.82248 | -4.22088 | 9.59E-05 | 0.001017 | 0.975904 |
| PDE3A | -1.41577 | 13.03811 | -4.21893 | 9.65E-05 | 0.001023 | 0.969743 |
| LGALSL | -1.06568 | 13.26977 | -4.21869 | 9.66E-05 | 0.001023 | 0.969001 |
| FXYD2 | 1.57745 | 13.07583 | 4.217477 | 9.70E-05 | 0.001026 | 0.965168 |
| F7 | 1.84892 | 13.32203 | 4.217398 | 9.70E-05 | 0.001026 | 0.964919 |
| AC090114.1 | 1.323254 | 13.43259 | 4.217114 | 9.71E-05 | 0.001026 | 0.964023 |
| VDAC2P3 | -1.67202 | 13.01433 | -4.2161 | 9.74E-05 | 0.001028 | 0.960835 |
| EEF1A1P13 | 1.498821 | 12.64055 | 4.215767 | 9.75E-05 | 0.001029 | 0.959772 |
| P2RY10 | 1.296684 | 13.21299 | 4.215359 | 9.76E-05 | 0.00103 | 0.958484 |
| TIGIT | 1.529235 | 13.0052 | 4.214891 | 9.78E-05 | 0.001031 | 0.957009 |
| CHKB-CPT1B | -1.02547 | 13.36681 | -4.21412 | 9.80E-05 | 0.001032 | 0.954568 |
| RPL7P51 | -1.93215 | 13.18017 | -4.2137 | 9.82E-05 | 0.001033 | 0.953243 |
| DLC1 | -1.1357 | 13.00083 | -4.21315 | 9.84E-05 | 0.001034 | 0.951516 |
| RTN4RL1 | 1.378082 | 13.08595 | 4.212133 | 9.87E-05 | 0.001037 | 0.94831 |
| SLC22A1 | -1.31125 | 13.23167 | -4.21186 | 9.88E-05 | 0.001037 | 0.947444 |
| LSMEM2 | -1.73625 | 13.1484 | -4.21148 | 9.89E-05 | 0.001038 | 0.946236 |
| CARD17 | -1.89231 | 13.15557 | -4.21147 | 9.89E-05 | 0.001038 | 0.946234 |
| XCL1 | 2.068319 | 12.70096 | 4.210147 | 9.93E-05 | 0.001042 | 0.942047 |
| CYP4F25P | -2.45929 | 12.26479 | -4.20999 | 9.94E-05 | 0.001042 | 0.941539 |
| CITED4 | -1.69764 | 12.57884 | -4.20966 | 9.95E-05 | 0.001043 | 0.940513 |
| NOX1 | 1.03088 | 13.35324 | 4.209537 | 9.95E-05 | 0.001043 | 0.940126 |
| SLC44A5 | 1.811124 | 12.95857 | 4.208957 | 9.97E-05 | 0.001044 | 0.938298 |
| AL049634.1 | -1.21553 | 13.30611 | -4.20889 | 9.98E-05 | 0.001044 | 0.938097 |
| DPEP1 | -2.58096 | 12.52992 | -4.20832 | 9.99E-05 | 0.001046 | 0.93628 |
| ZNF165 | 1.090704 | 13.16028 | 4.208074 | 0.0001 | 0.001046 | 0.935513 |
| TRAJ43 | 1.997163 | 12.87681 | 4.20807 | 0.0001 | 0.001046 | 0.935502 |
| CNIH2 | -1.03991 | 13.22477 | -4.20776 | 0.0001 | 0.001047 | 0.934531 |
| IGKJ5 | -1.60133 | 12.58193 | -4.20645 | 0.000101 | 0.00105 | 0.930388 |
| DPF1 | -1.07094 | 13.28918 | -4.20442 | 0.000101 | 0.001056 | 0.923987 |
| EFCAB2 | -1.23131 | 13.3416 | -4.2038 | 0.000101 | 0.001057 | 0.922056 |
| CYP24A1 | 2.045132 | 12.74889 | 4.203396 | 0.000102 | 0.001058 | 0.920772 |
| SPHK1 | -1.08706 | 13.13107 | -4.20153 | 0.000102 | 0.001063 | 0.9149 |
| AC112184.1 | 1.959017 | 13.04761 | 4.199836 | 0.000103 | 0.001068 | 0.90956 |
| MTND4P26 | -1.86216 | 13.13095 | -4.1998 | 0.000103 | 0.001068 | 0.909455 |
| CACNA1C | 1.357684 | 13.10831 | 4.199466 | 0.000103 | 0.001069 | 0.908394 |
| OBSCN-AS1 | 1.811506 | 12.79304 | 4.199033 | 0.000103 | 0.001069 | 0.907032 |
| CDH7 | -1.27938 | 13.25231 | -4.19822 | 0.000103 | 0.001071 | 0.90446 |
| TNFRSF21 | 1.929953 | 12.84721 | 4.195144 | 0.000104 | 0.00108 | 0.894789 |
| ADAMTS3 | -2.87841 | 11.48657 | -4.19511 | 0.000104 | 0.00108 | 0.894673 |
| IGHV3-36 | -1.87622 | 12.94909 | -4.19465 | 0.000105 | 0.001081 | 0.893234 |
| GRINA | -1.09924 | 13.11967 | -4.19446 | 0.000105 | 0.001081 | 0.892624 |
| LGR4 | 1.108408 | 13.05902 | 4.193931 | 0.000105 | 0.001083 | 0.890972 |
| MPO | -2.66959 | 11.36537 | -4.19234 | 0.000105 | 0.001088 | 0.885954 |
| TAS1R3 | -1.06703 | 13.33179 | -4.19061 | 0.000106 | 0.001092 | 0.880523 |
| DSC1 | 2.541643 | 12.58677 | 4.189716 | 0.000106 | 0.001095 | 0.877712 |
| SCUBE1 | -1.44716 | 13.16991 | -4.18862 | 0.000107 | 0.001098 | 0.874268 |
| FAM241B | 1.680976 | 12.9616 | 4.187303 | 0.000107 | 0.001102 | 0.870123 |
| ZMYND11 | 1.016078 | 13.36168 | 4.185991 | 0.000108 | 0.001106 | 0.865999 |
| CD247 | 1.393086 | 13.13621 | 4.184057 | 0.000108 | 0.001112 | 0.859919 |
| TRBV5-4 | 1.835615 | 12.8743 | 4.182778 | 0.000109 | 0.001117 | 0.8559 |
| H3C1 | -1.65328 | 13.27768 | -4.18212 | 0.000109 | 0.001118 | 0.85383 |
| IGHV3-63 | -2.21691 | 12.70621 | -4.18191 | 0.000109 | 0.001119 | 0.853164 |
| ARHGAP42 | -1.38309 | 13.06217 | -4.18079 | 0.000109 | 0.001122 | 0.849668 |
| SLC11A1 | -1.49459 | 13.02064 | -4.18073 | 0.000109 | 0.001122 | 0.84945 |
| IGHV1-69D | -3.23865 | 11.72449 | -4.18067 | 0.00011 | 0.001122 | 0.849277 |
| ZNF391 | 1.393302 | 13.19527 | 4.180443 | 0.00011 | 0.001122 | 0.848563 |
| ZNF404 | 1.262406 | 13.20184 | 4.179779 | 0.00011 | 0.001124 | 0.846477 |
| TLE2 | 1.495847 | 12.95025 | 4.17961 | 0.00011 | 0.001124 | 0.845944 |
| ANKRD30BL | -1.43026 | 13.18339 | -4.17814 | 0.00011 | 0.001129 | 0.841335 |
| GUCY2C | -1.20996 | 13.27946 | -4.17612 | 0.000111 | 0.001134 | 0.834985 |
| SLIT1 | -1.07393 | 13.39632 | -4.17611 | 0.000111 | 0.001134 | 0.834951 |
| H1-0 | -1.22926 | 12.81669 | -4.17439 | 0.000112 | 0.001139 | 0.829546 |
| SMPD3 | 1.340728 | 12.79831 | 4.174387 | 0.000112 | 0.001139 | 0.829542 |
| IGKV3-7 | -2.10626 | 12.5217 | -4.17331 | 0.000112 | 0.001142 | 0.826164 |
| PTK7 | 1.031689 | 13.26474 | 4.173003 | 0.000112 | 0.001143 | 0.825197 |
| AC103705.1 | 1.286527 | 13.19961 | 4.172884 | 0.000112 | 0.001143 | 0.824825 |
| CTBP2P8 | -1.76444 | 13.10824 | -4.1724 | 0.000113 | 0.001144 | 0.823319 |
| CILP2 | -1.753 | 12.43331 | -4.17224 | 0.000113 | 0.001144 | 0.822792 |
| PRR16 | -1.47777 | 13.18067 | -4.17203 | 0.000113 | 0.001144 | 0.822152 |
| C1orf167 | -1.00605 | 13.34346 | -4.17079 | 0.000113 | 0.001148 | 0.818258 |
| RPH3A | -2.20086 | 12.68872 | -4.16554 | 0.000115 | 0.001165 | 0.801789 |
| C15orf65 | -1.04659 | 13.3254 | -4.16249 | 0.000116 | 0.001175 | 0.79221 |
| AL136531.2 | -1.86885 | 13.14832 | -4.16128 | 0.000117 | 0.001179 | 0.788421 |
| PDE2A | -1.40181 | 13.1621 | -4.16071 | 0.000117 | 0.00118 | 0.786646 |
| SMIM10 | -1.46666 | 12.80902 | -4.1603 | 0.000117 | 0.001182 | 0.785367 |
| RLN2 | 1.883187 | 13.01716 | 4.158296 | 0.000118 | 0.001189 | 0.779075 |
| AC114797.1 | 3.827979 | 11.15044 | 4.157961 | 0.000118 | 0.00119 | 0.778022 |
| PTPN13 | 1.442697 | 13.07372 | 4.156791 | 0.000118 | 0.001193 | 0.774359 |
| FAM47E-STBD1 | -1.2292 | 12.87253 | -4.15652 | 0.000119 | 0.001194 | 0.773521 |
| SLC2A14 | -2.22072 | 12.23821 | -4.1547 | 0.000119 | 0.0012 | 0.767796 |
| SCN2B | -1.07053 | 13.39562 | -4.1511 | 0.000121 | 0.001212 | 0.756536 |
| PNMA3 | 1.446545 | 12.98595 | 4.150134 | 0.000121 | 0.001215 | 0.753511 |
| ZNF135 | 1.117958 | 13.29136 | 4.149601 | 0.000121 | 0.001217 | 0.751842 |
| ADAMTS10 | 1.58204 | 12.90966 | 4.149229 | 0.000121 | 0.001218 | 0.750678 |
| CXCL6 | 1.494494 | 13.02587 | 4.148473 | 0.000122 | 0.00122 | 0.748311 |
| ACOX2 | -1.67379 | 12.91445 | -4.14603 | 0.000123 | 0.001229 | 0.740666 |
| TRBV21-1 | 2.011051 | 12.5643 | 4.144251 | 0.000123 | 0.001235 | 0.735102 |
| DBH | 1.882498 | 12.74948 | 4.143611 | 0.000124 | 0.001238 | 0.7331 |
| C5orf47 | -1.99305 | 13.01372 | -4.14324 | 0.000124 | 0.001238 | 0.731952 |
| TRAV34 | 2.278238 | 12.63386 | 4.142678 | 0.000124 | 0.001239 | 0.73018 |
| AC239798.1 | -1.58531 | 12.90462 | -4.14257 | 0.000124 | 0.001239 | 0.729838 |
| SPIN3 | 1.078532 | 13.25533 | 4.141368 | 0.000125 | 0.001244 | 0.726083 |
| UBBP1 | 1.690219 | 12.72534 | 4.137738 | 0.000126 | 0.001257 | 0.714735 |
| RPL7P6 | 1.710545 | 13.39937 | 4.137293 | 0.000126 | 0.001258 | 0.713342 |
| KCNJ15 | -1.21092 | 13.30027 | -4.13636 | 0.000127 | 0.001261 | 0.710426 |
| OLIG2 | 2.286718 | 12.16101 | 4.136254 | 0.000127 | 0.001261 | 0.710096 |
| ZSCAN31 | 1.049788 | 13.33463 | 4.134691 | 0.000127 | 0.001266 | 0.705211 |
| AC022018.1 | 1.364502 | 13.80485 | 4.133232 | 0.000128 | 0.001271 | 0.700653 |
| ST6GALNAC1 | 1.806359 | 12.8448 | 4.13109 | 0.000129 | 0.001279 | 0.693964 |
| BCAS2P2 | 1.798695 | 13.09721 | 4.129476 | 0.00013 | 0.001285 | 0.688924 |
| CA15P2 | -1.65931 | 13.22518 | -4.12805 | 0.00013 | 0.001291 | 0.68447 |
| RPL10AP6 | 1.718638 | 13.06919 | 4.128015 | 0.00013 | 0.001291 | 0.684362 |
| ALS2CL | 1.301798 | 12.90253 | 4.127615 | 0.00013 | 0.001292 | 0.683112 |
| NAP1L4P1 | -1.29519 | 13.24852 | -4.1274 | 0.000131 | 0.001292 | 0.682454 |
| NOXRED1 | -1.31006 | 13.3571 | -4.12688 | 0.000131 | 0.001293 | 0.680804 |
| AC114801.3 | 1.03838 | 13.11025 | 4.124923 | 0.000132 | 0.0013 | 0.674709 |
| EEF1A1P11 | 1.660739 | 12.45918 | 4.123586 | 0.000132 | 0.001305 | 0.670537 |
| IRAK3 | -1.42076 | 13.12184 | -4.12306 | 0.000132 | 0.001307 | 0.668882 |
| MLNR | -2.52241 | 11.7723 | -4.12258 | 0.000133 | 0.001308 | 0.667403 |
| TRAV23DV6 | 1.742203 | 12.84598 | 4.121693 | 0.000133 | 0.001312 | 0.664633 |
| RPL5P4 | 1.533391 | 13.55509 | 4.121588 | 0.000133 | 0.001312 | 0.664304 |
| GPR39 | 1.422556 | 13.74743 | 4.121438 | 0.000133 | 0.001312 | 0.663837 |
| NATD1 | -1.12607 | 12.74453 | -4.1187 | 0.000134 | 0.001323 | 0.655295 |
| PLEKHB1 | 1.448033 | 13.10308 | 4.118083 | 0.000135 | 0.001325 | 0.653372 |
| PCYT1B | -1.43323 | 12.95277 | -4.11733 | 0.000135 | 0.001327 | 0.651016 |
| IGKV6D-21 | -2.33528 | 11.92193 | -4.11593 | 0.000136 | 0.001333 | 0.646659 |
| CNGB1 | -1.14936 | 13.26984 | -4.11585 | 0.000136 | 0.001333 | 0.646408 |
| CD248 | 1.93996 | 12.93674 | 4.115691 | 0.000136 | 0.001333 | 0.645915 |
| LYG2 | -1.70927 | 13.06206 | -4.11525 | 0.000136 | 0.001334 | 0.644541 |
| AC051619.1 | -1.37888 | 13.16747 | -4.1139 | 0.000136 | 0.001339 | 0.640344 |
| EDAR | 1.999808 | 12.82221 | 4.113747 | 0.000136 | 0.001339 | 0.639857 |
| IGLJ1 | -2.32799 | 12.68093 | -4.11175 | 0.000137 | 0.001347 | 0.633625 |
| TRAV39 | 1.980006 | 12.8417 | 4.111634 | 0.000137 | 0.001347 | 0.633269 |
| KDELR3 | -1.08805 | 13.40186 | -4.11149 | 0.000138 | 0.001347 | 0.632818 |
| TOM1L1 | -1.03599 | 13.14245 | -4.11076 | 0.000138 | 0.00135 | 0.630546 |
| PRSS3P3 | 1.326683 | 14.135 | 4.110434 | 0.000138 | 0.001351 | 0.629531 |
| PLSCR1 | -1.36421 | 13.28067 | -4.10723 | 0.000139 | 0.001364 | 0.619563 |
| CCDC102B | 1.019647 | 13.34448 | 4.106627 | 0.00014 | 0.001366 | 0.617676 |
| SERPING1 | -1.96017 | 13.04323 | -4.10647 | 0.00014 | 0.001366 | 0.617184 |
| AC099335.1 | 1.382497 | 13.80175 | 4.106362 | 0.00014 | 0.001366 | 0.61685 |
| CSNK1A1L | -1.51822 | 13.01503 | -4.10614 | 0.00014 | 0.001367 | 0.616162 |
| ROCK1P1 | 1.833421 | 12.78257 | 4.105345 | 0.00014 | 0.00137 | 0.613683 |
| CCDC140 | -2.05711 | 12.82669 | -4.10496 | 0.00014 | 0.001371 | 0.612471 |
| CEND1 | -1.89456 | 12.97326 | -4.10466 | 0.000141 | 0.001371 | 0.611548 |
| DOCK9 | 1.253214 | 13.24948 | 4.103791 | 0.000141 | 0.001373 | 0.608845 |
| GSG1L | -1.80115 | 12.99486 | -4.10345 | 0.000141 | 0.001374 | 0.607773 |
| CFAP206 | -1.61321 | 13.14602 | -4.10328 | 0.000141 | 0.001375 | 0.607266 |
| RPS4XP3 | 1.482988 | 13.75817 | 4.102236 | 0.000142 | 0.001378 | 0.604007 |
| DEFB109F | 1.571728 | 13.18028 | 4.098454 | 0.000144 | 0.001393 | 0.592239 |
| AC025181.1 | -1.8211 | 13.058 | -4.09837 | 0.000144 | 0.001393 | 0.591973 |
| AC091429.1 | 1.760106 | 13.10967 | 4.098156 | 0.000144 | 0.001394 | 0.591311 |
| IGHG2 | -1.86524 | 12.71974 | -4.09685 | 0.000144 | 0.001398 | 0.587264 |
| RPL21P1 | 1.306661 | 13.96374 | 4.096737 | 0.000144 | 0.001398 | 0.586898 |
| PDPN | -1.35402 | 13.31004 | -4.09539 | 0.000145 | 0.001404 | 0.582699 |
| HLA-DPA2 | 1.015263 | 14.50908 | 4.095089 | 0.000145 | 0.001405 | 0.581773 |
| SEPTIN4 | -1.47974 | 13.29751 | -4.09483 | 0.000145 | 0.001405 | 0.580964 |
| KLRA1P | 1.418317 | 13.03252 | 4.094726 | 0.000145 | 0.001405 | 0.580645 |
| IGHV3-69-1 | -1.8291 | 12.89973 | -4.09291 | 0.000146 | 0.001412 | 0.574997 |
| F12 | -1.31267 | 13.05945 | -4.09169 | 0.000147 | 0.001416 | 0.571213 |
| PCBP4 | 1.07542 | 13.20097 | 4.091576 | 0.000147 | 0.001416 | 0.570853 |
| TAAR1 | -2.00069 | 13.16567 | -4.0884 | 0.000148 | 0.00143 | 0.560993 |
| DNAH17 | -1.50012 | 13.19245 | -4.08818 | 0.000148 | 0.00143 | 0.560311 |
| TRAJ34 | 1.915242 | 12.8251 | 4.086732 | 0.000149 | 0.001435 | 0.555801 |
| TEX19 | -1.68209 | 13.26757 | -4.08663 | 0.000149 | 0.001435 | 0.555495 |
| AC134981.1 | -2.32496 | 12.2007 | -4.08645 | 0.000149 | 0.001435 | 0.554935 |
| IGHV1OR15-4 | -2.32496 | 12.2007 | -4.08645 | 0.000149 | 0.001435 | 0.554935 |
| TRAJ4 | 2.079817 | 12.81465 | 4.08623 | 0.000149 | 0.001436 | 0.554243 |
| CFAP97 | 1.040211 | 13.3301 | 4.086184 | 0.000149 | 0.001436 | 0.554099 |
| ARHGAP6 | -1.22694 | 13.15244 | -4.08594 | 0.00015 | 0.001436 | 0.553349 |
| ACSS3 | -1.28732 | 13.04389 | -4.08542 | 0.00015 | 0.001438 | 0.551723 |
| ACER1 | 1.39158 | 13.19869 | 4.084975 | 0.00015 | 0.001439 | 0.550344 |
| CALML6 | 1.422766 | 13.22879 | 4.084304 | 0.00015 | 0.001441 | 0.548262 |
| GZMA | 1.34491 | 13.0849 | 4.083263 | 0.000151 | 0.001445 | 0.54503 |
| ERICH5 | 1.433824 | 13.44568 | 4.081101 | 0.000152 | 0.001455 | 0.538318 |
| LRRFIP1P1 | -1.68795 | 12.74106 | -4.07989 | 0.000152 | 0.001459 | 0.534563 |
| PTPRVP | -1.07652 | 13.28733 | -4.07988 | 0.000152 | 0.001459 | 0.534518 |
| STX3 | -1.05649 | 13.2346 | -4.07939 | 0.000153 | 0.00146 | 0.532992 |
| TRAJ35 | 1.638596 | 13.35091 | 4.079067 | 0.000153 | 0.001461 | 0.532004 |
| RPL9P33 | -1.5801 | 13.23476 | -4.07833 | 0.000153 | 0.001465 | 0.529715 |
| NUGGC | -1.07326 | 13.35208 | -4.07804 | 0.000153 | 0.001465 | 0.528804 |
| DYNC1I1 | -1.21188 | 13.13947 | -4.07745 | 0.000154 | 0.001468 | 0.526987 |
| PRKCG | -1.44348 | 13.25785 | -4.07663 | 0.000154 | 0.001471 | 0.524429 |
| PRR15 | -1.92372 | 12.90146 | -4.07654 | 0.000154 | 0.001471 | 0.524154 |
| AC000089.1 | 1.484188 | 13.4947 | 4.076298 | 0.000154 | 0.001471 | 0.523413 |
| LPAL2 | 1.264742 | 13.17 | 4.076167 | 0.000154 | 0.001472 | 0.523007 |
| FIGN | -3.10416 | 10.11367 | -4.07589 | 0.000154 | 0.001473 | 0.522156 |
| SIT1 | 1.101916 | 13.21344 | 4.075778 | 0.000155 | 0.001473 | 0.521802 |
| NAP1L2 | 1.500872 | 13.14214 | 4.075205 | 0.000155 | 0.001474 | 0.520023 |
| GPR42 | -3.02116 | 12.44688 | -4.0752 | 0.000155 | 0.001474 | 0.519993 |
| SCO2 | -1.3023 | 13.32169 | -4.07502 | 0.000155 | 0.001475 | 0.519461 |
| IFNL1 | 1.687255 | 13.14483 | 4.074573 | 0.000155 | 0.001476 | 0.518063 |
| GALNT18 | -1.77161 | 13.13062 | -4.07299 | 0.000156 | 0.001483 | 0.513144 |
| LIN28A | -1.11887 | 13.25559 | -4.0717 | 0.000157 | 0.001489 | 0.509145 |
| TRAV21 | 1.727924 | 12.91079 | 4.071386 | 0.000157 | 0.00149 | 0.50818 |
| RERG | -1.64933 | 13.07078 | -4.06845 | 0.000158 | 0.001502 | 0.499094 |
| RPL21P119 | 3.305387 | 11.82398 | 4.06844 | 0.000158 | 0.001502 | 0.499048 |
| NOSTRIN | -1.11593 | 13.08193 | -4.06768 | 0.000159 | 0.001505 | 0.496705 |
| TRBJ2-2 | 1.504375 | 13.07632 | 4.066646 | 0.000159 | 0.001508 | 0.493491 |
| AC004692.1 | 1.026554 | 14.72484 | 4.065795 | 0.00016 | 0.001511 | 0.490853 |
| ZDHHC11B | 1.613588 | 12.78115 | 4.065236 | 0.00016 | 0.001512 | 0.489121 |
| SEC1P | 1.271733 | 13.27326 | 4.064891 | 0.00016 | 0.001513 | 0.488052 |
| FUT7 | -1.00906 | 13.32192 | -4.06451 | 0.00016 | 0.001514 | 0.48686 |
| ANKRD9 | -1.64497 | 12.08358 | -4.06433 | 0.00016 | 0.001515 | 0.486324 |
| AC233755.2 | -2.68104 | 11.99173 | -4.06401 | 0.000161 | 0.001515 | 0.485316 |
| MAN2A2 | -1.07541 | 13.22749 | -4.06311 | 0.000161 | 0.001519 | 0.482545 |
| ODCP | -1.17737 | 13.2571 | -4.06211 | 0.000162 | 0.001523 | 0.47945 |
| AP005431.2 | 1.497123 | 13.90574 | 4.062054 | 0.000162 | 0.001523 | 0.479267 |
| TMEM171 | 2.021994 | 12.94384 | 4.061635 | 0.000162 | 0.001525 | 0.47797 |
| ALOX12 | -1.25026 | 13.13289 | -4.06026 | 0.000163 | 0.001531 | 0.473725 |
| AMOT | 1.221875 | 13.18765 | 4.05989 | 0.000163 | 0.001533 | 0.472567 |
| FCRL5 | -1.16063 | 13.37388 | -4.05914 | 0.000163 | 0.001535 | 0.470254 |
| AC104451.1 | -2.32551 | 11.87502 | -4.05642 | 0.000165 | 0.001548 | 0.461826 |
| AC099489.2 | -1.64651 | 13.20301 | -4.05353 | 0.000166 | 0.00156 | 0.452874 |
| MAP2 | -1.54944 | 13.05969 | -4.05119 | 0.000167 | 0.00157 | 0.445653 |
| SIGLEC16 | -1.3563 | 13.19449 | -4.04893 | 0.000169 | 0.00158 | 0.438676 |
| CDH6 | -1.11066 | 12.7745 | -4.04888 | 0.000169 | 0.00158 | 0.438513 |
| PPL | -1.3648 | 13.29873 | -4.04852 | 0.000169 | 0.001582 | 0.437384 |
| TRAV9-2 | 1.475022 | 13.01661 | 4.046981 | 0.00017 | 0.001588 | 0.43264 |
| KIF3A | 1.017337 | 13.3646 | 4.046893 | 0.00017 | 0.001588 | 0.432367 |
| TRAV12-2 | 1.590017 | 13.00992 | 4.046717 | 0.00017 | 0.001589 | 0.431822 |
| STOM | -1.1192 | 12.8297 | -4.04468 | 0.000171 | 0.001598 | 0.42553 |
| ESM1 | 1.282186 | 13.23481 | 4.044433 | 0.000171 | 0.001599 | 0.424766 |
| PPP1R2P1 | -1.23852 | 13.36819 | -4.04318 | 0.000172 | 0.001604 | 0.420886 |
| TPST1 | -2.16888 | 12.63195 | -4.04147 | 0.000173 | 0.001612 | 0.415627 |
| REG4 | 2.674612 | 12.40896 | 4.041091 | 0.000173 | 0.001613 | 0.414444 |
| COPZ2 | 1.153846 | 13.19803 | 4.040733 | 0.000173 | 0.001614 | 0.413337 |
| IL20RA | 1.253857 | 13.62472 | 4.040158 | 0.000174 | 0.001616 | 0.411561 |
| TRGVA | 1.631429 | 13.06027 | 4.039998 | 0.000174 | 0.001616 | 0.411066 |
| BCL9L | 1.078893 | 13.21455 | 4.039937 | 0.000174 | 0.001616 | 0.410879 |
| SLC12A3 | 1.743169 | 13.08685 | 4.038856 | 0.000174 | 0.001622 | 0.40754 |
| BATF3 | 1.119612 | 12.99782 | 4.038595 | 0.000174 | 0.001622 | 0.406734 |
| ITK | 1.371442 | 13.14501 | 4.038209 | 0.000175 | 0.001624 | 0.405544 |
| MTRNR2L1 | 1.037734 | 13.08088 | 4.037956 | 0.000175 | 0.001625 | 0.404764 |
| AC091153.1 | 1.459552 | 13.26081 | 4.036015 | 0.000176 | 0.001635 | 0.398773 |
| NLGN4Y | 1.921229 | 13.04996 | 4.035268 | 0.000176 | 0.001638 | 0.396467 |
| H2AC12 | -1.44678 | 13.59448 | -4.03199 | 0.000178 | 0.001652 | 0.386364 |
| ACSL6 | 1.215427 | 12.44696 | 4.031951 | 0.000178 | 0.001652 | 0.386233 |
| RUNX1T1 | -1.42479 | 12.87712 | -4.03165 | 0.000178 | 0.001652 | 0.385317 |
| GRIK5 | -1.11109 | 13.1515 | -4.03133 | 0.000179 | 0.001654 | 0.384327 |
| RPL6P4 | 1.147368 | 14.2651 | 4.031233 | 0.000179 | 0.001654 | 0.384017 |
| LUM | 1.659283 | 13.05672 | 4.030858 | 0.000179 | 0.001655 | 0.382861 |
| NECTIN3 | 1.750588 | 13.07831 | 4.029207 | 0.00018 | 0.001662 | 0.377772 |
| SALL2 | 1.378314 | 13.15825 | 4.025784 | 0.000182 | 0.001677 | 0.367217 |
| KIAA0895 | 1.126648 | 13.30929 | 4.025666 | 0.000182 | 0.001677 | 0.366855 |
| CCNG1P1 | -1.95397 | 13.00769 | -4.02559 | 0.000182 | 0.001677 | 0.366629 |
| ADIPOQ | -1.25766 | 13.29857 | -4.02297 | 0.000184 | 0.00169 | 0.358559 |
| TMEM25 | 1.005288 | 13.29222 | 4.022717 | 0.000184 | 0.001691 | 0.357766 |
| ADGRG3 | -1.35681 | 13.11998 | -4.02198 | 0.000184 | 0.001695 | 0.355489 |
| CHRM3 | 1.247438 | 13.13158 | 4.021881 | 0.000184 | 0.001695 | 0.355192 |
| CDKN1C | 1.746518 | 11.8591 | 4.02086 | 0.000185 | 0.001699 | 0.352047 |
| ALOX15 | 3.532431 | 10.89127 | 4.020577 | 0.000185 | 0.0017 | 0.351174 |
| PLAAT2 | -1.98948 | 12.91826 | -4.02024 | 0.000185 | 0.001701 | 0.350128 |
| IGKV3D-11 | -2.55484 | 12.60204 | -4.01949 | 0.000186 | 0.001704 | 0.347818 |
| OSBPL6 | -1.21302 | 12.91305 | -4.01937 | 0.000186 | 0.001704 | 0.347459 |
| TRAV26-2 | 1.950318 | 12.83702 | 4.019147 | 0.000186 | 0.001705 | 0.346772 |
| CREB3L1 | -1.83041 | 12.1109 | -4.01806 | 0.000187 | 0.001711 | 0.343417 |
| AC098935.2 | 1.53804 | 13.05528 | 4.017779 | 0.000187 | 0.001712 | 0.342559 |
| PIWIL2 | 1.253353 | 13.14023 | 4.01772 | 0.000187 | 0.001712 | 0.342378 |
| EEF1A1P30 | 1.53613 | 13.10804 | 4.017258 | 0.000187 | 0.001713 | 0.340954 |
| PIFO | 1.399217 | 13.33047 | 4.016135 | 0.000188 | 0.001718 | 0.337496 |
| MTRNR2L2 | 1.146442 | 14.01248 | 4.016119 | 0.000188 | 0.001718 | 0.337448 |
| ZNF681 | 1.313557 | 13.25516 | 4.015003 | 0.000188 | 0.001723 | 0.334012 |
| MAB21L3 | -1.11623 | 13.307 | -4.01368 | 0.000189 | 0.001729 | 0.329954 |
| AC098590.1 | 1.183278 | 14.1035 | 4.012608 | 0.00019 | 0.001734 | 0.326643 |
| RBP4 | -2.79062 | 11.94192 | -4.01254 | 0.00019 | 0.001734 | 0.326434 |
| OR2B2 | -2.0937 | 12.99043 | -4.01235 | 0.00019 | 0.001734 | 0.32586 |
| AC234635.2 | 2.346977 | 12.54901 | 4.011893 | 0.00019 | 0.001736 | 0.324444 |
| NNMT | -2.92569 | 10.97319 | -4.01173 | 0.00019 | 0.001736 | 0.323939 |
| SUMO2P19 | -1.86354 | 13.08244 | -4.01167 | 0.00019 | 0.001736 | 0.323742 |
| FO082796.1 | -1.51027 | 13.06627 | -4.01018 | 0.000191 | 0.001742 | 0.319161 |
| CCR10 | -1.40797 | 13.15741 | -4.00953 | 0.000192 | 0.001745 | 0.317181 |
| PYGM | -1.00275 | 13.34233 | -4.00873 | 0.000192 | 0.001748 | 0.3147 |
| MLLT3 | 1.050748 | 13.25273 | 4.007411 | 0.000193 | 0.001754 | 0.310658 |
| RHD | -1.31381 | 12.47828 | -4.0067 | 0.000194 | 0.001757 | 0.308485 |
| ATP6V0E2 | 1.047334 | 13.23021 | 4.006128 | 0.000194 | 0.001759 | 0.306712 |
| CHIT1 | -2.54981 | 11.55451 | -4.0044 | 0.000195 | 0.001768 | 0.301398 |
| CKMT1B | -1.9877 | 12.82015 | -4.00355 | 0.000195 | 0.001772 | 0.298793 |
| RAPGEF5 | -1.72051 | 13.06102 | -4.00152 | 0.000197 | 0.001781 | 0.29254 |
| TRBV9 | 1.5314 | 12.9153 | 3.999168 | 0.000198 | 0.001793 | 0.285328 |
| RHOV | -1.76743 | 13.26242 | -3.99877 | 0.000199 | 0.001795 | 0.28409 |
| ANKRD28 | -1.07033 | 13.3006 | -3.99828 | 0.000199 | 0.001796 | 0.282603 |
| TRBV12-3 | 1.768167 | 12.86002 | 3.997879 | 0.000199 | 0.001797 | 0.281368 |
| KCNA5 | 2.598463 | 12.42125 | 3.997104 | 0.0002 | 0.001801 | 0.278987 |
| PDIA4 | -1.01181 | 13.35693 | -3.99568 | 0.000201 | 0.001808 | 0.274611 |
| FCER2 | 1.485498 | 12.99108 | 3.995548 | 0.000201 | 0.001808 | 0.274212 |
| TRHDE | -1.36124 | 12.74894 | -3.99549 | 0.000201 | 0.001808 | 0.274043 |
| KIF5A | 1.028996 | 13.28471 | 3.993901 | 0.000202 | 0.001815 | 0.269155 |
| RPL34P18 | 1.343672 | 13.61542 | 3.993567 | 0.000202 | 0.001817 | 0.26813 |
| CCR7 | 1.529026 | 13.06993 | 3.991788 | 0.000203 | 0.001826 | 0.262672 |
| AC134879.2 | -2.58898 | 11.59128 | -3.99105 | 0.000204 | 0.00183 | 0.260396 |
| AC027176.2 | -1.21653 | 13.35832 | -3.99055 | 0.000204 | 0.001832 | 0.258887 |
| EXOC3L4 | -1.39549 | 13.30653 | -3.99014 | 0.000204 | 0.001834 | 0.257613 |
| RSC1A1 | -4.3331 | 10.37136 | -3.9888 | 0.000205 | 0.001839 | 0.253512 |
| AC104339.1 | 1.156685 | 14.15903 | 3.987802 | 0.000206 | 0.001844 | 0.250443 |
| TDGF1 | -1.03298 | 13.26461 | -3.98741 | 0.000206 | 0.001846 | 0.249243 |
| TRBJ1-3 | 1.853665 | 12.73219 | 3.986544 | 0.000207 | 0.00185 | 0.246585 |
| NR2E1 | -2.4161 | 12.49808 | -3.98434 | 0.000208 | 0.001862 | 0.239832 |
| CACNG2 | -1.60149 | 13.24908 | -3.98309 | 0.000209 | 0.001869 | 0.235992 |
| ZNF154 | 1.035048 | 13.3309 | 3.981921 | 0.00021 | 0.001874 | 0.232415 |
| CCR4 | 1.265183 | 13.08666 | 3.981599 | 0.00021 | 0.001875 | 0.231427 |
| COL10A1 | -2.35726 | 12.13087 | -3.98146 | 0.00021 | 0.001875 | 0.231017 |
| SLC9C2 | 1.449864 | 13.86174 | 3.979997 | 0.000211 | 0.001883 | 0.22652 |
| TPBGL | 1.629869 | 13.20998 | 3.979336 | 0.000211 | 0.001885 | 0.224496 |
| CREB5 | -1.14499 | 13.28875 | -3.97933 | 0.000211 | 0.001885 | 0.224481 |
| GDPD2 | -1.46718 | 13.23813 | -3.97699 | 0.000213 | 0.001898 | 0.217305 |
| DOCK1 | -1.08401 | 13.38518 | -3.97645 | 0.000213 | 0.0019 | 0.215641 |
| BLZF2P | -1.54909 | 13.24365 | -3.97504 | 0.000214 | 0.001908 | 0.211341 |
| NACAP8 | -1.98173 | 12.79845 | -3.97465 | 0.000215 | 0.00191 | 0.210156 |
| TRAJ49 | 1.724427 | 13.02639 | 3.974573 | 0.000215 | 0.00191 | 0.209911 |
| KCNK10 | 1.502667 | 13.1864 | 3.974398 | 0.000215 | 0.00191 | 0.209372 |
| SLCO2A1 | -1.98117 | 12.97246 | -3.9729 | 0.000216 | 0.001915 | 0.204792 |
| TRAJ57 | 1.757711 | 13.00944 | 3.972807 | 0.000216 | 0.001915 | 0.204503 |
| FBXO6 | -1.01415 | 13.42281 | -3.97274 | 0.000216 | 0.001915 | 0.204289 |
| TMEM184A | -1.12501 | 13.1921 | -3.97196 | 0.000217 | 0.001919 | 0.201922 |
| VXN | 1.567014 | 12.86603 | 3.970593 | 0.000217 | 0.001926 | 0.197729 |
| TRAJ53 | 1.771312 | 12.95923 | 3.970523 | 0.000218 | 0.001926 | 0.197513 |
| CBR3 | 1.268649 | 13.07144 | 3.969736 | 0.000218 | 0.00193 | 0.195106 |
| RPS7P4 | 1.479815 | 13.48957 | 3.96784 | 0.000219 | 0.001937 | 0.189308 |
| CD7 | 1.120059 | 13.10833 | 3.966273 | 0.000221 | 0.001945 | 0.184514 |
| INPP4B | 1.674029 | 12.98684 | 3.964606 | 0.000222 | 0.001955 | 0.179416 |
| IGHGP | -2.03677 | 12.454 | -3.9635 | 0.000223 | 0.00196 | 0.176036 |
| SH3GL1P2 | -1.63239 | 13.30382 | -3.96348 | 0.000223 | 0.00196 | 0.175964 |
| CLUL1 | -1.17898 | 13.04543 | -3.96337 | 0.000223 | 0.00196 | 0.175638 |
| CYP2F2P | -1.48311 | 13.06804 | -3.96325 | 0.000223 | 0.001961 | 0.17527 |
| RPS2P7 | 1.701438 | 12.83078 | 3.96247 | 0.000223 | 0.001964 | 0.172888 |
| IGLC6 | -2.53462 | 12.14944 | -3.96229 | 0.000223 | 0.001964 | 0.172346 |
| FAM171B | 1.038144 | 13.30282 | 3.961995 | 0.000224 | 0.001966 | 0.171435 |
| TRBV5-7 | 1.105318 | 14.40104 | 3.960183 | 0.000225 | 0.001973 | 0.1659 |
| MKNK1 | -1.10304 | 13.27362 | -3.95837 | 0.000226 | 0.001983 | 0.160359 |
| AQP11 | 1.06264 | 13.2991 | 3.957834 | 0.000227 | 0.001983 | 0.158723 |
| FBXO39 | -2.93786 | 12.38075 | -3.95778 | 0.000227 | 0.001983 | 0.158565 |
| SPARCL1 | -2.01862 | 12.52225 | -3.95612 | 0.000228 | 0.001993 | 0.15349 |
| TXNRD3 | 1.532531 | 13.08576 | 3.955437 | 0.000228 | 0.001997 | 0.151402 |
| CASP5 | -1.45081 | 13.23526 | -3.95438 | 0.000229 | 0.002001 | 0.148176 |
| UTS2R | -2.01609 | 12.90909 | -3.9537 | 0.00023 | 0.002004 | 0.146101 |
| BMP8B | -1.08567 | 13.32031 | -3.95225 | 0.000231 | 0.00201 | 0.141686 |
| H3C12 | -2.04959 | 12.76923 | -3.94845 | 0.000234 | 0.00203 | 0.130087 |
| GUSBP5 | 1.658427 | 13.12572 | 3.947874 | 0.000234 | 0.002033 | 0.128322 |
| CSGALNACT2 | -1.06954 | 13.24963 | -3.94775 | 0.000234 | 0.002033 | 0.127943 |
| AL354710.1 | 1.058462 | 14.47193 | 3.947468 | 0.000234 | 0.002034 | 0.127082 |
| KLRC2 | 1.667552 | 12.76846 | 3.946796 | 0.000235 | 0.002038 | 0.125031 |
| MS4A2 | 1.596561 | 12.71993 | 3.946442 | 0.000235 | 0.002038 | 0.123954 |
| IQCF3 | -1.35021 | 13.38325 | -3.94553 | 0.000236 | 0.002043 | 0.121183 |
| IGHV3-53 | -2.26479 | 12.52624 | -3.9447 | 0.000236 | 0.002047 | 0.118632 |
| ADAM1A | 2.768398 | 11.37591 | 3.944461 | 0.000237 | 0.002048 | 0.117911 |
| MDK | -1.19177 | 13.22176 | -3.94376 | 0.000237 | 0.002052 | 0.115777 |
| SAMD14 | -1.97912 | 12.50011 | -3.94207 | 0.000238 | 0.002061 | 0.11061 |
| ZBP1 | -1.19632 | 13.37752 | -3.94173 | 0.000239 | 0.002063 | 0.109594 |
| HKDC1 | 1.592777 | 13.08374 | 3.941678 | 0.000239 | 0.002063 | 0.10943 |
| IGHV1OR16-1 | -2.86756 | 12.30316 | -3.94113 | 0.000239 | 0.002065 | 0.107747 |
| LMNTD1 | -1.51969 | 13.2856 | -3.94087 | 0.000239 | 0.002066 | 0.106975 |
| ENTHD1 | 1.782096 | 12.90732 | 3.940867 | 0.000239 | 0.002066 | 0.106956 |
| IGHV2-70 | -3.35187 | 11.71569 | -3.9404 | 0.00024 | 0.002068 | 0.105523 |
| PDLIM1P4 | -1.09208 | 13.38658 | -3.94027 | 0.00024 | 0.002068 | 0.10513 |
| H2AZ2P1 | -1.32604 | 13.31482 | -3.93958 | 0.00024 | 0.002072 | 0.103046 |
| AC068580.4 | -1.69495 | 13.05511 | -3.93952 | 0.00024 | 0.002072 | 0.102854 |
| DLG5 | 1.082716 | 13.22624 | 3.9381 | 0.000242 | 0.00208 | 0.098527 |
| MAP1A | -1.25804 | 13.02887 | -3.93784 | 0.000242 | 0.002081 | 0.097723 |
| RNF175 | -1.00171 | 13.30648 | -3.93665 | 0.000243 | 0.002087 | 0.094097 |
| AC092597.1 | 1.343189 | 13.2847 | 3.935295 | 0.000244 | 0.002095 | 0.089984 |
| TRBV16 | 1.786478 | 12.89324 | 3.934741 | 0.000244 | 0.002098 | 0.088295 |
| TRAJ32 | 1.786943 | 13.03882 | 3.9344 | 0.000244 | 0.002099 | 0.087256 |
| XK | -1.42476 | 12.52147 | -3.934 | 0.000245 | 0.0021 | 0.086045 |
| AL590233.1 | 1.18639 | 13.20367 | 3.933983 | 0.000245 | 0.0021 | 0.085989 |
| LINC01347 | -1.13446 | 13.22996 | -3.93367 | 0.000245 | 0.002102 | 0.085029 |
| LYPD2 | 2.664374 | 10.58119 | 3.932227 | 0.000246 | 0.002109 | 0.080644 |
| VSIG10L | -1.05958 | 13.42602 | -3.93147 | 0.000247 | 0.002113 | 0.078352 |
| AC025588.1 | 1.460203 | 13.68496 | 3.930546 | 0.000247 | 0.002118 | 0.075527 |
| SLC12A8 | -1.22072 | 13.36952 | -3.92858 | 0.000249 | 0.002129 | 0.069539 |
| SERPINI2 | 1.830388 | 12.68744 | 3.927075 | 0.00025 | 0.002138 | 0.064967 |
| MAP9 | 1.316246 | 13.28489 | 3.926859 | 0.00025 | 0.002138 | 0.064308 |
| FANK1 | 1.327606 | 12.53049 | 3.926824 | 0.00025 | 0.002138 | 0.064203 |
| DOC2GP | 1.589971 | 12.91389 | 3.926647 | 0.000251 | 0.002138 | 0.063665 |
| RRAS2 | 1.048336 | 13.36735 | 3.925685 | 0.000251 | 0.002143 | 0.060739 |
| KCNK17 | 1.810249 | 12.63679 | 3.924777 | 0.000252 | 0.002146 | 0.057978 |
| IZUMO1 | -1.27122 | 13.23349 | -3.92463 | 0.000252 | 0.002146 | 0.057524 |
| RPS18P12 | 1.248808 | 13.59418 | 3.92424 | 0.000253 | 0.002147 | 0.056346 |
| ATP8A2P2 | -1.68965 | 13.30788 | -3.92421 | 0.000253 | 0.002147 | 0.056249 |
| IGHV3-66 | -2.50455 | 12.44558 | -3.92414 | 0.000253 | 0.002147 | 0.056042 |
| PLVAP | 1.903979 | 11.42133 | 3.922243 | 0.000254 | 0.002158 | 0.050273 |
| NBPF5P | 1.415054 | 13.14963 | 3.921376 | 0.000255 | 0.002162 | 0.047639 |
| KBTBD12 | -1.33798 | 13.23562 | -3.92136 | 0.000255 | 0.002162 | 0.047581 |
| TRAJ42 | 1.687524 | 13.14054 | 3.919559 | 0.000256 | 0.002172 | 0.042117 |
| SIX5 | -1.05443 | 13.30654 | -3.9176 | 0.000258 | 0.002182 | 0.036156 |
| DOCK3 | 1.106825 | 13.18757 | 3.917487 | 0.000258 | 0.002183 | 0.035823 |
| MTRNR2L8 | 1.021147 | 13.03822 | 3.917066 | 0.000258 | 0.002185 | 0.034543 |
| BCORP1 | 2.10689 | 12.78461 | 3.915319 | 0.00026 | 0.002196 | 0.029236 |
| TMEM108 | -1.08343 | 13.14088 | -3.91513 | 0.00026 | 0.002197 | 0.028664 |
| TRGV10 | 1.586618 | 12.98581 | 3.913853 | 0.000261 | 0.002205 | 0.024784 |
| ROPN1L | -1.24574 | 13.07682 | -3.91184 | 0.000263 | 0.002217 | 0.018668 |
| NSA2P2 | -1.68843 | 13.07623 | -3.90754 | 0.000266 | 0.002246 | 0.005625 |
| IGHV1OR16-4 | -2.54021 | 12.52846 | -3.90683 | 0.000267 | 0.002249 | 0.003464 |
| FGF9 | 1.587309 | 13.11911 | 3.904962 | 0.000269 | 0.002261 | -0.00219 |
| AC138409.1 | -2.13232 | 12.87105 | -3.90456 | 0.000269 | 0.002263 | -0.00341 |
| LZTS3 | 1.016137 | 13.19675 | 3.902684 | 0.000271 | 0.002274 | -0.0091 |
| FSTL1 | -1.63494 | 12.72122 | -3.90188 | 0.000271 | 0.002279 | -0.01154 |
| MDFIC | 1.110935 | 13.27859 | 3.90106 | 0.000272 | 0.002282 | -0.01402 |
| AGAP7P | 1.553882 | 12.75741 | 3.900826 | 0.000272 | 0.002282 | -0.01473 |
| EEF1DP1 | 1.155138 | 14.24306 | 3.900515 | 0.000272 | 0.002284 | -0.01567 |
| TRAV8-1 | 1.767613 | 12.8363 | 3.90043 | 0.000273 | 0.002284 | -0.01593 |
| IGKV1D-39 | -2.47008 | 12.46072 | -3.90027 | 0.000273 | 0.002285 | -0.01642 |
| IL4R | -1.00192 | 13.2824 | -3.89978 | 0.000273 | 0.002288 | -0.01791 |
| NXPH4 | 1.810524 | 12.82326 | 3.899658 | 0.000273 | 0.002288 | -0.01827 |
| CPNE7 | 1.629343 | 12.908 | 3.899521 | 0.000273 | 0.002288 | -0.01869 |
| TRBV11-3 | 1.80871 | 12.8272 | 3.89923 | 0.000274 | 0.002288 | -0.01957 |
| IGHV3-42 | -2.34168 | 10.6658 | -3.89919 | 0.000274 | 0.002288 | -0.01967 |
| ZSWIM5 | 1.457794 | 13.03353 | 3.898708 | 0.000274 | 0.002291 | -0.02115 |
| TRDC | 1.865444 | 12.83376 | 3.898338 | 0.000274 | 0.002293 | -0.02227 |
| RNASE3 | -2.10863 | 12.10952 | -3.89821 | 0.000275 | 0.002293 | -0.02266 |
| AIF1L | -1.83062 | 12.41001 | -3.89736 | 0.000275 | 0.002298 | -0.02524 |
| SAMSN1 | -1.21536 | 13.25372 | -3.89623 | 0.000276 | 0.002306 | -0.02865 |
| TRAV17 | 1.606225 | 12.94377 | 3.895237 | 0.000277 | 0.002313 | -0.03166 |
| IQCA1 | 1.991572 | 13.11408 | 3.894502 | 0.000278 | 0.002318 | -0.03389 |
| PPP2R2C | -1.34043 | 13.45404 | -3.89431 | 0.000278 | 0.002318 | -0.03448 |
| AC244034.1 | 1.450085 | 13.23447 | 3.893857 | 0.000278 | 0.00232 | -0.03584 |
| CR2 | 1.192732 | 13.27795 | 3.892984 | 0.000279 | 0.002326 | -0.03848 |
| AC229888.1 | 1.683261 | 12.82442 | 3.892842 | 0.000279 | 0.002327 | -0.03891 |
| AC013474.1 | -1.56528 | 12.8534 | -3.89178 | 0.00028 | 0.002333 | -0.04213 |
| ANKRD34B | -2.28135 | 12.77226 | -3.89149 | 0.00028 | 0.002335 | -0.04301 |
| SPRED1 | 1.362167 | 12.97064 | 3.890944 | 0.000281 | 0.002338 | -0.04466 |
| AL163636.2 | -1.35774 | 13.14305 | -3.89006 | 0.000282 | 0.002344 | -0.04734 |
| TRAV3 | 1.593116 | 12.88984 | 3.888512 | 0.000283 | 0.002353 | -0.05202 |
| PNPLA5 | -1.28253 | 13.39436 | -3.88713 | 0.000284 | 0.002361 | -0.05621 |
| INHBB | -2.15857 | 12.57306 | -3.88709 | 0.000284 | 0.002361 | -0.05631 |
| SEMA6B | -1.62004 | 12.78341 | -3.88708 | 0.000284 | 0.002361 | -0.05636 |
| TTC25 | 3.057967 | 10.69721 | 3.88545 | 0.000286 | 0.002371 | -0.06128 |
| MAGED4B | 1.50842 | 13.0631 | 3.884759 | 0.000287 | 0.002375 | -0.06337 |
| WSCD1 | 1.824547 | 13.1599 | 3.881143 | 0.00029 | 0.0024 | -0.0743 |
| AL109809.1 | -1.34778 | 13.20912 | -3.87962 | 0.000291 | 0.002409 | -0.07891 |
| LHFPL4 | -1.43742 | 13.33591 | -3.87904 | 0.000292 | 0.002412 | -0.08064 |
| TRAJ23 | 1.68034 | 13.11969 | 3.878805 | 0.000292 | 0.002412 | -0.08137 |
| NR4A1 | 1.368753 | 12.61541 | 3.878599 | 0.000292 | 0.002413 | -0.08199 |
| GSTA1 | 2.182369 | 12.06739 | 3.87785 | 0.000293 | 0.002417 | -0.08425 |
| AC110056.1 | 1.021649 | 14.34455 | 3.877563 | 0.000293 | 0.002417 | -0.08512 |
| AC005480.2 | 1.24879 | 13.94807 | 3.876095 | 0.000295 | 0.002426 | -0.08955 |
| TRAV26-1 | 1.401288 | 13.00759 | 3.875827 | 0.000295 | 0.002427 | -0.09036 |
| KIAA1324L | 1.170093 | 13.23764 | 3.874463 | 0.000296 | 0.002437 | -0.09448 |
| FSTL3 | -1.35984 | 12.58098 | -3.87405 | 0.000297 | 0.002439 | -0.09571 |
| TIMM8AP1 | 1.93913 | 12.88741 | 3.870191 | 0.0003 | 0.002463 | -0.10737 |
| FAM221B | 1.560858 | 13.16624 | 3.870155 | 0.0003 | 0.002463 | -0.10748 |
| RPL27AP5 | 1.014763 | 14.2651 | 3.86954 | 0.000301 | 0.002467 | -0.10934 |
| ZAP70 | 1.134373 | 13.11363 | 3.868828 | 0.000302 | 0.002471 | -0.11149 |
| C11orf42 | -1.49806 | 13.12811 | -3.86752 | 0.000303 | 0.002481 | -0.11543 |
| TRAJ52 | 1.606709 | 13.04861 | 3.867297 | 0.000303 | 0.002482 | -0.1161 |
| ATP5PBP5 | 1.49279 | 13.28444 | 3.866243 | 0.000304 | 0.002488 | -0.11929 |
| GAS2L2 | 1.417838 | 13.44989 | 3.865179 | 0.000305 | 0.002494 | -0.12249 |
| TWIST2 | -2.55964 | 11.43687 | -3.86377 | 0.000306 | 0.0025 | -0.12674 |
| MAP1B | -1.75908 | 12.60057 | -3.86283 | 0.000307 | 0.002506 | -0.12958 |
| TNNI3K | 1.786626 | 13.08594 | 3.862473 | 0.000308 | 0.002508 | -0.13065 |
| MYRIP | -1.47706 | 13.22199 | -3.8617 | 0.000308 | 0.002513 | -0.13298 |
| ACE | 1.095165 | 13.16341 | 3.860785 | 0.000309 | 0.002519 | -0.13574 |
| AL034379.1 | 1.070707 | 13.09696 | 3.860752 | 0.000309 | 0.002519 | -0.13584 |
| AL034430.1 | -3.80203 | 11.47688 | -3.85925 | 0.000311 | 0.002527 | -0.14035 |
| COL23A1 | -1.61491 | 12.7315 | -3.85855 | 0.000312 | 0.002532 | -0.14248 |
| SFN | -1.02775 | 13.41415 | -3.85822 | 0.000312 | 0.002533 | -0.14347 |
| TGFB1I1 | -1.47234 | 12.81472 | -3.85819 | 0.000312 | 0.002533 | -0.14355 |
| AC125388.1 | 1.363223 | 13.19102 | 3.857979 | 0.000312 | 0.002534 | -0.14419 |
| AC138811.2 | -3.55661 | 11.29431 | -3.85727 | 0.000313 | 0.002538 | -0.14632 |
| CYP2B7P | 1.185071 | 13.19022 | 3.856988 | 0.000313 | 0.002539 | -0.14718 |
| ITGB5 | -1.21683 | 13.07506 | -3.85578 | 0.000314 | 0.002544 | -0.15082 |
| STOX2 | -2.11783 | 11.83496 | -3.85572 | 0.000314 | 0.002544 | -0.151 |
| CALD1 | -1.02603 | 13.1735 | -3.85488 | 0.000315 | 0.00255 | -0.15352 |
| PHLDB2 | 1.260554 | 13.24421 | 3.85447 | 0.000316 | 0.002553 | -0.15476 |
| FLVCR2 | -1.02234 | 13.25956 | -3.85413 | 0.000316 | 0.002554 | -0.15579 |
| NEO1 | 1.034159 | 13.27552 | 3.854071 | 0.000316 | 0.002554 | -0.15596 |
| HTR7 | 1.486435 | 13.06029 | 3.852314 | 0.000318 | 0.002566 | -0.16125 |
| P2RY1 | -1.20923 | 13.13098 | -3.85223 | 0.000318 | 0.002566 | -0.16149 |
| SLC4A4 | 1.696328 | 13.00389 | 3.85089 | 0.000319 | 0.002574 | -0.16553 |
| MSX2P1 | 1.380411 | 13.04086 | 3.850597 | 0.00032 | 0.002575 | -0.16642 |
| CFL1P5 | -1.28137 | 13.16725 | -3.8501 | 0.00032 | 0.002579 | -0.16791 |
| SAMD3 | 1.278214 | 13.14735 | 3.849634 | 0.000321 | 0.00258 | -0.16931 |
| NECTIN2 | -1.65889 | 13.01382 | -3.84891 | 0.000321 | 0.002584 | -0.1715 |
| SLC34A2 | -1.21044 | 13.2999 | -3.84815 | 0.000322 | 0.00259 | -0.17379 |
| ST8SIA1 | 1.073174 | 13.11569 | 3.847634 | 0.000323 | 0.002591 | -0.17533 |
| LLCFC1 | -1.57184 | 13.18072 | -3.84761 | 0.000323 | 0.002591 | -0.1754 |
| IGHV3OR15-7 | -2.59793 | 11.92793 | -3.84758 | 0.000323 | 0.002591 | -0.1755 |
| IGKV1-39 | -3.24577 | 12.10932 | -3.84655 | 0.000324 | 0.002598 | -0.17858 |
| AC114878.2 | -1.82084 | 13.06207 | -3.84644 | 0.000324 | 0.002598 | -0.17892 |
| MS4A14 | 1.283556 | 12.83908 | 3.845982 | 0.000324 | 0.002602 | -0.1803 |
| ADAMTS17 | 1.067915 | 13.14861 | 3.845538 | 0.000325 | 0.002604 | -0.18163 |
| CCDC162P | -1.45311 | 12.96257 | -3.84496 | 0.000325 | 0.002607 | -0.18336 |
| FAM153A | 1.722651 | 12.89841 | 3.844841 | 0.000326 | 0.002607 | -0.18373 |
| EEF1GP1 | 2.700189 | 12.45761 | 3.843719 | 0.000327 | 0.002614 | -0.1871 |
| C11orf87 | -1.85251 | 13.03659 | -3.8437 | 0.000327 | 0.002614 | -0.18717 |
| AC010343.1 | 1.470916 | 13.28267 | 3.843373 | 0.000327 | 0.002616 | -0.18814 |
| IGHA2 | -1.83498 | 12.76355 | -3.84334 | 0.000327 | 0.002616 | -0.18824 |
| BACH2 | 1.351821 | 13.21591 | 3.842972 | 0.000327 | 0.002618 | -0.18935 |
| TMEM88 | -1.20379 | 13.12436 | -3.84284 | 0.000328 | 0.002619 | -0.18973 |
| CLEC4E | -1.22274 | 13.23351 | -3.84176 | 0.000329 | 0.002624 | -0.19299 |
| IL2RB | 1.304026 | 13.17283 | 3.840636 | 0.00033 | 0.002632 | -0.19637 |
| AC136632.2 | 1.348975 | 13.67614 | 3.840359 | 0.00033 | 0.002634 | -0.1972 |
| LPAR6 | 1.141369 | 13.16337 | 3.837796 | 0.000333 | 0.002653 | -0.2049 |
| TEAD3 | -1.66713 | 13.02645 | -3.83778 | 0.000333 | 0.002653 | -0.20496 |
| KLHL29 | 1.039528 | 13.26117 | 3.837507 | 0.000333 | 0.002654 | -0.20577 |
| TGFBR3 | 1.550164 | 13.07302 | 3.83732 | 0.000333 | 0.002655 | -0.20633 |
| MS4A1 | 1.423427 | 13.16859 | 3.83696 | 0.000334 | 0.002658 | -0.20741 |
| NRP2 | 1.212154 | 13.08638 | 3.83634 | 0.000334 | 0.002662 | -0.20927 |
| AL031229.1 | 1.217855 | 14.12529 | 3.834798 | 0.000336 | 0.002673 | -0.2139 |
| FAM178B | 1.023502 | 13.01079 | 3.833937 | 0.000337 | 0.002678 | -0.21649 |
| HSPA8P8 | 1.237006 | 14.07531 | 3.83233 | 0.000339 | 0.00269 | -0.22131 |
| TMEM132E | 1.723326 | 13.07203 | 3.831448 | 0.00034 | 0.002695 | -0.22396 |
| AC069213.3 | 1.174937 | 13.92988 | 3.830689 | 0.00034 | 0.0027 | -0.22624 |
| CBX1P4 | -1.80063 | 13.29462 | -3.82911 | 0.000342 | 0.002711 | -0.23097 |
| AC044787.1 | 1.610491 | 13.21847 | 3.827993 | 0.000343 | 0.002719 | -0.23432 |
| ADGRB2 | 1.283327 | 13.07469 | 3.82689 | 0.000345 | 0.002727 | -0.23763 |
| MPIG6B | -1.26077 | 13.01346 | -3.8267 | 0.000345 | 0.002728 | -0.23821 |
| ODF3B | -1.16849 | 13.24914 | -3.82538 | 0.000346 | 0.002738 | -0.24215 |
| AC018868.1 | 1.392023 | 13.1771 | 3.825091 | 0.000347 | 0.002739 | -0.24303 |
| CNN3 | 1.490552 | 12.954 | 3.823842 | 0.000348 | 0.002746 | -0.24677 |
| HSPE1P18 | -1.37777 | 13.02696 | -3.82206 | 0.00035 | 0.002759 | -0.25212 |
| AC108022.2 | 1.028453 | 14.71684 | 3.821514 | 0.000351 | 0.002762 | -0.25375 |
| HPD | -1.43072 | 12.88511 | -3.81884 | 0.000354 | 0.002778 | -0.26177 |
| FAAH2 | 1.281064 | 13.12448 | 3.816359 | 0.000356 | 0.002797 | -0.26919 |
| FCGBP | 1.788613 | 12.86107 | 3.815882 | 0.000357 | 0.002799 | -0.27061 |
| NRADDP | -1.10148 | 13.16347 | -3.81585 | 0.000357 | 0.002799 | -0.27071 |
| CICP18 | -1.10411 | 13.21942 | -3.81559 | 0.000357 | 0.002801 | -0.2715 |
| OVOL1 | -1.99106 | 13.21452 | -3.81441 | 0.000359 | 0.00281 | -0.27503 |
| TRAV24 | 1.489956 | 12.92088 | 3.813363 | 0.00036 | 0.002816 | -0.27816 |
| GJA4 | -1.79515 | 12.85159 | -3.81117 | 0.000362 | 0.002831 | -0.28472 |
| KCNT2 | -1.97302 | 12.60354 | -3.81082 | 0.000363 | 0.002833 | -0.28578 |
| HLA-DOB | 1.050074 | 13.32029 | 3.810257 | 0.000363 | 0.002837 | -0.28745 |
| PYHIN1 | 1.337102 | 13.15259 | 3.810006 | 0.000364 | 0.002838 | -0.2882 |
| KCNG1 | 1.477748 | 12.96829 | 3.809271 | 0.000364 | 0.002843 | -0.2904 |
| PPIAP29 | -1.34941 | 13.17772 | -3.80908 | 0.000365 | 0.002844 | -0.29097 |
| KRT2 | 1.916634 | 12.91333 | 3.807883 | 0.000366 | 0.002853 | -0.29455 |
| LRRC7 | 1.188011 | 13.22803 | 3.807102 | 0.000367 | 0.002858 | -0.29689 |
| TM4SF20 | -1.40357 | 13.32789 | -3.80589 | 0.000368 | 0.002868 | -0.3005 |
| RGN | -1.39378 | 13.42362 | -3.80535 | 0.000369 | 0.002871 | -0.30213 |
| DCHS1 | 1.149443 | 13.11728 | 3.804113 | 0.00037 | 0.00288 | -0.30582 |
| CTSW | 1.421153 | 13.10434 | 3.804097 | 0.00037 | 0.00288 | -0.30587 |
| RPS10P28 | 1.059421 | 14.1613 | 3.80245 | 0.000372 | 0.002892 | -0.31079 |
| TRAJ18 | 1.753906 | 13.10781 | 3.80212 | 0.000373 | 0.002894 | -0.31178 |
| H2BW3P | 1.597268 | 12.93555 | 3.801171 | 0.000374 | 0.002901 | -0.31461 |
| STAC2 | -1.00066 | 13.29775 | -3.80056 | 0.000375 | 0.002905 | -0.31644 |
| RPL30P4 | 1.145672 | 14.27875 | 3.797845 | 0.000378 | 0.002925 | -0.32454 |
| SLC7A3 | 1.920528 | 13.17065 | 3.796601 | 0.000379 | 0.002934 | -0.32826 |
| AC091564.1 | 1.334159 | 13.0551 | 3.796307 | 0.00038 | 0.002936 | -0.32914 |
| ZNF80 | 1.428748 | 12.72706 | 3.793976 | 0.000383 | 0.002953 | -0.3361 |
| TMEM54 | -2.10805 | 12.66618 | -3.79388 | 0.000383 | 0.002953 | -0.3364 |
| YPEL1 | 1.053046 | 13.24625 | 3.793752 | 0.000383 | 0.002953 | -0.33676 |
| PLPPR3 | -2.54971 | 11.49285 | -3.7926 | 0.000384 | 0.002961 | -0.3402 |
| ICOS | 1.29531 | 13.16449 | 3.792373 | 0.000384 | 0.002962 | -0.34088 |
| TRAJ48 | 1.828591 | 12.9595 | 3.792266 | 0.000385 | 0.002962 | -0.3412 |
| SPCS2P4 | -1.22007 | 13.28957 | -3.79214 | 0.000385 | 0.002962 | -0.34156 |
| BCAR1 | -1.85897 | 12.83559 | -3.79211 | 0.000385 | 0.002962 | -0.34166 |
| LIPH | -1.46137 | 12.93466 | -3.7921 | 0.000385 | 0.002962 | -0.3417 |
| VSTM2L | -1.8899 | 12.75453 | -3.79161 | 0.000385 | 0.002965 | -0.34316 |
| H1-3 | -1.55723 | 13.24034 | -3.79007 | 0.000387 | 0.002977 | -0.34775 |
| PRSS23 | 1.119306 | 13.1601 | 3.787903 | 0.00039 | 0.002993 | -0.35421 |
| TSPAN6 | 1.240455 | 13.27251 | 3.787735 | 0.00039 | 0.002993 | -0.35471 |
| HPCAL4 | 1.613608 | 12.94257 | 3.787296 | 0.000391 | 0.002995 | -0.35602 |
| AC103810.3 | -1.68941 | 13.05057 | -3.7868 | 0.000391 | 0.002998 | -0.35751 |
| EFCAB12 | -1.10122 | 13.28627 | -3.78624 | 0.000392 | 0.003002 | -0.35917 |
| H2BC19P | -1.98879 | 13.00181 | -3.78597 | 0.000392 | 0.003003 | -0.35998 |
| PPIAL4C | 2.046796 | 11.9487 | 3.785129 | 0.000393 | 0.003011 | -0.36248 |
| TMPRSS2 | -1.91322 | 13.23877 | -3.78383 | 0.000395 | 0.003021 | -0.36634 |
| AGAP1 | 1.284833 | 13.10315 | 3.783044 | 0.000396 | 0.003026 | -0.36869 |
| TRAJ11 | 1.871451 | 13.13944 | 3.782909 | 0.000396 | 0.003026 | -0.36909 |
| TUBB4A | 1.667146 | 13.00583 | 3.781406 | 0.000398 | 0.003037 | -0.37357 |
| CD69 | 1.083599 | 13.31611 | 3.780227 | 0.0004 | 0.003048 | -0.37708 |
| LRP3 | -1.04633 | 12.80967 | -3.78006 | 0.0004 | 0.003048 | -0.37758 |
| CD274 | -1.44212 | 13.34659 | -3.77842 | 0.000402 | 0.003059 | -0.38246 |
| SPATA1 | -1.05671 | 13.18806 | -3.77742 | 0.000403 | 0.003065 | -0.38544 |
| TPRG1 | 1.000092 | 13.25676 | 3.776745 | 0.000404 | 0.00307 | -0.38745 |
| PGS1 | -1.03502 | 13.27111 | -3.77654 | 0.000404 | 0.003072 | -0.38807 |
| AC092155.2 | 1.250185 | 13.58535 | 3.776441 | 0.000404 | 0.003072 | -0.38836 |
| RNF208 | -1.31348 | 13.08749 | -3.7757 | 0.000405 | 0.003078 | -0.39056 |
| THY1 | 1.44307 | 13.75447 | 3.775399 | 0.000406 | 0.00308 | -0.39146 |
| MTND5P12 | -1.68862 | 13.07367 | -3.7745 | 0.000407 | 0.003088 | -0.39414 |
| EEF1A1P10 | 1.180838 | 13.4264 | 3.774013 | 0.000407 | 0.00309 | -0.39558 |
| COL1A1 | -1.56917 | 13.10212 | -3.77274 | 0.000409 | 0.003099 | -0.39938 |
| HSPB6 | -1.97987 | 12.64141 | -3.77239 | 0.00041 | 0.0031 | -0.40042 |
| DDR1 | 1.035712 | 13.19944 | 3.772241 | 0.00041 | 0.003101 | -0.40085 |
| AC007207.1 | -1.70137 | 13.52217 | -3.77 | 0.000413 | 0.00312 | -0.40752 |
| SLC4A7 | 1.122035 | 13.26104 | 3.769619 | 0.000413 | 0.003123 | -0.40865 |
| SIRT4 | 1.001632 | 13.34396 | 3.767975 | 0.000415 | 0.003134 | -0.41354 |
| RPGRIP1 | 1.112957 | 13.15005 | 3.767655 | 0.000416 | 0.003137 | -0.41449 |
| AC010522.1 | -1.51265 | 13.17241 | -3.76664 | 0.000417 | 0.003145 | -0.41751 |
| DSCAML1 | 1.933556 | 12.80704 | 3.765792 | 0.000418 | 0.003151 | -0.42003 |
| MT1G | -1.89116 | 12.25564 | -3.76507 | 0.000419 | 0.003157 | -0.42218 |
| IGKV2D-40 | -2.32228 | 12.34908 | -3.76443 | 0.00042 | 0.003161 | -0.42407 |
| RGS1 | 1.380584 | 12.87814 | 3.763933 | 0.000421 | 0.003165 | -0.42555 |
| ERFE | -2.34986 | 9.860313 | -3.76321 | 0.000422 | 0.003171 | -0.4277 |
| BCAN | -1.55734 | 13.2188 | -3.76123 | 0.000424 | 0.003187 | -0.43357 |
| C8orf86 | -1.1595 | 13.43628 | -3.76088 | 0.000425 | 0.003189 | -0.43463 |
| ADAMTS9 | -1.88382 | 12.22336 | -3.76006 | 0.000426 | 0.003196 | -0.43706 |
| IGHV6-1 | -3.21126 | 10.80125 | -3.75638 | 0.000431 | 0.003231 | -0.44799 |
| AL355877.2 | -1.63609 | 12.94343 | -3.75613 | 0.000431 | 0.003232 | -0.44871 |
| H2AC8 | -1.41262 | 13.05164 | -3.75491 | 0.000433 | 0.003243 | -0.45233 |
| RNF103-CHMP3 | -2.16021 | 12.07141 | -3.75353 | 0.000435 | 0.003257 | -0.45643 |
| ZNF469 | 1.021478 | 13.18991 | 3.751421 | 0.000437 | 0.003277 | -0.46269 |
| NALCN | -1.2847 | 13.18487 | -3.74729 | 0.000443 | 0.003314 | -0.47492 |
| ZNRF2P2 | -1.27981 | 13.18518 | -3.74712 | 0.000443 | 0.003314 | -0.47544 |
| SYTL4 | -1.18767 | 12.99919 | -3.74538 | 0.000446 | 0.00333 | -0.48059 |
| BEX5 | 1.248189 | 13.16867 | 3.745306 | 0.000446 | 0.00333 | -0.48081 |
| RTN4R | 1.130272 | 13.00384 | 3.743614 | 0.000448 | 0.003344 | -0.48582 |
| CCDC17 | -1.1701 | 13.16291 | -3.74325 | 0.000449 | 0.003347 | -0.48691 |
| TRAV13-1 | 1.436295 | 12.97202 | 3.739464 | 0.000454 | 0.003383 | -0.49811 |
| P3H3 | 1.422268 | 12.88466 | 3.73872 | 0.000455 | 0.003389 | -0.50031 |
| MED12L | -1.05997 | 13.17665 | -3.73692 | 0.000458 | 0.003405 | -0.50563 |
| DAZL | 1.788293 | 13.03783 | 3.736227 | 0.000459 | 0.003411 | -0.50769 |
| CC2D2B | -1.02512 | 13.22841 | -3.7361 | 0.000459 | 0.003411 | -0.50807 |
| C4orf48 | -1.35431 | 12.89102 | -3.73573 | 0.00046 | 0.003413 | -0.50914 |
| AL035456.1 | -1.70617 | 13.14953 | -3.73536 | 0.00046 | 0.003416 | -0.51026 |
| SLC8A2 | -2.26122 | 12.00741 | -3.73227 | 0.000465 | 0.003446 | -0.5194 |
| TRDJ3 | 2.034206 | 12.73712 | 3.731431 | 0.000466 | 0.003453 | -0.52187 |
| GDF15 | -1.67006 | 10.27817 | -3.73139 | 0.000466 | 0.003453 | -0.522 |
| MTRNR2L6 | 1.051393 | 12.9454 | 3.730622 | 0.000467 | 0.003459 | -0.52426 |
| HNRNPMP1 | -1.49227 | 13.34509 | -3.72818 | 0.000471 | 0.00348 | -0.53147 |
| FAM169A | 1.64162 | 13.09695 | 3.727595 | 0.000471 | 0.003484 | -0.53321 |
| SORCS2 | -1.35498 | 12.92449 | -3.72615 | 0.000474 | 0.003498 | -0.53749 |
| KCNH8 | 1.440664 | 13.1309 | 3.724373 | 0.000476 | 0.003514 | -0.54272 |
| ZNF709 | 1.268547 | 13.22331 | 3.723311 | 0.000478 | 0.003524 | -0.54586 |
| IGKV2D-29 | -2.24669 | 10.99245 | -3.72262 | 0.000479 | 0.003531 | -0.54789 |
| TRAT1 | 1.507199 | 13.05498 | 3.722462 | 0.000479 | 0.003532 | -0.54836 |
| PRYP4 | 1.233086 | 13.7704 | 3.71881 | 0.000485 | 0.003568 | -0.55914 |
| MYEF2 | 1.109256 | 13.32429 | 3.718349 | 0.000485 | 0.003572 | -0.5605 |
| ITGB4 | -1.73574 | 12.92173 | -3.71766 | 0.000486 | 0.003577 | -0.56254 |
| NUDT7 | 1.042829 | 13.27165 | 3.715887 | 0.000489 | 0.003594 | -0.56776 |
| PAGE2B | 2.480816 | 11.06143 | 3.715763 | 0.000489 | 0.003594 | -0.56813 |
| ABCA13 | -2.49277 | 11.50283 | -3.71558 | 0.00049 | 0.003596 | -0.56868 |
| GALNT15 | -1.46932 | 13.06108 | -3.71509 | 0.00049 | 0.0036 | -0.57012 |
| NAIPP3 | -1.82639 | 13.12811 | -3.71328 | 0.000493 | 0.003617 | -0.57545 |
| CCN3 | 1.678362 | 12.65817 | 3.710972 | 0.000497 | 0.003641 | -0.58225 |
| XKR3 | -2.29232 | 12.53127 | -3.70987 | 0.000498 | 0.003652 | -0.5855 |
| ASGR2 | -1.01011 | 13.15185 | -3.70963 | 0.000499 | 0.003653 | -0.5862 |
| HPSE2 | 1.587781 | 12.90704 | 3.709129 | 0.0005 | 0.003656 | -0.58768 |
| ARHGAP19-SLIT1 | -2.17979 | 12.66157 | -3.7088 | 0.0005 | 0.003657 | -0.58864 |
| IRF7 | -1.38966 | 13.2092 | -3.70863 | 0.0005 | 0.003658 | -0.58917 |
| AC099811.2 | -2.6736 | 12.67651 | -3.70738 | 0.000502 | 0.003671 | -0.59283 |
| EEF1B2P6 | 1.480888 | 13.18826 | 3.707203 | 0.000503 | 0.003672 | -0.59336 |
| AC112191.1 | -1.46291 | 13.31389 | -3.70598 | 0.000504 | 0.003683 | -0.59695 |
| ARMC12 | -1.86491 | 12.81971 | -3.70516 | 0.000506 | 0.00369 | -0.59938 |
| RPL13AP3 | 1.502813 | 13.31525 | 3.704064 | 0.000508 | 0.003699 | -0.6026 |
| RPL13AP20 | 1.183272 | 13.33857 | 3.703679 | 0.000508 | 0.003701 | -0.60373 |
| FRMD3 | -1.11734 | 13.27238 | -3.70327 | 0.000509 | 0.003705 | -0.60493 |
| CDS1 | 1.235255 | 13.12376 | 3.700378 | 0.000513 | 0.003736 | -0.61344 |
| PARP9 | -1.04393 | 13.4218 | -3.69995 | 0.000514 | 0.00374 | -0.61469 |
| ABCC6P2 | -2.30802 | 12.26409 | -3.69885 | 0.000516 | 0.003751 | -0.61793 |
| LINC00514 | -1.46712 | 13.01252 | -3.69874 | 0.000516 | 0.003752 | -0.61828 |
| TMEM52B | -1.55863 | 12.41015 | -3.6982 | 0.000517 | 0.003757 | -0.61984 |
| TRAJ58 | 1.610183 | 13.00782 | 3.69726 | 0.000518 | 0.003765 | -0.62261 |
| DNM1P34 | -1.67935 | 13.22747 | -3.69655 | 0.00052 | 0.003772 | -0.62471 |
| NFATC2 | 1.191556 | 13.22085 | 3.696248 | 0.00052 | 0.003775 | -0.62559 |
| EEF1A1P26 | 1.136494 | 14.08539 | 3.696086 | 0.00052 | 0.003776 | -0.62607 |
| ADAMTS7P1 | 1.99417 | 12.14497 | 3.695716 | 0.000521 | 0.003779 | -0.62715 |
| PRDX3P1 | -1.05216 | 13.07387 | -3.69497 | 0.000522 | 0.003785 | -0.62934 |
| AC069262.1 | -1.56149 | 13.18485 | -3.69448 | 0.000523 | 0.003789 | -0.63079 |
| LRIT2 | 1.118509 | 14.32014 | 3.694407 | 0.000523 | 0.003789 | -0.631 |
| FAM20C | -1.04565 | 13.14695 | -3.69394 | 0.000524 | 0.003793 | -0.63237 |
| EIF4HP2 | 1.301912 | 12.42251 | 3.693385 | 0.000525 | 0.003798 | -0.634 |
| EPDR1 | -1.08909 | 13.20341 | -3.69245 | 0.000526 | 0.003804 | -0.63674 |
| NFIB | -1.16093 | 12.9222 | -3.6924 | 0.000526 | 0.003804 | -0.63691 |
| AC141557.1 | -1.15798 | 13.09328 | -3.69113 | 0.000528 | 0.003815 | -0.64063 |
| ASB18 | -1.14516 | 13.24599 | -3.69095 | 0.000529 | 0.003817 | -0.64117 |
| MTND5P14 | -1.62243 | 13.12554 | -3.68957 | 0.000531 | 0.003831 | -0.6452 |
| ZNF429 | -1.19456 | 13.33843 | -3.68954 | 0.000531 | 0.003831 | -0.64529 |
| AC010886.1 | -1.49214 | 12.99079 | -3.6889 | 0.000532 | 0.003836 | -0.64718 |
| LARGE2 | 1.25012 | 13.14107 | 3.688413 | 0.000533 | 0.00384 | -0.64861 |
| CYP2S1 | 1.390715 | 12.81959 | 3.68771 | 0.000534 | 0.003847 | -0.65067 |
| SLC35G3 | 1.559833 | 13.43913 | 3.687357 | 0.000535 | 0.00385 | -0.65171 |
| SYTL2 | 1.321844 | 13.17188 | 3.684459 | 0.00054 | 0.003881 | -0.66022 |
| TRGV4 | 1.708276 | 12.94888 | 3.683686 | 0.000541 | 0.003889 | -0.66248 |
| TCEA3 | 1.527719 | 12.978 | 3.68293 | 0.000542 | 0.003897 | -0.6647 |
| AL442663.4 | 1.592338 | 13.04752 | 3.681968 | 0.000544 | 0.003907 | -0.66752 |
| LSM12P1 | -2.92254 | 12.55439 | -3.68188 | 0.000544 | 0.003907 | -0.6678 |
| TRBV1 | 1.893235 | 12.79162 | 3.680323 | 0.000547 | 0.003924 | -0.67235 |
| TRAJ38 | 1.75319 | 13.08319 | 3.680219 | 0.000547 | 0.003924 | -0.67265 |
| H2AC21 | -1.15399 | 13.87123 | -3.68017 | 0.000547 | 0.003924 | -0.67279 |
| ZNF280B | 1.054911 | 13.3239 | 3.676229 | 0.000554 | 0.003967 | -0.68435 |
| AC020636.2 | -2.27985 | 12.60672 | -3.67612 | 0.000554 | 0.003967 | -0.68468 |
| COL13A1 | 2.079626 | 12.77689 | 3.675587 | 0.000555 | 0.003971 | -0.68623 |
| MCOLN2 | 1.060382 | 13.21343 | 3.675401 | 0.000555 | 0.003972 | -0.68678 |
| SLC6A17 | -1.05083 | 13.18975 | -3.67307 | 0.000559 | 0.003997 | -0.69361 |
| NWD1 | -1.02711 | 13.36528 | -3.67142 | 0.000562 | 0.004013 | -0.69843 |
| SPIB | 1.182926 | 13.17439 | 3.671273 | 0.000562 | 0.004014 | -0.69887 |
| RETREG1 | 1.146185 | 13.34215 | 3.670457 | 0.000564 | 0.004023 | -0.70126 |
| BDKRB1 | -1.00241 | 13.3336 | -3.66978 | 0.000565 | 0.00403 | -0.70324 |
| AP000936.3 | 1.396875 | 13.23862 | 3.669747 | 0.000565 | 0.00403 | -0.70334 |
| CYSTM1 | -1.34083 | 12.69439 | -3.66657 | 0.000571 | 0.004064 | -0.71264 |
| ATP5PDP4 | -1.64115 | 12.91264 | -3.66562 | 0.000572 | 0.004074 | -0.71542 |
| HRH4 | 1.742594 | 12.54299 | 3.66517 | 0.000573 | 0.004076 | -0.71673 |
| B4GALNT4 | 1.734633 | 12.77984 | 3.664633 | 0.000574 | 0.004081 | -0.7183 |
| TSSK1A | -1.11933 | 13.36173 | -3.66171 | 0.000579 | 0.004114 | -0.72685 |
| HSF2BP | 1.441564 | 13.16958 | 3.659735 | 0.000583 | 0.004133 | -0.73262 |
| PLAU | -1.43995 | 12.58353 | -3.6594 | 0.000583 | 0.004137 | -0.73361 |
| TTLL2 | 1.15923 | 13.27906 | 3.657962 | 0.000586 | 0.004151 | -0.73781 |
| AL355075.5 | 1.826824 | 12.66934 | 3.657869 | 0.000586 | 0.004151 | -0.73808 |
| ASIC1 | 2.125593 | 12.62517 | 3.65736 | 0.000587 | 0.004154 | -0.73957 |
| CCDC184 | 1.315186 | 13.09024 | 3.656948 | 0.000588 | 0.004158 | -0.74077 |
| NYAP1 | 1.390214 | 13.02585 | 3.65597 | 0.00059 | 0.004169 | -0.74363 |
| AANAT | -1.34181 | 13.25263 | -3.65587 | 0.00059 | 0.004169 | -0.74391 |
| TBC1D8 | -1.03658 | 13.23799 | -3.65525 | 0.000591 | 0.004175 | -0.74573 |
| CDHR4 | -1.51849 | 13.32008 | -3.65237 | 0.000596 | 0.004205 | -0.75415 |
| PAX3 | -2.27129 | 12.59596 | -3.65023 | 0.0006 | 0.004229 | -0.76039 |
| SCD | -1.00911 | 12.98873 | -3.64927 | 0.000602 | 0.004239 | -0.76318 |
| CLDN18 | -1.64574 | 12.781 | -3.64728 | 0.000606 | 0.004258 | -0.76899 |
| AC136428.1 | -2.35597 | 12.5753 | -3.64718 | 0.000606 | 0.004259 | -0.76929 |
| MYL4 | 1.973556 | 11.60441 | 3.646933 | 0.000606 | 0.00426 | -0.77001 |
| RPL21P18 | 1.299701 | 13.70571 | 3.646584 | 0.000607 | 0.004263 | -0.77102 |
| COX6B2 | -1.57873 | 13.0518 | -3.6464 | 0.000607 | 0.004265 | -0.77156 |
| EBAG9P1 | -1.17892 | 13.12926 | -3.64492 | 0.00061 | 0.00428 | -0.77587 |
| GZMM | 1.200158 | 12.98624 | 3.644021 | 0.000612 | 0.00429 | -0.7785 |
| RPL12P41 | 1.055878 | 14.32298 | 3.64369 | 0.000613 | 0.004293 | -0.77946 |
| FBXO32 | 1.118591 | 13.27891 | 3.6424 | 0.000615 | 0.004306 | -0.78322 |
| HLA-DQB1 | 1.313912 | 12.93591 | 3.64191 | 0.000616 | 0.004311 | -0.78465 |
| GATA2 | 1.405357 | 12.709 | 3.638265 | 0.000623 | 0.004353 | -0.79527 |
| MINDY4B | -1.55983 | 13.44945 | -3.63638 | 0.000627 | 0.004376 | -0.80077 |
| RPS27AP6 | -1.52893 | 13.19137 | -3.63604 | 0.000627 | 0.00438 | -0.80175 |
| OR10G2 | 1.782398 | 12.85295 | 3.635296 | 0.000629 | 0.004389 | -0.80392 |
| BTBD6P1 | 1.380647 | 13.178 | 3.633529 | 0.000632 | 0.004409 | -0.80906 |
| TREML1 | -1.51912 | 12.84318 | -3.63223 | 0.000635 | 0.004421 | -0.81284 |
| BMP4 | 1.530977 | 13.25664 | 3.63189 | 0.000635 | 0.004424 | -0.81383 |
| AC097374.1 | 1.438326 | 13.05601 | 3.630178 | 0.000639 | 0.004446 | -0.81881 |
| SPARC | -1.21241 | 13.03972 | -3.6291 | 0.000641 | 0.004459 | -0.82196 |
| CD46P1 | -1.52098 | 13.17828 | -3.62852 | 0.000642 | 0.004465 | -0.82364 |
| S1PR5 | 1.387094 | 13.06138 | 3.628162 | 0.000643 | 0.004469 | -0.82467 |
| BBIP1P1 | -1.66072 | 13.3231 | -3.62706 | 0.000645 | 0.00448 | -0.82789 |
| ZFP2 | 1.494174 | 13.14551 | 3.626687 | 0.000646 | 0.004484 | -0.82896 |
| AC139491.7 | 2.565841 | 12.30818 | 3.626166 | 0.000647 | 0.00449 | -0.83048 |
| H1-5 | -1.52212 | 13.06255 | -3.62558 | 0.000648 | 0.004497 | -0.83218 |
| TRAJ20 | 1.874423 | 13.00373 | 3.625409 | 0.000648 | 0.004498 | -0.83268 |
| SLC6A9 | -1.59681 | 11.03651 | -3.62517 | 0.000649 | 0.004499 | -0.83337 |
| TRGV2 | 1.475556 | 12.93573 | 3.624988 | 0.000649 | 0.004501 | -0.8339 |
| LEP | -1.05543 | 13.29212 | -3.62356 | 0.000652 | 0.004517 | -0.83804 |
| OTOF | -2.94793 | 11.91906 | -3.62305 | 0.000653 | 0.004523 | -0.83952 |
| NR5A2 | -1.11108 | 13.28048 | -3.62256 | 0.000654 | 0.004527 | -0.84097 |
| SPEGNB | -1.2742 | 13.35572 | -3.62213 | 0.000655 | 0.004531 | -0.84221 |
| MTCO2P33 | -1.74681 | 12.89334 | -3.62152 | 0.000656 | 0.004537 | -0.84399 |
| OSM | -1.17978 | 13.24111 | -3.62142 | 0.000656 | 0.004537 | -0.84428 |
| AC099535.1 | 1.066505 | 14.16272 | 3.620231 | 0.000659 | 0.004552 | -0.84772 |
| USP32P3 | -1.08045 | 13.23563 | -3.61962 | 0.00066 | 0.00456 | -0.8495 |
| IRF4 | -1.14285 | 13.28209 | -3.61951 | 0.00066 | 0.00456 | -0.84983 |
| VWCE | 1.972017 | 11.78067 | 3.618859 | 0.000662 | 0.004568 | -0.85171 |
| SDR42E1 | 1.060199 | 13.29537 | 3.61825 | 0.000663 | 0.004575 | -0.85347 |
| ADAD2 | 1.395326 | 13.20014 | 3.615286 | 0.000669 | 0.004613 | -0.86208 |
| SPSB4 | -2.0788 | 12.41906 | -3.6148 | 0.00067 | 0.004615 | -0.86348 |
| TESC | 1.335986 | 12.09661 | 3.614694 | 0.00067 | 0.004615 | -0.86379 |
| RPS2P35 | 1.22744 | 13.86515 | 3.614675 | 0.00067 | 0.004615 | -0.86385 |
| LZTS1 | 1.19573 | 13.09435 | 3.611946 | 0.000676 | 0.004652 | -0.87176 |
| TPSD1 | 1.525503 | 12.62324 | 3.611655 | 0.000676 | 0.004655 | -0.87261 |
| PSMD10P1 | 1.495386 | 13.04026 | 3.611184 | 0.000677 | 0.004659 | -0.87397 |
| ZC2HC1A | 1.06999 | 13.26891 | 3.610958 | 0.000678 | 0.004661 | -0.87463 |
| AC022384.1 | -3.40492 | 12.27425 | -3.60974 | 0.00068 | 0.004676 | -0.87815 |
| AC008753.1 | -1.42222 | 13.16843 | -3.60904 | 0.000682 | 0.004685 | -0.8802 |
| SLC17A1 | 1.132298 | 13.90575 | 3.607756 | 0.000685 | 0.004703 | -0.88391 |
| TAS2R38 | -1.71531 | 13.20402 | -3.60689 | 0.000686 | 0.004713 | -0.88643 |
| BET1P1 | 1.743254 | 12.92428 | 3.606525 | 0.000687 | 0.004717 | -0.88748 |
| NPBWR1 | 1.080011 | 13.51197 | 3.604377 | 0.000692 | 0.004738 | -0.8937 |
| AC087632.2 | -1.93309 | 12.95338 | -3.60387 | 0.000693 | 0.004744 | -0.89516 |
| RPSAP45 | 1.390378 | 13.41828 | 3.601668 | 0.000698 | 0.004776 | -0.90155 |
| ANKRD2 | -1.91457 | 12.99879 | -3.59971 | 0.000702 | 0.004798 | -0.90721 |
| PTGDR | 1.211829 | 13.16105 | 3.599323 | 0.000703 | 0.004799 | -0.90834 |
| BASP1 | -1.15671 | 13.14604 | -3.59896 | 0.000703 | 0.004802 | -0.9094 |
| NTRK2 | 1.908355 | 12.96062 | 3.597839 | 0.000706 | 0.004814 | -0.91263 |
| SH3PXD2B | -1.65981 | 12.78322 | -3.5967 | 0.000708 | 0.004827 | -0.91593 |
| IGHV3-7 | -2.26489 | 12.32741 | -3.59517 | 0.000712 | 0.004843 | -0.92035 |
| RPL17P50 | 1.146183 | 13.21225 | 3.593641 | 0.000715 | 0.004859 | -0.92477 |
| FFAR2 | -1.0346 | 13.4051 | -3.59007 | 0.000723 | 0.004905 | -0.9351 |
| GNLY | 1.323618 | 13.00455 | 3.589674 | 0.000724 | 0.004906 | -0.93624 |
| AP3B2 | -1.85219 | 12.16993 | -3.58954 | 0.000724 | 0.004906 | -0.93664 |
| MPL | -1.49452 | 12.95377 | -3.58949 | 0.000724 | 0.004906 | -0.93677 |
| LY75-CD302 | -3.42553 | 11.41982 | -3.58849 | 0.000726 | 0.004917 | -0.93967 |
| SLC45A3 | 1.090404 | 12.8463 | 3.588468 | 0.000727 | 0.004917 | -0.93972 |
| SIGLEC5 | -2.79506 | 12.6034 | -3.58763 | 0.000728 | 0.004928 | -0.94215 |
| KLRF2 | 1.339825 | 13.8101 | 3.58724 | 0.000729 | 0.004932 | -0.94327 |
| ATP5MC1P4 | -1.20983 | 13.31677 | -3.58576 | 0.000733 | 0.004954 | -0.94756 |
| SH2D1B | 1.553841 | 13.04932 | 3.584007 | 0.000737 | 0.004977 | -0.9526 |
| TRAJ10 | 1.654193 | 13.10453 | 3.583284 | 0.000738 | 0.004984 | -0.95469 |
| RPL23AP55 | 1.02166 | 14.03423 | 3.583145 | 0.000739 | 0.004984 | -0.95509 |
| C5orf67 | -2.16262 | 12.72588 | -3.58265 | 0.00074 | 0.00499 | -0.95651 |
| AC008074.1 | -1.71958 | 13.1868 | -3.58203 | 0.000741 | 0.004998 | -0.95832 |
| TRAJ41 | 1.643052 | 13.09335 | 3.581435 | 0.000742 | 0.005006 | -0.96003 |
| CARNS1 | 1.135999 | 13.09056 | 3.580572 | 0.000744 | 0.005018 | -0.96252 |
| OSMR | -1.0427 | 13.16152 | -3.58029 | 0.000745 | 0.005021 | -0.96334 |
| H2AC14 | -1.73432 | 13.13878 | -3.5798 | 0.000746 | 0.005028 | -0.96475 |
| TERF1P7 | 1.083038 | 13.10502 | 3.578095 | 0.00075 | 0.005048 | -0.96966 |
| RPL35P5 | 1.23356 | 13.24931 | 3.575553 | 0.000756 | 0.005078 | -0.97699 |
| AC100756.2 | -2.00214 | 12.9507 | -3.57441 | 0.000759 | 0.005091 | -0.98028 |
| FA2H | -1.41774 | 13.15273 | -3.57403 | 0.00076 | 0.005094 | -0.98137 |
| FCRL3 | 1.333317 | 13.05604 | 3.571246 | 0.000766 | 0.005135 | -0.9894 |
| GLIS2 | -1.21747 | 13.16747 | -3.57077 | 0.000767 | 0.00514 | -0.99076 |
| HNRNPA1P21 | 1.389838 | 13.01538 | 3.568541 | 0.000772 | 0.00517 | -0.99719 |
| PVRIG | 1.022052 | 13.23027 | 3.568187 | 0.000773 | 0.005174 | -0.99821 |
| IFI6 | -1.84193 | 12.95131 | -3.56765 | 0.000775 | 0.005181 | -0.99975 |
| AC046144.1 | -1.27805 | 13.3174 | -3.56693 | 0.000776 | 0.005191 | -1.00183 |
| AC080023.2 | -2.29294 | 12.56952 | -3.56686 | 0.000776 | 0.005191 | -1.00202 |
| RPL12P12 | 1.862185 | 12.92306 | 3.566328 | 0.000778 | 0.005197 | -1.00356 |
| DNAI2 | 1.700451 | 12.86346 | 3.565909 | 0.000779 | 0.005202 | -1.00477 |
| AMPH | -2.65641 | 11.92586 | -3.56501 | 0.000781 | 0.005213 | -1.00737 |
| HMGB3P32 | 1.584611 | 13.03135 | 3.564795 | 0.000781 | 0.005214 | -1.00797 |
| PLCH2 | 1.024421 | 13.01215 | 3.561757 | 0.000789 | 0.005257 | -1.01671 |
| IGHV3-52 | -2.18872 | 12.27964 | -3.55986 | 0.000793 | 0.005283 | -1.02218 |
| SH2D2A | 1.03661 | 13.14466 | 3.559058 | 0.000795 | 0.005294 | -1.02447 |
| AC005726.1 | -1.97326 | 12.78132 | -3.55828 | 0.000797 | 0.005306 | -1.02671 |
| GTF2IP9 | -1.36301 | 13.26965 | -3.55795 | 0.000798 | 0.00531 | -1.02765 |
| BTBD19 | -1.37696 | 13.13495 | -3.55563 | 0.000804 | 0.005341 | -1.03432 |
| SDR42E1P5 | -2.44969 | 12.22615 | -3.5553 | 0.000804 | 0.005344 | -1.03528 |
| KLRF1 | 1.522523 | 13.11243 | 3.554431 | 0.000807 | 0.005354 | -1.03777 |
| BFSP2 | -1.32427 | 13.24895 | -3.55425 | 0.000807 | 0.005355 | -1.03829 |
| PTCH1 | 1.111052 | 13.22184 | 3.552988 | 0.00081 | 0.005374 | -1.04191 |
| TNNC1 | 1.33495 | 13.11662 | 3.550867 | 0.000815 | 0.005403 | -1.048 |
| HMGN1P3 | -1.70646 | 12.75092 | -3.54992 | 0.000818 | 0.005416 | -1.05072 |
| TRBV5-3 | 1.48089 | 13.47686 | 3.549238 | 0.00082 | 0.005425 | -1.05268 |
| TRBV12-4 | 1.819572 | 12.79523 | 3.548588 | 0.000821 | 0.005433 | -1.05454 |
| TRBJ1-6 | 1.346798 | 12.91903 | 3.547764 | 0.000823 | 0.005446 | -1.05691 |
| RPSAP3 | 1.432043 | 13.2666 | 3.547263 | 0.000825 | 0.00545 | -1.05835 |
| SBK1 | 1.169468 | 13.15016 | 3.547239 | 0.000825 | 0.00545 | -1.05841 |
| TRAJ50 | 1.536884 | 13.1479 | 3.545472 | 0.000829 | 0.00547 | -1.06348 |
| KLRC1 | 1.107533 | 13.19544 | 3.545423 | 0.000829 | 0.00547 | -1.06362 |
| RPL9P32 | 1.21183 | 13.76305 | 3.545103 | 0.00083 | 0.005474 | -1.06454 |
| IL18R1 | -1.89196 | 12.7687 | -3.54442 | 0.000832 | 0.005483 | -1.0665 |
| TCN1 | -2.02292 | 11.78571 | -3.54353 | 0.000834 | 0.005497 | -1.06905 |
| DLL4 | -1.55294 | 13.15646 | -3.54329 | 0.000835 | 0.005499 | -1.06974 |
| SETP5 | -1.22631 | 13.44441 | -3.543 | 0.000835 | 0.005503 | -1.07057 |
| RLN1 | 1.422831 | 13.09938 | 3.542662 | 0.000836 | 0.005508 | -1.07154 |
| TMPRSS11E | 2.07586 | 12.20506 | 3.5425 | 0.000837 | 0.005509 | -1.072 |
| RAB43 | -1.24694 | 13.08925 | -3.54132 | 0.00084 | 0.005526 | -1.0754 |
| EEF1A1P7 | 1.108084 | 13.54306 | 3.541267 | 0.00084 | 0.005526 | -1.07554 |
| TRIM64B | 2.381163 | 11.7256 | 3.539088 | 0.000845 | 0.005555 | -1.08178 |
| KIAA1210 | -1.09052 | 13.22457 | -3.53907 | 0.000845 | 0.005555 | -1.08183 |
| KRTAP5-11 | 1.448583 | 13.19863 | 3.537152 | 0.00085 | 0.005582 | -1.08732 |
| AL133260.1 | 1.446531 | 13.17726 | 3.535587 | 0.000855 | 0.005596 | -1.09181 |
| MUC6 | -1.49086 | 13.5975 | -3.53243 | 0.000863 | 0.005641 | -1.10085 |
| DKK2 | 1.755032 | 12.95192 | 3.532061 | 0.000864 | 0.005646 | -1.1019 |
| MACIR | -1.68213 | 12.689 | -3.53161 | 0.000865 | 0.005651 | -1.10319 |
| ASAH2 | 1.049341 | 13.29591 | 3.530564 | 0.000868 | 0.005666 | -1.10618 |
| H3C13 | -1.8575 | 12.70188 | -3.53034 | 0.000868 | 0.005668 | -1.10681 |
| RPL24P2 | 1.354669 | 13.2769 | 3.528457 | 0.000873 | 0.005697 | -1.1122 |
| ZNF112 | 1.262958 | 13.14811 | 3.528225 | 0.000874 | 0.005699 | -1.11287 |
| AC012435.2 | 1.190061 | 12.55536 | 3.52703 | 0.000877 | 0.005715 | -1.11629 |
| PTPRK | 1.392201 | 13.23511 | 3.525448 | 0.000881 | 0.005732 | -1.12081 |
| SFRP5 | 2.191886 | 12.51992 | 3.525379 | 0.000882 | 0.005732 | -1.12101 |
| CSF1R | 1.047626 | 13.02593 | 3.52413 | 0.000885 | 0.005752 | -1.12457 |
| LDOC1 | 1.216055 | 13.10487 | 3.52166 | 0.000892 | 0.005789 | -1.13163 |
| ELANE | -3.05229 | 10.59858 | -3.52076 | 0.000894 | 0.005803 | -1.13421 |
| ANGPT1 | 1.317372 | 12.95239 | 3.519693 | 0.000897 | 0.005816 | -1.13725 |
| AK8 | 1.374483 | 13.15575 | 3.518403 | 0.000901 | 0.005833 | -1.14093 |
| AC233282.1 | 1.899188 | 12.60008 | 3.518387 | 0.000901 | 0.005833 | -1.14098 |
| AC005840.1 | -1.48771 | 13.55914 | -3.51356 | 0.000914 | 0.005911 | -1.15474 |
| AL049757.2 | -1.33342 | 13.13256 | -3.51313 | 0.000915 | 0.005916 | -1.15596 |
| CICP27 | -1.05846 | 13.20433 | -3.51302 | 0.000915 | 0.005917 | -1.15627 |
| FOXD4L4 | -1.43217 | 13.40631 | -3.51239 | 0.000917 | 0.005923 | -1.15809 |
| AL669918.1 | -4.34988 | 10.08523 | -3.5113 | 0.00092 | 0.005935 | -1.16118 |
| TP53AIP1 | -1.26853 | 13.33454 | -3.51122 | 0.00092 | 0.005935 | -1.16142 |
| HBE1 | -2.78193 | 10.93091 | -3.51086 | 0.000921 | 0.005939 | -1.16244 |
| IRF2BPL | -1.036 | 13.2703 | -3.50975 | 0.000925 | 0.005956 | -1.16562 |
| TRIM51FP | 1.048068 | 13.55485 | 3.508975 | 0.000927 | 0.005965 | -1.16782 |
| AC093627.15 | -1.1441 | 13.31412 | -3.50875 | 0.000927 | 0.005966 | -1.16845 |
| AC215522.1 | -1.1441 | 13.31412 | -3.50875 | 0.000927 | 0.005966 | -1.16845 |
| FKBP5 | -1.36868 | 13.08037 | -3.50798 | 0.00093 | 0.005976 | -1.17065 |
| PRTN3 | -3.11903 | 10.66182 | -3.50792 | 0.00093 | 0.005976 | -1.17083 |
| TRAJ22 | 1.689378 | 12.94283 | 3.506741 | 0.000933 | 0.005995 | -1.17418 |
| TRAV25 | 1.78072 | 12.89425 | 3.506204 | 0.000935 | 0.006002 | -1.17571 |
| TJP3 | 1.214757 | 13.12221 | 3.506078 | 0.000935 | 0.006003 | -1.17607 |
| INHBA | -2.46633 | 11.40342 | -3.50598 | 0.000935 | 0.006004 | -1.17634 |
| DUX4L9 | -1.41334 | 13.27957 | -3.504 | 0.000941 | 0.006034 | -1.18198 |
| PDZK1IP1 | 1.713985 | 11.34617 | 3.500845 | 0.00095 | 0.006078 | -1.19097 |
| TRAJ24 | 1.844781 | 12.91372 | 3.496731 | 0.000962 | 0.006143 | -1.20267 |
| HNRNPH1P1 | -1.38046 | 13.3495 | -3.49524 | 0.000966 | 0.006165 | -1.2069 |
| AOC1 | -3.25444 | 11.55138 | -3.49468 | 0.000968 | 0.006174 | -1.2085 |
| LY6E | -1.58795 | 13.17808 | -3.49392 | 0.00097 | 0.006188 | -1.21067 |
| BANK1 | 1.000653 | 13.30396 | 3.493395 | 0.000972 | 0.006196 | -1.21216 |
| FBXO43 | -1.03017 | 12.87927 | -3.49207 | 0.000976 | 0.006217 | -1.21593 |
| SLC16A11 | 1.246175 | 13.16725 | 3.488083 | 0.000988 | 0.006282 | -1.22725 |
| VDAC2P2 | 1.603997 | 13.11062 | 3.486967 | 0.000991 | 0.006301 | -1.23041 |
| VIT | 1.423246 | 12.91494 | 3.486781 | 0.000991 | 0.006303 | -1.23094 |
| DLG3 | 1.029952 | 13.26066 | 3.485847 | 0.000994 | 0.00632 | -1.23359 |
| CCL5 | 1.056456 | 13.04676 | 3.485667 | 0.000995 | 0.006322 | -1.2341 |
| RTKN2 | 1.247123 | 13.13832 | 3.484618 | 0.000998 | 0.006336 | -1.23708 |
| RPSAP5 | 1.349076 | 13.5909 | 3.48453 | 0.000998 | 0.006336 | -1.23733 |
| PPIAL4F | 1.007214 | 14.15751 | 3.483933 | 0.001 | 0.006339 | -1.23902 |
| DACH1 | -1.28038 | 12.96199 | -3.48342 | 0.001002 | 0.006342 | -1.24047 |
| NUDT4B | -1.0959 | 12.62424 | -3.48121 | 0.001008 | 0.006373 | -1.24674 |
| NUDT4P2 | -1.0959 | 12.62423 | -3.48121 | 0.001008 | 0.006373 | -1.24674 |
| TEX11 | -1.52618 | 13.01851 | -3.48005 | 0.001012 | 0.006387 | -1.25003 |
| TRAJ1 | 1.683337 | 12.84893 | 3.479121 | 0.001015 | 0.006402 | -1.25267 |
| AC022210.1 | 1.534109 | 13.22731 | 3.477044 | 0.001021 | 0.006436 | -1.25856 |
| OLFML1 | -1.091 | 13.2245 | -3.47412 | 0.00103 | 0.006483 | -1.26683 |
| RPL7P9 | 1.738109 | 13.03794 | 3.471701 | 0.001038 | 0.006521 | -1.27369 |
| AC005531.1 | -1.0606 | 13.22887 | -3.47067 | 0.001041 | 0.006537 | -1.27662 |
| ZNF488 | 1.182491 | 13.79222 | 3.469875 | 0.001044 | 0.006548 | -1.27886 |
| MARK2P16 | 1.429471 | 13.00578 | 3.469056 | 0.001046 | 0.006557 | -1.28118 |
| GPR150 | 1.413947 | 13.03694 | 3.467094 | 0.001052 | 0.006589 | -1.28673 |
| AC069368.1 | -3.57175 | 11.64132 | -3.46545 | 0.001058 | 0.006621 | -1.29137 |
| RPS10P2 | 1.084402 | 14.2221 | 3.465289 | 0.001058 | 0.006623 | -1.29183 |
| RAB25 | 1.497001 | 13.02251 | 3.464824 | 0.00106 | 0.006629 | -1.29314 |
| SVOPL | 1.6588 | 12.87718 | 3.464001 | 0.001062 | 0.006642 | -1.29547 |
| RFPL2 | 1.429548 | 12.51016 | 3.463621 | 0.001064 | 0.006647 | -1.29655 |
| IGKV1OR2-108 | -1.90166 | 12.3002 | -3.46217 | 0.001068 | 0.00667 | -1.30064 |
| RTBDN | -1.04969 | 13.31495 | -3.46191 | 0.001069 | 0.006674 | -1.30139 |
| CES1 | -1.37024 | 13.14002 | -3.46019 | 0.001075 | 0.006702 | -1.30623 |
| AC117383.2 | -1.46416 | 13.35235 | -3.45986 | 0.001076 | 0.006707 | -1.30719 |
| KLF14 | -2.49386 | 11.89706 | -3.45951 | 0.001077 | 0.006711 | -1.30816 |
| SEMA3F | -1.72436 | 12.82512 | -3.45949 | 0.001077 | 0.006711 | -1.30821 |
| UBXN10 | 1.046296 | 12.71756 | 3.459374 | 0.001077 | 0.006712 | -1.30855 |
| MT1M | -1.32852 | 13.32174 | -3.45832 | 0.001081 | 0.00673 | -1.31153 |
| GRAMD1C | 1.118818 | 13.15993 | 3.457601 | 0.001083 | 0.006739 | -1.31355 |
| LRRC36 | 1.112889 | 13.19898 | 3.457336 | 0.001084 | 0.006743 | -1.3143 |
| CNN1 | -1.18868 | 12.93018 | -3.45599 | 0.001088 | 0.006762 | -1.31809 |
| IGKV1OR22-1 | -1.98867 | 12.75041 | -3.45271 | 0.001099 | 0.006825 | -1.32735 |
| IGFBP3 | 1.519895 | 12.81047 | 3.45124 | 0.001104 | 0.006849 | -1.33151 |
| PCK1 | -1.56485 | 12.89093 | -3.45059 | 0.001106 | 0.00686 | -1.33334 |
| SLC22A14 | -1.55276 | 12.78953 | -3.45013 | 0.001108 | 0.006868 | -1.33464 |
| SLC16A2 | 1.440734 | 13.23456 | 3.44989 | 0.001109 | 0.006871 | -1.33531 |
| SLC51A | -1.20211 | 13.01593 | -3.449 | 0.001112 | 0.006884 | -1.33782 |
| EBF4 | 1.659312 | 12.90036 | 3.448853 | 0.001112 | 0.006885 | -1.33824 |
| AC008443.7 | -1.58151 | 12.9536 | -3.44884 | 0.001112 | 0.006885 | -1.33829 |
| RPS2P55 | 1.377668 | 13.15473 | 3.448482 | 0.001113 | 0.006891 | -1.33928 |
| BNIP3P26 | -1.94445 | 12.96959 | -3.44765 | 0.001116 | 0.0069 | -1.34162 |
| IGKV2-29 | -2.64855 | 10.93841 | -3.44595 | 0.001122 | 0.006926 | -1.34643 |
| NEFLP1 | 1.406241 | 13.52259 | 3.44509 | 0.001125 | 0.006936 | -1.34884 |
| GPR4 | -1.18703 | 13.08124 | -3.44495 | 0.001125 | 0.006937 | -1.34924 |
| XKRX | 1.18068 | 13.23637 | 3.444706 | 0.001126 | 0.006941 | -1.34992 |
| TRBV10-1 | 1.745345 | 12.86732 | 3.444033 | 0.001128 | 0.006951 | -1.35182 |
| COLGALT2 | 1.288858 | 12.86095 | 3.442019 | 0.001135 | 0.006989 | -1.35749 |
| TRAJ9 | 1.559692 | 13.09468 | 3.440301 | 0.001141 | 0.007022 | -1.36233 |
| CPLX4 | -1.40546 | 13.201 | -3.43897 | 0.001146 | 0.007042 | -1.36608 |
| CPA3 | 2.077244 | 12.39498 | 3.438351 | 0.001148 | 0.007053 | -1.36782 |
| ACTG1P22 | -1.74502 | 13.16403 | -3.43814 | 0.001149 | 0.007055 | -1.36842 |
| TTC24 | 1.406684 | 12.99713 | 3.437413 | 0.001151 | 0.007068 | -1.37046 |
| KLRC4-KLRK1 | 1.661048 | 12.85924 | 3.4371 | 0.001152 | 0.007073 | -1.37134 |
| UPK3A | 1.695403 | 12.86411 | 3.436605 | 0.001154 | 0.007082 | -1.37273 |
| FOXJ1 | 1.333926 | 13.12602 | 3.436168 | 0.001155 | 0.00709 | -1.37396 |
| AC092658.1 | -1.53146 | 13.2478 | -3.43534 | 0.001158 | 0.007106 | -1.37629 |
| CAPN5 | 1.118459 | 12.04114 | 3.433055 | 0.001166 | 0.007147 | -1.38271 |
| DCDC2B | -1.03525 | 13.28552 | -3.43238 | 0.001169 | 0.00716 | -1.38461 |
| TRAV35 | 1.195425 | 13.1284 | 3.430142 | 0.001177 | 0.007198 | -1.3909 |
| HTR2A | -1.34531 | 13.02018 | -3.43012 | 0.001177 | 0.007198 | -1.39096 |
| YBX1P2 | 1.37707 | 13.18728 | 3.429215 | 0.00118 | 0.007216 | -1.3935 |
| LRIG3 | -1.57411 | 13.32874 | -3.42918 | 0.00118 | 0.007216 | -1.39361 |
| DEPTOR | 1.039546 | 13.21181 | 3.428987 | 0.001181 | 0.007217 | -1.39414 |
| UPK1B | -1.52411 | 13.20966 | -3.4287 | 0.001182 | 0.007222 | -1.39494 |
| PGLYRP1 | -2.13199 | 11.80992 | -3.42849 | 0.001182 | 0.007224 | -1.39555 |
| IGHV1-68 | -2.80813 | 9.569721 | -3.42846 | 0.001183 | 0.007224 | -1.39563 |
| AL138709.1 | -1.07489 | 13.30329 | -3.42738 | 0.001186 | 0.007242 | -1.39866 |
| MEP1A | 1.461628 | 13.31526 | 3.427176 | 0.001187 | 0.007245 | -1.39923 |
| AC107032.1 | 1.608881 | 13.10682 | 3.425609 | 0.001193 | 0.007275 | -1.40363 |
| BCL2 | 1.119714 | 13.23418 | 3.425608 | 0.001193 | 0.007275 | -1.40363 |
| KRT18P39 | 1.537919 | 13.05376 | 3.425463 | 0.001193 | 0.007275 | -1.40404 |
| AC002996.1 | -1.97561 | 12.59335 | -3.42522 | 0.001194 | 0.007277 | -1.40472 |
| KLF17P1 | -1.51178 | 13.3179 | -3.42442 | 0.001197 | 0.007293 | -1.40695 |
| PDSS1P1 | -1.32615 | 13.40727 | -3.42321 | 0.001201 | 0.007314 | -1.41035 |
| RARB | 1.022582 | 13.22965 | 3.423144 | 0.001202 | 0.007314 | -1.41055 |
| JAGN1 | -1.44589 | 13.03588 | -3.4224 | 0.001204 | 0.007324 | -1.41265 |
| EDN3 | 1.606379 | 13.02501 | 3.421457 | 0.001208 | 0.007341 | -1.41528 |
| TRBV7-6 | 1.458493 | 12.89939 | 3.421046 | 0.001209 | 0.007344 | -1.41643 |
| BOLA2 | 1.139372 | 13.26559 | 3.420448 | 0.001211 | 0.007356 | -1.41811 |
| KAZALD1 | -1.30017 | 13.01104 | -3.41917 | 0.001216 | 0.007381 | -1.42169 |
| FGFBP2 | 1.362645 | 12.95797 | 3.418971 | 0.001217 | 0.007384 | -1.42225 |
| RPL10P19 | 1.296962 | 12.85735 | 3.418797 | 0.001217 | 0.007386 | -1.42274 |
| ZCCHC2 | -1.03651 | 13.37303 | -3.41699 | 0.001224 | 0.007424 | -1.42781 |
| MMP19 | -1.14702 | 13.09143 | -3.41687 | 0.001224 | 0.007425 | -1.42815 |
| HPGDS | 1.421617 | 12.80746 | 3.416795 | 0.001225 | 0.007425 | -1.42835 |
| AC135983.5 | -2.28114 | 12.37459 | -3.41377 | 0.001236 | 0.007479 | -1.43683 |
| CREB3L3 | -1.69349 | 12.63689 | -3.41351 | 0.001237 | 0.00748 | -1.43755 |
| MYL12BP1 | 1.126347 | 13.11445 | 3.409206 | 0.001253 | 0.007572 | -1.44961 |
| TRIM49D1 | 1.826059 | 11.96131 | 3.408689 | 0.001255 | 0.00758 | -1.45105 |
| RPSAP53 | 1.379111 | 13.25643 | 3.408557 | 0.001255 | 0.007582 | -1.45142 |
| DDR2 | -1.24054 | 13.26342 | -3.40757 | 0.001259 | 0.007602 | -1.4542 |
| LDLRAD1 | -1.16958 | 13.25144 | -3.40613 | 0.001265 | 0.007633 | -1.45822 |
| PDE9A | 1.715022 | 12.99467 | 3.405583 | 0.001267 | 0.007644 | -1.45974 |
| IGFBP2 | -1.83785 | 12.40826 | -3.40498 | 0.001269 | 0.007654 | -1.46142 |
| PDE7B | 1.034053 | 13.12756 | 3.404896 | 0.001269 | 0.007655 | -1.46166 |
| MTRNR2L7 | 1.263085 | 13.48066 | 3.403573 | 0.001274 | 0.007679 | -1.46536 |
| AL450326.2 | 1.32654 | 13.08372 | 3.403562 | 0.001274 | 0.007679 | -1.4654 |
| OR7E14P | 1.811987 | 12.67306 | 3.403 | 0.001277 | 0.007686 | -1.46697 |
| GATM | 1.134658 | 13.21679 | 3.402611 | 0.001278 | 0.007689 | -1.46805 |
| CNKSR2 | 1.005883 | 13.30335 | 3.400737 | 0.001285 | 0.007726 | -1.47329 |
| DPYSL5 | -1.13842 | 13.10829 | -3.40055 | 0.001286 | 0.007729 | -1.47381 |
| AL512662.2 | -1.40764 | 13.34057 | -3.3995 | 0.00129 | 0.007749 | -1.47675 |
| GPR82 | 1.012054 | 13.18321 | 3.39711 | 0.001299 | 0.007795 | -1.48342 |
| CACNA2D2 | 1.228213 | 13.13072 | 3.395954 | 0.001304 | 0.007814 | -1.48665 |
| IGHV3-47 | -1.92543 | 12.50075 | -3.39562 | 0.001305 | 0.007821 | -1.48759 |
| RPS27P21 | 1.447379 | 13.22413 | 3.394964 | 0.001308 | 0.007833 | -1.48941 |
| CTTN | -1.06671 | 13.11013 | -3.39362 | 0.001313 | 0.007861 | -1.49317 |
| STC2 | -1.54783 | 13.42458 | -3.39313 | 0.001315 | 0.007868 | -1.49453 |
| RAI14 | 1.185192 | 13.18759 | 3.39094 | 0.001323 | 0.007909 | -1.50064 |
| AC104986.1 | -1.31753 | 13.23037 | -3.39076 | 0.001324 | 0.00791 | -1.50115 |
| ATP5MGL | -1.15174 | 13.23209 | -3.38952 | 0.001329 | 0.007933 | -1.50461 |
| C10orf105 | -1.3769 | 13.23613 | -3.38928 | 0.00133 | 0.007937 | -1.50527 |
| PTMAP11 | -1.18598 | 13.43688 | -3.38859 | 0.001333 | 0.007949 | -1.5072 |
| ZNF286B | 2.091569 | 12.8021 | 3.387797 | 0.001336 | 0.007963 | -1.50941 |
| OCA2 | 1.319199 | 13.63052 | 3.387155 | 0.001339 | 0.007974 | -1.5112 |
| ALDH1A3 | -1.21101 | 13.09093 | -3.38525 | 0.001346 | 0.008014 | -1.51651 |
| OLFML2A | -1.42049 | 13.06087 | -3.38522 | 0.001346 | 0.008014 | -1.51659 |
| CHORDC1P4 | -1.40844 | 13.05647 | -3.38352 | 0.001353 | 0.008046 | -1.52131 |
| ALDH7A1 | -1.08922 | 13.30474 | -3.38079 | 0.001364 | 0.0081 | -1.52893 |
| ZNF214 | -1.60295 | 13.05495 | -3.38051 | 0.001365 | 0.008105 | -1.52972 |
| C1orf21 | 1.038181 | 13.22581 | 3.379227 | 0.001371 | 0.008133 | -1.53328 |
| TLR9 | -1.0191 | 13.25435 | -3.37522 | 0.001387 | 0.008219 | -1.54442 |
| ADAT3 | -1.06154 | 13.06859 | -3.37417 | 0.001391 | 0.008237 | -1.54735 |
| AL139095.2 | 1.274372 | 13.55216 | 3.373092 | 0.001396 | 0.00826 | -1.55034 |
| MTCO1P30 | 1.277302 | 13.48071 | 3.372238 | 0.0014 | 0.008277 | -1.55272 |
| C2 | -1.33466 | 13.18927 | -3.37053 | 0.001407 | 0.00831 | -1.55747 |
| NFASC | -1.11003 | 13.20359 | -3.37049 | 0.001407 | 0.00831 | -1.55757 |
| TRAJ27 | 1.391232 | 13.16533 | 3.370486 | 0.001407 | 0.00831 | -1.55759 |
| RSPO2 | -1.87121 | 12.19118 | -3.3701 | 0.001408 | 0.008315 | -1.55866 |
| AKAP12 | 1.91003 | 12.44698 | 3.367189 | 0.001421 | 0.008376 | -1.56674 |
| IGKV2-40 | -2.18347 | 11.81188 | -3.36631 | 0.001424 | 0.00839 | -1.5692 |
| KCNJ4 | -1.77968 | 13.05812 | -3.36366 | 0.001436 | 0.00844 | -1.57655 |
| PRSS50 | -2.40838 | 11.77063 | -3.36217 | 0.001442 | 0.008472 | -1.58066 |
| IGHV1-45 | -2.86295 | 10.86801 | -3.36126 | 0.001446 | 0.00849 | -1.5832 |
| GPR162 | 1.203052 | 12.77621 | 3.360716 | 0.001448 | 0.008498 | -1.58471 |
| CALY | 1.436915 | 12.84777 | 3.358775 | 0.001457 | 0.008538 | -1.59009 |
| MID2 | 1.481138 | 13.0818 | 3.358749 | 0.001457 | 0.008538 | -1.59016 |
| RPS15AP27 | 1.362554 | 12.95608 | 3.358441 | 0.001458 | 0.008544 | -1.59101 |
| AC104563.1 | 1.56795 | 13.07041 | 3.357278 | 0.001463 | 0.008568 | -1.59424 |
| AC073111.3 | 1.349703 | 13.11894 | 3.352993 | 0.001482 | 0.008661 | -1.60611 |
| TRBJ2-3 | 1.001864 | 13.09309 | 3.351262 | 0.00149 | 0.008699 | -1.6109 |
| TRIM80P | -1.26383 | 13.60666 | -3.3507 | 0.001492 | 0.008711 | -1.61245 |
| RPSAP4 | 1.461598 | 13.37289 | 3.349766 | 0.001496 | 0.008731 | -1.61504 |
| CBLIF | -1.28267 | 13.14365 | -3.34958 | 0.001497 | 0.008734 | -1.61554 |
| TRAV40 | 1.599577 | 13.01401 | 3.34952 | 0.001497 | 0.008734 | -1.61572 |
| RPL4P3 | 1.26711 | 13.37391 | 3.34863 | 0.001501 | 0.008754 | -1.61819 |
| ANKRD65 | 1.312383 | 13.30143 | 3.348556 | 0.001502 | 0.008754 | -1.61839 |
| TRBV13 | 1.672705 | 12.60405 | 3.347669 | 0.001506 | 0.008772 | -1.62084 |
| PTPRS | 1.081434 | 12.72772 | 3.347595 | 0.001506 | 0.008773 | -1.62105 |
| AL049873.1 | 1.197948 | 13.37456 | 3.347006 | 0.001509 | 0.008783 | -1.62268 |
| FPR3 | 1.599702 | 12.61073 | 3.346443 | 0.001511 | 0.008793 | -1.62423 |
| CD200 | 1.225881 | 13.22573 | 3.345613 | 0.001515 | 0.008808 | -1.62653 |
| GRTP1 | -1.0136 | 13.19368 | -3.34531 | 0.001516 | 0.008814 | -1.62738 |
| FTH1P22 | 1.387598 | 12.8985 | 3.343752 | 0.001523 | 0.00885 | -1.63168 |
| AC106872.2 | 1.158133 | 13.47022 | 3.343322 | 0.001525 | 0.008857 | -1.63286 |
| CNN2P1 | -1.07351 | 12.98877 | -3.34289 | 0.001527 | 0.008864 | -1.63405 |
| OR52I2 | -1.36018 | 13.71178 | -3.34179 | 0.001532 | 0.008886 | -1.63711 |
| JAKMIP2 | 1.048989 | 13.22172 | 3.341631 | 0.001533 | 0.008888 | -1.63754 |
| TRBJ1-5 | 1.320389 | 12.83716 | 3.34077 | 0.001537 | 0.008908 | -1.63992 |
| RPL23AP18 | 1.12738 | 13.61625 | 3.339394 | 0.001543 | 0.008941 | -1.64372 |
| AC074141.1 | 1.445469 | 13.08097 | 3.335452 | 0.001561 | 0.009034 | -1.6546 |
| DNAJC19P5 | -1.04307 | 13.20616 | -3.33534 | 0.001562 | 0.009035 | -1.65491 |
| PRUNE2 | -1.56264 | 12.53468 | -3.33522 | 0.001562 | 0.009036 | -1.65524 |
| Z97206.1 | 1.002034 | 13.84693 | 3.33506 | 0.001563 | 0.009039 | -1.65568 |
| PLEKHG5 | 1.243682 | 13.00218 | 3.333071 | 0.001572 | 0.009082 | -1.66117 |
| CHRM4 | -1.34476 | 13.10489 | -3.33092 | 0.001582 | 0.00913 | -1.66711 |
| AP000925.1 | -1.14762 | 13.22849 | -3.32954 | 0.001589 | 0.009162 | -1.6709 |
| H2AC16 | -1.5173 | 13.05829 | -3.32859 | 0.001593 | 0.009177 | -1.67351 |
| IGLV5-48 | -2.06961 | 11.83407 | -3.32824 | 0.001595 | 0.009182 | -1.6745 |
| SDK2 | 1.151622 | 13.14081 | 3.327596 | 0.001598 | 0.009193 | -1.67627 |
| CST7 | -1.12768 | 13.07714 | -3.32751 | 0.001599 | 0.009193 | -1.67651 |
| ADAMTS7P3 | -1.25366 | 13.37358 | -3.3271 | 0.0016 | 0.009202 | -1.67764 |
| PCSK6 | -1.19668 | 12.90679 | -3.32693 | 0.001601 | 0.009205 | -1.67811 |
| TMEM240 | -1.49173 | 13.05141 | -3.32624 | 0.001605 | 0.009219 | -1.68002 |
| ARL4AP4 | -1.27877 | 13.26911 | -3.32614 | 0.001605 | 0.009219 | -1.68028 |
| RPL21P134 | 1.399255 | 13.20618 | 3.325219 | 0.001609 | 0.009236 | -1.68281 |
| MTCO3P11 | -1.49905 | 13.05584 | -3.32478 | 0.001612 | 0.009244 | -1.68403 |
| RPL21P44 | -1.34526 | 13.0094 | -3.32472 | 0.001612 | 0.009244 | -1.68418 |
| SLPI | -1.68622 | 12.41203 | -3.32424 | 0.001614 | 0.009256 | -1.68552 |
| SEPTIN5 | -1.10055 | 12.7857 | -3.32373 | 0.001617 | 0.009268 | -1.68692 |
| H2BC9 | -1.21712 | 13.22146 | -3.32142 | 0.001628 | 0.009319 | -1.69329 |
| AC011498.4 | -1.46022 | 12.84834 | -3.3209 | 0.00163 | 0.009328 | -1.69471 |
| AC247036.4 | -2.86403 | 11.85836 | -3.32043 | 0.001632 | 0.009336 | -1.696 |
| IGHV3-64D | -2.86403 | 11.85836 | -3.32043 | 0.001632 | 0.009336 | -1.696 |
| CRACR2B | -1.29634 | 12.76397 | -3.32031 | 0.001633 | 0.009337 | -1.69633 |
| AC055811.2 | -1.5959 | 12.89998 | -3.31952 | 0.001637 | 0.00935 | -1.69851 |
| IGLV3-6 | -1.6676 | 13.05488 | -3.31836 | 0.001642 | 0.009379 | -1.70169 |
| RGMB | 1.110305 | 13.23246 | 3.318162 | 0.001643 | 0.009383 | -1.70224 |
| PLCB1 | 1.010533 | 13.30176 | 3.317332 | 0.001647 | 0.009403 | -1.70452 |
| AC000093.1 | -1.20413 | 12.92047 | -3.31432 | 0.001662 | 0.009467 | -1.71281 |
| RPS4XP11 | 1.028581 | 13.97796 | 3.314311 | 0.001662 | 0.009467 | -1.71283 |
| PFN4 | -1.21751 | 13.02712 | -3.31139 | 0.001677 | 0.009543 | -1.72086 |
| RPL9P18 | 1.175847 | 13.63863 | 3.31019 | 0.001683 | 0.009574 | -1.72415 |
| AC004386.1 | -1.31319 | 13.28068 | -3.309 | 0.001688 | 0.009604 | -1.72741 |
| RPL21P3 | 1.222092 | 13.63525 | 3.306927 | 0.001699 | 0.009652 | -1.73311 |
| AC114786.1 | 1.93759 | 12.34124 | 3.306532 | 0.001701 | 0.009661 | -1.73419 |
| MTND4P14 | -1.52147 | 13.03944 | -3.30613 | 0.001703 | 0.009671 | -1.7353 |
| AOC4P | -1.39752 | 12.95262 | -3.30576 | 0.001705 | 0.009678 | -1.73632 |
| AC114737.3 | -1.11445 | 13.24787 | -3.30564 | 0.001705 | 0.009679 | -1.73663 |
| PHC2 | -1.06241 | 13.17652 | -3.30486 | 0.001709 | 0.009693 | -1.73878 |
| AC006517.4 | -1.45178 | 13.49466 | -3.30355 | 0.001716 | 0.009727 | -1.74238 |
| TSPO | -1.00421 | 13.01281 | -3.29998 | 0.001734 | 0.009811 | -1.75215 |
| RPL7AP11 | 1.314148 | 13.44575 | 3.299827 | 0.001735 | 0.009814 | -1.75258 |
| TMEM40 | -1.0418 | 13.14619 | -3.29953 | 0.001736 | 0.009817 | -1.75341 |
| IGHV3-62 | -1.70487 | 12.54509 | -3.29951 | 0.001736 | 0.009817 | -1.75345 |
| DYRK3 | -1.00762 | 11.79753 | -3.29934 | 0.001737 | 0.009821 | -1.75392 |
| MPP2 | 1.451327 | 13.0139 | 3.299182 | 0.001738 | 0.009823 | -1.75435 |
| AC068279.1 | 1.072432 | 13.23159 | 3.296214 | 0.001753 | 0.009895 | -1.76248 |
| SHROOM2 | -2.71166 | 11.25396 | -3.29534 | 0.001758 | 0.009918 | -1.76487 |
| AFF3 | 1.173709 | 13.22418 | 3.293215 | 0.001769 | 0.009968 | -1.77069 |
| HEATR9 | 1.081597 | 13.0108 | 3.291933 | 0.001776 | 0.009994 | -1.7742 |
| CXCL9 | 1.281811 | 12.76985 | 3.290723 | 0.001782 | 0.010024 | -1.77751 |
| IL1R1 | -1.55393 | 12.82077 | -3.29039 | 0.001784 | 0.010032 | -1.77843 |
| ENPP7P5 | -1.54782 | 12.8412 | -3.28793 | 0.001797 | 0.010092 | -1.78516 |
| ATRNL1 | 1.270626 | 13.25827 | 3.287785 | 0.001798 | 0.010094 | -1.78555 |
| SUMO1P3 | -1.92648 | 12.96637 | -3.28759 | 0.001799 | 0.010098 | -1.78607 |
| UGT2A3 | 1.974842 | 11.96309 | 3.287069 | 0.001801 | 0.010108 | -1.7875 |
| TRAJ7 | 1.477114 | 13.03372 | 3.285804 | 0.001808 | 0.010142 | -1.79096 |
| P4HA2 | -1.05086 | 13.24011 | -3.28561 | 0.001809 | 0.010146 | -1.7915 |
| FSCN3 | -1.02772 | 13.22544 | -3.2834 | 0.001821 | 0.010199 | -1.79753 |
| AC010878.1 | -1.44972 | 13.00134 | -3.28317 | 0.001822 | 0.010204 | -1.79816 |
| AC022795.1 | 1.074053 | 14.17917 | 3.280946 | 0.001834 | 0.010256 | -1.80423 |
| AC058822.1 | -2.20226 | 12.37604 | -3.28047 | 0.001837 | 0.010264 | -1.80553 |
| NTN5 | 1.306386 | 13.00315 | 3.27982 | 0.00184 | 0.010271 | -1.8073 |
| IGHV3-38 | -1.83354 | 12.40059 | -3.27889 | 0.001845 | 0.010291 | -1.80984 |
| AL354877.1 | -1.44544 | 12.82568 | -3.27775 | 0.001851 | 0.01032 | -1.81295 |
| TRGC1 | 1.502263 | 12.74038 | 3.277423 | 0.001853 | 0.010327 | -1.81385 |
| CNTN4 | 1.34371 | 13.20389 | 3.27738 | 0.001853 | 0.010327 | -1.81396 |
| MTCYBP23 | -1.69348 | 13.03221 | -3.27703 | 0.001855 | 0.010334 | -1.81492 |
| PADI2 | -1.04861 | 13.17444 | -3.27631 | 0.001859 | 0.010352 | -1.81689 |
| RPSAP9 | 1.07169 | 13.30966 | 3.276207 | 0.00186 | 0.010353 | -1.81716 |
| Z97353.1 | 1.297895 | 13.35748 | 3.276157 | 0.00186 | 0.010353 | -1.8173 |
| FRMD1 | -1.10639 | 13.30703 | -3.27599 | 0.001861 | 0.010356 | -1.81775 |
| GDF6 | -1.51074 | 12.9209 | -3.27586 | 0.001862 | 0.010359 | -1.81812 |
| HDGFP1 | 3.169656 | 11.3149 | 3.275682 | 0.001863 | 0.01036 | -1.8186 |
| LRRC55 | -1.11817 | 13.34853 | -3.27511 | 0.001866 | 0.010371 | -1.82017 |
| TBX3 | -1.50343 | 12.41991 | -3.27506 | 0.001866 | 0.010371 | -1.82029 |
| HDC | 2.922527 | 11.75863 | 3.2746 | 0.001869 | 0.010381 | -1.82155 |
| TRAJ17 | 1.71847 | 13.01641 | 3.274306 | 0.00187 | 0.010387 | -1.82235 |
| TNFSF11 | 1.348991 | 13.05482 | 3.273258 | 0.001876 | 0.010411 | -1.82521 |
| AC005377.1 | -1.62308 | 13.47608 | -3.2722 | 0.001882 | 0.010438 | -1.82809 |
| NRXN1 | 1.187435 | 13.0923 | 3.271034 | 0.001888 | 0.010462 | -1.83127 |
| LYVE1 | -1.14067 | 13.15152 | -3.26928 | 0.001898 | 0.010508 | -1.83604 |
| ZNF358 | 1.370364 | 13.25415 | 3.267672 | 0.001907 | 0.010535 | -1.84043 |
| SAMD9L | -1.15341 | 13.31337 | -3.26598 | 0.001916 | 0.010579 | -1.84502 |
| AC048351.2 | 1.243426 | 13.17229 | 3.265123 | 0.001921 | 0.010599 | -1.84737 |
| DNTT | 1.541489 | 13.04046 | 3.263602 | 0.00193 | 0.010636 | -1.8515 |
| SPINK13 | -1.3473 | 13.54772 | -3.26342 | 0.001931 | 0.01064 | -1.85201 |
| OR1X5P | 1.18098 | 13.83109 | 3.263082 | 0.001933 | 0.010646 | -1.85292 |
| C8orf89 | 1.18286 | 13.49642 | 3.262392 | 0.001937 | 0.01066 | -1.8548 |
| KLRC3 | 1.465654 | 12.86507 | 3.26133 | 0.001943 | 0.01068 | -1.85768 |
| ST13P3 | 1.085771 | 13.57749 | 3.260965 | 0.001945 | 0.010687 | -1.85868 |
| ZIK1P1 | -1.23357 | 13.09587 | -3.259 | 0.001956 | 0.010739 | -1.86401 |
| RNF165 | 1.11178 | 13.20477 | 3.257906 | 0.001962 | 0.010769 | -1.86699 |
| C1QTNF2 | -1.23489 | 13.25513 | -3.25248 | 0.001994 | 0.010855 | -1.88174 |
| RNF150 | 1.534271 | 12.78152 | 3.252335 | 0.001995 | 0.010858 | -1.88213 |
| KLHL34 | 1.136294 | 13.23523 | 3.251382 | 0.002 | 0.010875 | -1.88471 |
| MMP11 | 1.079813 | 13.28099 | 3.250738 | 0.002004 | 0.01089 | -1.88646 |
| ACCS | 1.1126 | 12.94024 | 3.249076 | 0.002014 | 0.010934 | -1.89097 |
| CDK15 | 1.624898 | 12.64778 | 3.248932 | 0.002015 | 0.010937 | -1.89136 |
| RPS15AP1 | 1.307905 | 13.29534 | 3.248009 | 0.00202 | 0.010961 | -1.89386 |
| SLC26A7 | 1.494094 | 13.0424 | 3.247851 | 0.002021 | 0.010964 | -1.89429 |
| RPL35AP26 | -1.24293 | 13.24641 | -3.24772 | 0.002022 | 0.010964 | -1.89466 |
| CEL | -1.26026 | 12.99712 | -3.24683 | 0.002027 | 0.010989 | -1.89707 |
| H3C2 | -1.52299 | 12.65068 | -3.24502 | 0.002038 | 0.011035 | -1.90197 |
| SMIM34A | -1.97697 | 12.63461 | -3.24468 | 0.00204 | 0.011044 | -1.90289 |
| EZHIP | 1.335025 | 13.09642 | 3.242813 | 0.002051 | 0.011095 | -1.90795 |
| GCNT7 | -1.18169 | 13.16621 | -3.24136 | 0.00206 | 0.011138 | -1.91189 |
| SHANK1 | 1.222303 | 13.16598 | 3.241222 | 0.00206 | 0.01114 | -1.91226 |
| MYO1D | -1.0007 | 13.21759 | -3.24063 | 0.002064 | 0.011158 | -1.91387 |
| BICDL2 | -1.82867 | 12.79267 | -3.2405 | 0.002065 | 0.01116 | -1.91422 |
| IGHV1-2 | -2.5246 | 11.93906 | -3.23901 | 0.002074 | 0.011196 | -1.91825 |
| MAOA | -3.89619 | 9.777239 | -3.23784 | 0.002081 | 0.011227 | -1.92142 |
| AC090515.2 | 1.299852 | 13.3375 | 3.235859 | 0.002093 | 0.011281 | -1.92678 |
| LRIT3 | 1.272828 | 13.03218 | 3.235231 | 0.002097 | 0.011298 | -1.92848 |
| TMIGD2 | 1.156092 | 13.14762 | 3.233789 | 0.002106 | 0.011334 | -1.93238 |
| ST6GALNAC3 | -1.13134 | 13.08032 | -3.23351 | 0.002107 | 0.011341 | -1.93314 |
| C16orf96 | 1.127995 | 13.14945 | 3.232765 | 0.002112 | 0.01136 | -1.93515 |
| VWF | -1.25818 | 12.78516 | -3.23256 | 0.002113 | 0.011364 | -1.93571 |
| GFRA2 | 1.500648 | 12.65254 | 3.232046 | 0.002116 | 0.011373 | -1.93709 |
| DOC2B | -1.85475 | 12.0181 | -3.23102 | 0.002123 | 0.011403 | -1.93986 |
| CD163 | -1.66717 | 12.67653 | -3.23093 | 0.002123 | 0.011404 | -1.9401 |
| AC004997.1 | 1.613788 | 12.87657 | 3.230701 | 0.002125 | 0.011409 | -1.94073 |
| IL18RAP | -1.28345 | 13.17009 | -3.23029 | 0.002127 | 0.01142 | -1.94185 |
| C6orf223 | -1.97935 | 12.06859 | -3.22972 | 0.002131 | 0.011435 | -1.94338 |
| IGFBP4 | 1.025832 | 13.24004 | 3.229415 | 0.002133 | 0.011443 | -1.9442 |
| ZNF385B | -1.57135 | 12.52398 | -3.22807 | 0.002141 | 0.011483 | -1.94783 |
| SLC22A31 | -1.34568 | 12.65174 | -3.2275 | 0.002145 | 0.0115 | -1.94936 |
| DMBT1L1 | -1.07452 | 13.25975 | -3.22728 | 0.002146 | 0.011506 | -1.94996 |
| HOOK1 | 1.147131 | 13.25415 | 3.22722 | 0.002146 | 0.011506 | -1.95013 |
| IQUB | -1.03238 | 13.22671 | -3.22642 | 0.002151 | 0.011531 | -1.95229 |
| RPS27AP2 | 1.304779 | 13.34541 | 3.224916 | 0.002161 | 0.011575 | -1.95635 |
| RIBC2 | -1.58826 | 12.23117 | -3.2246 | 0.002163 | 0.011582 | -1.95721 |
| TRAV30 | 1.549046 | 12.79428 | 3.22398 | 0.002167 | 0.011599 | -1.95888 |
| AL021937.2 | -1.2362 | 13.66494 | -3.22308 | 0.002172 | 0.011617 | -1.96131 |
| FTCDNL1 | 1.009919 | 13.12618 | 3.221465 | 0.002183 | 0.011661 | -1.96566 |
| PITX1 | -1.45736 | 12.26366 | -3.22125 | 0.002184 | 0.011666 | -1.96624 |
| PRL | -1.8431 | 12.59187 | -3.21962 | 0.002194 | 0.011716 | -1.97064 |
| AC073585.1 | 1.59754 | 13.02305 | 3.219358 | 0.002196 | 0.011722 | -1.97135 |
| TRAV6 | 1.207548 | 13.09802 | 3.218505 | 0.002202 | 0.011743 | -1.97365 |
| SLC38A11 | 1.485413 | 13.0484 | 3.21753 | 0.002208 | 0.01177 | -1.97627 |
| GRIK1 | -2.13367 | 12.71273 | -3.21564 | 0.00222 | 0.011825 | -1.98137 |
| IGKV3D-7 | -2.15298 | 12.46913 | -3.21482 | 0.002225 | 0.011847 | -1.98358 |
| HMGN2P28 | -1.29949 | 13.59079 | -3.21404 | 0.00223 | 0.011862 | -1.98569 |
| AC093591.1 | 1.952186 | 12.47154 | 3.213694 | 0.002233 | 0.011867 | -1.98661 |
| TRAJ8 | 1.635898 | 13.0852 | 3.212648 | 0.002239 | 0.011897 | -1.98943 |
| SASH1 | 1.210246 | 12.97596 | 3.212415 | 0.002241 | 0.011903 | -1.99005 |
| AC092017.2 | 1.357453 | 13.32722 | 3.210329 | 0.002255 | 0.01196 | -1.99567 |
| MOCS1 | 1.483222 | 12.83007 | 3.210203 | 0.002255 | 0.011963 | -1.99601 |
| AC027601.1 | -1.23488 | 13.22726 | -3.20985 | 0.002258 | 0.011971 | -1.99695 |
| IGHV3-76 | -1.84566 | 12.57713 | -3.20796 | 0.00227 | 0.012026 | -2.00204 |
| SLC10A6 | -1.31016 | 13.7154 | -3.20641 | 0.00228 | 0.012072 | -2.00621 |
| MARCO | -1.24924 | 13.06401 | -3.20615 | 0.002282 | 0.012076 | -2.00692 |
| LURAP1 | -1.09665 | 13.19403 | -3.20555 | 0.002286 | 0.012092 | -2.00854 |
| RPS20P14 | 1.228283 | 13.39935 | 3.204563 | 0.002293 | 0.012124 | -2.01118 |
| XCL2 | 1.877359 | 12.62872 | 3.203517 | 0.0023 | 0.012155 | -2.01399 |
| CR933540.2 | 1.010805 | 13.58156 | 3.203114 | 0.002302 | 0.012164 | -2.01507 |
| FCRLA | 1.171704 | 13.24707 | 3.197915 | 0.002337 | 0.012322 | -2.02904 |
| GDF7 | 1.432382 | 13.0465 | 3.197908 | 0.002337 | 0.012322 | -2.02906 |
| ANP32BP1 | 1.123409 | 13.51887 | 3.197549 | 0.00234 | 0.012332 | -2.03002 |
| AC007494.1 | -1.5751 | 13.21808 | -3.1956 | 0.002353 | 0.012376 | -2.03525 |
| COL5A3 | 1.870783 | 12.55989 | 3.194559 | 0.00236 | 0.012409 | -2.03804 |
| MTDHP3 | -2.35121 | 12.18045 | -3.19423 | 0.002362 | 0.012419 | -2.03893 |
| CLEC4M | -1.12287 | 13.16907 | -3.19387 | 0.002365 | 0.01243 | -2.03989 |
| SERPINB10 | -1.93208 | 11.76289 | -3.19123 | 0.002383 | 0.012517 | -2.04697 |
| OR10V2P | -1.55773 | 13.13109 | -3.1904 | 0.002389 | 0.012537 | -2.04919 |
| EPB41L4B | -3.02032 | 10.76338 | -3.19034 | 0.002389 | 0.012537 | -2.04936 |
| YBX1P10 | 1.174148 | 13.1224 | 3.190305 | 0.002389 | 0.012537 | -2.04945 |
| SFT2D3 | 2.477347 | 12.39548 | 3.188781 | 0.0024 | 0.012582 | -2.05353 |
| IGKV1OR10-1 | -2.2961 | 12.24231 | -3.18539 | 0.002424 | 0.012681 | -2.0626 |
| TACSTD2 | -2.12576 | 11.6675 | -3.18418 | 0.002432 | 0.012716 | -2.06584 |
| LINGO2 | 1.963778 | 12.69948 | 3.184154 | 0.002432 | 0.012716 | -2.06592 |
| ANTXRLP1 | 1.437197 | 13.01022 | 3.181998 | 0.002448 | 0.012782 | -2.07169 |
| IGKV1D-42 | -1.82324 | 12.47981 | -3.18175 | 0.002449 | 0.012789 | -2.07235 |
| ZNF860 | 1.320479 | 13.17593 | 3.180608 | 0.002457 | 0.012823 | -2.07541 |
| APOA2 | -1.14365 | 13.12463 | -3.17836 | 0.002473 | 0.012895 | -2.08143 |
| KIAA0408 | 1.832989 | 12.71673 | 3.178093 | 0.002475 | 0.012903 | -2.08214 |
| AOX2P | -1.10597 | 13.09074 | -3.17614 | 0.002489 | 0.012967 | -2.08736 |
| TRAJ21 | 1.687604 | 13.04886 | 3.175872 | 0.002491 | 0.012972 | -2.08807 |
| AC133435.1 | 1.24476 | 13.16877 | 3.175476 | 0.002494 | 0.012985 | -2.08913 |
| ERG | -1.80254 | 11.88146 | -3.17533 | 0.002495 | 0.012988 | -2.08951 |
| OAS1 | -1.40299 | 13.17189 | -3.1745 | 0.002501 | 0.013015 | -2.09173 |
| MTUS1 | 1.503935 | 12.81644 | 3.174064 | 0.002504 | 0.013029 | -2.0929 |
| AC087283.1 | -1.27722 | 13.24389 | -3.17379 | 0.002506 | 0.013035 | -2.09363 |
| PRKG2 | 1.05017 | 13.25261 | 3.173407 | 0.002509 | 0.013047 | -2.09465 |
| RD3L | -1.7067 | 12.97184 | -3.17183 | 0.002521 | 0.013095 | -2.09888 |
| RPSAP19 | 1.559103 | 13.03651 | 3.17146 | 0.002523 | 0.013098 | -2.09985 |
| AC104763.1 | -1.371 | 12.38407 | -3.17107 | 0.002526 | 0.013108 | -2.10089 |
| CMA1 | -1.40059 | 13.17808 | -3.1705 | 0.00253 | 0.013123 | -2.10242 |
| OR51AB1P | 1.571587 | 12.24957 | 3.170106 | 0.002533 | 0.013131 | -2.10347 |
| SAP30 | -1.32867 | 13.02929 | -3.16826 | 0.002547 | 0.013183 | -2.1084 |
| RPL31P63 | 1.265298 | 13.41881 | 3.166728 | 0.002558 | 0.013235 | -2.11248 |
| ZNF577 | 1.043468 | 13.31146 | 3.166702 | 0.002558 | 0.013235 | -2.11255 |
| AP001781.2 | -1.96946 | 12.63365 | -3.16402 | 0.002578 | 0.013327 | -2.1197 |
| AC079922.1 | 1.28333 | 13.30272 | 3.16367 | 0.002581 | 0.013336 | -2.12063 |
| S100A3 | -1.0515 | 12.88242 | -3.16326 | 0.002584 | 0.013344 | -2.12173 |
| AL391280.2 | -1.29502 | 13.03267 | -3.16224 | 0.002591 | 0.013371 | -2.12445 |
| IGHV7-34-1 | -1.85022 | 12.10223 | -3.16143 | 0.002597 | 0.013395 | -2.12661 |
| BCL10 | -1.46588 | 13.17335 | -3.16125 | 0.002599 | 0.013398 | -2.12708 |
| KLHL41 | 1.387069 | 13.20092 | 3.161157 | 0.002599 | 0.013399 | -2.12733 |
| PDGFD | 1.022225 | 13.26284 | 3.159889 | 0.002609 | 0.013434 | -2.1307 |
| MAMLD1 | 1.048716 | 12.87391 | 3.156528 | 0.002634 | 0.013544 | -2.13965 |
| SCN4B | 1.139488 | 13.17959 | 3.155357 | 0.002643 | 0.013578 | -2.14276 |
| ZG16 | -1.28174 | 13.0688 | -3.15432 | 0.002651 | 0.013605 | -2.14552 |
| IL7 | 1.044927 | 13.28396 | 3.153676 | 0.002656 | 0.013625 | -2.14724 |
| GAPDHP52 | -1.31059 | 13.25933 | -3.15311 | 0.00266 | 0.013636 | -2.14875 |
| DPPA2P2 | -1.46579 | 13.25555 | -3.15216 | 0.002668 | 0.013669 | -2.15127 |
| GNRH2 | -1.33195 | 13.30471 | -3.152 | 0.002669 | 0.013672 | -2.15169 |
| IGKV2D-24 | -2.09703 | 11.6067 | -3.15152 | 0.002673 | 0.013682 | -2.15297 |
| FOXI3 | -1.34948 | 13.28008 | -3.15134 | 0.002674 | 0.013682 | -2.15345 |
| WNT16 | 1.294187 | 13.18583 | 3.148038 | 0.002699 | 0.013784 | -2.16222 |
| RGL4 | -1.19718 | 12.69363 | -3.14562 | 0.002718 | 0.013858 | -2.16864 |
| PALMD | -1.20179 | 13.05846 | -3.14561 | 0.002718 | 0.013858 | -2.16866 |
| STRADB | 1.366694 | 11.78677 | 3.143922 | 0.002732 | 0.013916 | -2.17315 |
| GALR2 | -1.4756 | 13.12212 | -3.14377 | 0.002733 | 0.013919 | -2.17355 |
| TRIM9 | -1.0439 | 13.20491 | -3.14341 | 0.002736 | 0.013922 | -2.17451 |
| HK2P1 | -2.06278 | 12.12335 | -3.14243 | 0.002743 | 0.01395 | -2.17712 |
| RD3 | -1.29074 | 13.21839 | -3.14204 | 0.002746 | 0.013961 | -2.17815 |
| ADAMTS8 | 1.105738 | 13.26801 | 3.14101 | 0.002755 | 0.013993 | -2.18087 |
| SDHDP6 | -2.00537 | 12.71307 | -3.14003 | 0.002762 | 0.014025 | -2.18347 |
| AC135068.9 | -1.79786 | 12.53545 | -3.13897 | 0.002771 | 0.014061 | -2.18627 |
| CDC20P1 | -1.93665 | 12.53953 | -3.13881 | 0.002772 | 0.014062 | -2.1867 |
| DENND5B | -1.44967 | 13.02706 | -3.13839 | 0.002775 | 0.014077 | -2.18782 |
| FAM3C2 | -2.31517 | 12.76861 | -3.13658 | 0.00279 | 0.014139 | -2.19262 |
| CACNA2D1 | -1.38752 | 12.93524 | -3.13651 | 0.00279 | 0.014139 | -2.19279 |
| AL354718.1 | 1.089664 | 13.27387 | 3.135856 | 0.002796 | 0.014154 | -2.19453 |
| SLC25A39 | 1.584517 | 11.54591 | 3.134779 | 0.002804 | 0.014188 | -2.19738 |
| PRKAA2 | -1.07961 | 13.37513 | -3.13439 | 0.002807 | 0.014199 | -2.1984 |
| ARHGAP20 | 1.494073 | 13.03817 | 3.129055 | 0.002851 | 0.0144 | -2.21253 |
| IGHV3-65 | -1.48384 | 12.92922 | -3.1274 | 0.002864 | 0.014455 | -2.21691 |
| ACTBP14 | -1.41066 | 13.15297 | -3.12716 | 0.002866 | 0.01446 | -2.21755 |
| STMN4 | -1.27768 | 13.32087 | -3.12586 | 0.002877 | 0.014502 | -2.22097 |
| IGHV3OR16-16 | -1.53663 | 13.20949 | -3.12471 | 0.002886 | 0.014537 | -2.22401 |
| AL592148.2 | -1.42245 | 12.99839 | -3.12459 | 0.002887 | 0.01454 | -2.22433 |
| HSPE1-MOB4 | -1.61322 | 12.97749 | -3.12288 | 0.002901 | 0.01459 | -2.22886 |
| NAA11 | -1.33801 | 13.23793 | -3.12235 | 0.002906 | 0.014609 | -2.23025 |
| CLEC18A | -1.53185 | 12.88229 | -3.12182 | 0.00291 | 0.014624 | -2.23166 |
| MTRNR2L3 | 1.08287 | 13.15741 | 3.12105 | 0.002917 | 0.014654 | -2.23369 |
| LGR6 | 1.272405 | 13.00038 | 3.120858 | 0.002918 | 0.01466 | -2.2342 |
| SH2D4B | -1.20531 | 12.98111 | -3.12054 | 0.002921 | 0.014671 | -2.23505 |
| MYO6 | 1.093915 | 13.14811 | 3.11987 | 0.002927 | 0.014691 | -2.23681 |
| ATP1B4 | -1.17827 | 13.18077 | -3.11832 | 0.00294 | 0.014739 | -2.24089 |
| RPSAP22 | -1.17832 | 13.07835 | -3.11686 | 0.002952 | 0.014788 | -2.24475 |
| PRRT4 | -1.98028 | 11.50218 | -3.11682 | 0.002952 | 0.014788 | -2.24485 |
| TCTEX1D1 | -1.625 | 12.01453 | -3.11634 | 0.002956 | 0.014806 | -2.24611 |
| NPW | -1.29815 | 13.3882 | -3.1143 | 0.002974 | 0.01487 | -2.25149 |
| TMEM198 | -1.32931 | 13.05706 | -3.11382 | 0.002978 | 0.014883 | -2.25276 |
| IGHV1-69 | -2.48632 | 12.32289 | -3.11341 | 0.002981 | 0.014892 | -2.25386 |
| PJVK | 1.12465 | 13.03747 | 3.113384 | 0.002981 | 0.014892 | -2.25391 |
| CICP19 | -2.21826 | 12.3789 | -3.11275 | 0.002987 | 0.014913 | -2.25557 |
| RPL21P39 | 1.120472 | 13.73602 | 3.112728 | 0.002987 | 0.014913 | -2.25564 |
| SLC45A1 | 1.138656 | 13.07827 | 3.111578 | 0.002997 | 0.01495 | -2.25868 |
| IGLV4-60 | -2.50861 | 12.08866 | -3.11157 | 0.002997 | 0.01495 | -2.2587 |
| SDR16C5 | 1.453481 | 12.99978 | 3.109872 | 0.003011 | 0.015008 | -2.26317 |
| RPS7P1 | 1.59477 | 12.98987 | 3.105899 | 0.003046 | 0.015149 | -2.27363 |
| SCT | 1.317153 | 13.15421 | 3.105896 | 0.003046 | 0.015149 | -2.27364 |
| TRBV12-5 | 1.799918 | 12.63731 | 3.105139 | 0.003052 | 0.015169 | -2.27563 |
| GPRASP1 | 1.183971 | 13.23208 | 3.103008 | 0.003071 | 0.015252 | -2.28124 |
| LRRC37A7P | -1.21926 | 13.03569 | -3.10287 | 0.003072 | 0.015255 | -2.2816 |
| SEPTIN14P19 | -1.14936 | 13.12297 | -3.10237 | 0.003077 | 0.015266 | -2.28291 |
| SPOCK3 | 1.27223 | 13.12162 | 3.102285 | 0.003077 | 0.015266 | -2.28314 |
| WHRN | 1.26258 | 13.08353 | 3.101008 | 0.003089 | 0.015314 | -2.28649 |
| ENPP5 | 1.33403 | 13.10552 | 3.098948 | 0.003107 | 0.015389 | -2.29191 |
| AP006587.2 | 1.190556 | 13.21973 | 3.09887 | 0.003108 | 0.01539 | -2.29211 |
| LCT | -1.28636 | 13.28114 | -3.09825 | 0.003113 | 0.015404 | -2.29375 |
| PTGER4P2 | -1.53247 | 13.05568 | -3.09806 | 0.003115 | 0.015406 | -2.29423 |
| EMILIN3 | -1.53069 | 13.29087 | -3.09709 | 0.003123 | 0.015435 | -2.29679 |
| KCNH7 | -1.23838 | 13.09246 | -3.09662 | 0.003128 | 0.015453 | -2.29804 |
| RPS9P1 | 1.041479 | 13.09048 | 3.09656 | 0.003128 | 0.015453 | -2.29818 |
| RPS3AP49 | 1.019195 | 14.18544 | 3.095544 | 0.003137 | 0.015493 | -2.30085 |
| STPG4 | 1.379811 | 12.34232 | 3.093746 | 0.003153 | 0.015569 | -2.30557 |
| ZNF365 | 1.275387 | 12.99184 | 3.092436 | 0.003165 | 0.015615 | -2.30901 |
| TRAJ12 | 1.536639 | 13.02576 | 3.087089 | 0.003214 | 0.015826 | -2.32303 |
| RPSAP14 | 1.148539 | 13.20798 | 3.084192 | 0.00324 | 0.015942 | -2.33062 |
| S100P | -1.63906 | 12.58733 | -3.08341 | 0.003247 | 0.015969 | -2.33266 |
| AC183084.2 | -1.19953 | 13.02338 | -3.07943 | 0.003284 | 0.016117 | -2.34308 |
| CHST1 | -1.92059 | 11.94575 | -3.07801 | 0.003298 | 0.016172 | -2.3468 |
| PRY | 1.847976 | 12.75112 | 3.076641 | 0.00331 | 0.016227 | -2.35038 |
| PPIAP45 | 2.245992 | 12.21246 | 3.075967 | 0.003317 | 0.01625 | -2.35214 |
| INHBE | -1.0369 | 13.32554 | -3.07544 | 0.003322 | 0.016266 | -2.35352 |
| AC018644.1 | -1.11898 | 13.11798 | -3.07536 | 0.003322 | 0.016267 | -2.35373 |
| IGBP1P1 | -1.23831 | 13.34327 | -3.07412 | 0.003334 | 0.016317 | -2.35696 |
| PTCRA | -1.15121 | 13.06324 | -3.07344 | 0.003341 | 0.01634 | -2.35874 |
| AC025884.1 | 1.677005 | 12.49783 | 3.072554 | 0.003349 | 0.016379 | -2.36106 |
| EMX1 | -1.58216 | 13.042 | -3.07146 | 0.003359 | 0.016424 | -2.36392 |
| EEF1A1P36 | 1.029084 | 13.76427 | 3.071283 | 0.003361 | 0.01643 | -2.36438 |
| TULP2 | -1.08788 | 13.26942 | -3.07099 | 0.003364 | 0.016438 | -2.36515 |
| KCTD14 | -2.47796 | 12.14485 | -3.07069 | 0.003367 | 0.016447 | -2.36593 |
| AC233755.1 | -2.45316 | 12.21076 | -3.06866 | 0.003386 | 0.016526 | -2.37124 |
| RPSAP58 | 1.905405 | 12.57255 | 3.066978 | 0.003402 | 0.016591 | -2.37561 |
| PSG9 | -1.78218 | 12.70331 | -3.06634 | 0.003409 | 0.016608 | -2.37728 |
| CFAP77 | -2.1308 | 10.88729 | -3.06612 | 0.003411 | 0.016614 | -2.37785 |
| SMOC1 | 1.344589 | 13.15275 | 3.064963 | 0.003422 | 0.016657 | -2.38087 |
| CD1B | 1.873099 | 12.65833 | 3.064304 | 0.003428 | 0.016677 | -2.38259 |
| TRGV9 | 1.261529 | 12.99094 | 3.063703 | 0.003434 | 0.016691 | -2.38416 |
| TRBV7-1 | 1.410343 | 13.19121 | 3.063621 | 0.003435 | 0.016691 | -2.38437 |
| AC008984.3 | -1.13584 | 13.48775 | -3.06178 | 0.003453 | 0.016762 | -2.38917 |
| AC245884.7 | -1.13584 | 13.48775 | -3.06178 | 0.003453 | 0.016762 | -2.38917 |
| AC021148.1 | 1.185274 | 13.148 | 3.060251 | 0.003468 | 0.016818 | -2.39315 |
| LTF | -2.30604 | 11.15877 | -3.05925 | 0.003478 | 0.016858 | -2.39575 |
| IGLC7 | -2.16586 | 11.98215 | -3.05908 | 0.003479 | 0.016863 | -2.39621 |
| SHOX2 | -1.50944 | 12.73765 | -3.05843 | 0.003486 | 0.016886 | -2.39788 |
| BFSP1 | 1.148081 | 13.10065 | 3.057063 | 0.003499 | 0.016946 | -2.40145 |
| HNRNPA1P55 | 1.043976 | 13.93309 | 3.056077 | 0.003509 | 0.016977 | -2.40402 |
| IGHV3-48 | -2.01463 | 12.20833 | -3.05601 | 0.00351 | 0.016978 | -2.4042 |
| IGHV1OR21-1 | -1.97752 | 11.94952 | -3.05421 | 0.003528 | 0.017054 | -2.40889 |
| IGKV1D-13 | -2.96309 | 11.30457 | -3.05382 | 0.003532 | 0.01707 | -2.4099 |
| HIGD1C | -1.14577 | 13.37321 | -3.05228 | 0.003547 | 0.017138 | -2.41389 |
| TGFBI | 1.1914 | 12.97647 | 3.051803 | 0.003552 | 0.017151 | -2.41514 |
| AC141272.1 | -2.18725 | 12.17177 | -3.05095 | 0.00356 | 0.017184 | -2.41734 |
| SH3GL3 | -1.42429 | 12.20744 | -3.04648 | 0.003606 | 0.017369 | -2.42896 |
| ZNF683 | 1.591234 | 12.41485 | 3.045824 | 0.003612 | 0.01739 | -2.43067 |
| UGT2B11 | 2.485694 | 11.67877 | 3.044529 | 0.003625 | 0.01744 | -2.43403 |
| ABCB4 | 1.052485 | 13.25991 | 3.043425 | 0.003637 | 0.017483 | -2.4369 |
| EDNRB | -1.93209 | 12.65403 | -3.04222 | 0.003649 | 0.017531 | -2.44002 |
| OCM | 1.026734 | 13.14628 | 3.041157 | 0.00366 | 0.017567 | -2.44278 |
| AC073464.1 | -1.37056 | 12.66936 | -3.03531 | 0.003721 | 0.017834 | -2.45794 |
| AC139769.1 | 1.07034 | 13.20108 | 3.035001 | 0.003724 | 0.017844 | -2.45874 |
| TPTEP2-CSNK1E | -1.45992 | 12.97765 | -3.03453 | 0.003729 | 0.017864 | -2.45995 |
| PRDM12 | 1.080819 | 13.59461 | 3.034234 | 0.003732 | 0.017874 | -2.46072 |
| CSRNP3 | -1.25683 | 12.93832 | -3.03348 | 0.00374 | 0.017901 | -2.46268 |
| WFDC12 | -1.01025 | 13.38952 | -3.03275 | 0.003748 | 0.017931 | -2.46455 |
| SPOCD1 | -1.21802 | 12.87753 | -3.03245 | 0.003751 | 0.017939 | -2.46534 |
| SDC2 | 1.586965 | 12.65736 | 3.031404 | 0.003762 | 0.017983 | -2.46805 |
| ACSM5 | 1.043048 | 13.43319 | 3.030108 | 0.003776 | 0.018037 | -2.4714 |
| IL1R2 | -2.32882 | 11.72391 | -3.02871 | 0.003791 | 0.018094 | -2.47503 |
| H2BC14 | -1.40356 | 12.70848 | -3.02699 | 0.003809 | 0.018164 | -2.47947 |
| PSG6 | -1.83056 | 11.99986 | -3.02688 | 0.00381 | 0.018165 | -2.47976 |
| TRIM49D2 | 1.67967 | 12.39408 | 3.026621 | 0.003813 | 0.018172 | -2.48042 |
| MAGED4 | 1.143623 | 13.17293 | 3.025934 | 0.003821 | 0.018192 | -2.4822 |
| MMP25 | -1.01551 | 13.19521 | -3.02315 | 0.003851 | 0.018313 | -2.4894 |
| AL121871.1 | 1.077046 | 13.91067 | 3.02278 | 0.003855 | 0.018324 | -2.49034 |
| SRGAP2D | -1.99539 | 12.87808 | -3.02265 | 0.003856 | 0.018324 | -2.49069 |
| PRSS30P | 1.389844 | 12.88504 | 3.021555 | 0.003868 | 0.018377 | -2.49351 |
| TGM3 | 1.66643 | 12.41126 | 3.019922 | 0.003886 | 0.018453 | -2.49772 |
| SERINC2 | -1.19256 | 12.86912 | -3.01972 | 0.003888 | 0.018461 | -2.49825 |
| FOXD4L5 | -1.07677 | 13.18355 | -3.01777 | 0.003909 | 0.018545 | -2.50328 |
| PLA2G1B | 1.077005 | 13.94773 | 3.017687 | 0.00391 | 0.018546 | -2.50349 |
| LRRC43 | 1.196295 | 13.17686 | 3.016003 | 0.003929 | 0.018616 | -2.50783 |
| RPSAP18 | 1.468778 | 12.93698 | 3.015102 | 0.003939 | 0.018642 | -2.51015 |
| OR2T8 | -1.68945 | 10.70702 | -3.01438 | 0.003947 | 0.01867 | -2.51201 |
| DCLK1 | -1.09346 | 13.17991 | -3.01418 | 0.003949 | 0.018676 | -2.51252 |
| LILRP1 | -1.55537 | 12.65385 | -3.01371 | 0.003954 | 0.018693 | -2.51375 |
| RPL23P2 | 1.127724 | 13.14341 | 3.012457 | 0.003968 | 0.018742 | -2.51697 |
| NT5C1B | 1.199142 | 13.19619 | 3.012433 | 0.003968 | 0.018742 | -2.51703 |
| SHD | 1.632532 | 12.74621 | 3.011523 | 0.003978 | 0.018777 | -2.51938 |
| ELAVL4 | 1.40348 | 12.89893 | 3.00916 | 0.004005 | 0.018891 | -2.52546 |
| ID1 | -1.46103 | 12.20039 | -3.0072 | 0.004027 | 0.018981 | -2.53051 |
| MRAP2 | -1.4633 | 12.96643 | -3.00679 | 0.004032 | 0.018996 | -2.53156 |
| GEMIN8P4 | -1.12746 | 12.64775 | -3.00597 | 0.004041 | 0.019034 | -2.53367 |
| BBOF1 | 1.01168 | 12.09118 | 3.005725 | 0.004044 | 0.019044 | -2.5343 |
| GPR33 | 1.239204 | 13.31282 | 3.003756 | 0.004066 | 0.019141 | -2.53937 |
| COL9A3 | -1.45073 | 12.67867 | -3.00255 | 0.00408 | 0.019184 | -2.54246 |
| COL17A1 | -2.2456 | 11.44842 | -3.00205 | 0.004085 | 0.019201 | -2.54374 |
| RPL12P27 | -1.01522 | 13.26577 | -3.00203 | 0.004086 | 0.019201 | -2.54381 |
| KLRC4 | 1.85225 | 12.41631 | 3.000731 | 0.004101 | 0.019259 | -2.54714 |
| PTPRF | -1.25736 | 12.11133 | -3.00063 | 0.004102 | 0.019261 | -2.5474 |
| SLC10A4 | 1.253171 | 13.15165 | 2.99965 | 0.004113 | 0.019305 | -2.54992 |
| UBD | 1.549334 | 12.74324 | 2.999248 | 0.004118 | 0.019324 | -2.55095 |
| CNFN | 1.133976 | 13.0252 | 2.999089 | 0.004119 | 0.019329 | -2.55136 |
| SGCD | 1.610692 | 12.80114 | 2.996576 | 0.004149 | 0.019426 | -2.55781 |
| TPTE2P5 | 1.16482 | 13.09868 | 2.996458 | 0.00415 | 0.019427 | -2.55812 |
| NUTM1 | -1.32683 | 13.45688 | -2.99603 | 0.004155 | 0.019444 | -2.5592 |
| PRB3 | 1.458522 | 13.09886 | 2.995536 | 0.004161 | 0.019462 | -2.56048 |
| IL17D | 1.001512 | 13.21199 | 2.993604 | 0.004183 | 0.019549 | -2.56544 |
| KRT8P46 | -1.01996 | 13.31076 | -2.9935 | 0.004184 | 0.019552 | -2.56571 |
| MAGEA8 | -1.32118 | 13.37216 | -2.99335 | 0.004186 | 0.019557 | -2.56609 |
| WNT1 | 1.212429 | 12.98422 | 2.991073 | 0.004213 | 0.019661 | -2.57193 |
| TRBJ1-2 | 1.404532 | 12.85393 | 2.990958 | 0.004214 | 0.019664 | -2.57223 |
| OR52B3P | -1.1692 | 13.21722 | -2.99057 | 0.004219 | 0.019679 | -2.57322 |
| AP000866.6 | 1.068857 | 13.36593 | 2.990525 | 0.004219 | 0.019679 | -2.57333 |
| LINGO4 | 1.413405 | 13.13612 | 2.98996 | 0.004226 | 0.019696 | -2.57478 |
| AC104843.1 | 1.009326 | 14.20764 | 2.989167 | 0.004235 | 0.019721 | -2.57681 |
| RLN3 | -2.28362 | 12.07812 | -2.98903 | 0.004237 | 0.019724 | -2.57716 |
| KDM4E | 1.274343 | 13.65445 | 2.987702 | 0.004253 | 0.019785 | -2.58057 |
| DNAH10 | -1.53909 | 12.01191 | -2.98711 | 0.00426 | 0.019815 | -2.58209 |
| SPNS3 | 1.15809 | 12.60541 | 2.986732 | 0.004264 | 0.019824 | -2.58305 |
| NECAB1 | -1.10649 | 12.95334 | -2.98377 | 0.0043 | 0.019955 | -2.59063 |
| MTND6P22 | 1.215525 | 13.53354 | 2.983371 | 0.004304 | 0.019968 | -2.59166 |
| NMD3P1 | 1.419346 | 13.14603 | 2.981373 | 0.004329 | 0.020064 | -2.59677 |
| IGHV3-25 | -1.71535 | 12.50017 | -2.9812 | 0.004331 | 0.020067 | -2.5972 |
| PZP | 1.736159 | 12.6243 | 2.980599 | 0.004338 | 0.020092 | -2.59875 |
| RPL29P25 | -1.14017 | 13.16173 | -2.97984 | 0.004347 | 0.020125 | -2.60067 |
| SH2D4A | -1.81722 | 12.34744 | -2.97917 | 0.004355 | 0.02016 | -2.60239 |
| APBB2 | -1.0272 | 13.23011 | -2.97907 | 0.004356 | 0.020161 | -2.60266 |
| TXNDC2 | -1.06704 | 13.26419 | -2.97687 | 0.004383 | 0.020252 | -2.60827 |
| OR1AB1P | -1.21862 | 13.37083 | -2.97627 | 0.004391 | 0.020277 | -2.6098 |
| EMP1 | -1.18566 | 13.07136 | -2.97478 | 0.004409 | 0.020352 | -2.6136 |
| OR2B11 | -1.06893 | 13.21823 | -2.97262 | 0.004435 | 0.020459 | -2.61913 |
| AC066616.2 | -1.53737 | 13.07084 | -2.97241 | 0.004438 | 0.020468 | -2.61968 |
| IGKV2D-18 | -1.5761 | 12.65611 | -2.97225 | 0.00444 | 0.020471 | -2.62008 |
| PTTG4P | -1.4855 | 13.05855 | -2.97101 | 0.004455 | 0.020519 | -2.62323 |
| FCRL6 | 1.188454 | 12.97275 | 2.970671 | 0.00446 | 0.020533 | -2.62411 |
| AC243919.1 | 1.219815 | 12.96841 | 2.969611 | 0.004473 | 0.020584 | -2.62681 |
| TRAJ56 | 1.570649 | 12.87418 | 2.969254 | 0.004477 | 0.020597 | -2.62772 |
| ZNF578 | 1.094812 | 13.24187 | 2.968847 | 0.004482 | 0.020615 | -2.62876 |
| Z99129.1 | -1.08688 | 13.13389 | -2.96813 | 0.004491 | 0.020647 | -2.63059 |
| NCOA4P2 | -1.60688 | 13.1024 | -2.9668 | 0.004508 | 0.020714 | -2.63397 |
| SMC3P1 | 1.402655 | 13.01701 | 2.965637 | 0.004523 | 0.020762 | -2.63694 |
| H3P13 | -1.16606 | 13.26881 | -2.96551 | 0.004524 | 0.020767 | -2.63727 |
| KIF24 | -1.56037 | 12.80974 | -2.96506 | 0.00453 | 0.02078 | -2.6384 |
| RPL12P2 | 1.112736 | 14.03769 | 2.964252 | 0.00454 | 0.02082 | -2.64047 |
| AC098934.1 | -1.52281 | 12.26833 | -2.96338 | 0.004551 | 0.020861 | -2.6427 |
| RPL12P16 | 1.188326 | 13.02532 | 2.960124 | 0.004592 | 0.021029 | -2.65098 |
| MMP27 | -1.94433 | 11.39083 | -2.96007 | 0.004593 | 0.021029 | -2.65112 |
| LCN10 | 1.086267 | 13.23578 | 2.959863 | 0.004596 | 0.021037 | -2.65164 |
| CALHM3 | -1.30866 | 13.39371 | -2.9581 | 0.004618 | 0.021115 | -2.65612 |
| NKX3-1 | 1.452003 | 12.61364 | 2.95448 | 0.004665 | 0.021296 | -2.66533 |
| SPDEF | -1.21769 | 13.32189 | -2.95362 | 0.004676 | 0.021337 | -2.66752 |
| CALR3 | -1.32313 | 13.43065 | -2.95352 | 0.004677 | 0.02134 | -2.66777 |
| EVPLL | -1.3165 | 13.33834 | -2.95225 | 0.004694 | 0.02141 | -2.67099 |
| DIRC3 | 1.188517 | 13.14529 | 2.951996 | 0.004697 | 0.021414 | -2.67164 |
| GPC5 | -1.40991 | 13.02859 | -2.9508 | 0.004713 | 0.021466 | -2.67468 |
| CD177P1 | -2.52309 | 11.03534 | -2.94979 | 0.004726 | 0.02152 | -2.67725 |
| CYP4B1 | -1.02503 | 13.10954 | -2.94679 | 0.004765 | 0.021664 | -2.68485 |
| AC121334.1 | 1.012466 | 14.08172 | 2.943697 | 0.004806 | 0.02183 | -2.6927 |
| FABP6 | -1.44496 | 12.95222 | -2.94337 | 0.004811 | 0.021843 | -2.69352 |
| PNMT | -1.79968 | 12.33066 | -2.94219 | 0.004827 | 0.021908 | -2.69651 |
| LHX1 | -2.25484 | 11.72775 | -2.93929 | 0.004866 | 0.022058 | -2.70386 |
| RSPH10B | -1.08534 | 13.07705 | -2.93888 | 0.004871 | 0.02207 | -2.70491 |
| PGLYRP4 | -1.22005 | 13.32663 | -2.93626 | 0.004907 | 0.022211 | -2.71154 |
| AC103563.1 | -1.63053 | 12.36553 | -2.9358 | 0.004913 | 0.022236 | -2.71269 |
| AKR1B10 | -1.1635 | 13.20019 | -2.93504 | 0.004923 | 0.022279 | -2.71461 |
| AC083923.1 | 1.19605 | 13.35401 | 2.934315 | 0.004933 | 0.022314 | -2.71645 |
| SLC2A3P2 | -1.11321 | 13.25729 | -2.93367 | 0.004942 | 0.022344 | -2.71808 |
| FLT1P1 | -1.37248 | 12.9825 | -2.93327 | 0.004947 | 0.022365 | -2.7191 |
| CASP12 | -1.30431 | 13.2685 | -2.9331 | 0.004949 | 0.022372 | -2.71952 |
| SPP1 | 1.602351 | 11.99418 | 2.932387 | 0.004959 | 0.02241 | -2.72132 |
| CLGN | -1.06594 | 13.0321 | -2.93233 | 0.00496 | 0.02241 | -2.72146 |
| AC245517.5 | -1.46842 | 13.01689 | -2.93153 | 0.004971 | 0.022456 | -2.72348 |
| CCDC42 | 1.145836 | 13.232 | 2.929056 | 0.005005 | 0.022573 | -2.72974 |
| AC234635.3 | 3.136512 | 11.41753 | 2.927833 | 0.005022 | 0.022642 | -2.73283 |
| PKHD1 | -1.36356 | 12.98719 | -2.92732 | 0.005029 | 0.022668 | -2.73412 |
| SLC24A3 | -1.11398 | 12.9097 | -2.92661 | 0.005039 | 0.022706 | -2.73592 |
| WTAPP1 | -1.106 | 13.56747 | -2.92594 | 0.005048 | 0.022743 | -2.73761 |
| PDCD1LG2 | -1.16378 | 13.26379 | -2.9257 | 0.005052 | 0.022752 | -2.73821 |
| AC007066.1 | 1.022033 | 13.69954 | 2.925187 | 0.005059 | 0.022774 | -2.73951 |
| ACRV1 | -1.30068 | 12.87063 | -2.92479 | 0.005064 | 0.022793 | -2.74052 |
| AL592429.1 | -1.45789 | 12.89074 | -2.92445 | 0.005069 | 0.022811 | -2.74137 |
| CYP4F10P | -1.40388 | 13.0174 | -2.92371 | 0.00508 | 0.02285 | -2.74322 |
| IGKV2OR22-3 | -1.44916 | 12.36311 | -2.92336 | 0.005085 | 0.022866 | -2.74413 |
| CFAP73 | 1.208925 | 13.17409 | 2.923209 | 0.005087 | 0.022872 | -2.7445 |
| YWHAQP6 | -1.28201 | 13.49308 | -2.92194 | 0.005104 | 0.022934 | -2.74771 |
| MAP3K7CL | 1.039223 | 13.14692 | 2.921133 | 0.005116 | 0.022976 | -2.74973 |
| GTF2IP13 | -1.92781 | 12.53301 | -2.92106 | 0.005117 | 0.022977 | -2.74991 |
| ZP1 | 1.195062 | 13.22446 | 2.920824 | 0.00512 | 0.022985 | -2.75051 |
| AC004069.2 | -1.31195 | 13.4254 | -2.9199 | 0.005133 | 0.023037 | -2.75284 |
| TRBV24-1 | 1.297602 | 12.82156 | 2.917614 | 0.005166 | 0.023152 | -2.7586 |
| ASB9P1 | -1.51495 | 13.09152 | -2.91755 | 0.005167 | 0.023152 | -2.75876 |
| RNF212 | 1.246008 | 12.74779 | 2.917131 | 0.005173 | 0.023176 | -2.75981 |
| HNRNPA1P36 | -1.14344 | 13.45748 | -2.917 | 0.005174 | 0.02318 | -2.76013 |
| SLC4A9 | -1.46018 | 12.89912 | -2.91457 | 0.005209 | 0.023326 | -2.76625 |
| WDR45P1 | -1.42768 | 13.16005 | -2.91402 | 0.005217 | 0.023354 | -2.76763 |
| FASLG | 1.070451 | 13.15829 | 2.912752 | 0.005235 | 0.023412 | -2.77084 |
| AC012158.1 | -1.32192 | 13.21196 | -2.91135 | 0.005256 | 0.023481 | -2.77435 |
| CHRNA2 | -1.29869 | 12.94042 | -2.91046 | 0.005268 | 0.023526 | -2.7766 |
| TTLL10 | 1.157935 | 13.32756 | 2.905439 | 0.005342 | 0.023803 | -2.78922 |
| CACNA1G | -1.16974 | 13.21967 | -2.90406 | 0.005362 | 0.023876 | -2.79269 |
| AC133134.1 | 1.109818 | 13.56134 | 2.902316 | 0.005388 | 0.023969 | -2.79705 |
| SMARCA1 | 1.279403 | 13.11613 | 2.901115 | 0.005406 | 0.024038 | -2.80007 |
| Z83844.3 | -1.86166 | 12.97563 | -2.90033 | 0.005417 | 0.024086 | -2.80203 |
| C10orf88B | 1.103902 | 13.22927 | 2.899003 | 0.005437 | 0.02415 | -2.80536 |
| EIF2AK2 | -1.01486 | 13.31084 | -2.89893 | 0.005438 | 0.02415 | -2.80554 |
| GPER1 | -1.63608 | 12.75295 | -2.8952 | 0.005494 | 0.02437 | -2.81489 |
| RGS9BP | 1.110316 | 13.222 | 2.893532 | 0.005519 | 0.024453 | -2.81907 |
| AC023050.5 | -1.06131 | 13.30382 | -2.89301 | 0.005527 | 0.024478 | -2.82038 |
| SLC1A1 | 1.313811 | 13.04416 | 2.892615 | 0.005533 | 0.024497 | -2.82136 |
| PTMAP4 | 1.423876 | 12.95349 | 2.892399 | 0.005536 | 0.024504 | -2.8219 |
| GPR20 | 1.604378 | 11.83852 | 2.88934 | 0.005583 | 0.02466 | -2.82956 |
| LGR5 | -1.15955 | 13.40185 | -2.8892 | 0.005585 | 0.024666 | -2.8299 |
| H4C3 | -1.31025 | 13.25508 | -2.88898 | 0.005589 | 0.024673 | -2.83045 |
| AC009779.5 | -1.59682 | 12.95404 | -2.8889 | 0.00559 | 0.024675 | -2.83065 |
| AC074143.1 | -1.43569 | 12.7181 | -2.88606 | 0.005633 | 0.024837 | -2.83776 |
| PCDH1 | 1.137922 | 12.51265 | 2.88506 | 0.005649 | 0.024889 | -2.84026 |
| RBAK-RBAKDN | -1.16702 | 13.2485 | -2.88391 | 0.005667 | 0.02495 | -2.84313 |
| PTMAP8 | 1.008394 | 13.92409 | 2.883892 | 0.005667 | 0.02495 | -2.84317 |
| ETV7 | -1.35616 | 13.08147 | -2.88339 | 0.005675 | 0.024974 | -2.84443 |
| GZMH | 1.198755 | 12.89401 | 2.882635 | 0.005687 | 0.025011 | -2.84631 |
| AGGF1P2 | 1.558039 | 12.88564 | 2.881603 | 0.005703 | 0.025067 | -2.84889 |
| AL359555.4 | -1.46896 | 12.19132 | -2.88062 | 0.005718 | 0.025116 | -2.85135 |
| AC131235.2 | 1.098955 | 13.43099 | 2.879186 | 0.005741 | 0.025178 | -2.85492 |
| DNAH6 | -1.68134 | 12.29105 | -2.8789 | 0.005745 | 0.025191 | -2.85564 |
| RAB43P1 | -1.50943 | 13.07642 | -2.87872 | 0.005748 | 0.025199 | -2.85608 |
| CCL18 | 1.113705 | 13.34017 | 2.87818 | 0.005756 | 0.025226 | -2.85743 |
| FAM167A | 1.322047 | 12.83143 | 2.876875 | 0.005777 | 0.025308 | -2.86068 |
| CLIC3 | 1.322889 | 12.88361 | 2.876392 | 0.005785 | 0.025331 | -2.86189 |
| RPL12P4 | 1.092011 | 13.37884 | 2.874913 | 0.005808 | 0.025422 | -2.86557 |
| CCT5P1 | -1.10903 | 13.42193 | -2.87089 | 0.005872 | 0.02567 | -2.87559 |
| SYDE1 | 1.132048 | 13.18672 | 2.867198 | 0.005932 | 0.025884 | -2.88478 |
| GPC3 | 1.499953 | 12.82736 | 2.866545 | 0.005942 | 0.025913 | -2.8864 |
| IGHV7-56 | -1.79962 | 12.49245 | -2.86504 | 0.005967 | 0.026 | -2.89014 |
| AC023050.1 | -1.24649 | 13.41567 | -2.86427 | 0.005979 | 0.026044 | -2.89207 |
| GSTT4 | -1.12647 | 13.44952 | -2.86296 | 0.006001 | 0.026123 | -2.8953 |
| HNRNPA1P8 | 1.039223 | 13.52817 | 2.862948 | 0.006001 | 0.026123 | -2.89534 |
| OR4C6 | -1.46321 | 13.05223 | -2.86237 | 0.00601 | 0.02616 | -2.89679 |
| ITLN1 | 1.625806 | 11.42133 | 2.860511 | 0.006041 | 0.026274 | -2.9014 |
| OGFOD1P1 | -1.24633 | 13.06435 | -2.85997 | 0.00605 | 0.026305 | -2.90275 |
| DPY19L1P1 | -1.13188 | 12.29266 | -2.85882 | 0.006069 | 0.026365 | -2.9056 |
| AC234301.4 | -2.31785 | 12.20627 | -2.85731 | 0.006094 | 0.026461 | -2.90933 |
| AC138951.1 | -1.47814 | 12.88087 | -2.85731 | 0.006094 | 0.026461 | -2.90935 |
| CU457734.2 | -1.97114 | 12.79036 | -2.85715 | 0.006096 | 0.026466 | -2.90975 |
| IGKV2-26 | -1.5857 | 12.3995 | -2.85678 | 0.006103 | 0.026485 | -2.91066 |
| AQP10 | -1.37524 | 12.73249 | -2.85548 | 0.006124 | 0.02656 | -2.91388 |
| COL1A2 | -1.84034 | 11.52238 | -2.85453 | 0.00614 | 0.02661 | -2.91623 |
| AL589935.2 | -1.24773 | 13.10661 | -2.85352 | 0.006157 | 0.026664 | -2.91874 |
| TMC1 | -1.28514 | 13.08573 | -2.85307 | 0.006164 | 0.026689 | -2.91986 |
| AC093520.2 | -1.03634 | 13.11997 | -2.85287 | 0.006168 | 0.026699 | -2.92035 |
| AC004910.1 | -1.01962 | 13.36069 | -2.85263 | 0.006172 | 0.026709 | -2.92093 |
| TRDV1 | 1.596249 | 12.39334 | 2.852513 | 0.006174 | 0.026714 | -2.92123 |
| RPL5P34 | 1.188186 | 13.26081 | 2.849301 | 0.006228 | 0.02691 | -2.92919 |
| AGAP13P | 1.516625 | 12.80532 | 2.847866 | 0.006252 | 0.026995 | -2.93274 |
| AC116353.2 | -1.12332 | 13.7594 | -2.84675 | 0.006271 | 0.027066 | -2.93549 |
| AC005912.1 | 1.535058 | 12.86083 | 2.846091 | 0.006283 | 0.027099 | -2.93713 |
| TSPAN12 | -1.51431 | 12.30866 | -2.84449 | 0.00631 | 0.027194 | -2.9411 |
| PRG3 | -2.15685 | 9.937171 | -2.84383 | 0.006321 | 0.027238 | -2.94271 |
| WNT9A | 1.143575 | 12.98771 | 2.843723 | 0.006323 | 0.02724 | -2.94299 |
| AL354980.2 | -1.75526 | 12.26387 | -2.84338 | 0.006329 | 0.02726 | -2.94384 |
| CA8 | 1.124139 | 12.30077 | 2.843014 | 0.006335 | 0.027283 | -2.94474 |
| RHBDL3 | -1.21823 | 12.65204 | -2.84253 | 0.006343 | 0.027311 | -2.94593 |
| DLL1 | 1.300977 | 13.01502 | 2.841969 | 0.006353 | 0.027341 | -2.94732 |
| FAM166B | 1.031136 | 13.19699 | 2.841713 | 0.006358 | 0.027351 | -2.94796 |
| IFITM3P1 | -1.66716 | 12.57419 | -2.8408 | 0.006373 | 0.027405 | -2.95021 |
| RTP5 | 1.101923 | 12.87698 | 2.839202 | 0.006401 | 0.027481 | -2.95416 |
| TRPM3 | -1.22005 | 13.27247 | -2.83871 | 0.00641 | 0.027513 | -2.95536 |
| CSMD1 | 1.207915 | 12.43094 | 2.836798 | 0.006443 | 0.027633 | -2.96009 |
| LINC01619 | 1.253215 | 13.05745 | 2.836439 | 0.006449 | 0.027649 | -2.96098 |
| AC011043.1 | -1.30897 | 12.95373 | -2.83633 | 0.006451 | 0.027652 | -2.96124 |
| IGHV2-70D | -2.92739 | 10.55233 | -2.833 | 0.00651 | 0.027852 | -2.96945 |
| TTC39DP | -1.35415 | 13.54959 | -2.83242 | 0.00652 | 0.02788 | -2.97088 |
| AP005212.4 | 1.49942 | 12.86052 | 2.82958 | 0.00657 | 0.028051 | -2.97789 |
| OLFM5P | -1.4455 | 13.03791 | -2.82808 | 0.006597 | 0.028126 | -2.98158 |
| AC009977.1 | 1.514105 | 12.84874 | 2.827638 | 0.006605 | 0.028156 | -2.98267 |
| TIMP3 | -1.53588 | 12.39305 | -2.82696 | 0.006617 | 0.0282 | -2.98435 |
| NCLP1 | -1.08934 | 13.16888 | -2.82558 | 0.006642 | 0.028289 | -2.98774 |
| HYAL4 | -1.55418 | 12.99392 | -2.82541 | 0.006645 | 0.028297 | -2.98816 |
| FAM71F1 | -1.27019 | 13.0085 | -2.82493 | 0.006654 | 0.028319 | -2.98934 |
| IGHV5-10-1 | -3.34518 | 10.87767 | -2.8241 | 0.006669 | 0.028375 | -2.99139 |
| AC247036.3 | -3.34518 | 10.87832 | -2.82409 | 0.006669 | 0.028375 | -2.99139 |
| AL050341.1 | -1.1166 | 13.49809 | -2.8207 | 0.00673 | 0.028604 | -2.99974 |
| SNX9 | -2.3429 | 12.23587 | -2.81989 | 0.006745 | 0.028654 | -3.00171 |
| HLA-H | -1.90292 | 11.85702 | -2.8193 | 0.006756 | 0.028692 | -3.00317 |
| MARK1 | 1.146636 | 13.51929 | 2.817241 | 0.006793 | 0.028824 | -3.00823 |
| H4C14 | -1.01076 | 13.30623 | -2.81643 | 0.006808 | 0.028875 | -3.01022 |
| DYNLRB2 | 1.046963 | 12.96171 | 2.815536 | 0.006825 | 0.028933 | -3.01242 |
| SPINK8 | -1.42349 | 12.65705 | -2.81507 | 0.006833 | 0.028961 | -3.01356 |
| KRT17P2 | 1.842764 | 12.36227 | 2.810725 | 0.006914 | 0.029249 | -3.02422 |
| RPS26P8 | 1.248672 | 13.31153 | 2.809013 | 0.006946 | 0.029356 | -3.02841 |
| DLL3 | 1.039931 | 13.41548 | 2.808337 | 0.006958 | 0.029405 | -3.03007 |
| MTCYBP14 | -1.15662 | 13.13111 | -2.80751 | 0.006974 | 0.02945 | -3.03209 |
| GGN | -1.02781 | 13.35145 | -2.80671 | 0.006989 | 0.029497 | -3.03406 |
| UCN2 | -1.4076 | 13.04234 | -2.80527 | 0.007016 | 0.029583 | -3.03757 |
| PSG3 | -1.3301 | 13.03778 | -2.80495 | 0.007022 | 0.0296 | -3.03835 |
| FABP4 | -1.48864 | 11.67299 | -2.80442 | 0.007032 | 0.029624 | -3.03967 |
| CATSPERD | -1.23624 | 13.35741 | -2.80439 | 0.007033 | 0.029624 | -3.03973 |
| ACTL8 | -1.10495 | 14.13146 | -2.80426 | 0.007035 | 0.029631 | -3.04005 |
| TRBV27 | 1.8795 | 12.56972 | 2.803552 | 0.007049 | 0.029679 | -3.04178 |
| AC231657.3 | 1.508755 | 12.58496 | 2.802157 | 0.007075 | 0.029778 | -3.0452 |
| EPHB2 | -1.41058 | 13.14153 | -2.80184 | 0.007081 | 0.029796 | -3.04598 |
| LCN2 | -2.09681 | 11.08756 | -2.80165 | 0.007085 | 0.029807 | -3.04644 |
| FRMD7 | -1.08691 | 13.88646 | -2.80034 | 0.00711 | 0.029882 | -3.04964 |
| PLIN5 | -1.24022 | 12.85274 | -2.8003 | 0.007111 | 0.029882 | -3.04974 |
| RPL12P15 | 1.032516 | 13.55046 | 2.799081 | 0.007134 | 0.029946 | -3.05271 |
| DZIP1 | -1.24195 | 13.09944 | -2.79857 | 0.007144 | 0.02998 | -3.05397 |
| GCK | 1.253465 | 13.16872 | 2.798314 | 0.007149 | 0.029992 | -3.05459 |
| HTR1F | -1.28648 | 12.99871 | -2.79815 | 0.007152 | 0.029993 | -3.05499 |
| SORCS3 | 1.789786 | 12.18028 | 2.798134 | 0.007152 | 0.029993 | -3.05503 |
| RFPL1 | 1.051422 | 13.26477 | 2.796788 | 0.007178 | 0.030074 | -3.05832 |
| CLC | 1.605505 | 12.35156 | 2.795969 | 0.007194 | 0.030135 | -3.06031 |
| BLACE | -1.0374 | 13.24606 | -2.79517 | 0.00721 | 0.030183 | -3.06227 |
| PRLR | 1.336258 | 12.94547 | 2.794624 | 0.00722 | 0.030219 | -3.0636 |
| PFKFB2 | -1.4528 | 12.61762 | -2.79416 | 0.007229 | 0.030249 | -3.06474 |
| MESP1 | -1.22895 | 13.19083 | -2.79252 | 0.007261 | 0.030347 | -3.06873 |
| BPI | -1.81836 | 11.3648 | -2.79245 | 0.007263 | 0.030347 | -3.06891 |
| LPAR4 | -1.17572 | 13.20056 | -2.79208 | 0.00727 | 0.030363 | -3.0698 |
| MYBPC2 | 1.379332 | 12.96388 | 2.79012 | 0.007308 | 0.030507 | -3.07459 |
| NDUFC2-KCTD14 | -2.2779 | 12.16529 | -2.78874 | 0.007335 | 0.030612 | -3.07795 |
| RPS29P5 | -1.33355 | 13.00423 | -2.7876 | 0.007358 | 0.030686 | -3.08073 |
| RPL7L1P1 | 1.197025 | 13.31862 | 2.787075 | 0.007368 | 0.030713 | -3.082 |
| TREML4 | -1.35194 | 12.9629 | -2.78563 | 0.007397 | 0.030813 | -3.08552 |
| FOXD4L1 | -1.31338 | 12.49965 | -2.78473 | 0.007415 | 0.030862 | -3.08771 |
| IGLV1-62 | -1.12566 | 13.24131 | -2.78373 | 0.007435 | 0.030928 | -3.09015 |
| TSPEAR | 1.778979 | 11.7733 | 2.783451 | 0.00744 | 0.030943 | -3.09083 |
| FRG1KP | -1.22804 | 13.11228 | -2.78208 | 0.007467 | 0.031043 | -3.09415 |
| HNRNPA1P10 | 1.596819 | 12.95482 | 2.776062 | 0.007589 | 0.031457 | -3.10879 |
| AC100854.1 | 1.187056 | 13.20815 | 2.776016 | 0.00759 | 0.031457 | -3.1089 |
| HJV | -1.24777 | 13.29875 | -2.77582 | 0.007594 | 0.031465 | -3.10938 |
| STMN1P1 | -1.08197 | 13.38307 | -2.77482 | 0.007614 | 0.031529 | -3.11182 |
| RPS26P31 | 1.256988 | 13.06415 | 2.774425 | 0.007622 | 0.031544 | -3.11277 |
| AC010507.1 | -1.02045 | 13.00683 | -2.77396 | 0.007632 | 0.031578 | -3.11389 |
| A3GALT2 | -1.68407 | 12.7585 | -2.77044 | 0.007704 | 0.031834 | -3.12245 |
| RPSAP75 | -1.4173 | 13.13189 | -2.76777 | 0.007759 | 0.031996 | -3.1289 |
| ANTXR1 | -1.09937 | 13.24695 | -2.76719 | 0.007771 | 0.032041 | -3.13031 |
| CCDC173 | -1.34543 | 12.74739 | -2.76699 | 0.007776 | 0.032055 | -3.13081 |
| OR56B4 | -1.14217 | 13.3375 | -2.76668 | 0.007782 | 0.032077 | -3.13155 |
| PSMD8P1 | -1.10361 | 13.53755 | -2.76562 | 0.007804 | 0.032159 | -3.13411 |
| TSGA10IP | 1.135639 | 13.42401 | 2.765425 | 0.007808 | 0.032171 | -3.13459 |
| FSTL4 | -1.22297 | 13.04486 | -2.76516 | 0.007814 | 0.032189 | -3.13522 |
| GPR174 | 1.297401 | 12.98991 | 2.764851 | 0.00782 | 0.032212 | -3.13598 |
| FANCD2OS | -1.07344 | 13.31572 | -2.76238 | 0.007872 | 0.032403 | -3.14196 |
| AF279873.2 | 1.07865 | 13.47964 | 2.761237 | 0.007896 | 0.032467 | -3.14473 |
| MYL12BP2 | -1.48984 | 12.93933 | -2.75964 | 0.00793 | 0.032582 | -3.14859 |
| LHX4 | -1.35427 | 12.62188 | -2.7596 | 0.00793 | 0.032582 | -3.14868 |
| IGHV3OR16-6 | -1.50804 | 11.35525 | -2.75953 | 0.007932 | 0.032584 | -3.14886 |
| HCG4 | 1.305236 | 13.09668 | 2.757588 | 0.007973 | 0.032731 | -3.15355 |
| AC092953.1 | -1.16232 | 13.17605 | -2.75704 | 0.007985 | 0.032774 | -3.15488 |
| NEURL3 | -1.29391 | 13.07518 | -2.75549 | 0.008018 | 0.032888 | -3.15863 |
| CUZD1 | 1.35217 | 13.05113 | 2.753786 | 0.008054 | 0.033019 | -3.16273 |
| C11orf88 | -1.02322 | 13.27143 | -2.75231 | 0.008086 | 0.033122 | -3.1663 |
| IDO1 | 1.889222 | 12.16043 | 2.752223 | 0.008088 | 0.033122 | -3.1665 |
| ATP5MFP4 | -1.20219 | 13.12622 | -2.75222 | 0.008088 | 0.033122 | -3.16651 |
| AC010332.1 | 1.144734 | 13.32988 | 2.751945 | 0.008094 | 0.033141 | -3.16718 |
| SHROOM3 | -1.06472 | 13.35446 | -2.75119 | 0.00811 | 0.033199 | -3.16899 |
| NT5DC4 | -1.06847 | 13.16096 | -2.74987 | 0.008139 | 0.033289 | -3.17218 |
| RPL29P11 | 1.20253 | 13.21994 | 2.749362 | 0.00815 | 0.03333 | -3.17341 |
| SGCE | 1.010945 | 13.25619 | 2.747983 | 0.00818 | 0.03343 | -3.17673 |
| AKR1D1P1 | 1.325369 | 13.03256 | 2.74648 | 0.008213 | 0.033545 | -3.18035 |
| CICP4 | -1.85792 | 12.51467 | -2.7459 | 0.008225 | 0.033584 | -3.18176 |
| THBS1 | -1.13526 | 12.99054 | -2.74512 | 0.008243 | 0.033631 | -3.18364 |
| AC010422.5 | -3.19257 | 10.39114 | -2.74476 | 0.00825 | 0.033658 | -3.1845 |
| TRBV4-1 | 1.215727 | 12.75972 | 2.740048 | 0.008354 | 0.034009 | -3.19584 |
| SLC25A27 | 1.003503 | 13.14931 | 2.73633 | 0.008437 | 0.0343 | -3.20477 |
| LGALS9B | 1.036735 | 13.12516 | 2.735851 | 0.008448 | 0.03433 | -3.20592 |
| IGLV3-29 | -1.7174 | 12.69457 | -2.73585 | 0.008448 | 0.03433 | -3.20593 |
| TRBV7-3 | 1.214186 | 12.76156 | 2.734932 | 0.008469 | 0.0344 | -3.20813 |
| TRBV25-1 | 1.292461 | 12.92316 | 2.734872 | 0.00847 | 0.034401 | -3.20828 |
| IGHVII-60-1 | -1.17075 | 13.47459 | -2.73479 | 0.008472 | 0.034404 | -3.20847 |
| RPL26P19 | 1.253533 | 13.09308 | 2.733963 | 0.008491 | 0.034452 | -3.21046 |
| RPL36AP15 | -1.10712 | 13.40227 | -2.73355 | 0.0085 | 0.03448 | -3.21146 |
| RPL12P42 | 1.022144 | 13.48539 | 2.732772 | 0.008517 | 0.034539 | -3.21332 |
| NSA2P3 | 1.282494 | 12.94066 | 2.732703 | 0.008519 | 0.034539 | -3.21349 |
| CCR12P | 1.668491 | 12.6422 | 2.732553 | 0.008522 | 0.034549 | -3.21385 |
| MNX1 | -1.23546 | 12.66211 | -2.73234 | 0.008527 | 0.034549 | -3.21435 |
| HTR1B | 1.03089 | 13.23725 | 2.732134 | 0.008532 | 0.034564 | -3.21485 |
| ARHGEF17 | -1.16027 | 12.80154 | -2.72779 | 0.008631 | 0.034903 | -3.22527 |
| RPL32P1 | 1.31914 | 12.99078 | 2.726487 | 0.008661 | 0.035005 | -3.22839 |
| MYH7 | -1.16317 | 13.7331 | -2.72371 | 0.008725 | 0.035241 | -3.23505 |
| EDA | 1.094817 | 12.9637 | 2.723173 | 0.008737 | 0.035281 | -3.23633 |
| KLRD1 | 1.020092 | 13.18602 | 2.722782 | 0.008746 | 0.035313 | -3.23727 |
| FAM90A24P | 1.031115 | 13.6007 | 2.720484 | 0.008799 | 0.035481 | -3.24277 |
| CAP2P1 | 1.007597 | 13.35496 | 2.718127 | 0.008854 | 0.035658 | -3.2484 |
| OOEP | -1.12727 | 13.19634 | -2.7181 | 0.008855 | 0.035658 | -3.24847 |
| SNRPCP3 | 1.246319 | 12.99874 | 2.717791 | 0.008862 | 0.035676 | -3.24921 |
| IGFBP6 | 1.181585 | 12.87435 | 2.717757 | 0.008863 | 0.035676 | -3.24929 |
| SCTR | -1.06398 | 13.28228 | -2.71538 | 0.008919 | 0.035858 | -3.25497 |
| UNC13C | -1.16819 | 12.94244 | -2.71452 | 0.008939 | 0.035929 | -3.25701 |
| PPBP | -1.05373 | 12.82182 | -2.71395 | 0.008953 | 0.03598 | -3.25839 |
| OACYLP | -1.15599 | 13.66274 | -2.71329 | 0.008969 | 0.036033 | -3.25997 |
| SAMD13 | 1.137642 | 12.99783 | 2.713218 | 0.00897 | 0.036035 | -3.26013 |
| MESP2 | -1.01229 | 13.19139 | -2.71306 | 0.008974 | 0.036045 | -3.26051 |
| AC234301.1 | -2.90375 | 11.2581 | -2.71229 | 0.008992 | 0.036099 | -3.26234 |
| COL4A1 | -1.6404 | 12.76118 | -2.71163 | 0.009008 | 0.036157 | -3.26393 |
| FCRL4 | -1.30038 | 13.21538 | -2.71151 | 0.009011 | 0.036163 | -3.2642 |
| S100A7 | -1.40588 | 13.19516 | -2.71083 | 0.009027 | 0.03621 | -3.26583 |
| SYT1 | -1.18389 | 13.02115 | -2.7107 | 0.00903 | 0.036215 | -3.26614 |
| IGHV3-16 | -1.37197 | 12.82742 | -2.71034 | 0.009039 | 0.036237 | -3.26699 |
| DNAAF3 | -1.62965 | 12.09563 | -2.70842 | 0.009085 | 0.036397 | -3.27158 |
| AL020997.1 | -1.02058 | 13.52473 | -2.7077 | 0.009102 | 0.036452 | -3.27329 |
| BEX1 | -1.58953 | 11.14193 | -2.70738 | 0.00911 | 0.036469 | -3.27407 |
| F10 | 1.046616 | 13.39799 | 2.706324 | 0.009135 | 0.036546 | -3.27658 |
| ITGA9 | -1.2686 | 12.38261 | -2.70572 | 0.009149 | 0.036594 | -3.27801 |
| TMEM178A | 1.052504 | 13.24143 | 2.704659 | 0.009175 | 0.036682 | -3.28054 |
| OR52K2 | -1.31812 | 13.1474 | -2.70446 | 0.00918 | 0.036692 | -3.28101 |
| ALPK2 | 1.305628 | 12.48443 | 2.703637 | 0.0092 | 0.036762 | -3.28298 |
| PRKY | 1.70275 | 12.81538 | 2.701863 | 0.009243 | 0.036905 | -3.2872 |
| KCNS2 | -1.08654 | 13.53516 | -2.69981 | 0.009293 | 0.037056 | -3.29208 |
| NDUFV2P1 | -1.37121 | 12.99316 | -2.69919 | 0.009309 | 0.037103 | -3.29357 |
| IGHJ2P | -1.17426 | 13.27906 | -2.69704 | 0.009361 | 0.037283 | -3.29866 |
| PSG2 | -1.51314 | 11.75201 | -2.69652 | 0.009374 | 0.037315 | -3.29992 |
| Z97985.1 | 1.015689 | 13.75753 | 2.695163 | 0.009408 | 0.037438 | -3.30313 |
| AC127032.2 | 1.998039 | 11.632 | 2.691669 | 0.009495 | 0.03769 | -3.31143 |
| LHFPL6 | -1.27578 | 12.85956 | -2.69138 | 0.009502 | 0.037698 | -3.3121 |
| MTND4P23 | -1.22957 | 13.16843 | -2.69102 | 0.009511 | 0.037723 | -3.31297 |
| F8A3 | -3.23923 | 11.31656 | -2.69093 | 0.009513 | 0.037723 | -3.31318 |
| AL139415.1 | 1.341187 | 13.0411 | 2.690363 | 0.009527 | 0.037757 | -3.31453 |
| NSUN7 | -1.23201 | 13.05057 | -2.68798 | 0.009587 | 0.037957 | -3.32017 |
| BANF1P1 | -1.18747 | 13.57558 | -2.68631 | 0.009629 | 0.0381 | -3.32414 |
| OR7E140P | -1.32344 | 12.98687 | -2.68514 | 0.009659 | 0.038202 | -3.32691 |
| CEACAM8 | -2.01674 | 11.12855 | -2.68466 | 0.009671 | 0.038239 | -3.32804 |
| RPL15P3 | 1.425668 | 12.84174 | 2.682029 | 0.009738 | 0.038394 | -3.33427 |
| IGHJ1P | -1.50659 | 12.70599 | -2.68184 | 0.009743 | 0.038408 | -3.33473 |
| KLHL33 | 1.332301 | 12.83752 | 2.681371 | 0.009755 | 0.03845 | -3.33583 |
| DEFB1 | -1.70919 | 12.17395 | -2.67965 | 0.009799 | 0.03859 | -3.33991 |
| ORM2 | -1.08717 | 12.63288 | -2.6783 | 0.009834 | 0.038701 | -3.3431 |
| FNDC4 | -1.31208 | 12.37132 | -2.67819 | 0.009837 | 0.038702 | -3.34335 |
| ORM1 | -1.74616 | 11.66876 | -2.67405 | 0.009944 | 0.039028 | -3.35314 |
| TCF3P1 | -1.13893 | 13.25384 | -2.67368 | 0.009954 | 0.039056 | -3.35402 |
| FAT4 | 1.032378 | 13.07474 | 2.672161 | 0.009993 | 0.039187 | -3.3576 |
| MTDHP1 | -1.10019 | 13.14329 | -2.66996 | 0.010051 | 0.039371 | -3.36279 |
| PLSCR2 | -1.24599 | 13.25288 | -2.6694 | 0.010066 | 0.039424 | -3.36411 |
| AL360181.3 | 1.789787 | 12.65478 | 2.667169 | 0.010125 | 0.039622 | -3.36937 |
| AC020765.4 | -1.17112 | 13.0704 | -2.66689 | 0.010132 | 0.03963 | -3.37004 |
| AZU1 | -2.14498 | 10.87499 | -2.6667 | 0.010137 | 0.039636 | -3.37047 |
| RPL12P28 | 1.483184 | 12.86218 | 2.665257 | 0.010176 | 0.039745 | -3.37388 |
| AC006116.2 | 1.142251 | 12.89677 | 2.663944 | 0.010211 | 0.039846 | -3.37697 |
| GUCY2F | -1.02013 | 13.83205 | -2.66232 | 0.010254 | 0.039985 | -3.3808 |
| AHCTF1P1 | 1.93655 | 12.31209 | 2.661286 | 0.010282 | 0.040077 | -3.38322 |
| NKAIN1 | -1.33057 | 12.98231 | -2.66084 | 0.010294 | 0.040119 | -3.38427 |
| FAM218A | 1.007578 | 13.13553 | 2.658919 | 0.010346 | 0.040284 | -3.38879 |
| PROS1 | -1.12165 | 12.70653 | -2.65816 | 0.010366 | 0.040333 | -3.39058 |
| NLRP14 | -1.27908 | 13.15656 | -2.65782 | 0.010375 | 0.040358 | -3.39138 |
| FOXD4 | -1.1376 | 13.11241 | -2.65694 | 0.010399 | 0.040415 | -3.39345 |
| AC093909.1 | -1.79513 | 12.5507 | -2.65655 | 0.01041 | 0.040443 | -3.39436 |
| PCDHB19P | -1.16863 | 13.27875 | -2.65652 | 0.010411 | 0.040443 | -3.39443 |
| ART4 | -1.26396 | 10.30809 | -2.65359 | 0.010491 | 0.040707 | -3.40132 |
| MYBPH | 1.590196 | 12.71203 | 2.653501 | 0.010493 | 0.040711 | -3.40152 |
| GUCY2EP | -1.22682 | 13.28972 | -2.65289 | 0.01051 | 0.040765 | -3.40296 |
| PRY2 | 1.506264 | 12.67898 | 2.650509 | 0.010575 | 0.040966 | -3.40854 |
| ANAPC1P1 | 1.14369 | 12.92555 | 2.648965 | 0.010618 | 0.041095 | -3.41216 |
| CBX1P2 | -1.24924 | 13.22792 | -2.6419 | 0.010815 | 0.041729 | -3.4287 |
| OR52L2P | -1.37173 | 13.11753 | -2.63958 | 0.01088 | 0.041949 | -3.43413 |
| DEGS2 | 1.16166 | 12.92887 | 2.639244 | 0.01089 | 0.041981 | -3.43492 |
| CEACAM6 | -2.05621 | 10.98027 | -2.63905 | 0.010895 | 0.041992 | -3.43539 |
| MEOX1 | 1.25474 | 12.80656 | 2.638271 | 0.010917 | 0.042044 | -3.4372 |
| AL512625.2 | -1.0021 | 13.20877 | -2.63782 | 0.01093 | 0.042082 | -3.43826 |
| AL109827.1 | 1.435524 | 11.66408 | 2.637717 | 0.010933 | 0.042082 | -3.43849 |
| KNDC1 | 1.642363 | 11.67497 | 2.636401 | 0.010971 | 0.042186 | -3.44157 |
| HMCN1 | -1.13289 | 13.19258 | -2.63483 | 0.011016 | 0.04233 | -3.44524 |
| IGLVI-56 | -1.32799 | 12.87421 | -2.63364 | 0.01105 | 0.042434 | -3.44802 |
| MT3 | 1.216259 | 13.41754 | 2.633102 | 0.011065 | 0.042482 | -3.44927 |
| AC142381.1 | -1.56043 | 12.56104 | -2.63293 | 0.01107 | 0.042491 | -3.44968 |
| ZAR1L | -1.02109 | 13.48417 | -2.63236 | 0.011086 | 0.042538 | -3.45101 |
| DEFA8P | -2.40596 | 10.89571 | -2.63166 | 0.011107 | 0.042565 | -3.45264 |
| AC136616.3 | -1.9963 | 10.70868 | -2.63112 | 0.011122 | 0.042593 | -3.4539 |
| CDH19 | -1.11407 | 13.27798 | -2.63095 | 0.011127 | 0.042601 | -3.45428 |
| AL353616.1 | -1.2115 | 13.31495 | -2.63085 | 0.01113 | 0.042607 | -3.45453 |
| FBXL13 | -1.07893 | 13.36246 | -2.63013 | 0.011151 | 0.042676 | -3.4562 |
| CTAGE3P | -1.02277 | 13.60132 | -2.62837 | 0.011202 | 0.042822 | -3.46031 |
| AC114781.3 | -1.22722 | 12.71957 | -2.62767 | 0.011222 | 0.042884 | -3.46194 |
| TVP23C-CDRT4 | -2.07037 | 12.0804 | -2.62623 | 0.011264 | 0.043023 | -3.4653 |
| IFI44 | -1.51167 | 12.90667 | -2.62538 | 0.011289 | 0.043085 | -3.46727 |
| IGHJ6 | -4.41039 | 8.347881 | -2.6249 | 0.011303 | 0.043121 | -3.46838 |
| AC134669.1 | -1.15543 | 13.18223 | -2.62417 | 0.011324 | 0.043193 | -3.47009 |
| RPS18P1 | -1.01977 | 12.88143 | -2.62296 | 0.01136 | 0.043285 | -3.47291 |
| DEFA4 | -2.19364 | 10.89702 | -2.62223 | 0.011381 | 0.04336 | -3.47459 |
| GPR26 | -1.46282 | 13.03923 | -2.62206 | 0.011387 | 0.043364 | -3.47501 |
| MRC2 | 1.142887 | 11.61111 | 2.619976 | 0.011448 | 0.043549 | -3.47985 |
| BX679664.3 | 1.760094 | 11.40378 | 2.617653 | 0.011517 | 0.043749 | -3.48524 |
| CITED1 | -1.15637 | 13.30411 | -2.61643 | 0.011554 | 0.043856 | -3.48808 |
| CAMP | -1.52045 | 11.81423 | -2.61585 | 0.011571 | 0.043911 | -3.48942 |
| AC022217.2 | -1.37207 | 12.91382 | -2.61411 | 0.011623 | 0.044047 | -3.49348 |
| CHI3L1 | 1.162227 | 12.68834 | 2.614044 | 0.011625 | 0.044047 | -3.49363 |
| ANKRD20A1 | -1.5602 | 12.39133 | -2.61403 | 0.011626 | 0.044047 | -3.49367 |
| CR381653.2 | 1.354972 | 12.64658 | 2.612901 | 0.01166 | 0.044155 | -3.49628 |
| CR769776.2 | -1.03775 | 12.64415 | -2.61288 | 0.01166 | 0.044155 | -3.49633 |
| AP001372.4 | 1.09951 | 13.3049 | 2.612836 | 0.011661 | 0.044155 | -3.49643 |
| RPS12P24 | -1.34528 | 12.89756 | -2.60933 | 0.011768 | 0.044461 | -3.50455 |
| AC104996.3 | -1.03582 | 13.43344 | -2.60805 | 0.011807 | 0.044581 | -3.50752 |
| SPATC1L | 1.157827 | 13.00611 | 2.606751 | 0.011846 | 0.044669 | -3.51053 |
| LYPD6B | 1.270143 | 12.84701 | 2.605397 | 0.011888 | 0.044803 | -3.51367 |
| MTCO3P23 | -1.03018 | 13.71293 | -2.60414 | 0.011926 | 0.044937 | -3.51657 |
| IGHV7-40 | -1.868 | 10.90006 | -2.60319 | 0.011956 | 0.045026 | -3.51878 |
| CPLX1 | 1.074873 | 13.14673 | 2.602945 | 0.011963 | 0.045037 | -3.51934 |
| GLOD5 | -1.23962 | 12.49435 | -2.60289 | 0.011965 | 0.045037 | -3.51948 |
| ACKR1 | 2.34588 | 10.86633 | 2.602762 | 0.011969 | 0.045041 | -3.51977 |
| TULP1 | -1.25438 | 13.15252 | -2.60088 | 0.012027 | 0.045216 | -3.52412 |
| AC011503.3 | 1.098892 | 13.21766 | 2.600664 | 0.012034 | 0.045229 | -3.52462 |
| IL4I1 | 1.033806 | 13.08103 | 2.598964 | 0.012087 | 0.045378 | -3.52855 |
| COX5BP6 | 1.210444 | 13.07118 | 2.5983 | 0.012107 | 0.045444 | -3.53008 |
| ARPIN | 1.123587 | 12.87692 | 2.594789 | 0.012217 | 0.045771 | -3.53819 |
| HBG2 | 2.414354 | 9.791607 | 2.594591 | 0.012223 | 0.045783 | -3.53864 |
| TAC3 | 1.054504 | 12.90722 | 2.594159 | 0.012237 | 0.045812 | -3.53964 |
| AC023050.4 | -1.05158 | 13.61121 | -2.59356 | 0.012256 | 0.045876 | -3.54103 |
| EYA2 | -1.4876 | 13.0201 | -2.59201 | 0.012305 | 0.04603 | -3.54459 |
| AC007318.3 | -1.12665 | 13.42511 | -2.59086 | 0.012341 | 0.046144 | -3.54724 |
| ARHGEF33 | 1.081946 | 13.21794 | 2.590496 | 0.012353 | 0.046176 | -3.54809 |
| SLC22A3 | 1.148682 | 13.14812 | 2.589111 | 0.012397 | 0.046324 | -3.55128 |
| ARHGEF15 | -1.09255 | 12.97535 | -2.58886 | 0.012405 | 0.046348 | -3.55187 |
| SLC29A1 | 1.137607 | 12.55495 | 2.588259 | 0.012424 | 0.046402 | -3.55324 |
| AC244260.1 | -1.89787 | 12.2742 | -2.58744 | 0.01245 | 0.046477 | -3.55512 |
| AL049714.1 | -1.14227 | 13.29126 | -2.58665 | 0.012476 | 0.046554 | -3.55695 |
| ZBED2 | 1.015193 | 13.13418 | 2.585996 | 0.012497 | 0.046615 | -3.55845 |
| ZP2 | -1.00208 | 13.56866 | -2.58592 | 0.012499 | 0.046619 | -3.55863 |
| RSAD2 | -1.95127 | 12.19906 | -2.58548 | 0.012513 | 0.046665 | -3.55963 |
| ENPP7P3 | -1.13932 | 13.29761 | -2.58505 | 0.012527 | 0.046711 | -3.56063 |
| PHLDA2 | -1.36806 | 12.82136 | -2.5849 | 0.012532 | 0.046724 | -3.56099 |
| AL137792.2 | -1.06435 | 13.33445 | -2.58445 | 0.012546 | 0.046766 | -3.562 |
| ST13P5 | 1.015519 | 13.08715 | 2.584134 | 0.012557 | 0.046787 | -3.56274 |
| CPLX3 | -1.02448 | 13.15477 | -2.57873 | 0.012732 | 0.047306 | -3.57516 |
| IRX3 | -1.65236 | 12.2923 | -2.57833 | 0.012745 | 0.047348 | -3.57607 |
| RCAN2 | 1.36566 | 12.45613 | 2.576437 | 0.012807 | 0.047538 | -3.58042 |
| CKM | -1.11332 | 13.3176 | -2.57563 | 0.012833 | 0.047607 | -3.58228 |
| RPL23AP2 | 1.261082 | 12.95606 | 2.575049 | 0.012853 | 0.047666 | -3.58361 |
| AP002472.1 | 1.796367 | 11.08244 | 2.572627 | 0.012933 | 0.047892 | -3.58916 |
| GK3P | -1.33249 | 12.97574 | -2.57129 | 0.012977 | 0.048033 | -3.59223 |
| AL354714.2 | 1.091067 | 13.38538 | 2.570035 | 0.013019 | 0.04817 | -3.5951 |
| TEAD4 | -1.08041 | 12.8119 | -2.56983 | 0.013025 | 0.048183 | -3.59557 |
| IGHV3-22 | -1.60418 | 12.27612 | -2.56875 | 0.013062 | 0.048281 | -3.59805 |
| TM4SF18 | -1.14445 | 13.20239 | -2.56855 | 0.013068 | 0.048299 | -3.5985 |
| TNN | -1.09517 | 13.3669 | -2.56844 | 0.013072 | 0.048307 | -3.59875 |
| SAP18P3 | -1.01788 | 13.24771 | -2.56775 | 0.013095 | 0.048387 | -3.60033 |
| NRXN2 | 1.035457 | 13.04518 | 2.567587 | 0.0131 | 0.048401 | -3.60071 |
| AC093525.2 | -1.69431 | 12.44734 | -2.56648 | 0.013138 | 0.048503 | -3.60325 |
| KIAA1143P1 | -1.81068 | 11.60997 | -2.56619 | 0.013147 | 0.048533 | -3.60391 |
| CHL1 | 1.377239 | 12.85857 | 2.564987 | 0.013188 | 0.048659 | -3.60666 |
| TPBG | 1.369926 | 12.90863 | 2.562978 | 0.013256 | 0.048855 | -3.61125 |
| AC023825.1 | -1.34932 | 12.74966 | -2.56289 | 0.013259 | 0.04886 | -3.61145 |
| FAM163A | -1.12154 | 13.3507 | -2.56273 | 0.013264 | 0.048874 | -3.61181 |
| LGALS2 | 1.738716 | 12.34866 | 2.561518 | 0.013305 | 0.048968 | -3.61459 |
| ROBO2 | -1.17355 | 13.26601 | -2.5615 | 0.013306 | 0.048968 | -3.61462 |
| MT1H | -1.15654 | 12.27314 | -2.56017 | 0.013351 | 0.049111 | -3.61766 |
| TRBV28 | 2.579979 | 11.2869 | 2.560042 | 0.013356 | 0.049112 | -3.61796 |
| LCE5A | -1.59558 | 11.29593 | -2.55985 | 0.013362 | 0.049122 | -3.61841 |
| NPIPB7 | -1.08174 | 13.18827 | -2.55957 | 0.013372 | 0.049148 | -3.61905 |
| IGKV1D-35 | -1.46424 | 12.8583 | -2.55918 | 0.013385 | 0.049161 | -3.61993 |
| AC133065.2 | 1.219242 | 13.07221 | 2.558907 | 0.013394 | 0.049184 | -3.62055 |
| AL355076.3 | 1.151359 | 12.9058 | 2.55882 | 0.013397 | 0.049185 | -3.62075 |
| AL133268.1 | -1.42988 | 12.88105 | -2.55875 | 0.0134 | 0.049186 | -3.6209 |
| ADGRF5 | -1.42163 | 12.59677 | -2.55778 | 0.013433 | 0.049275 | -3.62313 |
| TUBB8P1 | 1.061429 | 13.45002 | 2.557767 | 0.013433 | 0.049275 | -3.62315 |
| NPM1P26 | -1.07733 | 12.85713 | -2.55768 | 0.013436 | 0.04928 | -3.62335 |
| GGTA1P | 1.001027 | 12.45917 | 2.556848 | 0.013465 | 0.049343 | -3.62525 |
| RBBP4P2 | -1.13486 | 13.06272 | -2.55569 | 0.013505 | 0.049453 | -3.6279 |
| AL136295.4 | -1.53545 | 12.99773 | -2.55557 | 0.013509 | 0.049462 | -3.62816 |
| CHGA | -1.20694 | 13.23838 | -2.55525 | 0.01352 | 0.049485 | -3.6289 |
| CEACAM7 | -1.18388 | 13.3481 | -2.55494 | 0.013531 | 0.049518 | -3.62961 |
| CRISP3 | -1.74593 | 11.61538 | -2.554 | 0.013563 | 0.049598 | -3.63174 |
| NLGN1 | -1.28258 | 12.97987 | -2.55397 | 0.013564 | 0.049598 | -3.63182 |
| AP000790.1 | -1.15102 | 13.05823 | -2.55234 | 0.013621 | 0.049769 | -3.63554 |
| AC123912.3 | -1.52071 | 12.70561 | -2.55186 | 0.013637 | 0.049817 | -3.63662 |
| IFIT3 | -1.40682 | 12.88952 | -2.55144 | 0.013652 | 0.049852 | -3.63758 |
