# Supplementary material for: Bioinformatics and System Biology Approach to Identify the Influences of COVID-19 on Rheumatoid Arthritis
Source: Front Immunol. 2022 Apr 7;13:860676. doi: 10.3389/fimmu.2022.860676 (PMC9021444; doi:10.3389/fimmu.2022.860676)
Supplement: Supplementary file 4 [file Table_3.docx]

Table S3. Common genes of RA and COVID-19 (n=103).

| NCAPG | RPL18 | HPN | EVL | SLC4A7 |
| --- | --- | --- | --- | --- |
| CLSPN | HLA-DPB1 | FFAR3 | DNMT3B | BDKRB1 |
| FOXM1 | QPCT | ARG1 | TMEM132C | GNLY |
| SPC25 | MMP9 | GPR18 | PROK2 | HBE1 |
| EEF1A1 | HLA-DRA | AP1S2 | ALOX5AP | BANK1 |
| RPS3 | HLA-DPA1 | LDHB | IL7R | CCL5 |
| KLRB1 | FCGR1A | TNFAIP6 | SLC22A1 | RAI14 |
| RPL13A | TMC5 | ZNF549 | OLIG2 | C2 |
| CD1C | HLA-DMA | UPB1 | TPST1 | CXCL9 |
| CCNE2 | FGFR2 | LCK | REG4 | MAOA |
| MND1 | FAM102A | B3GNT8 | CDKN1C | DOC2B |
| RPL13 | NELL2 | STAT4 | OSBPL6 | MARCO |
| ID3 | NEK6 | MYL9 | NNMT | SHOX2 |
| RPSA | CYP2U1 | ALDH1A1 | CCR7 | TGFBI |
| RPL3 | S100A12 | NQO2 | DOCK3 | IL1R2 |
| TXNDC5 | CD74 | TLR2 | ROPN1L | CLGN |
| MAOB | TBC1D4 | C1QTNF7 | CLEC4E | GZMH |
| GZMK | ANXA3 | HIP1 | KRT2 | SNX9 |
| S100A9 | KLRK1 | KL | TM4SF20 | CLC |
| PRSS35 | CD3E | IL32 | CTSW | PPBP |
| DEFA4 | CAMP | IFIT3 |  |  |
