# Supplementary material for: Bioinformatics and System Biology Approach to Identify the Influences of COVID-19 on Rheumatoid Arthritis
Source: Front Immunol. 2022 Apr 7;13:860676. doi: 10.3389/fimmu.2022.860676 (PMC9021444; doi:10.3389/fimmu.2022.860676)
Supplement: Supplementary file 5 [file Table_4.docx]

Table S4. GO pathway.

| ONTOLOGY | ID | Description | Gene Ratio | | Bg Ratio | P value | P adjust | Q value | Gene ID | Count |
| --- | --- | --- | --- | --- | --- | --- | --- | --- | --- | --- |
| CC | GO:0042613 | MHC class II protein complex | | 5/101 | 16/19520 | 1.40E-08 | 2.34E-06 | 1.74E-06 | HLA-DPB1/HLA-DRA/HLA-DPA1/HLA-DMA/CD74 | 5 |
| CC | GO:0042611 | MHC protein complex | | 5/101 | 25/19520 | 1.64E-07 | 1.37E-05 | 1.02E-05 | HLA-DPB1/HLA-DRA/HLA-DPA1/HLA-DMA/CD74 | 5 |
| CC | GO:0030669 | clathrin-coated endocytic vesicle membrane | | 5/101 | 39/19520 | 1.68E-06 | 8.37E-05 | 6.23E-05 | HLA-DPB1/HLA-DRA/HLA-DPA1/FCGR1A/CD74 | 5 |
| CC | GO:0030666 | endocytic vesicle membrane | | 8/101 | 163/19520 | 2.08E-06 | 8.37E-05 | 6.23E-05 | HLA-DPB1/HLA-DRA/HLA-DPA1/FCGR1A/CD74/ANXA3/TLR2/MARCO | 8 |
| CC | GO:0030665 | clathrin-coated vesicle membrane | | 7/101 | 117/19520 | 2.51E-06 | 8.37E-05 | 6.23E-05 | HLA-DPB1/HLA-DRA/HLA-DPA1/FCGR1A/CD74/HIP1/IL7R | 7 |
| CC | GO:0034774 | secretory granule lumen | | 10/101 | 322/19520 | 6.61E-06 | 0.000144 | 0.000107 | EEF1A1/TXNDC5/S100A9/QPCT/S100A12/ARG1/CTSW/PPBP/DEFA4/CAMP | 10 |
| CC | GO:0030136 | clathrin-coated vesicle | | 8/101 | 192/19520 | 7.01E-06 | 0.000144 | 0.000107 | HLA-DPB1/HLA-DRA/HLA-DPA1/FCGR1A/CD74/HIP1/IL7R/SNX9 | 8 |
| CC | GO:0060205 | cytoplasmic vesicle lumen | | 10/101 | 326/19520 | 7.37E-06 | 0.000144 | 0.000107 | EEF1A1/TXNDC5/S100A9/QPCT/S100A12/ARG1/CTSW/PPBP/DEFA4/CAMP | 10 |
| CC | GO:0031983 | vesicle lumen | | 10/101 | 328/19520 | 7.78E-06 | 0.000144 | 0.000107 | EEF1A1/TXNDC5/S100A9/QPCT/S100A12/ARG1/CTSW/PPBP/DEFA4/CAMP | 10 |
| CC | GO:1904724 | tertiary granule lumen | | 5/101 | 55/19520 | 9.51E-06 | 0.000159 | 0.000118 | QPCT/MMP9/TNFAIP6/PPBP/CAMP | 5 |
| CC | GO:0045334 | clathrin-coated endocytic vesicle | | 5/101 | 57/19520 | 1.14E-05 | 0.000172 | 0.000128 | HLA-DPB1/HLA-DRA/HLA-DPA1/FCGR1A/CD74 | 5 |
| CC | GO:0071556 | integral component of lumenal side of endoplasmic reticulum membrane | | 4/101 | 29/19520 | 1.45E-05 | 0.000186 | 0.000139 | HLA-DPB1/HLA-DRA/HLA-DPA1/CD74 | 4 |
| CC | GO:0098553 | lumenal side of endoplasmic reticulum membrane | | 4/101 | 29/19520 | 1.45E-05 | 0.000186 | 0.000139 | HLA-DPB1/HLA-DRA/HLA-DPA1/CD74 | 4 |
| CC | GO:0022626 | cytosolic ribosome | | 6/101 | 107/19520 | 1.96E-05 | 0.000233 | 0.000174 | RPS3/RPL13A/RPL13/RPSA/RPL3/RPL18 | 6 |
| CC | GO:0030139 | endocytic vesicle | | 9/101 | 307/19520 | 3.08E-05 | 0.000343 | 0.000255 | HLA-DPB1/HLA-DRA/HLA-DPA1/FCGR1A/CD74/ANXA3/TLR2/GNLY/MARCO | 9 |
| CC | GO:0098576 | lumenal side of membrane | | 4/101 | 36/19520 | 3.50E-05 | 0.000365 | 0.000272 | HLA-DPB1/HLA-DRA/HLA-DPA1/CD74 | 4 |
| CC | GO:0030662 | coated vesicle membrane | | 7/101 | 182/19520 | 4.49E-05 | 0.000441 | 0.000328 | HLA-DPB1/HLA-DRA/HLA-DPA1/FCGR1A/CD74/HIP1/IL7R | 7 |
| CC | GO:0032588 | trans-Golgi network membrane | | 5/101 | 95/19520 | 0.000135 | 0.001248 | 0.000928 | HLA-DPB1/HLA-DRA/HLA-DPA1/CD74/AP1S2 | 5 |
| CC | GO:0030135 | coated vesicle | | 8/101 | 295/19520 | 0.000148 | 0.001303 | 0.000969 | HLA-DPB1/HLA-DRA/HLA-DPA1/FCGR1A/CD74/HIP1/IL7R/SNX9 | 8 |
| CC | GO:0022625 | cytosolic large ribosomal subunit | | 4/101 | 57/19520 | 0.000216 | 0.001804 | 0.001342 | RPL13A/RPL13/RPL3/RPL18 | 4 |
| CC | GO:0012507 | ER to Golgi transport vesicle membrane | | 4/101 | 62/19520 | 0.000299 | 0.002271 | 0.001689 | HLA-DPB1/HLA-DRA/HLA-DPA1/CD74 | 4 |
| CC | GO:0035580 | specific granule lumen | | 4/101 | 62/19520 | 0.000299 | 0.002271 | 0.001689 | QPCT/ARG1/DEFA4/CAMP | 4 |
| CC | GO:0005802 | trans-Golgi network | | 7/101 | 251/19520 | 0.000329 | 0.002391 | 0.001778 | HLA-DPB1/HLA-DRA/HLA-DPA1/CD74/AP1S2/TGFBI/SNX9 | 7 |
| CC | GO:0044391 | ribosomal subunit | | 6/101 | 187/19520 | 0.000426 | 0.002961 | 0.002202 | RPS3/RPL13A/RPL13/RPSA/RPL3/RPL18 | 6 |
| CC | GO:0001772 | immunological synapse | | 3/101 | 41/19520 | 0.001243 | 0.0083 | 0.006174 | HLA-DRA/CD3E/LCK | 3 |
| CC | GO:0030134 | COPII-coated ER to Golgi transport vesicle | | 4/101 | 94/19520 | 0.001441 | 0.009043 | 0.006726 | HLA-DPB1/HLA-DRA/HLA-DPA1/CD74 | 4 |
| CC | GO:0042581 | specific granule | | 5/101 | 160/19520 | 0.001462 | 0.009043 | 0.006726 | QPCT/ANXA3/ARG1/DEFA4/CAMP | 5 |
| CC | GO:0070820 | tertiary granule | | 5/101 | 164/19520 | 0.00163 | 0.009537 | 0.007093 | QPCT/MMP9/TNFAIP6/PPBP/CAMP | 5 |
| CC | GO:0005840 | ribosome | | 6/101 | 243/19520 | 0.001656 | 0.009537 | 0.007093 | RPS3/RPL13A/RPL13/RPSA/RPL3/RPL18 | 6 |
| CC | GO:0015934 | large ribosomal subunit | | 4/101 | 115/19520 | 0.003009 | 0.016749 | 0.012458 | RPL13A/RPL13/RPL3/RPL18 | 4 |
| CC | GO:0005765 | lysosomal membrane | | 7/101 | 378/19520 | 0.003499 | 0.018262 | 0.013583 | EEF1A1/HLA-DPB1/HLA-DRA/HLA-DPA1/HLA-DMA/CD74/AP1S2 | 7 |
| CC | GO:0098852 | lytic vacuole membrane | | 7/101 | 378/19520 | 0.003499 | 0.018262 | 0.013583 | EEF1A1/HLA-DPB1/HLA-DRA/HLA-DPA1/HLA-DMA/CD74/AP1S2 | 7 |
| CC | GO:1904813 | ficolin-1-rich granule lumen | | 4/101 | 124/19520 | 0.003941 | 0.019945 | 0.014835 | EEF1A1/QPCT/MMP9/TNFAIP6 | 4 |
| CC | GO:0009897 | external side of plasma membrane | | 7/101 | 402/19520 | 0.004888 | 0.024009 | 0.017858 | CD1C/CD74/KLRK1/CD3E/IL7R/CCR7/CXCL9 | 7 |
| CC | GO:0005774 | vacuolar membrane | | 7/101 | 431/19520 | 0.00708 | 0.033782 | 0.025126 | EEF1A1/HLA-DPB1/HLA-DRA/HLA-DPA1/HLA-DMA/CD74/AP1S2 | 7 |
| CC | GO:0030176 | integral component of endoplasmic reticulum membrane | | 4/101 | 157/19520 | 0.008995 | 0.041727 | 0.031036 | HLA-DPB1/HLA-DRA/HLA-DPA1/CD74 | 4 |
| CC | GO:0031227 | intrinsic component of endoplasmic reticulum membrane | | 4/101 | 165/19520 | 0.010656 | 0.048098 | 0.035774 | HLA-DPB1/HLA-DRA/HLA-DPA1/CD74 | 4 |
| CC | GO:0031234 | extrinsic component of cytoplasmic side of plasma membrane | | 3/101 | 100/19520 | 0.015136 | 0.066521 | 0.049477 | LCK/HIP1/SNX9 | 3 |
| CC | GO:0101002 | ficolin-1-rich granule | | 4/101 | 185/19520 | 0.015643 | 0.066983 | 0.04982 | EEF1A1/QPCT/MMP9/TNFAIP6 | 4 |
