# Supplementary material for: Bioinformatics and System Biology Approach to Identify the Influences of COVID-19 on Rheumatoid Arthritis
Source: Front Immunol. 2022 Apr 7;13:860676. doi: 10.3389/fimmu.2022.860676 (PMC9021444; doi:10.3389/fimmu.2022.860676)
Supplement: Supplementary file 6 [file Table_5.docx]

Table S5. KEGG pathway.

| ID | Description | Gene Ratio | Bg Ratio | P value | P adjust | Q value | Gene ID | Count |
| --- | --- | --- | --- | --- | --- | --- | --- | --- |
| hsa04640 | Hematopoietic cell lineage | 9/63 | 99/8112 | 5.71E-08 | 8.62E-06 | 6.55E-06 | CD1C/HLA-DPB1/HLA-DRA/HLA-DPA1/FCGR1A/HLA-DMA/CD3E/IL7R/IL1R2 | 9 |
| hsa05150 | Staphylococcus aureus infection | 8/63 | 96/8112 | 6.50E-07 | 4.91E-05 | 3.73E-05 | HLA-DPB1/HLA-DRA/HLA-DPA1/FCGR1A/HLA-DMA/C2/DEFA4/CAMP | 8 |
| hsa05140 | Leishmaniasis | 7/63 | 77/8112 | 1.90E-06 | 9.57E-05 | 7.28E-05 | EEF1A1/HLA-DPB1/HLA-DRA/HLA-DPA1/FCGR1A/HLA-DMA/TLR2 | 7 |
| hsa04658 | Th1 and Th2 cell differentiation | 7/63 | 92/8112 | 6.33E-06 | 2.39E-04 | 0.000181 | HLA-DPB1/HLA-DRA/HLA-DPA1/HLA-DMA/CD3E/LCK/STAT4 | 7 |
| hsa05152 | Tuberculosis | 9/63 | 180/8112 | 9.01E-06 | 2.50E-04 | 0.00019 | HLA-DPB1/HLA-DRA/HLA-DPA1/FCGR1A/HLA-DMA/CD74/TLR2/CLEC4E/CAMP | 9 |
| hsa05321 | Inflammatory bowel disease | 6/63 | 65/8112 | 9.95E-06 | 2.50E-04 | 0.00019 | HLA-DPB1/HLA-DRA/HLA-DPA1/HLA-DMA/STAT4/TLR2 | 6 |
| hsa05323 | Rheumatoid arthritis | 6/63 | 93/8112 | 7.75E-05 | 1.67E-03 | 0.001271 | HLA-DPB1/HLA-DRA/HLA-DPA1/HLA-DMA/TLR2/CCL5 | 6 |
| hsa05310 | Asthma | 4/63 | 31/8112 | 8.88E-05 | 1.68E-03 | 0.001274 | HLA-DPB1/HLA-DRA/HLA-DPA1/HLA-DMA | 4 |
| hsa04145 | Phagosome | 7/63 | 152/8112 | 0.000162 | 2.68E-03 | 0.00204 | HLA-DPB1/HLA-DRA/HLA-DPA1/FCGR1A/HLA-DMA/TLR2/MARCO | 7 |
| hsa04659 | Th17 cell differentiation | 6/63 | 108/8112 | 0.000178 | 2.68E-03 | 0.00204 | HLA-DPB1/HLA-DRA/HLA-DPA1/HLA-DMA/CD3E/LCK | 6 |
| hsa05330 | Allograft rejection | 4/63 | 38/8112 | 0.0002 | 2.75E-03 | 0.002087 | HLA-DPB1/HLA-DRA/HLA-DPA1/HLA-DMA | 4 |
| hsa05166 | Human T-cell leukemia virus 1 infection | 8/63 | 222/8112 | 0.000294 | 3.31E-03 | 0.002512 | CCNE2/HLA-DPB1/HLA-DRA/HLA-DPA1/HLA-DMA/CD3E/LCK/IL1R2 | 8 |
| hsa05332 | Graft-versus-host disease | 4/63 | 42/8112 | 0.000296 | 3.31E-03 | 0.002512 | HLA-DPB1/HLA-DRA/HLA-DPA1/HLA-DMA | 4 |
| hsa04940 | Type I diabetes mellitus | 4/63 | 43/8112 | 0.000325 | 3.31E-03 | 0.002512 | HLA-DPB1/HLA-DRA/HLA-DPA1/HLA-DMA | 4 |
| hsa04612 | Antigen processing and presentation | 5/63 | 78/8112 | 0.000328 | 3.31E-03 | 0.002512 | HLA-DPB1/HLA-DRA/HLA-DPA1/HLA-DMA/CD74 | 5 |
| hsa05171 | Coronavirus disease - COVID-19 | 8/63 | 232/8112 | 0.000395 | 3.73E-03 | 0.002835 | RPS3/RPL13A/RPL13/RPSA/RPL3/RPL18/TLR2/C2 | 8 |
| hsa04672 | Intestinal immune network for IgA production | 4/63 | 49/8112 | 0.000539 | 4.79E-03 | 0.003636 | HLA-DPB1/HLA-DRA/HLA-DPA1/HLA-DMA | 4 |
| hsa05322 | Systemic lupus erythematosus | 6/63 | 136/8112 | 0.000617 | 5.17E-03 | 0.003932 | HLA-DPB1/HLA-DRA/HLA-DPA1/FCGR1A/HLA-DMA/C2 | 6 |
| hsa05320 | Autoimmune thyroid disease | 4/63 | 53/8112 | 0.000727 | 5.78E-03 | 0.004393 | HLA-DPB1/HLA-DRA/HLA-DPA1/HLA-DMA | 4 |
| hsa05169 | Epstein-Barr virus infection | 7/63 | 202/8112 | 0.000908 | 6.86E-03 | 0.005211 | CCNE2/HLA-DPB1/HLA-DRA/HLA-DPA1/HLA-DMA/CD3E/TLR2 | 7 |
| hsa05416 | Viral myocarditis | 4/63 | 60/8112 | 0.001163 | 8.36E-03 | 0.006356 | HLA-DPB1/HLA-DRA/HLA-DPA1/HLA-DMA | 4 |
| hsa03010 | Ribosome | 6/63 | 158/8112 | 0.00135 | 9.26E-03 | 0.007039 | RPS3/RPL13A/RPL13/RPSA/RPL3/RPL18 | 6 |
| hsa05145 | Toxoplasmosis | 5/63 | 112/8112 | 0.001705 | 1.12E-02 | 0.008507 | HLA-DPB1/HLA-DRA/HLA-DPA1/HLA-DMA/TLR2 | 5 |
| hsa05340 | Primary immunodeficiency | 3/63 | 38/8112 | 0.003103 | 1.95E-02 | 0.014835 | CD3E/LCK/IL7R | 3 |
| hsa05215 | Prostate cancer | 4/63 | 97/8112 | 0.006677 | 3.81E-02 | 0.028977 | CCNE2/MMP9/FGFR2/IL1R2 | 4 |
| hsa05144 | Malaria | 3/63 | 50/8112 | 0.006749 | 3.81E-02 | 0.028977 | KLRB1/KLRK1/TLR2 | 3 |
| hsa00330 | Arginine and proline metabolism | 3/63 | 51/8112 | 0.007132 | 3.81E-02 | 0.028977 | MAOB/ARG1/MAOA | 3 |
| hsa04061 | Viral protein interaction with cytokine and cytokine receptor | 4/63 | 100/8112 | 0.007427 | 3.81E-02 | 0.028977 | CCR7/CCL5/CXCL9/PPBP | 4 |
| hsa00360 | Phenylalanine metabolism | 2/63 | 17/8112 | 0.007489 | 3.81E-02 | 0.028977 | MAOB/MAOA | 2 |
| hsa04060 | Cytokine-cytokine receptor interaction | 7/63 | 295/8112 | 0.007577 | 3.81E-02 | 0.028977 | IL32/IL7R/CCR7/CCL5/CXCL9/IL1R2/PPBP | 7 |
| hsa05146 | Amoebiasis | 4/63 | 102/8112 | 0.007957 | 3.88E-02 | 0.029451 | CD1C/ARG1/TLR2/IL1R2 | 4 |
| hsa05164 | Influenza A | 5/63 | 171/8112 | 0.010303 | 4.86E-02 | 0.036942 | HLA-DPB1/HLA-DRA/HLA-DPA1/HLA-DMA/CCL5 | 5 |
| hsa00340 | Histidine metabolism | 2/63 | 22/8112 | 0.012408 | 5.68E-02 | 0.043141 | MAOB/MAOA | 2 |
