# Supplementary material for: Bioinformatics and System Biology Approach to Identify the Influences of COVID-19 on Rheumatoid Arthritis
Source: Front Immunol. 2022 Apr 7;13:860676. doi: 10.3389/fimmu.2022.860676 (PMC9021444; doi:10.3389/fimmu.2022.860676)
Supplement: Supplementary file 7 [file Table_6.docx]

Table S6. PPI topology table.

| Shared name | Name | Betweenness Centrality | Closeness Centrality | Degree | Stress |
| --- | --- | --- | --- | --- | --- |
| ALOX5AP | ALOX5AP | 0 | 0.32642487 | 3 | 0 |
| TLR2 | TLR2 | 0.128845 | 0.459854015 | 20 | 1694 |
| S100A12 | S100A12 | 0.035083 | 0.386503067 | 11 | 550 |
| S100A9 | S100A9 | 0.025351 | 0.384146341 | 10 | 480 |
| ANXA3 | ANXA3 | 0 | 0.285067873 | 2 | 0 |
| AP1S2 | AP1S2 | 0.06298 | 0.328125 | 6 | 966 |
| CD74 | CD74 | 0.156617 | 0.428571429 | 12 | 2332 |
| TXNDC5 | TXNDC5 | 0 | 0.248031496 | 1 | 0 |
| SNX9 | SNX9 | 0 | 0.248031496 | 1 | 0 |
| HLA-DRA | HLA-DRA | 0.258535 | 0.417218543 | 10 | 3116 |
| HLA-DPA1 | HLA-DPA1 | 0.004339 | 0.344262295 | 7 | 132 |
| HLA-DPB1 | HLA-DPB1 | 0.004339 | 0.344262295 | 7 | 132 |
| ARG1 | ARG1 | 0.001183 | 0.391304348 | 7 | 24 |
| KLRK1 | KLRK1 | 0.00597 | 0.388888889 | 12 | 162 |
| CCR7 | CCR7 | 0.11684 | 0.456521739 | 18 | 1644 |
| FCGR1A | FCGR1A | 0.010822 | 0.406451613 | 10 | 290 |
| CXCL9 | CXCL9 | 0.119368 | 0.470149254 | 15 | 1864 |
| CCL5 | CCL5 | 0.153302 | 0.470149254 | 22 | 2014 |
| MMP9 | MMP9 | 0.28341 | 0.4375 | 15 | 3418 |
| BANK1 | BANK1 | 0 | 0.285067873 | 1 | 0 |
| STAT4 | STAT4 | 0.033581 | 0.396226415 | 7 | 350 |
| CAMP | CAMP | 0.101393 | 0.4375 | 9 | 1288 |
| GNLY | GNLY | 0.015724 | 0.368421053 | 9 | 362 |
| MARCO | MARCO | 0 | 0.344262295 | 3 | 0 |
| DEFA4 | DEFA4 | 0.032248 | 0.36 | 4 | 236 |
| GZMH | GZMH | 0.005235 | 0.353932584 | 6 | 148 |
| KLRB1 | KLRB1 | 0.003092 | 0.375 | 10 | 80 |
| GZMK | GZMK | 0.013997 | 0.370588235 | 10 | 354 |
| TNFAIP6 | TNFAIP6 | 0 | 0.335106383 | 2 | 0 |
| PPBP | PPBP | 0.00223 | 0.370588235 | 6 | 84 |
| IL7R | IL7R | 0.044988 | 0.39375 | 13 | 736 |
| CD3E | CD3E | 0.057824 | 0.422818792 | 14 | 1140 |
| CD1C | CD1C | 0.011293 | 0.401273885 | 11 | 268 |
| IFIT3 | IFIT3 | 0 | 0.338709677 | 2 | 0 |
| IL32 | IL32 | 0 | 0.321428571 | 1 | 0 |
| CCNE2 | CCNE2 | 0.031746 | 0.252 | 3 | 336 |
| NCAPG | NCAPG | 0.03405 | 0.255060729 | 6 | 348 |
| FOXM1 | FOXM1 | 0.203021 | 0.328125 | 6 | 2244 |
| CDKN1C | CDKN1C | 0 | 0.201923077 | 1 | 0 |
| FAM102A | FAM102A | 0.031746 | 0.318181818 | 2 | 248 |
| LCK | LCK | 0.018723 | 0.379518072 | 8 | 392 |
| TM4SF20 | TM4SF20 | 0.031746 | 0.304347826 | 2 | 312 |
| HLA-DMA | HLA-DMA | 0 | 0.321428571 | 4 | 0 |
| CLC | CLC | 0 | 0.265822785 | 1 | 0 |
| CLEC4E | CLEC4E | 0 | 0.316582915 | 1 | 0 |
| CLSPN | CLSPN | 0 | 0.25 | 2 | 0 |
| CTSW | CTSW | 0 | 0.28 | 3 | 0 |
| DNMT3B | DNMT3B | 0 | 0.25 | 2 | 0 |
| EEF1A1 | EEF1A1 | 0.200717 | 0.316582915 | 7 | 2176 |
| RPS3 | RPS3 | 0 | 0.247058824 | 6 | 0 |
| RPL13 | RPL13 | 0 | 0.247058824 | 6 | 0 |
| RPL18 | RPL18 | 0.015617 | 0.248031496 | 7 | 282 |
| RPL13A | RPL13A | 0 | 0.247058824 | 6 | 0 |
| RPSA | RPSA | 0 | 0.247058824 | 6 | 0 |
| RPL3 | RPL3 | 0.015617 | 0.248031496 | 7 | 282 |
| NELL2 | NELL2 | 0 | 0.242307692 | 1 | 0 |
| FGFR2 | FGFR2 | 1 | 1 | 2 | 2 |
| SLC4A7 | SLC4A7 | 0 | 0.666666667 | 1 | 0 |
| KL | KL | 0 | 0.666666667 | 1 | 0 |
| SPC25 | SPC25 | 0 | 0.25 | 2 | 0 |
| ID3 | ID3 | 0 | 0.283783784 | 1 | 0 |
| IL1R2 | IL1R2 | 0.00288 | 0.355932203 | 4 | 66 |
| LDHB | LDHB | 0 | 0.2 | 2 | 0 |
| MAOA | MAOA | 0 | 1 | 1 | 0 |
| MAOB | MAOB | 0 | 1 | 1 | 0 |
| PROK2 | PROK2 | 0 | 0.313432836 | 2 | 0 |
| TGFBI | TGFBI | 0 | 0.305825243 | 1 | 0 |
| MND1 | MND1 | 0 | 0.203883495 | 1 | 0 |
| NNMT | NNMT | 0 | 1 | 1 | 0 |
| RAI14 | RAI14 | 0 | 1 | 1 | 0 |
| REG4 | REG4 | 0 | 0.234200743 | 1 | 0 |
