# Supplementary material for: Bioinformatics and System Biology Approach to Identify the Influences of COVID-19 on Rheumatoid Arthritis
Source: Front Immunol. 2022 Apr 7;13:860676. doi: 10.3389/fimmu.2022.860676 (PMC9021444; doi:10.3389/fimmu.2022.860676)
Supplement: Supplementary file 8 [file Table_7.docx]

Table S7. TF-Gene topology table.

| ID | Label | Degree | Betweenness |
| --- | --- | --- | --- |
| 2296 | FOXC1 | 18 | 151.94 |
| 26064 | RAI14 | 17 | 106.61 |
| 2624 | GATA2 | 16 | 102.74 |
| 10783 | NEK6 | 15 | 88.05 |
| 9235 | IL32 | 13 | 76.91 |
| 79838 | TMC5 | 13 | 71.43 |
| 114905 | C1QTNF7 | 13 | 54.37 |
| 2263 | FGFR2 | 12 | 58.94 |
| 3725 | JUN | 12 | 56.81 |
| 7528 | YY1 | 12 | 54.32 |
| 241 | ALOX5AP | 11 | 46.95 |
| 4753 | NELL2 | 11 | 42.78 |
| 3437 | IFIT3 | 11 | 40.97 |
| 4782 | NFIC | 10 | 32.92 |
| 6722 | SRF | 10 | 31.86 |
| 167681 | PRSS35 | 9 | 74.89 |
| 717 | C2 | 9 | 36.31 |
| 5468 | PPARG | 9 | 32.31 |
| 83853 | ROPN1L | 9 | 30.4 |
| 3849 | KRT2 | 9 | 29.94 |
| 5970 | RELA | 9 | 28.05 |
| 4790 | NFKB1 | 9 | 27.68 |
| 55024 | BANK1 | 9 | 26.26 |
| 63967 | CLSPN | 9 | 25.75 |
| 79853 | TM4SF20 | 9 | 24.9 |
| 51733 | UPB1 | 8 | 21.5 |
| 7157 | TP53 | 7 | 20.53 |
| 4129 | MAOB | 7 | 18.6 |
| 3092 | HIP1 | 7 | 17.8 |
| 1236 | CCR7 | 7 | 17.59 |
| 5452 | POU2F2 | 7 | 16.77 |
| 25988 | HINFP | 7 | 16.28 |
| 3727 | JUND | 7 | 15.6 |
| 3202 | HOXA5 | 7 | 14.67 |
| 4800 | NFYA | 7 | 12.94 |
| 2300 | FOXL1 | 7 | 11.21 |
| 1521 | CTSW | 6 | 14.69 |
| 2002 | ELK1 | 6 | 13.72 |
| 7392 | USF2 | 6 | 11.43 |
| 1789 | DNMT3B | 6 | 11.37 |
| 2625 | GATA3 | 6 | 11.18 |
| 7003 | TEAD1 | 5 | 9.52 |
| 6774 | STAT3 | 5 | 9.22 |
| 6772 | STAT1 | 5 | 8.24 |
| 7020 | TFAP2A | 5 | 8.08 |
| 6720 | SREBF1 | 5 | 7.4 |
| 1869 | E2F1 | 5 | 7.13 |
| 3172 | HNF4A | 5 | 6.95 |
| 1385 | CREB1 | 4 | 6.3 |
| 2353 | FOS | 4 | 4.77 |
| 4205 | MEF2A | 4 | 2.44 |
| 51450 | PRRX2 | 1 | 0 |
