# Supplementary material for: Bioinformatics and System Biology Approach to Identify the Influences of COVID-19 on Rheumatoid Arthritis
Source: Front Immunol. 2022 Apr 7;13:860676. doi: 10.3389/fimmu.2022.860676 (PMC9021444; doi:10.3389/fimmu.2022.860676)
Supplement: Supplementary file 9 [file Table_8.docx]

Table S8. MiRNA-Gene topology table.

| ID | Label | Degree | Betweenness |
| --- | --- | --- | --- |
| MIMAT0000069 | hsa-mir-16-5p | 8 | 148.04 |
| MIMAT0000265 | hsa-mir-204-5p | 7 | 205.87 |
| MIMAT0000093 | hsa-mir-93-5p | 7 | 196.59 |
| MIMAT0000422 | hsa-mir-124-3p | 7 | 178.81 |
| MIMAT0000765 | hsa-mir-335-5p | 7 | 149.33 |
| MIMAT0000680 | hsa-mir-106b-5p | 7 | 122.2 |
| 1915 | EEF1A1 | 6 | 274.26 |
| MIMAT0000083 | hsa-mir-26b-5p | 6 | 160.12 |
| MIMAT0000087 | hsa-mir-30a-5p | 6 | 110.64 |
| MIMAT0003283 | hsa-mir-615-3p | 6 | 97.4 |
| 51429 | SNX9 | 4 | 195.82 |
| 6122 | RPL3 | 3 | 76.25 |
| 1789 | DNMT3B | 3 | 65.51 |
| 399665 | FAM102A | 3 | 38.16 |
| 7045 | TGFBI | 3 | 37.95 |
| 9497 | SLC4A7 | 3 | 37.81 |
| 256051 | ZNF549 | 2 | 49.53 |
| 2305 | FOXM1 | 2 | 29.76 |
| 7097 | TLR2 | 2 | 20.09 |
| 9134 | CCNE2 | 2 | 7.76 |
| 3092 | HIP1 | 2 | 3.43 |
| 6352 | CCL5 | 2 | 3.43 |
| 3921 | RPSA | 2 | 1.42 |
| 63967 | CLSPN | 2 | 1.42 |
| 6188 | RPS3 | 2 | 1.42 |
| 6141 | RPL18 | 1 | 0 |
| 4318 | MMP9 | 1 | 0 |
| 64151 | NCAPG | 1 | 0 |
| 113612 | CYP2U1 | 1 | 0 |
| 9882 | TBC1D4 | 1 | 0 |
| 3945 | LDHB | 1 | 0 |
| 6474 | SHOX2 | 1 | 0 |
| 1028 | CDKN1C | 1 | 0 |
| 3575 | IL7R | 1 | 0 |
| 57405 | SPC25 | 1 | 0 |
| 81567 | TXNDC5 | 1 | 0 |
| 3399 | ID3 | 1 | 0 |
| 4837 | NNMT | 1 | 0 |
| 3437 | IFIT3 | 1 | 0 |
| 8905 | AP1S2 | 1 | 0 |
| 79853 | TM4SF20 | 1 | 0 |
| 4129 | MAOB | 1 | 0 |
| 92293 | TMEM132C | 1 | 0 |
